# Electronic and Magnetic Interactions in 6-Oxoverdazyl Diradicals: Connection through N(1) vs C(3) Revisited

**DOI:** 10.1021/acs.joc.4c00303

**Published:** 2024-04-16

**Authors:** Agnieszka Bodzioch, Emilia Obijalska, Rafał Jakubowski, Małgorzata Celeda, Anita Gardias, Damian Trzybiński, Paweł Tokarz, Jacek Szczytko, Krzysztof Woźniak, Piotr Kaszyński

**Affiliations:** § Centre of Molecular and Macromolecular Studies, Polish Academy of Sciences, 90-363 Łódź, Poland; # Faculty of Chemistry, University of Łódź, 91-403 Łódź, Poland; † Institute of Experimental Physics Faculty of Physics, University of Warsaw, 02-093 Warsaw, Poland; ‡ Biological and Chemical Research Centre, University of Warsaw, 02-089 Warsaw, Poland; ¶ Department of Chemistry, Middle Tennessee State University, Murfreesboro, Tennessee 37132, United States

## Abstract

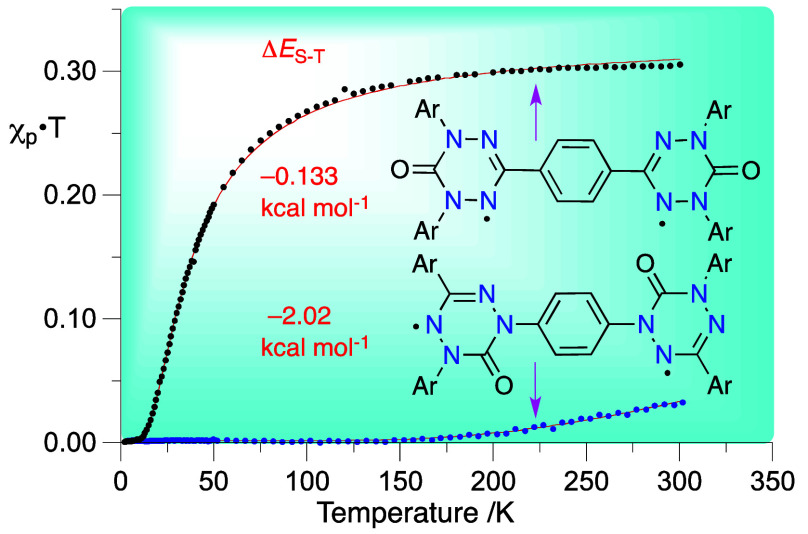

Four isomeric di-6-oxoverdazyl diradicals connected at their N(1) or C(3) positions with either 1,3- or 1,4-phenylene linkers were obtained and characterized by spectroscopic, electrochemical, magnetic, and structural methods. These results were compared to those for the corresponding 6-oxoverdazyl monoradicals. UV–vis spectroscopy demonstrated that only the N(1)-connected *para*-through-benzene diradical has a distinct spectrum with significant bathochromic and hypsochromic shifts relative to the remaining species. Electrochemical analysis revealed two one-electron reduction processes in all diradiacals, while only the N(1)-connected *para*-through-benzene diradical exhibits two one-electron oxidation processes separated by 0.10 V. Variable temperature EPR measurements in polystyrene solid solutions gave negative mean exchange interaction energies *J* for all diradicals, suggesting the dominance of conformers with significant intramolecular antiferromagnetic interactions for the *meta*-through-benzene isomers. DFT calculations predict a small preference for the triplet state with the Δ*E*_S–T_ of about 0.25 kcal mol^–1^ for both *meta*-through-benzene connected diradicals.

## Introduction

There is a rapidly increasing interest in stable topologically coupled multispin organic systems^1−5^ driven by fundamental science and development of functional materials.^6−9^ In particular, robust high-spin diradicals^10−13^ are of importance for organic spintronics^14,15^ (e.g., acting as spin filters)^16−18^ and as building blocks for molecular magnetic materials.^7,19^ On the other hand, low-spin diradicals with weak antiferromagnetic (AFM) exchange interactions are being investigated in the context of triplon excitation in strong magnetic fields and study of critical magnetic phenomena.^20−22^ One such promising group of diradicals with a weakly AFM coupled system is derived from the 6-oxoverdazyl radical,^23,24^ which justifies continuous development of synthetic methods for its preparation and functionalization.^25−27^

**Figure 1:**
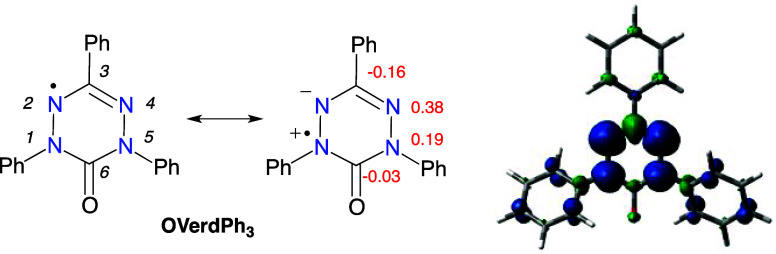
Left: Two resonance forms of the prototypical 6-oxoverdazyl derivative **OxoVerdPh**_**3**_ with indicated numbering scheme and DFT-derived spin density (UB3LYP/6-31G(2d,p) in vacuum). Right: DFT-derived spin density map with the positive density in blue and negative in green.

**Figure 2:**
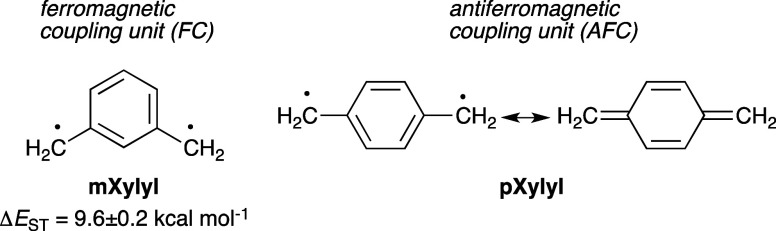
Structure of *m*-xylylene (**mXylyl**) with experimental singlet–triplet energy gap (ref (35)) and *p*-xylylene (**pXylyl**) with a closed-shell (quinoid) resonance structure.

6-Oxoverdazyls^28^ (e.g., 1,3,5-triphenyl-6-oxoverdazyl, **OVerdPh**_**3**_,^29^Figure 1) belong to a family^30^ of robust, π-delocalized heterocyclic radicals with reversible redox behavior^31^ and a broad absorption in the visible range.^32^ Therefore, they have been investigated as convenient spin sources in topologically coupled two-spin systems, in which two verdazyl radicals are connected either directly^33,34^ or through a π-spacer, typically *para*-through-benzene (1,4-phenylene) or *meta*-through-benzene (1,3-phenylene, Figure 2). The latter linker is an effective “ferromagnetic coupler”, FC, while the former acts as a strong antiferromagnetic coupler, AFC.^4^ The strength of the coupling and the singlet–triplet energy gap depend on the spin density at the benzylic positions and is the greatest for the prototypical *meta*-xylylene (**mXylyl**, Δ*E*_S–T_ = 9.6 ± 0.2 kcal mol^–1^, Figure 2),^35^ while *para*-xylylene (**pXylyl**) strongly prefers the closed-shell quinoid form.

Analysis of the prototypical 1,3,5-triphenyl-6-oxoverdazyl radical^29^ (**OVerdPh**_**3**_, Figure 1) indicates that only two different positions can be substituted, the N(1/5) and C(3). The former N(1) and N(5) are symmetry-equivalent positions carrying positive spin density, which can be justified through polar resonance structures (Figure 1). Consequently, the spin density ρ_N1_ of 0.19 calculated for the N(1/5) positions in vacuum is medium polarity-dependent. In contrast, the nodal C(3) position caries negative spin density calculated at ρ_C_ = −0.16. This characteristic of the 6-oxoverdazyl radical determines its use in the design of molecular materials: spin delocalization through Kekulé resonance structures is possible from positions N(1/5),^36^ while spin propagation from the C(3) position takes place mainly through a spin polarization mechanism. The amount of spin transferred to the π substituent depends also on the π–π overlap, and is more effective for coplanar arrangements. In **OVerdPh**_**3**_ (Figure 1), lower interplanar angle and hence higher propensity for spin propagation is calculated for the C(3)-Ph/verdazyl (8.9°) than for the N(1/5)-Ph/verdazyl rings (36.9°). Considering both factors, spin concentration and π–π overlap, and the mechanism for spin exchange interaction, resonance vs polarization, still less spin is transferred to the C(3)–Ph (Σρ = −0.036) than to N(1/5)–Ph (Σρ = 0.046) substituent in the prototypical **OxoVerdPh**_**3**_, as evident from the spin density map in Figure 1.

**Figure 3:**
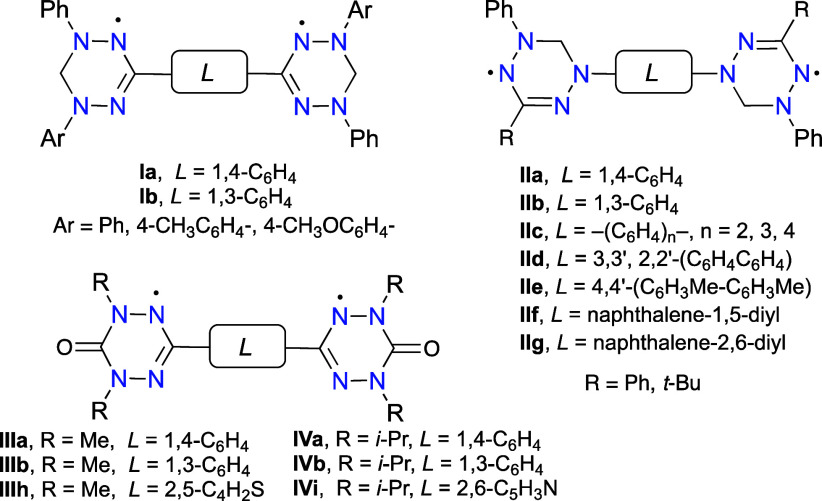
Structures of previously investigated verdazyl and 6-oxoverdazyl diradicals.

Verdazyl diradicals studied until now can be grouped in four general classes shown in Figure 3. Thus, the earliest investigated verdazyl diradicals are those connected at the C(3) position, **Ia** and **Ib**,^36−38^ and several connected at the N(1) position (**II**).^39^ Although these diradicals suffered from low solubility and limited chemical stability, early studies determined significant spin interactions in two diradicals **I** and strong electronic coupling in N-connected diradicals **II**. In the former class of diradicals, the Weiss constant θ_w_ = −100(20) K was determined for **Ia**,^38^ and a triplet ground state was found for **Ib** with an exchange interaction energy of *J* = 28.3 cal mol^–1^ (on the basis of *Ĥ* = −2*JŜ*_1_**·***Ŝ*_2_ Hamiltonian).^40^ In a series of oligophenylene derivatives **II**, exchange interactions were much stronger, with *J* > −1.5 kcal mol^–1^ for **IIa**, *J* = −0.3 kcal mol^–1^ for **IIc** (n = 2), *J* = −0.05 kcal mol^–1^ for **IIc** (n = 3), and *J* = −0.75 kcal mol^–1^ for **IIg**.^39^ For other derivatives in series **II** (**IId**–**IIf**), it was concluded that the singlet and triplet states are nearly degenerate^39^ due to poor π–π overlap and insufficient interactions between the spin sites. It should be noted that these studies did not include diradical **IIb** containing the 1,3-phenylene linker, for which a triplet ground state might be expected.

Investigation of electronic and spin communication in 6-oxoverdazyl diradicals concentrated only on derivatives **III** and **IV** connected at the nodal C(3) position, presumably due to synthetic constrains (Figure 3). Thus, studies of diradicals **III** revealed that the *para*-through-benzene connection in **IIIa** leads to greater electronic communication between the radical centers than in the *meta* isomer **IIIb** (based on UV), but variable temperature electron paramagnetic resonance spectroscopy (VT EPR) analysis of frozen solutions of **III** gave only a linear temperature dependence, and exchange interactions *J* could not be determined.^41^ A decade later, detailed spectroscopic, electrochemical, and magnetic investigation of more chemically robust *i*-propyl analogues **IVa** and **IVb** demonstrated the extent of electronic and magnetic interactions between the 6-oxoverdazyl units mediated by the benzene ring with exchange interactions *J* = −43.3(20) cal mol^–1^ and *J* = 27.6(25) cal mol^–1^, respectively.^42^ Similar results were obtained for the 2,6-pyridinediyl-connected 6-oxoverdazyl radical **IVi**, for which ferromagnetic exchange interaction *J* = 114 cal mol^–1^ was obtained.^43^ Since the spin delocalization in **OVerdPh**_**3**_ is greater for the N(1/5)–Ph than the C(3)–Ph group, larger exchange interaction energy values *J* are expected for diradicals connected through the N(1) than through the C(3) position in the 6-oxoverdazyl. A set of isomers to test this hypothesis was not available until now.

**Figure 4:**
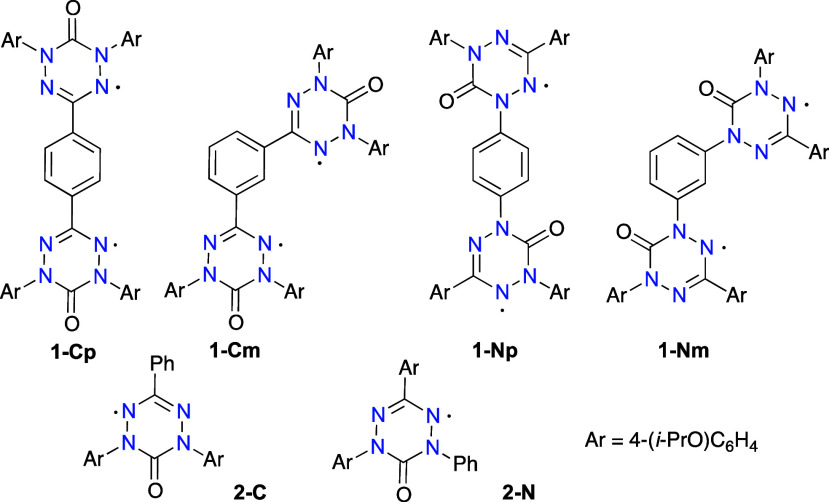
Structures of diradicals **1** and reference radicals **2**.

Prompted by our interest in paramagnetic self-organizing materials derived from 6-oxoverdazyl,^44−49^ we focused on an isomeric series **1** (Figure 4), as potential structural elements of high-spin liquid crystals.^50^ The series represents a unique set of isomers in which the connection site (N(1) vs C(3)) and the nature of the connector (*m*-phenylene vs *p*-phenylene) are systematically varied. A similar series of isomers was investigated previously using only computational methods.^51^ For solubility reasons, the phenyl groups are substituted with the *i*-propoxy groups.

Herein, we report the synthesis and comprehensive characterization of diradicals **1**, in which two 6-oxoverdazyl units are connected at the C(3) (**1-C**) or N(1) (**1-N**) positions with a 1,4- or 1,3-phenylene bridge. For a better understanding of intramolecular interactions in series **1**, *C*-phenyl and *N*-phenyl substituted 6-oxoverdazyl monoradicals **2-C** and **2-N** were also prepared and analyzed. Electronic communication in the diradicals was probed with UV–vis spectroscopy and E-chem analysis, while magnetic properties were investigated with VT EPR spectroscopy and SQUID magnetometry. Analysis of series **1** and **2** was supported with two crystal structures and extensive DFT calculations.

## Results and Discussion

### Synthesis

**Scheme 1:**
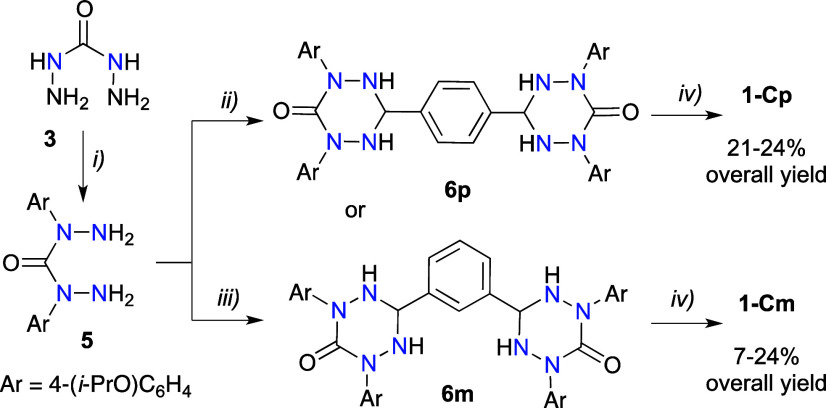
Synthesis of C(3)-Connected Di-6-oxoverdazyls **1-Cp** and **1-Cm** Reagents and conditions: (i) 4-iodo-1-(*i*-propoxy)benzene (**4**), CuI, 1,10-phenanthroline, K_3_PO_4_, DMF, 90 °C, 3 h, 54% yield; (ii) terephthalaldehyde, EtOH, reflux, 8 h, 78% yield; (iii) isophthalaldehyde, EtOH, reflux, 8 h, 82% yield; (iv) NaIO_4_, [Bu_4_N]^+^Br^–^ (cat.), CH_2_Cl_2_/MeOH (1:1), rt, 12 h, 47–54% yield for **1-Cp** and 17–60% yield for **1-Cm**.

The synthesis of diradicals **1-Cp** and **1-Cm**, in which two 6-oxoverdazyl units are connected at the C(3) positions through a phenylene spacer, followed a general procedure^42^ and was accomplished in three steps and 21–24% and 7–24% overall yields, respectively, based on carbohydrazide (**3**, Scheme 1). Thus, Ullmann arylation of **3** with 4-iodo-1-(*i*-propoxy)benzene (**4**) under general literature conditions^52^ gave *N,N*′-diaryl derivative **5** isolated in 54% yield. Condensation of the hydrazide **5** with terephthalaldehyde and isophthalaldehyde gave bis-tetrazanes **6p** and **6m** in yields 78% and 82%, respectively. The resulting bis-tetrazanes **6** were oxidized with NaIO_4_ in the presence of [Bu_4_N]^+^Br^–^,^27^ and the desired bis-oxoverdazyls **1-Cp** and **1-Cm** were isolated in yields up to about 50% and 60%, respectively, after 12 h. Extension of the reaction time to 48 h led to a complex mixture of products, from which the desired radical **1-Cm** was isolated only in 17% yield.

**Scheme 2:**
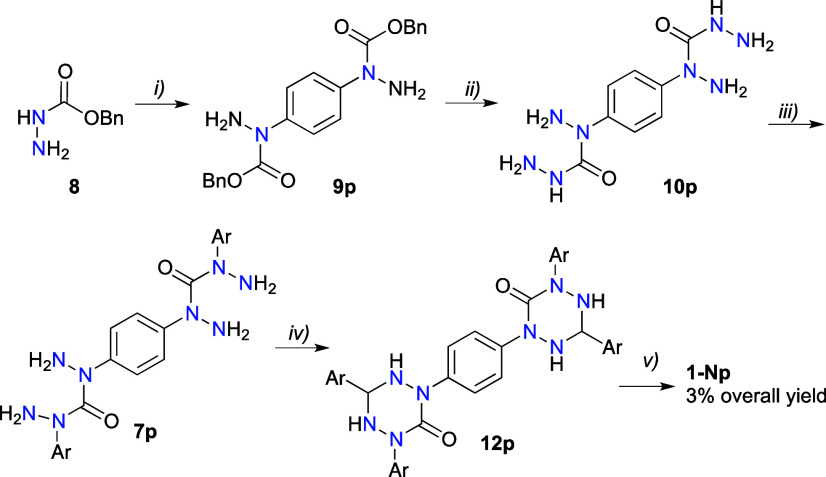
Synthesis of N(1)-Connected Di-6-oxoverdazyl **1-Np** Reagents and conditions: (i) 1,4-diiodobenzene, CuI, 1,10-phenanthroline, Cs_2_CO_3_, DMF, 90 °C, 5 h, 78% yield; (ii) hydrazine monohydrate, 100 °C, 1 h, 38% yield; (iii) 4-iodo-1-(*i*-propoxy)benzene (**4**), CuI, K_3_PO_4_, DMSO, 80 °C, 24 h, 60% yield or CuI, Cs_2_CO_3_, DMSO, 50 °C, 24 h, 50% yield; (iv) 4-(*i*-propoxy)benzaldehyde (**11**), EtOH, reflux, 8 h, 48–56% yield; (v) NaIO_4_, [Bu_4_N]^+^Br^–^ (cat.), CH_2_Cl_2_/MeOH (1:1), rt, 6 h, 31% yield.

In contrast to diradicals **1-C**, synthesis of the isomeric N(1)-substituted bis-oxoverdazyls **1-Np** and **1-Nm** was much more complicated and required differentiation of the N(1) and N(5) positions in the verdazyl skeleton. The strategy used for the preparation of **1-C**, involving condensation of an aldehyde with a substituted carbohydrazide, was adopted for the preparation of **1-Np** diradical. Thus, the requisite bis-carbohydrazide **7p** was obtained in three steps starting with arylation of benzyl carbazate (**8**) with 1,4-diiodobenzene in the presence of CuI, 1,10-phenanthroline, and Cs_2_CO_3_ in DMF (Scheme 2), according to a modified general procedure.^52^ The resulting bis-hydrazide **9p** was isolated in 78% yield by extraction from the crude reaction mixture followed by chromatography. The product was subjected to hydrazinolysis in neat hydrazine monohydrate at 100 °C. The desired biscarbohydrazide **10p** was isolated in 38% after 1 h by filtration of the reaction mixture and washing the solid product with H_2_O, MeOH, and finally with Et_2_O. Extension of the reaction time to 3 h did not affect the yield. Attempts at using EtOH as the reaction medium gave only traces of product **10p**.

N-arylation of the bis-carbohydrazide **10p** with iodide **4**, using conditions for the preparation of **9p**, gave **7p** in low and irreproducible yields (Scheme 2). A brief investigation^53^ demonstrated that the N-arylation of **10p** catalyzed with CuI takes place efficiently in DMSO at 50 °C in the presence of either Cs_2_CO_3_ or K_3_PO_4_ as a base and without phenanthroline.^54^ The desired arylation product **7p** was obtained from **10p** in 50% yield, using Cs_2_CO_3_, or 60% yield using K_3_PO_4_. Pure **7p** was isolated by removal of DMSO in vacuum and direct chromatography of the resulting viscous residue. Extraction, as means of partial purification of the crude product, was ineffective.

Condensation of **7p** with 4-(*i*-propoxy)benzaldehyde (**11**) gave the desired bis-tetrazane **12p** in 48–56% yield, which was subsequently oxidized with NaIO_4_ to bis-oxoverdazyl **1-Np** (Scheme 2). TLC monitoring of the progress of the oxidation reaction demonstrated that the optimum reaction time was 6 h, and the diradical **1-Np** was isolated in 31% yield from bis-tetrazane **12p** or 3% overall yield based on carbazate **8**. Elongation of the reaction time to 24 h resulted in significant decomposition and, consequently, lower yields of the isolated product.

**Scheme 3:**
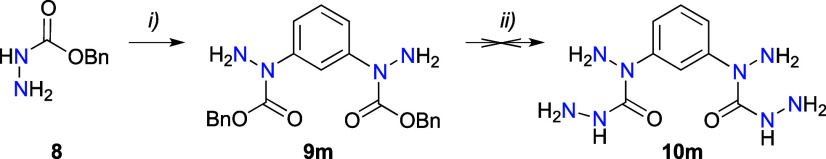
Attempted Synthesis of Bis-carbohydrazide **10m** Reagents and conditions: (i) 1,3-diiodobenzene, CuI, 1,10-phenanthroline, Cs_2_CO_3_, DMF, 90 °C, 5 h, 71% yield; (ii) hydrazine monohydrate, 100 °C, 1 h.

The strategy used for the preparation of **1-Np** failed for synthesis of the *meta* isomer **1-Nm**, since the required intermediate, bis-carbohydrazide **10m**, could not be obtained. While N-arylation of carbazate **8** with 1,3-diiodobenzene, using the condition for the preparation of **9p**, worked well and the bis-hydrazide **9m** was obtained in 71% yield, the subsequent hydrazinolysis did not give the expected **10m** (Scheme 3). Thus, heating of **9m** at 100 °C with hydrazine monohydrate for 15 min gave benzyl alcohol as the only isolated organic product. Similar results were obtained in hydrazinolysis of **9m** in refluxing MeOH over 24 h. This suggests extensive fragmentation of **9m**, while the relatively efficient preparation of the *para* isomer **10p** is presumably due to its low solubility and fortuitous precipitation from the reaction mixture preventing it from further reactions. In an attempt at selective activation of the RO group in the bis-carbazate **9m** toward replacement with hydrazine, substitution of the benzyl group with phenyl was considered. Unfortunately, N-arylation of phenyl hydrazinecarboxylate with 1,3-diiodobenzene in the presence^52^ of CuI, 1,10-phenanthroline, and Cs_2_CO_3_ in hot DMF gave a complex mixture of products from which only phenol was isolated and identified by NMR.

**Scheme 4:**
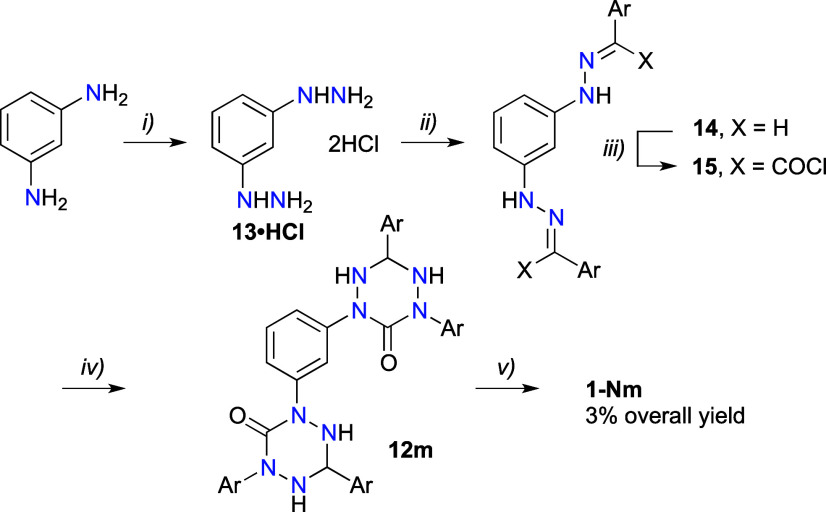
Synthesis of Di-6-oxoverdazyl **1-Nm** Reagents and conditions: (i) 1. NaNO_2_, HCl, −5 °C, 45 min; 2. SnCl_2_, HCl, 45% yield;^11^ (ii) 4-(*i*-propoxy)benzaldehyde (**11**), AcONa, H_2_O, rt, 30 min, 70% yield; (iii) triphosgene, Et_3_N, CH_2_Cl_2_, 0 °C, 1 h, then rt, 30 min; (iv) 4-(*i*-propoxy)phenylhydrazine hydrochloride (**16·HCl**), Et_3_N, EtOH, reflux, 12 h, 26% overall yield; (v) NaIO_4_, [Bu_4_N]^+^Br^–^ (cat.), CH_2_Cl_2_/MeOH (1:1), rt, 6 h, 39% yield.

An alternative synthetic path to bis-oxoverdazyl **1-Nm** was offered by the recently described 1,3-phenylenedihydrazine (**13**),^11^ which could be used in the general Milcent method^55^ for synthesis of 6-oxoverdazyls. Thus, a condensation reaction of crude dihydrazine dihydrochloride^11^**13·HCl** (45–75% pure by NMR) with 2 equiv of 4-(*i*-propoxy)benzaldehyde (**11**) in water in the presence of AcONa afforded bis-hydrazone **14** in 70% yield (Scheme 4). The reaction was taking place in neat droplets of the aldehyde. Attempts at purifying this bishydrazone **14** or conducting the condensation reaction in an organic solvent (e.g., EtOH) gave only decomposition products. Treatment of crude **14** with triphosgene gave the corresponding biscarbamoyl chloride **15**, which was used without purification in a reaction with 4-(*i*-propoxy)phenylhydrazine dihydrochloride (**16·HCl**). The resulting bis-tetrazane **12m**, obtained in 26% yield, was then oxidized with NaIO_4_, giving the desired bis-oxoverdazyl **1-Nm** in 39% yield. A similar strategy could be used for the preparation of **1-Np** taking advantage of the recently reported benzene-1,4-didiazonium salt.^56^ The overall yield for diradicals **1-N** is about 3%, which reflects more synthetic steps with lower yields and lower stability of the diradicals, particularly the *meta* isomer **1N-m**, when compered to **1C**.

**Scheme 5:**
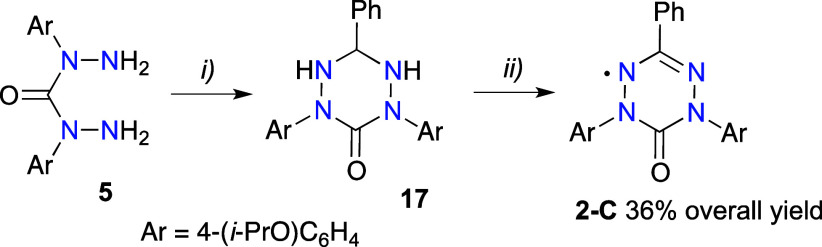
Synthesis of 6-Oxoverdazyl **2-C** Reagents and conditions: (i) PhCHO, EtOH, reflux, 6 h, 59% yield; (ii) NaIO_4_, [Bu_4_N]^+^Br^–^ (cat.), CH_2_Cl_2_/MeOH (1:1), rt, 6 h, 63% yield.

For comparison purposes, C(3) and N(1) Ph-substituted monoradicals **2** were also synthesized. Thus, C(3)-phenyl-6-oxoverdazyl **2-C** was obtained in 36% overall yield in a two-step synthesis involving reaction of bis-hydrazide **5** with PhCHO followed by oxidation of the resulting tetrazane **17** with NaIO_4_ (Scheme 5).

**Scheme 6:**
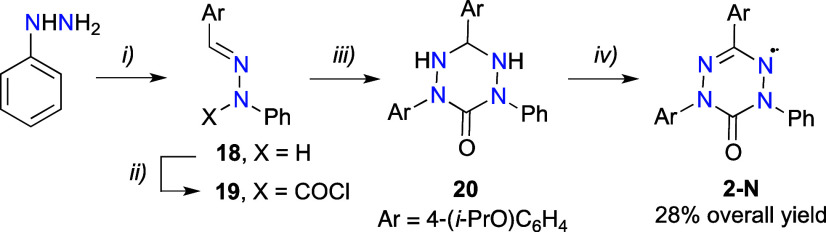
Synthesis of 6-Oxoverdazyl **2-N** Reagents and conditions: (i) 4-(*i*-propoxy)benzaldehyde (**11**), EtOH, AcOH (cat.), rt, 10 h, 79% yield; (ii) triphosgene, Et_3_N, CH_2_Cl_2_, 0 °C, 1 h then rt, 30 min, 90% yield; (iii) 4-(*i*-propoxy)phenylhydrazine hydrochloride (**16·HCl**), Et_3_N, EtOH, reflux, 10 h, 60% yield; (iv) NaIO_4_, [Bu_4_N]^+^Br^–^ (cat.), CH_2_Cl_2_/MeOH (1:1), rt, 6 h, 66% yield.

Synthesis of the isomeric N(1)-phenyl substituted 6-oxoverdazyl **2-N** took advantage of the classical Milcent method,^55^ and it was accomplished in four steps and 28% overall yield (Scheme 6). Thus, a reaction of phenylhydrazine and aldehyde **11** gave hydrazone **18** in 79% yield, which upon treatment with triphosgene afforded the carbamoyl chloride **19**. Condensation of the chloride with hydrochloride **16·HCl** gave tetrazane **20** in 60% yield, which was oxidized with NaIO_4_ to give the desired 6-oxoverdazyl **2-N** isolated in 66% yield.

All mono- and diradicals were freshly purified before measurements.

### Molecular and Crystal Structures

**Figure 5:**
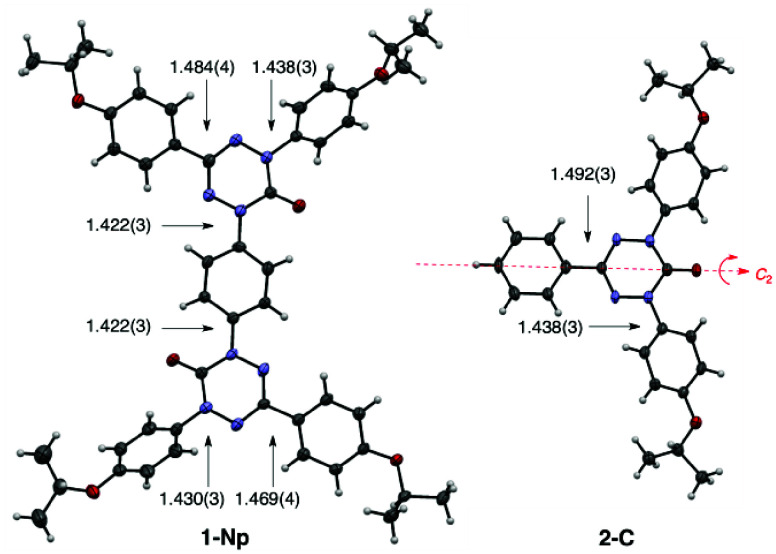
Molecular structures of **1-Np** (left) and **2-C** (right) with pertinent distances (Å). Displacement ellipsoids are drawn at 50% probability level. Hydrogen atoms are shown as small spheres of arbitrary radius. Color code: C black, N blue, O red, H gray. For full geometrical parameters, see the SI.

**Figure 6:**
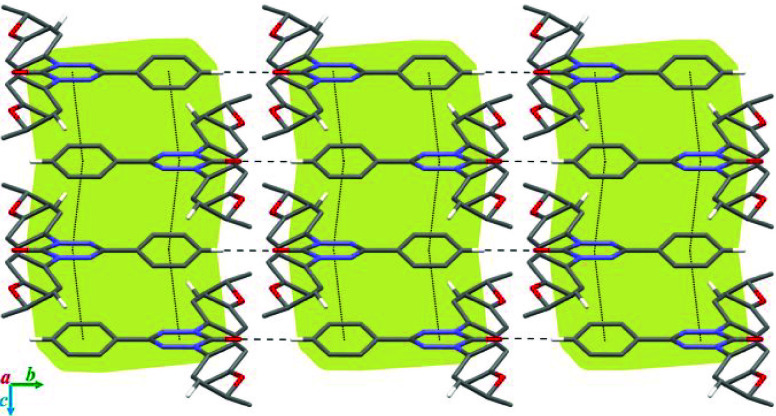
Supramolecular architecture of the crystal of **2-C**: 2D sheet formed by H-bonded stacks of molecules viewed from the side (single stacks are highlighted in green). The dashed lines indicate the C–H···O hydrogen bonds, and the dotted lines connecting ring geometrical centers show π–π contacts. The H atoms not involved in the intermolecular interactions are omitted for clarity. Color code: C black, N blue, O red, H gray.

**Figure 7:**
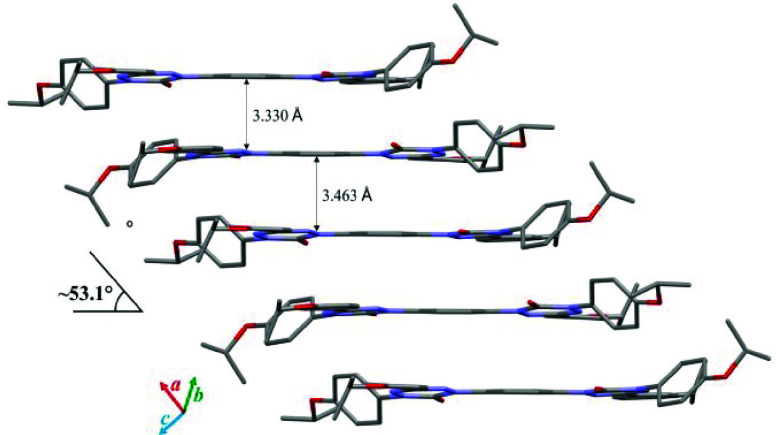
A slipped stack of **1-Np** with indicated alternating distances, defined by the central benzene ring, and the slippage angle. Hydrogen atoms are omitted for clarity. Color code: C black, N blue, O red.

For a better understanding of magnetic properties of radicals **1** and **2**, their solid-state structures were investigated. Thus, black-red crystals of **1-Np** and **2-C** were obtained by slow evaporation of heptane/CH_2_Cl_2_ and CH_2_Cl_2_/hexane solutions, respectively. Their solid-state structures were determined by single-crystal X-ray diffraction analysis and results are shown in Figures 5–7. Full details of crystallographic data collection, refinement, and analysis are provided in the SI. Attempts at growing crystals of **1-Cp**, **1-Cm**, **1-Nm**, and **2-N** suitable for XRD analysis from heptane/CH_2_Cl_2_, CH_2_Cl_2_/hexane, or CH_2_Cl_2_/MeCN solutions or by solvent diffusion methods were unsuccessful.

The compounds crystallize in the orthorhombic P*bcn* (**2-C**) or triclinic *P*-1 (**1-Np**) space group with a half (**2-C**) or one (**1-Np**) molecule in the asymmetric unit of the crystal lattice. The molecular structures are shown in Figure 5, while full geometrical parameters are listed in the Supporting Information.

The verdazyl rings in both compounds are essentially planar, and geometrical parameters are similar to those found in other 6-oxoverdazyl derivatives.^29,57^ Radical **2-C** has a 2-fold axis defined by the O–C(6)···C(3) atoms of the 6-oxoverdazyl ring (Figure 5). The C(3)–Ph bond (1.492 Å) is longer than the N–C_6_H_4_OPr-*i* (1.438(3) Å). The C(3)–Ph and the N–C_6_H_4_OPr-*i* ring planes form with the verdazyl heterocycle angles of 18.41(12)° and 33.70(7)°, respectively. All these values are typical for aryl derivatives of the 6-oxoverdazyl.

Molecular dimensions in diradical **1-Np** are also typical, albeit with a few exceptions. Thus, the central benzene ring is connected to the 6-oxoverdazyl with the N–Ph bond shorter than that in the monoradical (1.422(3) Å in **1-Np** vs 1.438(3) Å in **2-C**), and the two central benzene/oxoverdazyl interplanar angles are unusually low, 5.39(8)° and 8.35(8)°, when compared to that in, e.g., **2-C** (33.70(7)°). Both of these structural features are consistent with significantly enhanced electronic communication between the two π systems, which requires planarization and results in bond shortening, as corroborated with DFT results (*vide infra*). On the other hand, the N–C_6_H_4_OPr-*i* torsion angles are significantly higher than typical (oxoverdazyl/aryl interplanar angles are 48.94(9)° and 53.16(9)°), while the C(3)–C_6_H_4_OPr-*i* torsion angles are small (8.49(8)° and 1.99(8)°) and within the usual limits. The large distribution of torsion angle values observed in the solid-state structure of **1-Np** indicates a significant conformational flexibility of this and other diradicals, which has an impact on magnetic properties of the solids.

The crystal structure of **2-C** consists of infinite chains of C–H···O hydrogen bonded molecules running along the [010] direction (Figure 6). The individual chains are arranged antiparallel in stacks propagating along the [001] direction, which leads to the formation of a 2D sheet parallel to the (100) plane. The 6-oxoverdazyl and C–Ph rings of the neighboring molecules form an angle of 18.41(10)° and interact through weak π–π contacts defined by the distance of 3.938(2) Å between their geometrical centers. The entire crystal structure is stabilized by weak C–H···π contacts between the *i*-propoxy groups and benzene rings of neighboring molecules in the adjacent sheets. A view of the crystal structure of **2-C** along the *b*-direction reveals the characteristic “herringbone” supramolecular architecture, in which the mean molecular planes in adjacent sheets form an angle of about 144.5° (SI).

In contrast to **2-C**, molecules of **1-Np** form slipped stacks with alternating distances of 3.330 and 3.463 Å between planes, defined by the central benzene rings and the slippage angle of 53.1° (Figure 7). All molecules in the stack exhibit π–π interactions between the central benzene ring and the 6-oxoverdazyl heterocycle. In the discrete dimer, mutual nonbonding close contacts of 3.288 Å are observed between C(1) of the benzene ring and C(3) of the 6-oxoverdazyl, which are 0.112 Å inside the van der Waals (VDW) separation. Neighboring dimers in the stacks exhibit weak C–H···O=C hydrogen bonds of 2.399 Å (0.321 Å inside the VDW separation) involving the N(5)-benzene ring position of one of the two 6-oxoverdazyls. The second carbonyl group is involved in the interstack interaction with a CH fragment of an *i*-PrO group (2.570 Å, 0.150 Å inside the VDW separation). The stacks run parallel to each other and interact through a layer of *i*-PrO···PhOPr-*i* close contacts.

**Figure 8:**
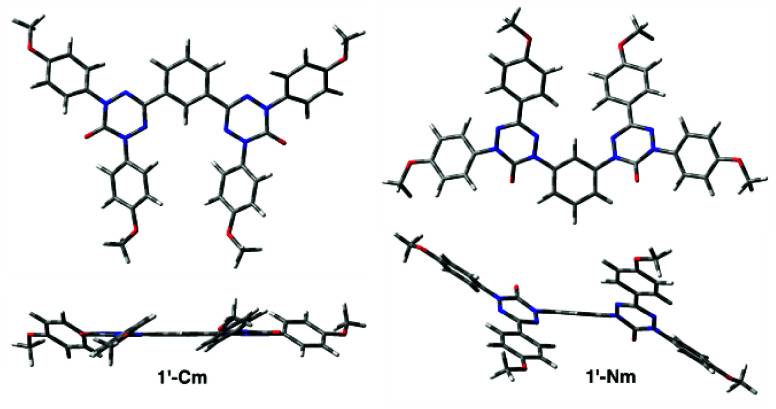
Two views of models **1′-Cm** (left) and **1′-Nm** (right) with geometries fully optimized in the triplet state in the gas phase at the UB3LYP/6-31G(2d,p) level of theory.

Since experimental molecular structures of other diradicals could not be obtained, for comparison purposes molecular structures of models of all four diradicals **1** and two prototypical monoradicals **2**, in which the *i*-PrO groups were replaced with OMe, were obtained at the UB3LYP/6-31G(2d,p) level of theory. The molecules were optimized without symmetry constraints in vacuum, and the lowest energy conformers were considered further. Their equilibrium geometry was close to the *C*_2_ point group symmetry for all except for **1′-Cp** (*D*_2_ symmetry) and **2′-N** (*C*_1_ symmetry). The results are shown in Table 1 and Figure 8.

**Table 1:** Key Structural Data for Model Diradicals **1′** and Monoradicals **2′**^a^

radical	spin state	symm	X–Ph^b^/Å	θ^c^/°	ω^d^/°
**2′-C**	D	*C*_2_	1.4847	9.31	36.25
			1.492(3)^e^	8.4(1)^e^	33.70(7)^e^
**1′-Cp**	OSS	*D*_2_	1.4828	4.7	36.3
**1′-Cm**	T	*C*_2_	1.4860	2.9	36.5, 32.6
**2′-N**	D	*C*_1_	1.4333	36.0	36.5, 7.6
**1′-Np**	OSS	*C*_2_	1.4285	31.7	37.7, 8.6
			1.422(3)^e^	5.4, 8.4^e^	51, 5.2^e^^,^^f^
**1′-Nm**	T	*C*_2_	1.4319	34.0	36.2, 1.25

a UB3LYP/6-31G(2d,p) in vacuum for model with RO = OMe. D–doublet, OSS–open shell singlet, T–triplet.

b X = C for the **C** series and X = N for the **N** series.

c Angle between Ph and the 6-oxoverdazyl planes.

d Angle between MeOC_6_H_4_ and the 6-oxoverdazyl planes.

e Experimental values.

f Average of two values.

DFT calculations reproduced well the main geometrical features of the experimental molecular structures (Table 1). Thus, both experiment and DFT demonstrate the *C*_2_ point group symmetry for **2-C**. Also, the aromatic core of **1-Np** nearly conforms to the *C*_2_ symmetry in the XRD structure. The biggest differences between the gas-phase DFT optimized geometry and the experiment is in the torsion angles between the central 1,4-phenylene ring and the 6-oxoverdazyl unit in **1-Np**: while the theory predicts about 32°, experiment shows the torsion angle of about 7°. This significant planarization of the core strengthens electronic interactions between the two π-systems, which results in the short N–Ph bond (exp. 1.422(2) Å, DFT 1.4285 Å). On the other hand, the torsion around the (4-*i*-PrOC_6_H_4_)–N bond is about 15° higher in the XRD structure than predicted by theory, but it has marginal impact on the diradical’s spin state.

Analysis of DFT structural data in Table 1 indicates that connection of two prototypical radicals **2′-C** shortens the C(3)–Ph bond by 0.002 Å in the *para*-through-benzene-connected **1′-Cp** and slightly expands it by about 0.001 Å in *meta*-through-benzene-connected **1′-Cm**. At the same time, the torsion angle θ associated with the C(3)–Ph bond is lowered from 9.3° to 4.7° and 2.9°, respectively.

**Figure 9:**
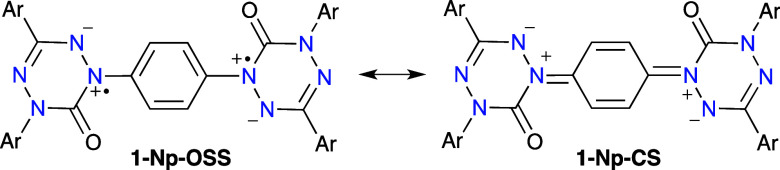
Two resonance structures for **1-Np** showing open-shell (OSS) and closed-shell (CS) structures.

The same analysis for the “N-series” indicates a significant shortening of the N–Ph bond in **2′-N** by 0.005 Å in the *para* diradical **1′-Np**, which compares to 0.001 Å shortening in the *meta* isomer **1′-Nm**. The bond shortening is paralleled by the decrease of the torsion angle between the Ph and the 6-oxoverdazyl by about 4° in **1′-Np** and by much less (∼2°) in **1-Nm**. These trends are consistent with the tendency for an effective π–π overlap and electron pairing, as particularly apparent in the Kekulé closed-shell structure **1-Np-CS** shown in Figure 9.

### Electronic Absorption Spectroscopy

**Figure 10:**
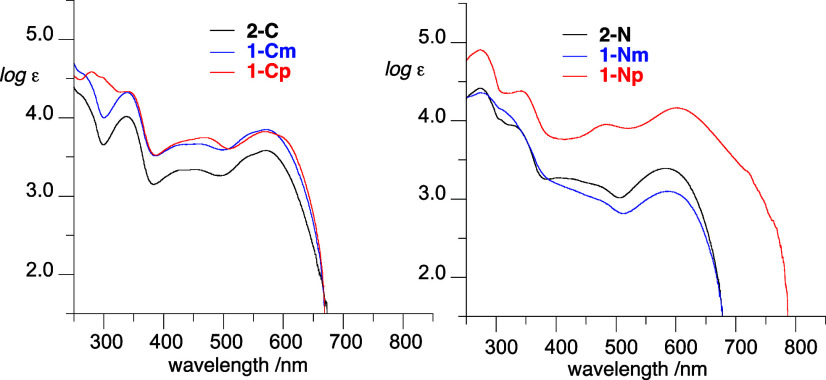
UV–vis absorption spectra for series **C** (left) and series **N** (right) in CH_2_Cl_2_.

**Figure 11:**
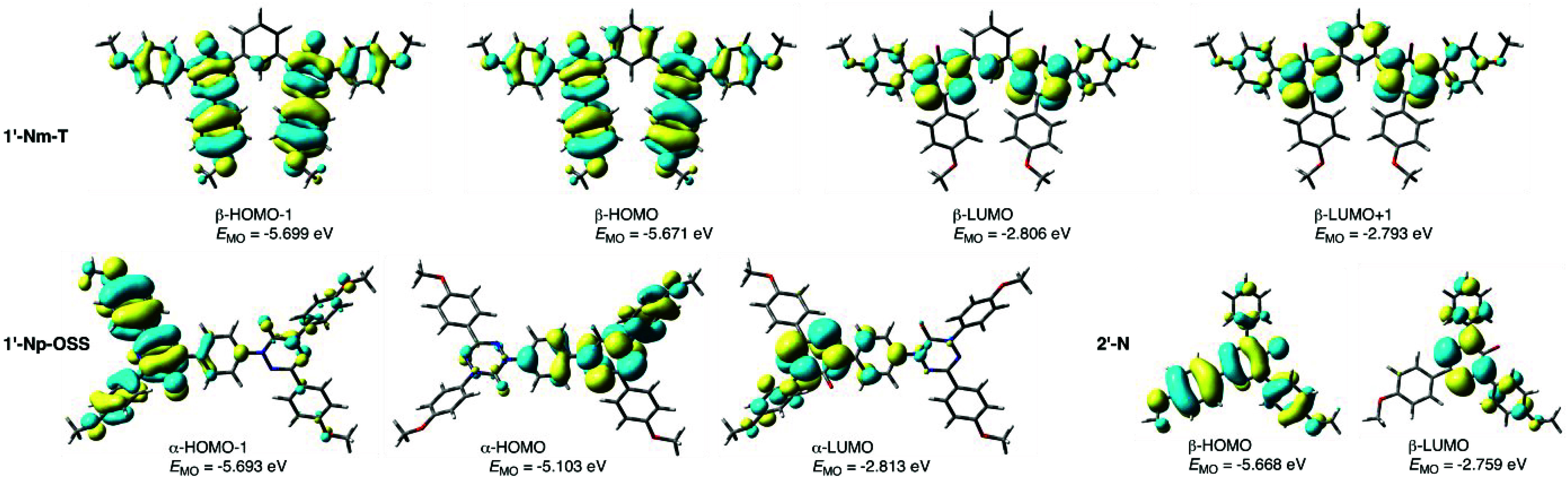
Contours and energies of MOs relevant to low-energy excitations in the triplet **1′-Nm-T**, open-shell singlet **1′-Np-OSS**, and **2′-N** calculated with UB3LYP/6-31G(2d,p)//UB3LYP/6-31G(2d,p) level of theory in CH_2_Cl_2_ dielectric medium. MO isovalue = 0.02, density = 0.0004.

The effect of connection of two oxoverdazyl fragments through a phenylene linker on electronic properties was investigated with absorption spectroscopy in CH_2_Cl_2_ solutions. The two isomeric monoradicals **2-C** and **2-N** exhibit broad absorption bands in the visible range, with the lowest energy maximum at 571 nm (log ε = 3.58) for the former and 583 nm (log ε = 3.39) for the latter radical (Figure 10, Table 2). On the basis of TD-DFT calculations for models **1′** and **2′**, this absorption band can be ascribed solely to the β-HOMO → β-LUMO transition (about 90%) calculated at 568 nm (*f* = 0.138) for **2′-C** and 599 nm (*f* = 0.067) for **2′-N** (Figure 11). The lower excitation energy in the latter radical is related to the calculated higher energy of its β-HOMO (by 97 meV) and consequently lower HOMO–LUMO gap in **2′-N** (by 116 meV) when compared to **2′-C** (Table 2).

**Table 2:** Selected Experimental and Calculated Electronic Parameters for Diradicals **1** and Monoradicals **2**

radical	exp λ_max_^a^/nm	λ_max_ (*f*)^b^^,^^c^/nm	*E*_g_(opt)^d^/eV	*E*_β-HOMO_^b^/eV	*E*_β-LUMO_^b^/eV
**2-C**	571	568 (0.138)^e^	1.94	–5.765	–2.740
**1-Cm**	571	568 (0.061)^e^	1.93	–5.748	–2.762
		565 (0.209)^f^			
**1-Cp**	569	570 (0.243)^g^	1.91	–5.021	–2.755
**2-N**	583	599 (0.067)^e^	1.88	–5.668	–2.759
**1-Nm**	587	607.5 (0.147)^e^	1.86	–5.671	–2.806
**1-Np**	601	660 (0.361)^f^	1.76	–5.103	–2.813

a Maximum of the lowest energy absorption band (recorded in CH_2_Cl_2_).

b Obtained with TD-UB3LYP/6-31G(2d,p) level of theory in CH_2_Cl_2_ dielectric medium for model radicals in T (*meta*) or OSS (*para*) state.

c Lowest energy excitation with significant oscillator strength (*f*).

d Optical band gap determined from the onset of absorption, λ_onset_.

e State 1.

f State 2.

g State 3.

Connecting two 6-oxoverdazyls at the C(3)-positions with a phenylene linker results in a small hyperchromic increase (log ε ∼ 3.85) essentially without affecting the position of the maximum of absorption (Figure 10). In contrast, connecting two oxoverdazyl radicals at the N(1) positions causes a modest bathochromic shift (Δλ = +4 nm) in the *meta* isomer **1-Nm** and a significantly larger shift, Δλ = +18 nm, in the *para* isomer **1-Np**. In addition, spectrum of the latter diradical exhibits a significant hyperchromic increase and a broad shoulder, which moves the absorption edge from 684 nm in **2-N** and **1-Nm** to 789 nm in **1-Np** (Figure 10). These results indicate strong electronic interactions only in **1-Np** diradical, modest in **1-Cp**, and essentially none in the *meta* isomers. They are consistent with results found for phenylene derivatives in series **I** and **II**.^36,39^

Analysis of the absorption onset indicates higher optical band gap *E*_g_(opt) in series **C** than in series **N** by about 0.06 eV, and a relatively little change of *E*_g_(opt) in series **C** (from 1.94 eV in **2-C** to 1.91 eV in **1-Cp**). In series **N**, however, the *E*_g_(opt) value for the *meta* isomer **1-Nm** is slightly lower (by 0.02 eV) relative to the monoradical **2-N**, but for the *para* isomer **1-Np** the optical band gap is markedly narrower by 0.12 eV (Table 2).

The experimentally observed trends in the position and intensity of the low-energy absorption maxima in series **1** and **2** are well reproduced by TD-DFT calculations (Table 2). Thus, the lowest-energy excitations in model diradicals **1′** are close to those for the corresponding parent monoradical **2′**, except for that of open-shell singlet (OSS) **1′-Np-OSS**, which has a 0.19 eV (60 nm) lower energy and is more intense. TD-DFT modeling revealed that the lowest-energy excitation in the triplet *meta* isomers involves nearly exclusively the β electron manifold, with the dominant β-HOMO → β-LUMO (∼55%) and β-HOMO–1 → β-LUMO+1 (∼40%) transitions in state 1 (Figure 11). In state 2 of triplet form **1′-Cm-T** with higher oscillator strength (*f* = 0.209 vs 0.061 for state 1), the excitation involves mainly the β-HOMO → β-LUMO+1 (54%) and β-HOMO–1 → β-LUMO (36%) transitions.

A similar analysis of the *para* OSS diradicals demonstrated essentially equal participation from both electron manifolds in the lowest-energy excitations with significant oscillator strengths. Thus, in the open-shell singlet **1′-Cp-OSS**, state 3 excitation (569.6 nm, *f* = 0.243) involves mainly (85%) the α-HOMO–1 → α-LUMO and β-HOMO–1 → β-LUMO transitions, while in **1′-Np-OSS** (state 2, 659.9 nm, *f* = 0.361) there are two main components: α-HOMO → α-LUMO and β-HOMO → β-LUMO (72%) and a pair of α-HOMO–1 → α-LUMO and β-HOMO–1 → β-LUMO (20%) transitions.

Analysis of the MO density revealed that low-energy excitations in the *meta* diradicals are qualitatively similar to those of the monoradicals, as shown in Figure 11 for the **N** series. Thus, in both, triplet **1′-Nm-T** and **2′-N** the active β-HOMO is delocalized mainly over the 3-(4-ROC_6_H_4_)verdazyl fragment, while the β-LUMO is localized on the nitrogen atoms of the 6-oxoverdazyl heterocycle. In the *para* diradicals, the situation is different and the main transition involves the HOMO, localized on the 5-(4-ROC_6_H_4_)verdazyl of one spin unit, to the LUMO of the other spin unit. Such transitions are characteristic for diradical/diradicaloid systems.

### Electrochemical Characterization

Cyclic voltammetry (CV) studies of monoradicals **2-C** and **2-N** revealed the expected^31^ quasireversible redox processes with a difference between them of about 1.44 V (Table 3). The oxidation potential is shifted anodically by about 0.05 V in the latter relative to that in the **2-C** isomer, which is consistent with swapping the Ph and electron-donating 4-ROC_6_H_4_ groups at the N(1) and nodal C(3) positions, and consequently, lowering the HOMO energy in **2-N** by 75 meV.

**Table 3:** Electrochemical Data for Diradicals **1** and Radicals **2**^a^

radical	*E*_1/2_^2–/–^ /V	*E*_1/2_^–/0^/V	*E*_1/2_^0/+^/V	*E*_1/2_^+/2+^/V	*E*_cell_^b^/V
**1-Cm**	–1.37	–1.14	0.33^c^		1.47
**1-Cp**	–1.30	–1.10	0.32^c^		1.42
**2-C**	–	–1.13	0.31	–	1.44
**1-Nm**	–1.16	–1.03	0.39^c^		1.42
**1-Np**	–1.20	–1.03	0.36	0.45	1.39
**2-N**	–	–1.08	0.35	–	1.43

a Measured in CH_2_Cl_2_ [*n*-Bu_4_N]^+^[PF_6_]^−^(50 mM), ca. 20 °C, 50 mV s^–1^, glassy carbon electrode. Potentials vs Fc/Fc^+^ (0.46 V vs SCE; ref (58)) determined from differential pulse voltammetry (DPV) for **1** and cyclic voltammetry (CV) for **2**.

b *E*_cell_ = *E*_1/2_^0/+^ – *E*_1/2_^–/0^.

c Two-electron peak.

Connecting two 6-oxoverdazyl radicals with a phenylene linker at their C(3) positions has little effect on the redox potentials of the heterocyclic units: the oxidation potential is shifted anodically by 0.01 and 0.02 V in **1-Cm** and **1-Cp**, respectively, while the reduction potential is essentially unchanged for the former and shifted anodically by 0.03 V for the latter. Consequently, the electrochemical window for the *meta* isomer is slightly expanded (+0.03 V), while for the *para***1-Cp** is contracted by 0.02 V (Table 3).

Both diradicals **1-C** exhibit two poorly resolved quasireverisble reduction peaks separated by about 0.2 V and a single oxidation peak. The separation of the current peaks in the latter process, Δ*E*_p_, of 110 mV indicates that the two one-electron oxidation processes occur at nearly the same potential. These results compare with 0.1 V separation of the reduction potentials and a single oxidation event with Δ*E*_p_ = 170 mV observed for diradicals **IVa** and **IVb** in MeCN solutions.^42^ Unfortunately, insufficient solubility of diradicals **1** in MeCN did not permit a direct comparison of the two systems.

A similar electrochemical analysis of the isomeric diradicals **1-Nm** and **1-Np** demonstrated a small anodic shift of the *E*_1/2_^–/0^ potential relative to the monoradical **2-N**, by 0.05 V, and a separation between the two one-electron reduction processes smaller than in the C-analogues, 0.13 and 0.17 V, respectively. The oxidation process in **1-Nm** is represented by a single unresolved peak, with the DPV peak shifted anodically by 0.04 V relative to the parent **2-N**. The separation of the cathodic and anodic current peaks, Δ*E*_p_, by 107 mV suggests again two one-electron processes occurring at nearly the same potential.

**Figure 12:**
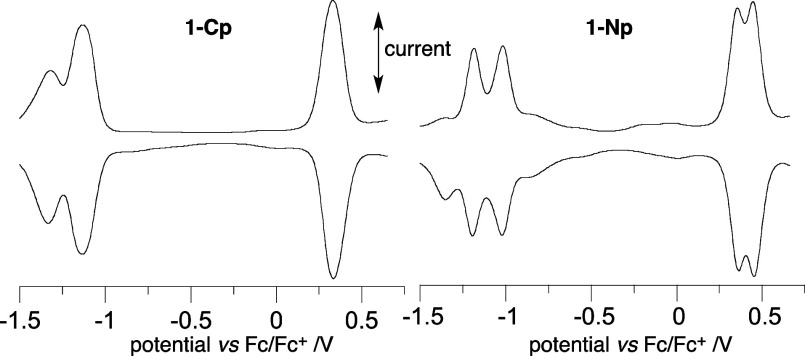
Differential pulse voltammograms (DVP) for diradicals **1-Cp** (left) and **1-Np** (right) referenced to the Fc/Fc^+^ couple (IUPAC convention): 0.5 mM in CH_2_Cl_2_ [*n*-Bu_4_N]^+^[PF_6_]^−^ (50 mM), at ca. 20 °C, 5 mV s^–1^, glassy carbon (ϕ = 2 mm) working electrode, Pt counter electrode, and Ag/AgCl pseudoreference electrode.

In contrast to the previous three diradicals, the *para* analogue, diradical **1-Np**, clearly shows two quasireversible oxidation processes spaced by 0.10 V (Figure 12), in which the *E*_1/2_^0/+^ potential differs little from that in the monoradical **2-N**. The observed two separate oxidation events indicate strong electronic interactions between the redox-active sites in **1-Np**, while their absence in other diradicals points to weak communication between the radical sites, which is consistent with results of absorption spectroscopy (*vide supra*).

### Determination of the Singlet–Triplet Energy Gap in Solid Solutions

**Figure 13:**
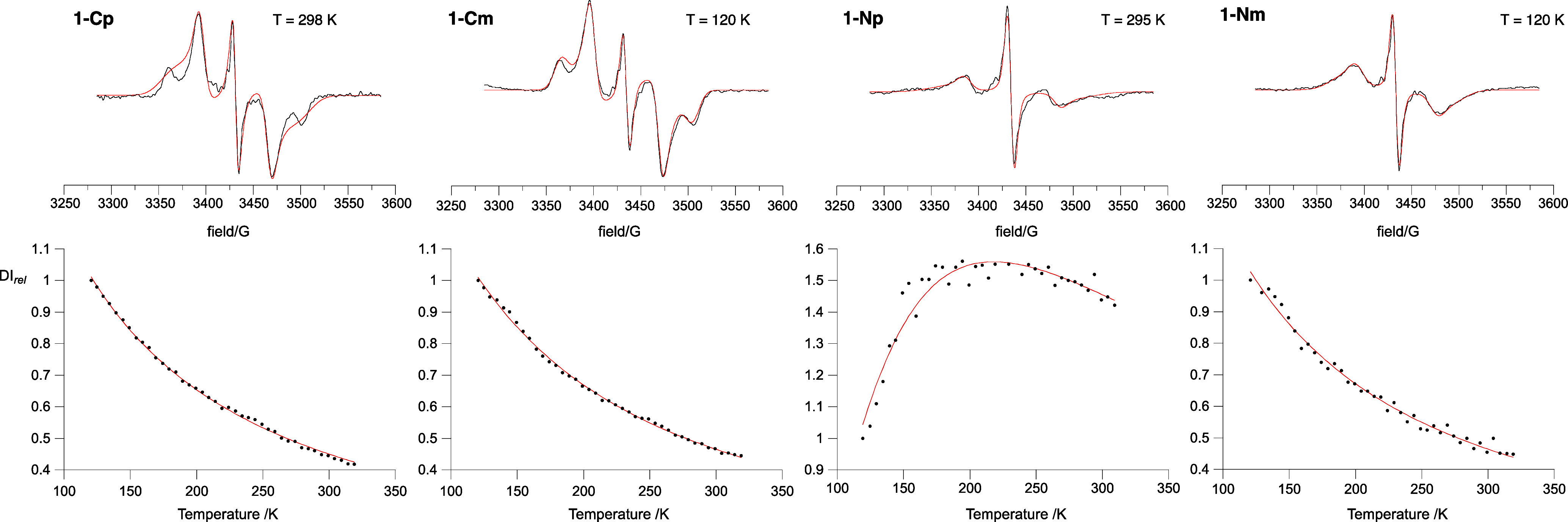
Top: Experimental (black) and simulated (red) EPR spectra of **1** in polystyrene solid solution. Bottom: Experimental DI_rel_ vs *T* data points (black dots) and Bleaney–Bowers fitting curves (red line) for diradicals **1**.

To establish the singlet–triplet energy gaps, diradicals were investigated as solid solutions in polystyrene by variable temperature EPR spectroscopy in a typical temperature range of 120–320 K (Figure 13). All diradicals **1** exhibited EPR signals characteristic for randomly oriented triplets with doublet impurities, but none of them showed the forbidden |Δ*m*_s_| = 2 transition. Therefore, analysis focused on the relative intensity of the |Δ*m*_s_| = 1 signal, reported as double integral DI_rel_, which was analyzed on the basis of Heisenberg Hamiltonian for two spins *S* = 1/2, *Ĥ* = −2*JŜ*_1_**·***Ŝ*_2_.

The intensity of the spectra increased with increasing temperature only for diradical **1-Np**, which is consistent with the thermally populated triplet state, while for the remaining three diradicals, the intensity had a decreasing character (Figure 13).^53^ Numerical fitting of the DI_rel_(*T*) data points to the modified Bleaney–Bowers model^59^ (eq 1) gave the singlet–triplet energy gaps, Δ*E*_S–T_ (as 2*J*), ranging from −0.13(1) kcal mol^–1^ for **1-Cp** to −0.69(1) kcal mol^–1^ for **1-Np** (Table 4).
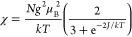
1

**Table 4:** Results of VT EPR Analysis and DFT Calculations of Diradicals **1**

radical	Δ*E*_S–T_ exp^a^/kcal mol^–1^	DFT^b^/kcal mol^–1^	Δ*H*_OSS–CS_^c^/kcal mol^–1^	|*D*/*hc*| × 10^–3^/cm^–1^	|*E*/*hc*| × 10^–4^/cm^–1^	*r*^d^/Å
**1-Cp**	–0.13(1)	–0.81	24.66	6.28	1.12	7.5
**1-Cm**	–0.16(1)	0.23	24.50	6.34	1.05	7.4
**1-Np**	–0.69(1)	–0.87	14.46	7.11	6.04	7.2
**1-Nm**	–0.14(1)	0.25	24.41	5.75	6.00	7.7

a Data from VT EPR measurements in polystyrene solid solutions.

b Adiabatic Δ*E*_S–T_ with the ZPE correction at the UB3LYP/6-311++G(2d,p)//UB3LYP/6-31G(2d,p) level in benzene obtained using eq 2.

c Adiabatic enthalpy difference between the OSS and CSS states.

d Distance between spin centers.

While antiferromagnetic (AFM) exchange interactions are expected for the *para* isomers, negative values of *J* for the *meta* isomers **1-Cm** and **1-Nm** are surprising, especially that for a close analogue of the latter *J* = 0.53(1) kcal mol^–1^ was demonstrated.^50^ Such results are, however, not unprecedented. For instance, negative mean exchange interactions were determined for two Blatter diradicals with the expected triplet ground state, which was attributed to the conformational flexibility of the molecules.^60,61^ In one case, a dependence of the *J* value on the matrix used for solid solutions was reported.^60^ DFT investigation of 6-oxoverdazyl diradicals^50,51^ closely related to **1-Nm** and other diradical systems^62−64^ revealed significant dependence of the exchange interaction energy *J* on the torsion angle between magnetically interacting units. For angles above 70°, negative *J* values were calculated.

**Figure 14:**
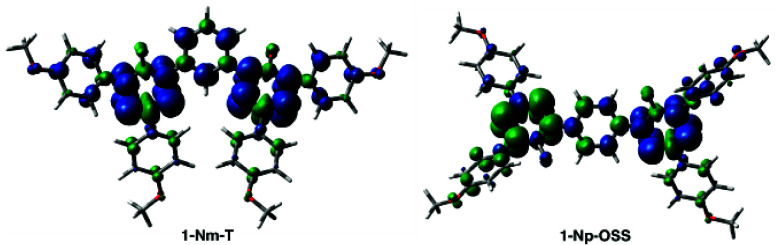
Spin density map for model **1-Nm-T** and **1-Np-OSS** (density = 0.0014).

Simulation of the experimental triplet pattern for diradicals **1** (Figure 13) gave zero-field splitting (*zfs*) parameters |*D*/*hc*| and |*E*/*hc*| (Table 4). The latter parameter is smaller for **1-C** diradicals than for the **1-N** analogues and is about 1 × 10^–4^ cm^–1^ for the former and about 6 × 10^–4^ cm^–1^ for **1-N** (Table 4). The |*D*/*hc*| parameter is about 6.3 × 10^–3^ cm^–1^ for two **1-C** diradicals, while it is significantly larger for **1-Np** (7.11 × 10^–3^ cm^–1^) and lower for **1-Nm** (5.75 × 10^–3^ cm^–1^, Table 4). The point-dipole approximation model indicates that the spins are separated by about 7.4 Å for **1-C**, while for **1-Np** they are closer by ∼0.3 Å and for **1-Nm** are farther apart by ∼0.25 Å. These values compare well to the calculated separation of about 7.5 Å. The constant separation for **1C** is consistent with the lack of spin delocalization onto the bridging benzene ring, while the markedly shorter separation observed in the **1-Np** isomer is in agreement with spin delocalization onto the central ring through a Kekulé resonance form (Figure 9). The DFT spin density maps for two **1-N** diradicals are shown in Figure 14.

For comparison purposes, the exchange interactions in all four diradicals **1** were calculated at the UB3LYP/6-311+G(2d,p)//UB3LYP/6-31G(2d,p) level of theory^65^ using the usual broken symmetry (BS) approach and the Yamaguchi formalism (eq 2).^66,67^
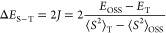
2where *E* is the SFC energy corrected for ZPE and ⟨S^2^⟩ is the total spin angular momentum of high (T) or low (OSS) spin state. DFT results demonstrated moderate Δ*E*_S–T_ values of about −0.8 kcal mol^–1^ for the *para* isomers and 0.25 kcal mol^–1^ for the *meta* isomers (Table 4). The calculated values are consistent with those calculated for similar diradicals,^51,68,69^ and the latter compares to the value of Δ*E*_S–T_ = 0.28 kcal mol^–1^ for **IVi**.^43^ Spin-restricted calculations revealed that the closed-shell singlet state (CS) is above the OSS state by Δ*H*_OSS–CS_ = 14.46 kcal mol^–1^ for **1-Np**, which is the only diradical in series **1** with a CS Kekulé structure (Figure 9). For the remaining diradicals, no closed-shell Kekulé structures are available, and the calculated enthalpy difference Δ*H*_OSS–CS_ is significantly higher (∼24.5 kcal mol^–1^). Associated with the availability of the CS Kekulé resonance form for **1-Np** is also the calculated diradicaloid index^70,71^*y* = 0.88, which compares to *y* = 0.99 for **1-Cp**.

A comparison of experimental and calculated S–T energy gaps demonstrates a reasonable close match for **1-Np**. The significantly negative experimental Δ*E*_S–T_ values for the *meta* isomers may result from a relatively weak preference for the triplet state (0.25 kcal mol^–1^) overridden by antiferromagnetic exchange interactions in easily accessible conformational states^50,51^ and possible molecular aggregations in the solid solutions.

### Solid-State Magnetic Properties

**Figure 15:**
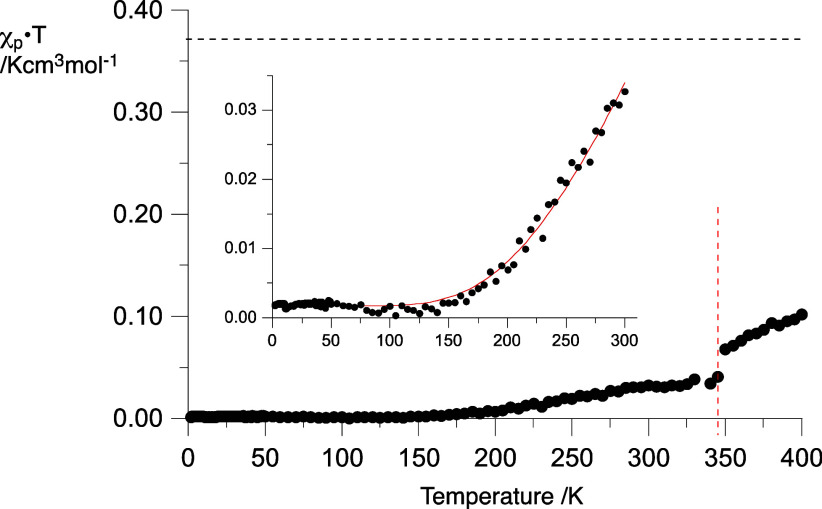
χ_p_*T* (per spin) vs temperature plot for **1-Np**. The horizontal dotted line marks χ_p_*T* = 0.375 cm^3^ mol^–1^ K for an ideal paramagnet. The red vertical line marks an abrupt change. The inset shows an expanded portion of the plot with the fit (red line) to the Bleaney–Bowers model (eq 1) and the 2*J*/*k*_B_ = −1018(18) K (*r*^2^ = 0.990).

Magnetic susceptibility of four diradicals and two monoradicals was measured in the polycrystalline solid using a SQUID magnetometer. Results indicate antiferromagnetic interactions for all six compounds,^53^ while only two, **1-Np** and **2-C**, can be properly analyzed using their crystal structures (*vide supra*). Diradical **1-Np** exhibits the strongest antiferromagnetic (AFM) interactions in the series with essentially no paramagnetic signal below 150 K (Figure 15). Thermal population of higher spin states leads to slow increase of the χ_p_*T* values, which reaches 8% of free spins at 300 K. At about 325 K, the χ_p_*T*(*T*) curve changes its character with a small discontinuity observed in both cooling and heating modes, which may indicate a structural transition (such as a Cr–Cr transition) in the solid.

Assuming that the intramolecular AFM exchange interactions are significantly stronger and dominant relative to those of intermolecular nature (|*J*_intra_| ≫ |*J*_inter_|), the observed AFM behavior can be ascribed solely to individual diradical molecules and hence can be analyzed with the Bleaney–Bowers model^59^ (eq 1). Thus, fitting the χ_p_*T*(*T*) data for **1-Np** in a range of 2–300 K to eq 1 gave the *J/k*_B_ = −509(9) K or Δ*E*_S–T_ = −2.02(4) kcal mol^–1^. UB3LYP/6-311++G(2d,p) level of theory single-point calculations of the S–T energy gap in a molecule of **1-Np** at its crystallographic coordinates gave a comparable value of Δ*E*_S–T_ (DFT) = −2.74 kcal mol^–1^. Calculations for the nearest pair of molecules of **1-Np** shows nearly the same energy gap (Δ*E*_S–T_ = −2.77 kcal mol^–1^), demonstrating validity of the assumption |*J*_intra_| ≫ |*J*_inter_|. This value for intramolecular exchange interaction is significantly greater than the −0.87 kcal mol^–1^ calculated for **1-Np** in equilibrium geometry in the gas phase and −0.69 kcal mol^–1^ obtained from the solid-solution studies. This large difference is presumably due to a markedly lower torsion angle and hence better π–π overlap between the central benzene ring and the 6-oxoverdazyls in the solid state structure (exp. 5.4° and 8.4° vs DFT 31.7°, Table 1). It is also expected that the experimental high 6-oxoverdazyl–phenyl torsion angles at the N(5) and C(3) positions lead to higher spin concentration on the 6-oxoverdazyl unit and hence stronger AFM exchange interactions mediated by the 1,4-phenylene than those found in gas-phase optimized molecules.

**Figure 16:**
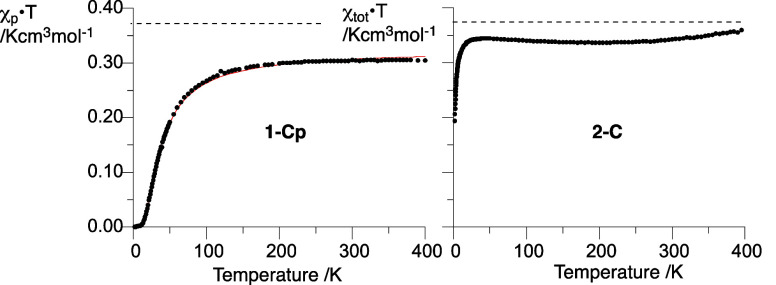
Left: χ_p_*T* (per spin) vs temperature plot for **1-Cp** with the fit (red line) to the Bleaney–Bowers model (eq 1) and 2*J*/*k*_B_ = −66.9(2) K (*r*^2^ = 0.9995). Right: χ_tot_*T* vs temperature plot for **2-C**. The horizontal dotted line marks χ_p_*T* = 0.375 cm^3^ mol^–1^ K for an ideal paramagnet.

In contrast to **1-Np**, the onset of paramagnetic behavior in diradical **1-Cp** takes place at a much lower temperature, 13 K, and reaches the level of 80% of free spins at 200 K (Figure 16; for details, see the SI). A similar analysis of the entire curve with the Bleaney–Bowers model^59^ (eq 1) yields the exchange interaction *J*/*k*_B_ = −33.5(1) K or Δ*E*_S–T_ = −132.9(4) cal mol^–1^. The remaining two diradicals, **1-Nm** and **1-Cm**, show qualitatively similar χ_tot_*T*(*T*) curves, although they cannot be analyzed, considering the expected triplet GS and the lack of solid-state structures.

Analysis of the monoradical **2-C** revealed a high concentration of free spins at 2 K (51%), which increases to over 90% at 45 K, indicating very weak antiferromagnetic interactions (Figure 16). DFT calculations for a pair of neighboring molecules in the chain (Figure 6) at their crystallographic coordinates demonstrates essentially no exchange interactions (Δ*E*_S–T_ = −0.1 cal mol^–1^) in agreement with the experiment. Radical **2-N** also saturates quickly to 93% of spins at 50 K,^53^ although its detailed analysis was hampered by the absence of the solid-state structure.

## Conclusions

The development of synthetic access to 6-oxoverdazyl diradicals connected with a phenylene linker at the N(1) position for the first time opened up access to a complete series of closely related isomeric diradicals and enabled their extensive comparison as to the effect of the connectivity and linker’s topology on diradical properties. Results indicate that the newly available *para*-phenylene derivative, **1-Np**, is the only diradical in the series with the available closed-shell Kekulé resonance structure. For this reason, structural, spectroscopic, and electrochemical properties of **1-Np** are markedly distinct in the series.

DFT calculations predict small preference for the triplet state of 0.25 kcal mol^–1^ in both *meta* diradicals and moderate open-shell singlet ground state stability (Δ*E*_S–T_ ∼ – 0.8 kcal mol^–1^) for the *para* isomers, regardless of the connectivity. This suggests a similar effectiveness of the combination of spin density and the π–π overlap at the C(3) and N(1) connecting sites: lower spin density and higher coplanarity at C(3) vs higher spin density and lower coplanarity at the N(1). VT EPR spectroscopy analysis yielded mean negative exchange interaction energy, *J*, in all four diradicals with either a *meta*-phenylene or *para*-phenylene connector. While negative values are expected for the latter diradicals, results suggest the dominance of conformational effects in intramolecular spin–spin exchange interactions compounded with possible aggregation in the solid solutions of the *meta*-phenylene-connected diradicals.

The availability of a synthetic route to the N(1)-phenylene-connected 6-oxoverdazyls opens up access to diradicals with finely tuned properties and also to diradicals with more complex molecular architectures. One such diradical, a functionalized derivative of **1-Nm**, is reported elsewhere.^50^

## Experimental Section

### Computational Details

Quantum-mechanical calculations were carried out using the Gaussian 09 suite of programs.^72^ Geometry optimizations were conducted at the UB3LYP/6-31G(2d,p) level of theory in vacuum without symmetry constraints for the triplet state, which then served as the starting point for geometry optimization of the corresponding open-shell singlet for each diradical. Calculations involving the open-shell singlet (OSS) used the “guess(mix, always)” keyword. Several conformers of model **1′** were analyzed, and the most stable geometries were used for calculations of the OSS states. All equilibrium molecular geometries are shown in the Supporting Information.

Frequency calculations were performed to verify the nature of the stationary points and to obtain ZPE corrections for each diradical. Electronic excitation energies of open-shell diradicals **1′** in CH_2_Cl_2_ dielectric medium were obtained at the UB3LYP/6-31G(2d,p)//UB3LYP/6-31G(2d,p) level of theory using the time-dependent TD-DFT method.^73^ The solvation model was implemented with the PCM model^74^ using the SCRF(solvent = CH2Cl2) keyword.

Adiabatic singlet–triplet energy gaps, Δ*E*_S–T_, for series **1** were calculated as a difference of *E*_BS_ and *E*_T_ energies obtained at the UB3LYP/6-311++G(2d,p)//UB3LYP/6-31G(2d,p) level of theory in benzene and corrected for ZPE using the Yamaguchi formalism shown in eq 2.^66,67,75^

Diradical character *y* was calculated by a symmetry-broken UHF/6-311G(d,p)//UB3LYP/6-31G(2d,p) method (pop = no) using the occupancy of natural orbital (*y* = 0 for the closed-shell and *y* = 1 for the pure singlet diradical).^75^ Other computational details are provided in the SI.

### General Methods

Solvents and reagents were purchased and used as received without further purification. Heat in reactions involving elevated temperatures was supplied using oil baths, and reported temperature refers to that of the bath. Products were purified by flash chromatography on silica gel (230–400 mesh). NMR spectra were recorded with Bruker AVIII 600 instrument. Chemical shifts are reported in CDCl_3_ or DMSO-*d*_6_ relative to solvent residual peaks (^1^H NMR, δ = 2.50 ppm for DMSO-*d*_6_ and δ = 7.26 ppm for CDCl_3_; ^13^C NMR, δ = 39.52 ppm for DMSO-*d*_6_ and δ = 77.16 for CDCl_3_).^76^ The ESI-MS spectra were obtained using a Varian 500 MS LS Ion Trap spectrometer. IR spectra were recorded in KBr using a NEXUS FT-IR spectrophotometer. UV–vis spectra were recorded in spectroscopic-grade CH_2_Cl_2_ in concentration range of 2–20 × 10^–5^ M. Molar extinction coefficients ε were obtained by fitting the maximum absorbance against concentration in agreement with Beer’s law. More details are provided in the SI. Deactivated SiO_2_ was prepared by treatment with MeOH containing 10% (v/v) of H_2_O and subsequent drying at 90 °C for 1 h.

### Preparation of Diradicals **1**. A General Procedure.

A solution of bis-tetrazane **6** or **12** (205 mg, 0.25 mmol), NaIO_4_ (320 mg, 1.5 mmol), and [Bu_4_N]^+^Br^–^ (cat.) in a mixture of CH_2_Cl_2_ and MeOH (10 mL, 1:1 *v*/*v*) were intensively stirred at rt for 12 h. The solid was filtrated and solvents removed under reduced pressure. Crude products were purified by fast filtration trough a deactivated SiO_2_ pad (pet. ether/CH_2_Cl_2_, gradient 0–100%). Analytically pure diradicals **1** were obtained by recrystallization from CH_2_Cl_2_/MeCN and then from CH_2_Cl_2_/hexane.

#### 1,4-Bis[1,5-di(4-isopropoxyphenyl)-6-oxo-3-verdazyl)benzene radical (**1-Cp**)

Obtained in 54% yield (109 mg, 0.135 mmol) from bis-tetrazane **6p** as a dark brown solid: mp 315–316 °C (CH_2_Cl_2_/MeCN); UV–vis (CH_2_Cl_2_), λ_max_ (log ε) 280 (4.59), 339 (4.34), 469 (3.75), 571 (3.82); IR (KBr) ν 2974, 1695 (C=O), 1502, 1245 cm^–1^; MS (ESI-TOF) *m*/*z* 809 (85, [M + H]^+^), 831 (100, [M + Na]^+^); HRMS (ESI-TOF) *m*/*z* [M + Na]^+^ calcd for C_46_H_48_N_8_O_6_Na: 831.3595, found 831.3594. Anal. Calcd for C_46_H_48_N_8_O_6_: C, 68.30; H, 5.98; N, 13.85. Found: C, 68.06; H, 5.95; N, 13.96.

#### 1,3-Bis[1,5-di(4-isopropoxyphenyl)-6-oxo-3-verdazyl)benzene radical (**1-Cm**)

Obtained in 60% yield (121 mg, 0.150 mmol) from bis-tetrazane **6m** as a dark pink-brown solid: mp 98–100 °C (CH_2_Cl_2_/hexane); UV–vis (CH_2_Cl_2_), λ_max_ (log ε) 267 sh (4.57), 339 (4.32), 459 (3.67), 571 (3.85); IR (KBr) ν 2974, 1695 (C=O), 1499, 1239 cm^–1^; MS (ESI-TOF) *m*/*z* 808 (100, [M]^+^), 831 (85, [M + Na]^+^); HRMS (ESI-TOF) *m*/*z* [M + Na]^+^ calcd for C_46_H_48_N_8_O_6_Na: 831.3595, found 831.3602. Anal. Calcd for C_46_H_48_N_8_O_6_: C, 68.30; H, 5.98; N, 13.85. Found: C, 68.27; H, 5.93; N, 14.05.

#### 1,4-Bis[3,5-di(4-isopropoxyphenyl)-6-oxo-1-verdazyl)benzene radical (**1-Np**)

Obtained in 31% yield (63 mg, 0.078 mmol) from bis-tetrazane **12p** as a blue-black solid: mp 208–209 °C (CH_2_Cl_2_/hexane); UV–vis (CH_2_Cl_2_), λ_max_ (log ε) 274 (4.91), 341 (4.38), 484 (3.96), 601 (4.16); MS (ESI-TOF) *m*/*z* 809 (50, [M + H]^+^), 831 (50, [M + Na]^+^); HRMS (ESI-TOF) *m*/*z* [M]^+^ calcd for C_46_H_48_N_8_O_6_: 808.3697, found 808.3705. Anal. Calcd for C_46_H_48_N_8_O_6_: C, 68.30; H, 5.98; N, 13.85. Found: C, 68.17; H, 5.96; N, 13.92.

#### 1,3-Bis[3,5-di(4-isopropoxyphenyl)-6-oxo-1-verdazyl)benzene radical (**1-Nm**)

Obtained in 39% yield (80 mg. 0.099 mmol) from bis-tetrazane **12m** as a black-green solid: mp 201–203 °C (CH_2_Cl_2_/hexane); UV–vis (CH_2_Cl_2_), λ_max_ (log ε) 274 (4.36), 587 (3.10); IR (KBr) ν 2977, 1695 (C=O), 1606, 1505, 1249 cm^–1^; MS (ESI-TOF) *m*/*z* 809 (70, [M + H]^+^), 831 (100, [M + Na]^+^); HRMS (ESI-TOF) *m*/*z* [M + Na]^+^ calcd for C_46_H_48_N_8_O_6_Na: 831.3595, found 831.3588. Anal. Calcd for C_46_H_48_N_8_O_6_: C, 68.30; H, 5.98; N, 13.85. Found: C, 68.33; H, 5.97; N, 13.67.

### Preparation of Other Compounds

#### 1,5-Di(4-isopropoxyphenyl)-3-phenyl-6-oxoverdazyl radical (**2-C**)

A solution of tetrazane **17** (222 mg, 0.50 mmol), NaIO_4_ (320 mg, 1.5 mmol), and [Bu_4_N]^+^Br^–^ (3 mg, 0.009 mmol) in a mixture of CH_2_Cl_2_ and MeOH (10 mL, 1:1 *v*/*v*) was intensively stirred for 6 h. Then, the solid was filtrated off and the solvent removed under reduced pressure. The residue was purified by column chromatography (deactivated SiO_2_, pet. ether/CH_2_Cl_2_, 50–100%), giving 140 mg (0.316 mmol, 63% yield) of radical **2-C** as a dark-purple solid. Analytically pure product was obtained by recrystallization from EtOH and then from CH_2_Cl_2_/hexane: mp 136–138 °C (CH_2_Cl_2_/hexane); UV–vis (CH_2_Cl_2_), λ_max_ (log ε) 337 (4.02), 450 (3.34), 571 (3.58); MS (ESI-TOF) *m*/*z* 466 (100, [M + Na]^+^), 444 (70, [M + H]^+^); HRMS (ESI-TOF) *m*/*z* [M + Na]^+^ calcd for C_26_H_27_N_4_O_3_Na: 466.1981, found 466.1987. Anal. Calcd for C_26_H_27_N_4_O_3_: C, 70.41; H, 6.14; N, 12.63. Found: C, 70.45; H, 6.22; N, 12.62.

#### 1,3-Di(4-isopropoxyphenyl)-5-phenyl-6-oxoverdazyl radical (**2-N**)

Following the procedure for preparation of **2-C**, radical **2-N** was obtained in 66% yield (146 mg, 0.330 mmol) from 223 mg (0.50 mmol) of tetrazane **20** as a black-green solid: mp 106–107 °C (hexane); UV–vis (CH_2_Cl_2_), λ_max_ (log ε) 273 (4.42), 327 sh (3.96), 402 (3.27), 583 (3.39); IR (KBr) ν 2978, 1694 (C=O), 1505, 1247 cm^–1^; MS (ESI-TOF) *m*/*z* 466 (100, [M + Na]^+^), 444 (60, [M + H]^+^). Anal. Calcd for C_26_H_27_N_4_O_3_: C, 70.41; H, 6.14; N, 12.63. Found: C, 70.39; H, 6.16; N, 12.72.

#### *N,N*′-Bis(4-*i*-propoxyphenyl)carbohydrazide (**5**)

Carbohydrazide (**3**, 0.859 g, 9.54 mmol), 1-iodo-4-(*i*-propoxy)benzene (**4**, 5.0 g, 19.1 mmol), 1,10-phenanthroline (0.344 g, 1.91 mmol), CuI (0.272 g, 1.43 mmol), and K_3_PO_4_ (4.86 g, 22.9 mmol) were dissolved in degassed, anhydrous DMF (35 mL). The resulting mixture was degassed again and then heated at 90 °C for 3 h. The solvent was removed under reduced pressure. The residue was suspended in water (50 mL) and extracted with AcOEt (8 × 100 mL). Combined organic layers were dried (Na_2_SO_4_) and solvent was evaporated. The crude product was purified on automatized flash chromatography (SiO_2_, pet. ether/AcOEt, gradient 0–100%), giving 1.85 g (5.17 mmol, 55% yield) of carbohydrazide **5** as a pale-yellow solid: mp 84–87 °C (pet. ether/AcOEt)*;*^1^H NMR (CDCl_3_, 600 MHz) δ 6.89 (dt, *J*_1_*=* 8.9 Hz, *J*_2_*=* 3.4 Hz, 4H), 6.65 (dt, *J*_1_*=* 8.9 Hz, *J*_2_*=* 3.4 Hz, 4H), 4.47 (bs, 4H), 4.41 (sept, *J* = 6.1 Hz, 2H), 1.27 (d, *J* = 6.1 Hz, 12H); ^13^C{^1^H} NMR (CDCl_3_, 151 MHz) δ 162.9, 155.9, 138.0, 126.4, 116.4, 70.4, 22.1; IR (KBr) ν 3348 (N–H), 2968, 1632 (C=O), 1506, 1240 cm^–1^. Anal. Calcd for C_19_H_26_N_4_O_3_: C, 63.67; H, 7.31; N, 15.63. Found: C, 63.70; H, 7.31; N, 15.58.

#### 3,3′-(1,4-Phenylene)bis[1,5-di(isopropoxyphenyl)-1,2,4,5-tetrazinan-6-one] (**6p**)

A mixture of carbohydrazide **5** (460 mg, 1.28 mmol) and terephthaldehyde (80.5 mg, 0.60 mmol) in EtOH (10 mL) was reflux for 8 h under Ar atmosphere. The reaction mixture was cooled to rt and then kept overnight in the freezer. The resulting precipitate was filtered off, washed with cold EtOH (2 × 10 mL) and Et_2_O (2 × 10 mL), and dried under vacuum, giving 380 mg (0.47 mmol, 78% yield) of bis(terazinan-6-one) **6p** as a colorless amorphous solid: mp 246–247 °C (EtOH); ^1^H NMR (DMSO-*d*_6_, 600 MHz) δ 7.53 (s, 4H), 7.40 (d, *J* = 8.9 Hz, 8H), 6.85 (d, *J* = 8.9 Hz, 8H), 6.27 (d, *J* = 8.9 Hz, 4H), 5.32 (t, *J* = 8.9 Hz, 2H), 4.56 (sept, *J* = 5.9 Hz, 4H), 1.26 (d, *J* = 5.9 Hz, 24 H); ^13^C{^1^H} NMR (DMSO-*d*_6_, 151 MHz) δ 153.9, 137.0, 136.1, 126.9, 124.2, 124.0, 115.1, 71.3, 69.3, 21.8; IR (KBr) ν 3227 (N–H), 3227, 2974, 1625 (C=O), 1505, 1242 cm^–1^; MS (ESI-TOF) *m*/*z* 837 (100, [M + Na]^+^). Anal. Calcd for C_46_H_54_N_8_O_6_: C, 67.79; H, 6.68; N, 13.75. Found: C, 67.66; H, 6.68; N, 13.61.

#### 3,3′-(1,3-Phenylene)bis[1,5-di(isopropoxyphenyl)-1,2,4,5-tetrazinan-6-one] (**6m**)

Following the procedure for preparation of **6p**, bis(tetrazinan-6-one) **6m** was obtained in 82% (0.53 mmol, 430 mg) from 460 mg (1.28 mmol) of carbohydrazide **5** and isophthalaldehyde as a colorless amorphous solid: mp 215–217 °C (EtOH); ^1^H NMR (DMSO-*d*_6_, 600 MHz) δ 7.85 (s, 1H), 7.53 (d, *J* = 7.7 Hz, 2H), 7.42 (d, *J* = 8.9 Hz, 8H), 7.36 (t, *J* = 7.7 Hz, 1H), 6.84 (d, *J* = 9.0 Hz, 8H), 6.24 (d, *J* = 9.0 Hz, 4H), 5.31 (t, *J* = 9.0 Hz, 2H), 4.54 (sept, *J* = 6.0 Hz, 4H), 1.26 (d, *J* = 6.1 Hz, 24H); ^13^C{^1^H} NMR (DMSO-*d*_6_, 151 MHz) δ 153.9, 137.0, 136.1, 128.2, 126.6, 126.1, 124.2, 115.0, 71.3, 69.3, 21.8; IR (KBr) ν 3246 (N–H), 2977, 1628 (C=O), 1499, 1242 cm^–1^; MS (ESI-TOF) *m*/*z* 837 (100, [M + Na]^+^). Anal. Calcd for C_46_H_54_N_8_O_6_: C, 67.79; H, 6.68; N, 13.75. Found: C, 67.67; H, 6.77; N, 13.59.

#### *N,N*′-Bis(4-*i*-propoxyphenyl)tetrahydrazide (**7p**).

A mixture of bishydrazide **10p** (227 mg, 0.90 mmol), 1-iodo-4-(*i*-propoxy)benzene (**4**, 420 mg, 1.60 mmol), CuI (14 mg, 0.075 mmol), and K_3_PO_4_ (478 mg, 2.25 mmol) in degassed, anhydrous DMF (1 mL) was heated at 50 °C for 24 h. Solvent was removed under reduced pressure, and the crude product was purified by column chromatography (AcOEt and then AcOEt/MeOH, 9:1), giving 242 mg of bishydrazide **7p** as a light-brown solid. Recrystallization from CH_2_Cl_2_/EtOH gave 236 mg (0.45 mmol, 50% yield based on **10p**) of analytically pure product as a white solid: mp 181–183 °C (CH_2_Cl_2_/EtOH); ^1^H NMR (DMSO-*d*_6_, 600 MHz) δ 7.15 (d, *J* = 8.9 Hz, 4H), 7.08 (s, 4H), 6.77 (d, *J* = 8.9 Hz, 4H), 5.03 (s, 8H), 4.50 (sept, *J* = 6.0 Hz, 2H), 1.22 (d, *J* = 6.0 Hz, 12H); ^13^C{^1^H} NMR (DMSO-*d*_6_, 151 MHz) δ 161.0, 153.7, 141.2, 138.7, 123.9, 121.3, 115.3, 69.3, 21.8; IR (KBr) ν 3288 (N–H), 3193 (N–H), 2974, 1647 (C=O), 1511, 1241 cm^–1^; MS (ESI-TOF) *m*/*z* 545 (100, [M + Na]^+^). Anal. Calcd for C_26_H_34_N_8_O_4_: C, 59.75; H, 6.56; N, 21.44. Found: C, 59.78; H, 6.59; N, 21.50.

#### Dibenzyl 1,1′-(1,4-phenylene)bis(hydrazinecarboxylate) (**9p**)

1,4-Diiodobenzene (9.87 g, 30.0 mmol), benzyl hydrazinecarboxylate (**8**, 13.96 g, 84 mmol), 1,10-phenanthroline (1.62 g, 9.0 mmol), and CuI (0.857 g, 4.50 mmol) were dissolved in degassed, anhydrous DMF (100 mL). The mixture was degassed again and heated at 90 °C for 4 h. During heating, Cs_2_CO_3_ (21.5 g, 66 mmol) was added in equal portions every 15 min. Then, solvent was removed under reduced pressure, and the residue was suspended in water (100 mL) and extracted with mixture of CH_2_Cl_2_/MeOH (5:1; 8 × 150 mL). Combined organic layers were dried (Na_2_SO_4_) and solvents were evaporated. The crude product was purified on automatized flash chromatography system (SiO_2_-cartridge, pet. ether/AcOEt, gradient 0–70%), giving 9.38 g (23.1 mmol, 78% yield) of bis-carbazide **9p** as a pale-yellow solid: mp 96–99 °C (pet. ether/AcOEt); ^1^H NMR (CDCl_3_, 600 MHz) δ 7.44 (s, 4H), 7.32–7.37 (m, 10H), 5.23 (s, 4H), 4.50 (s, 4H); ^13^C{^1^H} NMR (CDCl_3_, 151 MHz) δ 156.1, 136.1, 128.7, 128.5, 128.45, 128.35, 128.3, 68.3; IR (KBr) ν 3354 (N–H), 3031, 2965, 1689, 1657 (C=O), 1435, 1356, 1188 cm^–1^; MS (ESI-TOF) *m*/*z* 429 (100, [M + Na]^+^); HRMS (ESI-TOF) *m*/*z* [M + Na]^+^ calcd for C_22_H_22_N_4_O_4_Na: 429.1539, found 429.1543.

#### Dibenzyl 1,1′-(1,3-phenylene)bis(hydrazinecarboxylate) (**9m**)

Following the procedure for preparation of **9p**, biscarbazide **9m** was obtained in 71% yield (8.66 g, 21.3 mmol) from 9.90 g (30.0) of 1,3-diiodobenzene and 13.96 g (84.0 mmol) of benzyl hydrazinecarboxylate (**8**) as a yellow oil: ^1^H NMR (CDCl_3_, 500 MHz) δ 7.70 (bs, 1H), 7.22–7.36 (m, 13H), 5.22 (s, 4H), 4.45 (bs, 4H); ^13^C{^1^H} NMR (CDCl_3_, 125 MHz) δ 155.9, 142.8, 136.0, 128.6, 128.4, 128.22, 128.20, 120.4, 118.8, 68.3; CI-MS *m*/*z* 407 (8, [M + H]^+^), 91 (100). Anal. Calcd for C_22_H_22_N_4_O_4_: C, 65.01; H, 5.46; N, 13.78. Found: C, 65.09; H, 5.58; N, 13.59.

#### *N,N*′-(1,4-Phenylene)bis(hydrazinecarboxamide) (**10p**)

The mixture of bis-carbazide **9p** (4.06 g, 10.0 mmol) and hydrazine monohydrate (20 mL) was heated at 100 °C for 1 h. The reaction mixture was cooled below 0 °C, and the resulting precipitated solid was filtered. Then, the solid was washed with cold H_2_O (3 × 10 mL), cold MeOH (1 × 10 mL), and Et_2_O (5 × 10 mL) and finally dried under reduced pressure, giving 0.98 g (3.86 mmol, 38% yield) of bishydrazide **10p** as a white solid: mp 334 °C (dec.); ^1^H NMR (DMSO-*d*_6_ + CF_3_COOD, 600 MHz) δ 9.68 (bs, 4H), 7.56 (s, 4H), 5.48 (bs, 2H), 3.53 (bs, 4H); ^13^C{^1^H} NMR (DMSO-*d*_6_ + CF_3_COOD, 151 MHz) δ 156.7, 140.0, 121.9; IR (KBr) ν 3335 (N–H), 3316 (N–H), 3205 (N–H), 1654, 1622 (C=O), 1505 cm^–1^. Anal. Calcd for C_8_H_14_N_8_O_2_: C, 37.79; H, 5.55; N, 44.07. Found: C, 37.79; H, 5.58; N, 44.02.

#### 1,1′-(1,4-Phenylene)bis[3,5-di(*i*-propoxyphenyl)]-1,2,4,5-tetrazinan-6-one (**12p**)

A solution of *N*,*N*′-bis(4-*i*-propoxyphenyl)tetrahydrazide (**7b**, 313 mg, 0.60 mmol) and 4-(*i*-propoxy)benzaldehyde (**11**, 210 mg, 1.28 mmol) in EtOH (10 mL) was refluxed for 8 h under Ar atmosphere. The reaction mixture was cooled to rt and then kept overnight in the freezer. The resulting precipitate was filtered, washed with cold EtOH (2 × 10 mL) and Et_2_O (2 × 10 mL), and then dried under vacuo, giving 270 mg (0.332 mmol, 55% yield; 48–56% in several runs) of bis(tetrazinan-6-one) **12p** as a colorless amorphous solid: mp 206–208 °C (EtOH); ^1^H NMR (DMSO-*d*_6_, 600 MHz) δ 7.50 (s, 4H), 7.44 (t, *J* = 8.9 Hz, 8H), 6.90 (d, *J* = 8.6 Hz, 4H), 6.87 (d, *J* = 9.0 Hz, 4H), 6.23 (d, *J* = 9.2 Hz, 2H), 6.20 (d, *J* = 9.5 Hz, 2H), 5.27 (t, *J* = 9.4 Hz, 2H), 4.60 (sept, *J* = 6.1 Hz, 2H), 4.56 (sept, *J* = 6.0 Hz, 2H), 1.26 (d, *J* = 6.0 Hz, 12H), 1.25 (d, *J* = 6.1 Hz, 12H); ^13^C{^1^H} NMR (DMSO-*d*_6_, 151 MHz) δ 157.3, 154.7, 153.9, 138.7, 136.0, 128.9, 128.2, 124.0, 121.5, 115.3, 115.1, 71.5, 69.4, 69.1, 21.84, 21.75; IR (KBr) ν 3240 (N–H), 2974, 1625, 1609 (C=O), 1499, 1245 cm^–1^; MS (ESI-TOF) *m*/*z* 837 (100, [M + Na]^+^), 815 (20, [M + H]^+^); HRMS (ESI-TOF) *m*/*z* [M + Na]^+^ calcd for C_46_H_54_N_8_O_6_Na: 837.4064, found 837.4073. Anal. Calcd for C_46_H_54_N_8_O_6_: C, 67.79; H, 6.68; N, 13.75. Found: C, 67.54; H, 6.58; N, 13.73.

#### 1,1′-(1,3-Phenylene)bis[3,5-di(*i*-propoxyphenyl)-1,2,4,5-tetrazinan-6-one (**12m**)

A mixture of crude bis-carbamoyl chloride **15 (**277 mg, 0.50 mmol), 4-*i*-propoxyphenylhydrazine hydrochloride (**16·HCl**, 223 mg, 1.10 mmol) and Et_3_N (303 mg, 3.00 mmol) in EtOH (5 mL) was heated at 75 °C for 12 h under Ar atmosphere. The reaction mixture was cooled to rt and then kept overnight in the freezer. The resulting precipitate was filtered, washed with cold EtOH (2 × 10 mL) and dried under vacuum, giving 105 mg (0.129 mmol, 26% yield) of bis(tetrazinan-6-one) **12m** as colorless amorphous solid: mp 190–192 °C (EtOH); ^1^H NMR (DMSO-*d*_6_, 600 MHz) δ 7.89 (s, 1H), 7.43 (t, *J* = 7.4 Hz, 8H), 7.31 (d, *J* = 8.0 Hz, 2H), 7.22 (t, *J* = 8.3 Hz, 1H), 6.87 (t, *J* = 7.7 Hz, 8H), 6.25 (d, *J* = 9.2 Hz, 2H), 6.21 (d, *J* = 9.4 Hz, 2H), 5.25 (t, *J* = 9.2 Hz, 2H), 4.57 (sept, *J* = 6.1 Hz, 4H), 1.26 (d, *J* = 5.9 Hz, 12H), 1.24 (d, *J* = 5.9 Hz, 12H); ^13^C{^1^H} NMR (DMSO-*d*_6_, 151 MHz) δ 157.3, 155.2, 153.9, 142.9, 136.0, 129.0, 128.2, 127.1, 124.0, 117.1, 115.4, 115.3, 115.1, 71.8, 69.4, 69.1, 21.9, 21.8; IR (KBr) ν 3231 (N–H), 2976, 1625 (C=O), 1508, 1244 cm^–1^; MS (ESI-TOF) *m*/*z* 815 (100, [M + H]^+^); HRMS (ESI-TOF) *m*/*z* [M + H]^+^ calcd for C_46_H_55_N_8_O_6_: 815.4245, found 815.4254. Anal. Calcd for C_46_H_54_N_8_O_6_: C, 67.79; H, 6.68; N, 13.75. Found: C, 67.41; H, 6.59; N, 13.82.

#### 1,3-Bis[2-(4-*i*-propoxybenzylidene)hydrazinyl]benzene (**14**)

Crude 1,3-phenylenedihydrazine dihydrochloride^11^ (**13·HCl**, 44% purity, 960 mg, 2.0 mmol) and 4-isopropoxybenzaldehyde (**11**, 656 mg, 4.0 mmol) were suspended in H_2_O (10 mL). Then, a portion of AcONa (656 mg, 8.0 mmol) was added, and the reaction was vigorously stirred at rt for 30 min. The reaction mixture was extracted with CH_2_Cl_2_ (4 × 15 mL), combined organic layers were dried (Na_2_SO_4_), and solvent evaporated. The residual solid was washed with hexane and dried under vacuum, giving 600 mg (1.395 mmol, 70% yield) of bis-hydrazone **14** as a light orange solid: mp 212–215 °C (CH_2_Cl_2_); ^1^H NMR (DMSO-*d*_6_, 600 MHz) δ 10.04 (s, 2H), 7.79 (s, 2H), 7.54 (d, *J* = 8.6 Hz, 4H), 7.01 (t, *J* = 8.0 Hz, 1H), 6.94 (d, *J* = 8.6 Hz, 4H), 6.80 (s, 1H), 6.44 (dd, *J*_1_ = 8.0 Hz, *J*_2_ = 1.6 Hz, 2H), 4.64 (sept, *J* = 6.1 Hz, 2H), 1.28 (d, *J* = 6.0 Hz, 12H); ^13^C{^1^H} NMR (DMSO-*d*_6_, 151 MHz) δ 157.4, 146.4, 136.1, 129.6, 128.4, 126.9, 115.8, 102.9, 95.5, 69.2, 21.8; IR (KBr) ν 3323, 3285 (N–H), 2976, 1605, 1488, 1245, 1195 cm^–1^. Anal. Calcd for C_26_H_30_N_4_O_2_: C, 72.53; H, 7.02; N, 13.01. Found: C, 72.53; H, 6.96; N, 12.99.

#### 1,1′-(1,3-Phenylene)bis(2-(4-*i*-propoxybenzylidene)hydrazinecarbonyl chloride (**15**)

To a suspension of bis-hydrazone **14** (215 mg, 0.50 mmol) in CH_2_Cl_2_ (10 mL) cooled to 0 °C, Et_3_N (0.28 mL, 2.0 mmol) and triphosgene (130 mg, 0.44 mmol) were added. The resulting mixture was stirred for 1 h at 0 °C and then 30 min at rt. The solvent was evaporated, and product **15** was directly used in the next step without further purification.

#### 4*-i*-Propoxyphenylhydrazine hydrochloride (**16·HCl**)

The mixture of conc. HCl (6 mL) and H_2_O (6 mL) was cooled to −5 °C and then 4-*i*-propoxyaniline hydrochloride (2.42 g, 13.0 mmol) and solution of NaNO_2_ (0.96 g, 14.0 mmol) in H_2_O (3 mL) were slowly added with vigorous stirring. Then, a solution of SnCl_2_**·**2H_2_O (6.60 g, 25.6 mmol) in conc. HCl (15 mL) was added dropwise, and the reaction mixture was stirred for 30 min. The resulting precipitate was filtered and washed with a cooled, saturated solution of NaCl and Et_2_O. The solid was dried under vacuum and recrystallized from MeOH/Et_2_O, giving 1.86 g (9.20 mmol, 71% yield) of hydrazine hydrochloride **16·HCl** as a white microcrystalline solid: mp 168–169 °C (MeOH/Et_2_O); ^1^H NMR (DMSO-*d*_6_, 600 MHz) δ 10.14 (bs, 3H), 7.91 (br s, 1H), 7.00 (d, *J* = 8.9 Hz, 2H), 6.85 (d, *J* = 8.8 Hz, 2H), 4.49 (sept, *J* = 5.9 Hz, 1H), 1.22 (d, *J* = 6.1 Hz, 6H); ^13^C{^1^H} NMR (DMSO-*d*_6_, 151 MHz) δ 152.8, 138.8, 117.2, 116.4, 69.6, 21.8; IR (KBr) ν 3227 (N–H), 2971, 2908, 2673, 1591, 1508, 1236, 1125 cm^–1^. Anal. Calcd for C_9_H_15_ClN_2_O: C, 53.33; H, 7.46; N, 13.82. Found: C, 53.44; H, 7.44; N, 13.91.

#### 1,5-Di(4-*i*-propoxyphenyl)-3-phenyl-1,2,4,5-tetrazinan-6-one (**17**)

A solution of carbonohydrazide **5** (717 mg, 2.00 mmol) and benzaldehyde (217 mg, 2.05 mmol) in EtOH (10 mL) was refluxed for 6 h under Ar atmosphere. After cooling to rt, the reaction mixture was kept overnight in the freezer. The resulting precipitate was filtered, washed with cold EtOH (2 × 10 mL) and Et_2_O (2 × 10 mL), and dried under vacuum, giving 510 mg (1.186 mmol, 59% yield) of tetrazane **17** as a pale-rose solid: mp 190–192 °C (EtOH); ^1^H NMR (DMSO-*d*_6_, 600 MHz) δ 7.56 (d, *J* = 7.3 Hz, 2H), 7.43 (d, *J* = 9.0 Hz, 4H), 7.31–7.39 (m, 3H), 6.86 (d, *J* = 9.0 Hz, 4H), 6.26 (d, *J* = 9.3 Hz, 2H), 5.33 (t, *J* = 9.3 Hz, 1H), 4.56 (sept, *J* = 6.0 Hz, 2H), 1.26 (d, *J* = 6.1 Hz, 12H); ^13^C{^1^H} NMR (DMSO-*d*_6_, 151 MHz) δ 154.3, 153.9, 137.0, 136.1, 128.2, 128.1, 126.9, 124.0, 115.0, 71.5, 69.3, 21.8; IR (KBr) ν 3234 (N–H), 2968, 1622 (C=O), 1603, 1502, 1369, 1239 cm^–1^. Anal. Calcd for C_26_H_30_N_4_O_3_: C, 69.93; H, 6.77; N, 12.55. Found: C, 69.87; H, 6.72; N, 12.75.

#### Phenyl 4-*i*-propoxybenzhydrazone (**18**)

Phenylhydrazine (1.12 g, 10.4 mmol) and 4-*i*-propoxybenzaldehyde (**11**, 1.64 g, 10.0 mmol) were dissolved in EtOH (15 mL). Then, a catalytic amount of AcOH was added and the mixture was stirred for 10 h at rt. The resulting precipitate was filtered, then the filtrate concentrated and placed in a freezer. Next, a portion of the precipitate was filtrated. Precipitates were combined and dried under vacuum, giving 2.02 g (7.95 mmol, 79% yield) of hydrazone **18** as a pale yellow solid: mp 105–107 °C; ^1^H NMR (CDCl_3_, 600 MHz) δ 7.53 (s, 1H), 7.47 (d, *J* = 8.7 Hz, 2H), 7.15–7.17 (m, 2H), 6.99 (d, *J* = 7.7 Hz, 2H), 6.79 (d, *J* = 8.7 Hz, 2H), 6.75 (t, *J* = 7.3 Hz, 1H), 4.47 (sept, *J* = 6.1 Hz, 1H), 1.25 (d, *J* = 6.1 Hz, 6H); ^13^C{^1^H} NMR (CDCl_3_, 151 MHz) δ 158.6, 145.2, 137.7, 129.4, 128.1, 127.8, 120.0, 116.2, 112.9, 70.2, 22.2; IR (KBr) ν 3338 (N–H), 2977, 1594, 1505, 1236, 1138, 1112 cm^–1^; MS (ESI-TOF) *m*/*z* 255 (100, [M + H]^+^). Anal. Calcd for C_16_H_18_N_2_O: C, 75.56; H, 7.13; N, 11.01. Found: C, 75.41; H, 7.34; N, 11.01.

#### Phenyl 4-*i*-propoxybenzhydrazide carbamoyl chloride (**19**)

Hydrazone **18** (762 mg, 3.0 mmol) and Et_3_N (404 mg, 4.00 mmol) were added dropwise to a solution of triphosgene (390 mg, 1.31 mmol) in anhydrous CH_2_Cl_2_ at 0 °C. The resulting mixture was stirred for 1 h at 0 °C and then 30 min at rt. The solvent was evaporated and the product purified by flash column chromatography (SiO_2_, pet. ether/CH_2_Cl_2_, gradient 0–70%), giving 860 mg (2.72 mmol, 90% yield) of carbamoyl chloride **19** as a yellowish oil, which was used in the next step without further purification.

#### 1,3-Di(4-*i*-propoxyphenyl)-5-phenyl-1,2,4,5-tetrazinan-6-one (**20**)

A mixture of carbamoyl chloride **19** (630 mg, 2.00 mmol), 4-isopropoxyphenylhydrazine hydrochloride (**16·HCl**, 447 mg, 2.20 mmol), and Et_3_N (455 mg, 4.50 mmol) in EtOH (5 mL) was reflux for 10 h under Ar atmosphere. After cooling to rt, the reaction mixture was concentrated to half of the volume and kept overnight in the freezer. The resulting precipitate was filtered, washed with cold EtOH (2 × 5 mL) and Et_2_O (2 × 5 mL), and dried under vacuum, giving 535 mg (1.20 mmol, 60% yield) of tetrazane **20** as a yellowish solid: mp 124–127 °C (EtOH); ^1^H NMR (DMSO-*d*_6_, 600 MHz) δ 7.60 (d, *J* = 8.4 Hz, 2H), 7.43 (d, *J* = 8.9 Hz, 4H), 7.30 (t, *J* = 7.5 Hz, 2H), 7.05 (t, *J* = 7.4 Hz, 1H), 6.88 (t, *J* = 9.2 Hz, 4H), 6.24 (d, *J* = 5.– Hz, 1H), 6.22 (d, *J* = 6.1 Hz, 1H), 5.27 (t, *J* = 9.4 Hz, 1H), 4.61 (sept, *J* = 6.0 Hz, 1H), 4.56 (sept, *J* = 6.1 Hz, 1H), 1.26 (d, *J* = 6.0 Hz, 6H), 1.25 (d, *J* = 6.0 Hz, 6H); ^13^C{^1^H} NMR (DMSO-*d*_6_, 151 MHz) δ 157.3, 155.3, 154.0, 143.1, 135.9, 129.0, 128.2, 127.9, 124.0, 123.2, 121.5, 115.3, 115.1, 71.7, 69.4, 69.1, 21.87, 21.86, 21.8; and IR (KBr) ν 3230 (N–H), 2977, 1622 (C=O), 1507, 1243 cm^–1^. Anal. Calcd for C_26_H_30_N_4_O_3_: C, 69.93; H, 6.77; N, 12.55. Found: C, 69.97; H, 6.72; N, 12.41.

### X-ray Data Collection

Suitable crystals of **2-C** and **1-Np** obtained by slow evaporation of heptane/CH_2_Cl_2_ and CH_2_Cl_2_/hexane solutions, respectively, were selected for X-ray diffraction experiments at 100(2) K. Diffraction data were collected on the Agilent Technologies SuperNova Dual Source diffractometer equipped with an Atlas detector with Cu Kα radiation (λ = 1.54184 Å) using CrysAlis Pro software.^77^ The structural determination procedure was carried out using the SHELX package.^78^ The structures were solved with direct methods, and then successive least-squares refinement was carried out based on the full-matrix least-squares method on *F*^2^ using the SHELXL program^78^ within the Olex2 program.^79^ All H atoms were positioned geometrically and constrained to ride on their parent atoms. All molecular interactions in the crystal were identified using PLATON program.^80^ Full details are provided in the Supporting Information.

### Electrochemical Measurements

The electrochemical characterization of radicals **1** and **2** was conducted using a Metrohm Autolab PGSTAT128N potentiostat/galvanostat instrument. Radicals **1** and **2** were dissolved in dry, spectroscopic-grade CH_2_Cl_2_ (0.5 mM) in the presence of [*n*-Bu_4_N]^+^[PF_6_]^−^ as an electrolyte (50 mM), and the resulting solution was degassed by purging with Ar gas for 20 min. A three-electrode electrochemical cell was used with glassy carbon disk as the working electrode (ϕ = 2 mm, alumina polished), Pt wire as the counter electrode, and Ag/AgCl wire as the pseudoreference electrode. All samples were measured without internal reference followed by measurements with added ferrocene as the internal reference with a scan rate of 50 mV s^–1^ at ca. 20 °C. The oxidation potential for the Fc/Fc^+^ couple was set at 0.0 V. Cyclic voltammetry (CV) measurements were started from 0.0 V in the oxidative direction.

### Variable Temperature EPR Spectroscopy

Variable temperature EPR spectra for diradicals **1** were recorded on a X-band EMX-Nano Band spectrometer equipped with a frequency counter and nitrogen flow temperature control (100 to 320 K) in degassed solid polystyrene solution (4.42–5.35 mM). At 120 K, they exhibited patterns consistent with randomly oriented triplets contaminated with a signal from the doublet impurity (the middle singlet). No half-field transition |Δ*m*_s_| = 2 was observed for any of the diradicals. Therefore, variable temperature EPR spectra were double integrated and the resulting relative intensities (DI_rel_) were analyzed with the Bleaney–Bowers model^59^ (eq 1). Details are provided in the Supporting Information.

### SQUID Measurements

Magnetic susceptibility of polycrystalline samples of diradicals **1** and monoradicals **2** was measured in a polycarbonate capsule fitted in a plastic straw as a function of temperature in cooling (300 K → 2 K) and then in heating (2 K → 400 K) modes with 0.2 K increments in a range of 2 to 10 K, 1 K increments in the range of 11 to 49 K, and 5 K increments in the range of 50 to 300 K) at 0.60 T, using a SQUID magnetometer (Quantum Design MPMS-XL-7T). No significant differences in magnetic susceptibility were observed for data collected in heating and cooling modes. Analysis was conducted for data obtained on the first heating run. Details of data collection, processing, and analysis are provided in the Supporting Information.

## Data Availability

The data underlying this study are available in the published article and its Supporting Information.

## Supporting Information Available

Additional synthetic procedures, NMR spectra, details of XRD, UV–vis, EPR spectroscopy, magnetic measurements, and results of DFT calculations (PDF)
FAIR data, including the primary NMR FID files, for compounds **4**–**7**, **9**–**18**, **20** (ZIP)
