# Supplementary material for: Electronic and Magnetic Interactions in 6-Oxoverdazyl Diradicals: Connection through N(1) vs C(3) Revisited
Source: J Org Chem. 2024 Apr 16;89(9):6306–21. doi: 10.1021/acs.joc.4c00303 (PMC11077500; doi:10.1021/acs.joc.4c00303)
Supplement: Supplementary file 1 — jo4c00303_si_001.pdf [file jo4c00303_si_001.pdf]

Supporting Information for

Electronic and magnetic interactions in 6-oxoverdazyl diradicals: Connection through N(1) *vs* C(3) revisited

Agnieszka Bodzioch,[§] Emilia Obijalska,[#] Rafał Jakubowski,[§] Małgorzata Celeda,[#] Anita Gardias,[†] Damian Trzybiński,[‡] Paweł Tokarz,[#] Jacek Szczytko, [†] Krzysztof Woźniak,[‡] and Piotr Kaszyński* [§#¶]

Corresponding Author: piotr.kaszynski@cbmm.lodz.pl

[§] Centre of Molecular and Macromolecular Studies, Polish Academy of Sciences, 90-363 Łódź, Poland

[#] Faculty of Chemistry, University of Łódź, 91-403 Łódź, Poland

[†] Institute of Experimental Physics, Faculty of Physics, University of Warsaw, 02-093 Warsaw, Poland

[‡] Biological and Chemical Research Centre, University of Warsaw, 02-089 Warsaw, Poland

[¶] Department of Chemistry, Middle Tennessee State University, Murfreesboro, TN, 37132, USA

Table of contents **Page**

1. Additional synthetic details ……S2
2. NMR spectra ……S5
3. XRD data collection and refinement ……S24
4. UV-vis spectroscopy ……S32
5. Electrochemical results ……S36
6. EPR spectroscopy ……S38
   a) liquid solution spectroscopy …S38
   b) solid solution spectroscopy …S39
7. Magnetization measurements and data analysis ……S48
   a) diradical **1-Cp** ...S50
   b) diradical **1-Cm** ...S51
   c) diradical **1-Np** ...S52
   d) diradical **1-Nm** ...S54
   e) radical **2-C** ...S55
   f) radical **2-N** ...S55
8. Computational details ……S57
   a) ΔE_{S-T} energy gap calculations …S57
   b) exchange interaction energy in XRD structures …S58
   c) diradicaloid character …S58
   d) electronic excitation data …S59
   e) partial output from TD-DFT calculation …S60
   f) contours of MOs involved in low energy excitation in **1** and **2** …S67
9. Archive for DFT calculations for model compounds ……S69
10. References ……S82

1. Additional synthetic details.

Optimization of N-arylation of benzyl carbazate (8).

It was observed that arylation of carbazate **8** gives two isomers. Therefore this reaction was optimized to maximize the yield of the desired N(1)-aryl derivative using 4-iodoanisole as the model aryl iodide as shown in Scheme S1. Conditions used and the reaction outcomes are listed in Table S1. The originally reported procedure[1] was modified by changing the solvent and temperature which led to the varied amounts of the two isomers **A** and **B** (Scheme 1). The best result was obtained for the CuI, Cs_2CO_3 in DMF at 50 °C conditions. The crude reaction mixture was separated by column chromatography (hexane/AcOEt, 2:3) giving the desired isomer **A** in 66% yield [^{1}H NMR ($CDCl_3$, 500 MHz) δ 7.28-7.40 (m, 7H), 6.83-6.89 (m, 2H), 5.20 (s, 2H), 4.53 (bs, 2H), 3.80 (s, 3H)] and isomer **B** in 22% yield [^{1}H NMR ($CDCl_3$, 500 MHz) δ 7.31-7.42 (m, 7H), 6.82-6.88 (m, 2H), 6.60 (bs, 1H), 5.19 (s, 2H), 4.70 (s, 1H), 3.78 (s, 3H)]. Similar results were obtained with K_3PO_4 as the base.

Scheme S1. Cu-catalyzed coupling of 4-iodobenzene with benzyl carbazate (**8**).

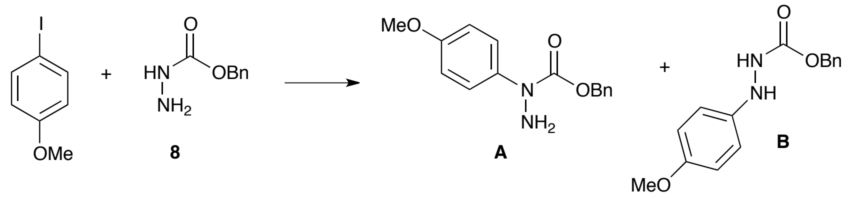

Table S1. Optimization of reaction conditions of Cu-catalyzed coupling of 4-iodobenzene with benzyl carbazate (**8**).

Run	Conditions	Results [a]
1	CuI, L-proline, Cs_2CO_3, DMF, 80 °C, 24 h	Full conversion of 4-iodoanisole, mixture of products, isomer **B** as the major product
2	CuI, L-proline, Cs_2CO_3, DMSO, 80 °C, 24 h	Partial conversion of 4-iodoanisole, mixture of products **A** and **B**
3	CuI, Cs_2CO_3, DMF, 80 °C, 24 h	Partial conversion of 4-iodoanisole, mixture of products **A** and **B**
4	CuI, Cs_2CO_3, DMF, 50 °C, 24 h	Full conversion of 4-iodoanisole, mixture of products, isomer **A** as the major product

[a]Based on NMR.

1-Iodo-4-(*i*-propoxy)benzene (4).[2] Following the literature procedure, isopropyl bromide (7.00 g, 56.9 mmol) and K_2CO_3 (15.11 g, 109.5 mmol) were added to a solution of 4-iodophenol (9.63 g, 43.8 mmol) in anhydrous DMF (20 mL). The resulting mixture was stirred at 100 °C under Ar atmosphere until TLC showed absence of starting materials. The solution was cooled and solvent was removed under reduced pressure. The residue was dissolved in pentane (50 mL) and washed with 1N NaOH (50 mL). The organic layer was dried (Na_2SO_4), filtered and evaporated under reduced pressure. The crude product was purified by flash column chromatography on SiO_2 (petroleum ether/CH_2Cl_2, gradient 0-30%) giving 8.72 g (33.3 mmol, 76% yield) of pure **4** as a colorless low-melting solid: mp 27–28 °C (petroleum ether/CH_2Cl_2); ^{1}H NMR ($CDCl_3$, 600 MHz) δ 7.54 (d, J= 8.9 Hz, 2H), 6.67 (d, J = 8.8 Hz, 2H), 4.49 (sept, J= 6.1 Hz, 1H), 1.32 (d, J = 6.1 Hz, 6H); ^{13}C{^{1}H} NMR ($CDCl_3$, 126 MHz) δ 157.9, 138.3, 118.4, 82.5, 70.2, 22.0; IR (KBr) v 2971, 1489, 1245 cm^{-1}; MS (EI) *m/z* 262 (63, [M]$^+$), 220 (100, [M-*i*Pr]$^+$). Anal. Calcd for $C_9H_{11}OI$: C, 41.24; H, 4.23. Found: C, 41.17; H, 4.21.

4-(*i*-Propoxy)benzaldehyde (11).[3] Following the procedure for preparation of iodide **4**, aldehyde **11** (6.50 g, 39.6 mmol, 90% yield) was obtained from 5.37 g (44.0 mmol) of 4-hydroxybenzaldehyde as a yellowish oil; ^{1}H NMR ($CDCl_3$, 600 MHz) δ 9.86 (s, 1H), 7.81 (d, J = 8.1 Hz, 2H), 6.96 (d, J = 8.1 Hz, 2H), 4.66 (sept, J = 6.0 Hz, 1H), 1.37 (d, J = 6.1 Hz, 6H); ^{13}C{^{1}H} NMR ($CDCl_3$, 151 MHz) δ 190.9, 163.3, 132.2, 129.7, 115.7, 70.5, 22.0; IR (KBr) v 2977, 1692 (C=O), 1505, 1268 cm^{-1}.

1,3-Phenylenedihydrazine dihydrochloride (13•HCl).[4] Following the literature procedure, to a mixture of 1,3-phenylenediamine (4.34 g, 40 mmol) in H_2O (44 mL) at –5 °C conc. HCl (156 mL) a solution of $NaNO_2$ (5.78 g, 80 mmol) in H_2O (40 mL) was added dropwise. The resulting mixture was stirred at -5 °C for 45 min and then transferred dropwise to the cooled solution of $SnCl_2$•$2H_2O$ (70 g, 0.31 mol), Sn (0.4 g, 3.4 mmol) in conc. HCl (160 mL). The resulting precipitate was filtered, washed with Et_2O and dried in vacuum. The precipitate was suspended in EtOH and filtrated. The obtained solid (3.6 g), containing about 44% of pure 1,3-phenylenedihydrazine hydrochloride (**13•HCl**), was dried in vacuum. Et_2O was added to the filtrate, the resulting precipitate was filtrated and dried giving 5.15 g of a light pink solid, which contained about 75% of pure **13•HCl** (3.86 g and 46% yield). Concentration of pure 1,3-phenylenedihydrazine hydrochloride (**13•HCl**) in the obtained solids was established by integration of ^{1}H NMR characteristic signal for **13•HCl** (triplet at 7.34 ppm) and for 1,1,2,2,-tetrachloroethane (singlet at 6.48 ppm; see Figure S11) recorded in methanol-d_4 for known

amounts of the solid and the reference. The solid containing 75% of pure **13•HCl** was used in further reactions.

Synthesis of 4-*i*-propoxyaniline hydrochloride.[5] To a solution of 1-(*i*-propoxy)-4-nitrobenzene (1.81 g, 10.0 mmol in MeOH (30 mL) 5% Pd/C (5 mol%, 0.106 g, 0.5 mmol) was added and the resulting mixture was hydrogenated under balloon over 12 h. The mixture was passed through Celite pad and solution was bubbled with gaseous HCl. After evaporation of solvent crude product was purified by crystallization (MeOH/Et_2O, 1:1) giving 1.21 g (6.45 mmol, 65% yield) of 4-*i*-propoxyaniline hydrochloride as a white crystalline solid: mp 207–210 ^oC (MeOH/Et_2O); ^1H NMR (DMSO-d_6, 600 MHz) δ 10.28 (bs, 3H), 7.31 (d, J= 8.9 Hz, 2H), 7.00 (d, J = 8.9 Hz, 2H), 4.60 (sept, J= 6.1 Hz, 1H), 1.26 (d, J= 6.1 Hz, 6H); $^{13}C\{^1H\}$ NMR (DMSO-d_6, 151 MHz) δ 156.9, 124.4, 124.0, 116.4, 69.6, 21.7; IR (KBr) v 2980, 2873, 2613, 2585, 1999, 1511, 1258 cm^{-1}; MS (ESI-TOF) *m/z* 152 (100, $[M+H]^+$). Anal. Calcd for $C_9H_{14}ClNO$: C, 57.60; H, 7.52; N, 7.46. Found: C, 57.55; H, 7.52; N, 7.59.

Synthesis of 1-(*i*-propoxy)-4-nitrobenzene.[5] Following the procedure for preparation of **4**, 1-(*i*-propoxy)-4-nitrobenzene (6.50 g, 35.9 mmol, 82% yield) was obtained from 6.11 g (44.0 mmol) of 4-nitrophenol as a yellowish low-melting solid: mp 31–33 ^oC (pet. ether/CH_2Cl_2); ^1H NMR ($CDCl_3$, 600 MHz) δ 8.18 (d, J= 9.2 Hz, 2H), 6.92 (d, J = 9.2 Hz, 2H), 4.66 (sept, J= 6.1 Hz, 1H), 1.39 (d, J= 6.1 Hz, 6H); $^{13}C\{^1H\}$ NMR ($CDCl_3$, 151 MHz) δ 163.4, 141.3, 126.1, 115.4, 71.1, 21.9; IR (KBr) v 2980, 2939, 1597, 1515, 1344, 1328, 1268 cm^{-1}; MS (ESI-TOF) *m/z* 182 (100, $[M+H]^+$). Anal. Calcd for $C_9H_{11}NO_3$: C, 59.66; H, 6.12; N, 7.73. Found: C, 59.65; H, 6.08; N, 7.64.

1. NMR spectra

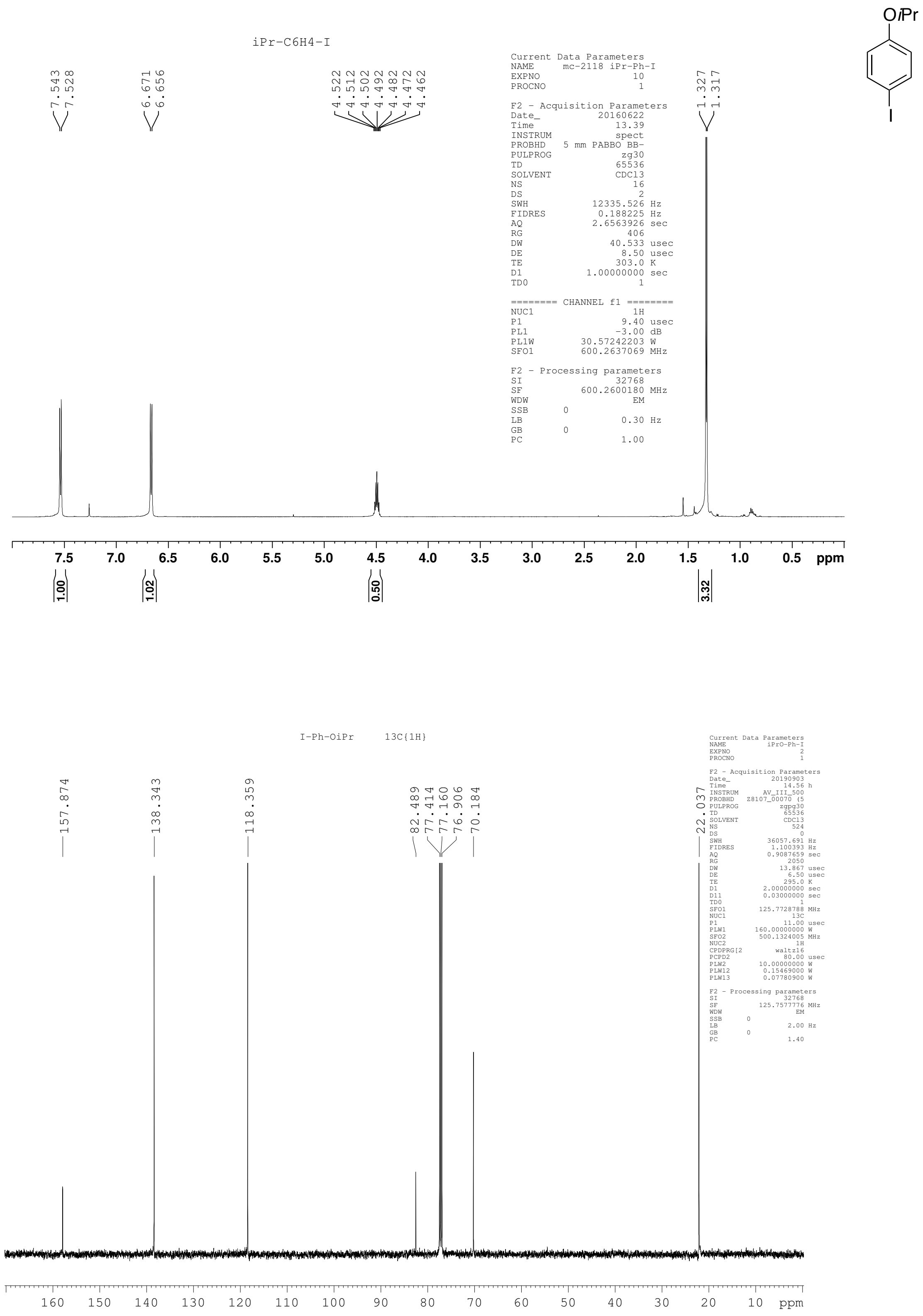

Figure S1. ^{1}H NMR (600 MHz) and ^{13}C{^{1}H} NMR (126 MHz) of **4** ($CDCl_3$).

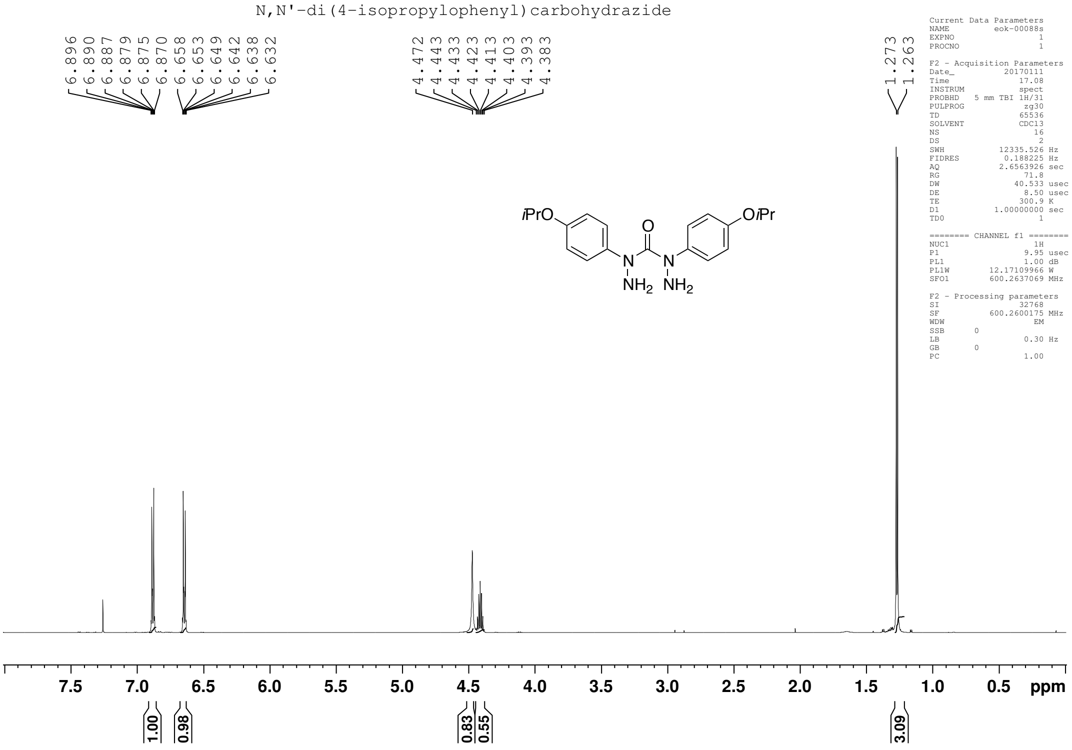

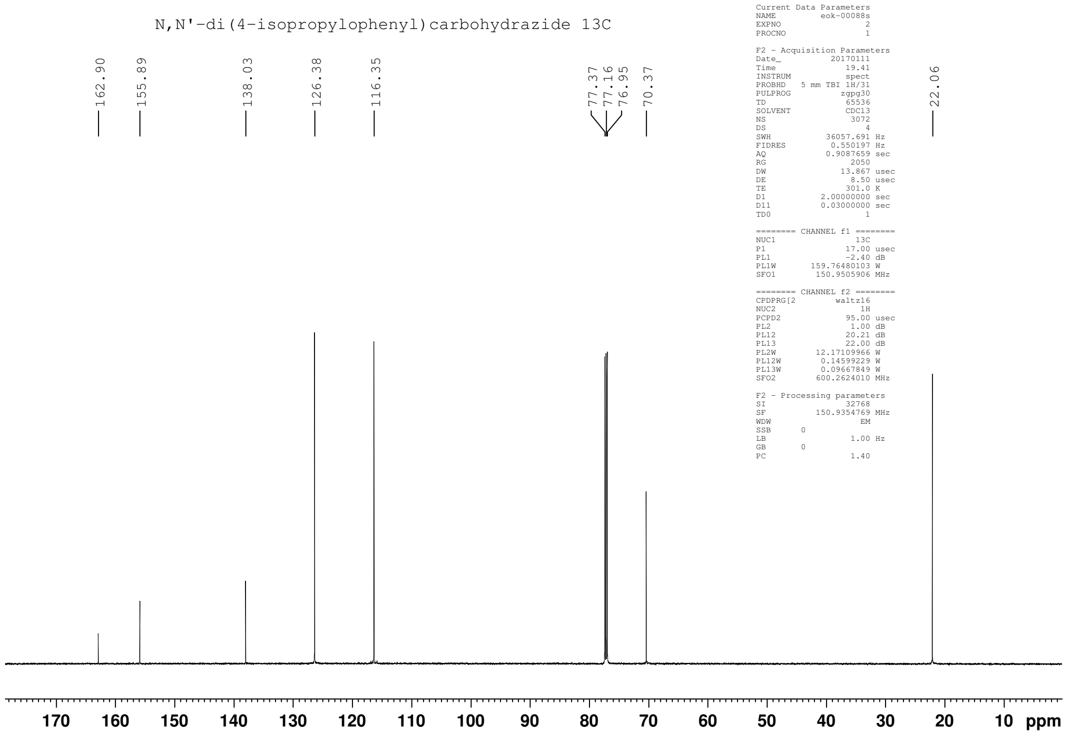

Figure S2. ^{1}H NMR (600 MHz) and ^{13}C{^{1}H} NMR (151 MHz) of **5** ($CDCl_3$).

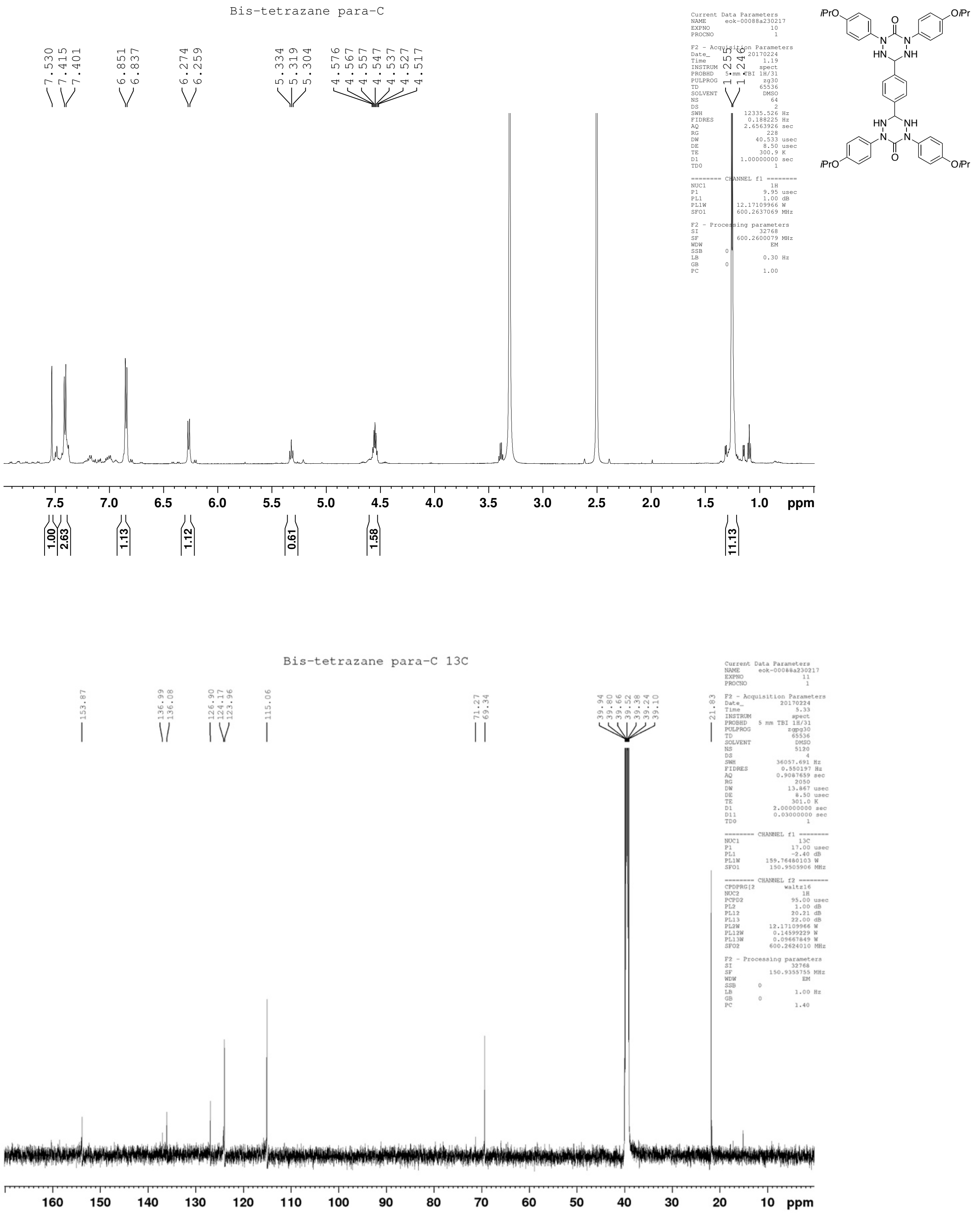

Figure S3. ^{1}H NMR (600 MHz) and $^{13}C\{^{1}H\}$ NMR (151 MHz) of **6p** (DMSO-d_6).

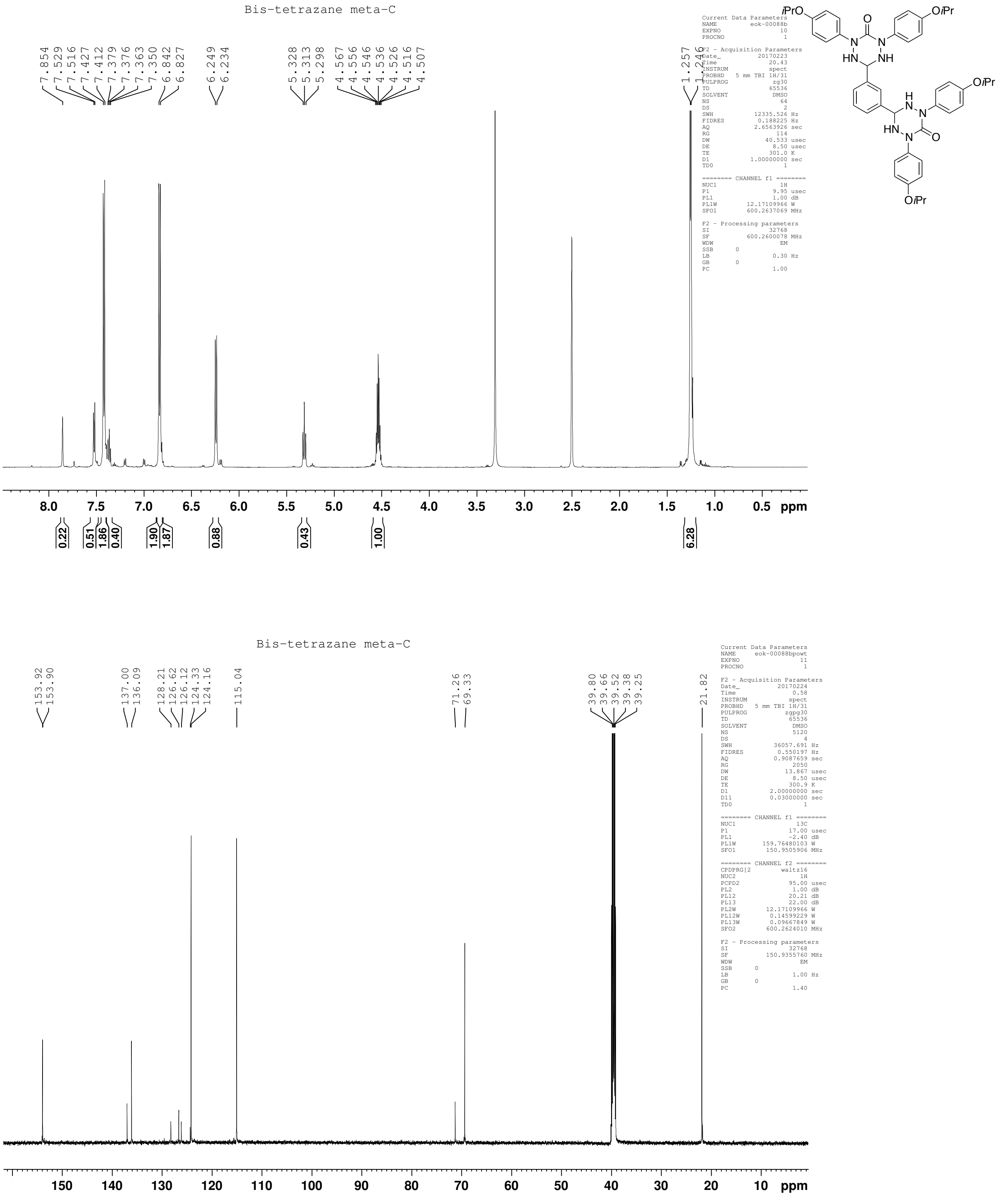

Figure S4. ^{1}H NMR (600 MHz) and ^{13}C{^{1}H} NMR (151 MHz) of **6m** (DMSO-d_6).

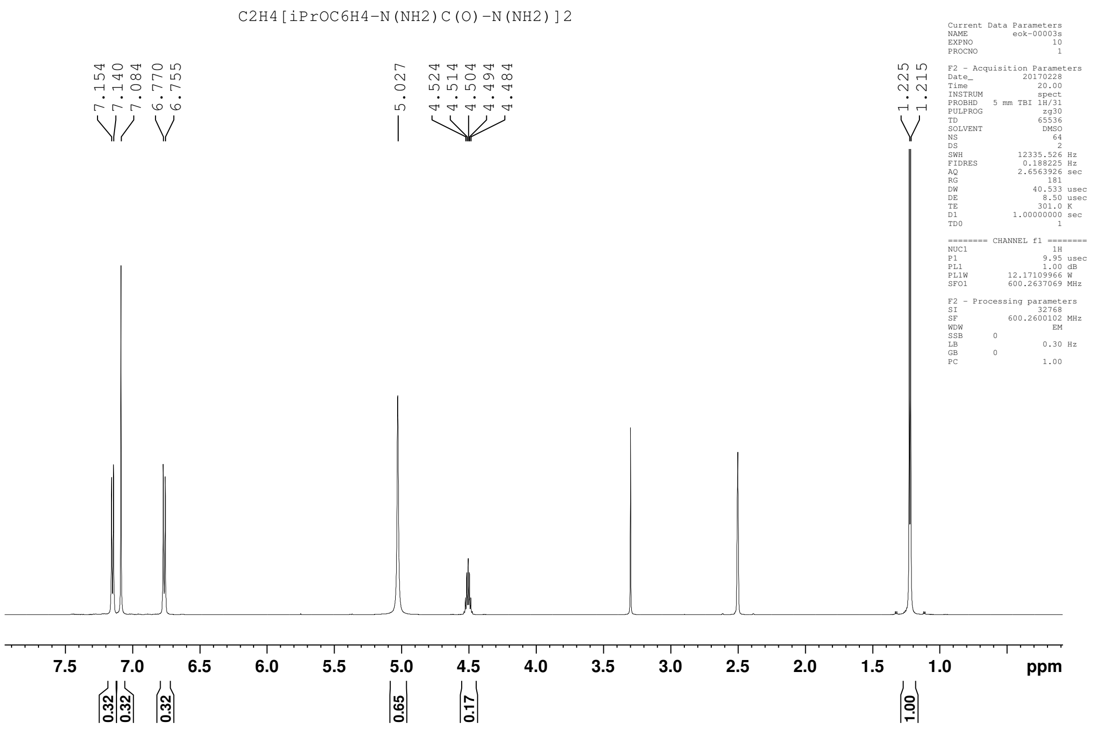

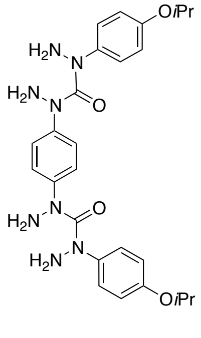

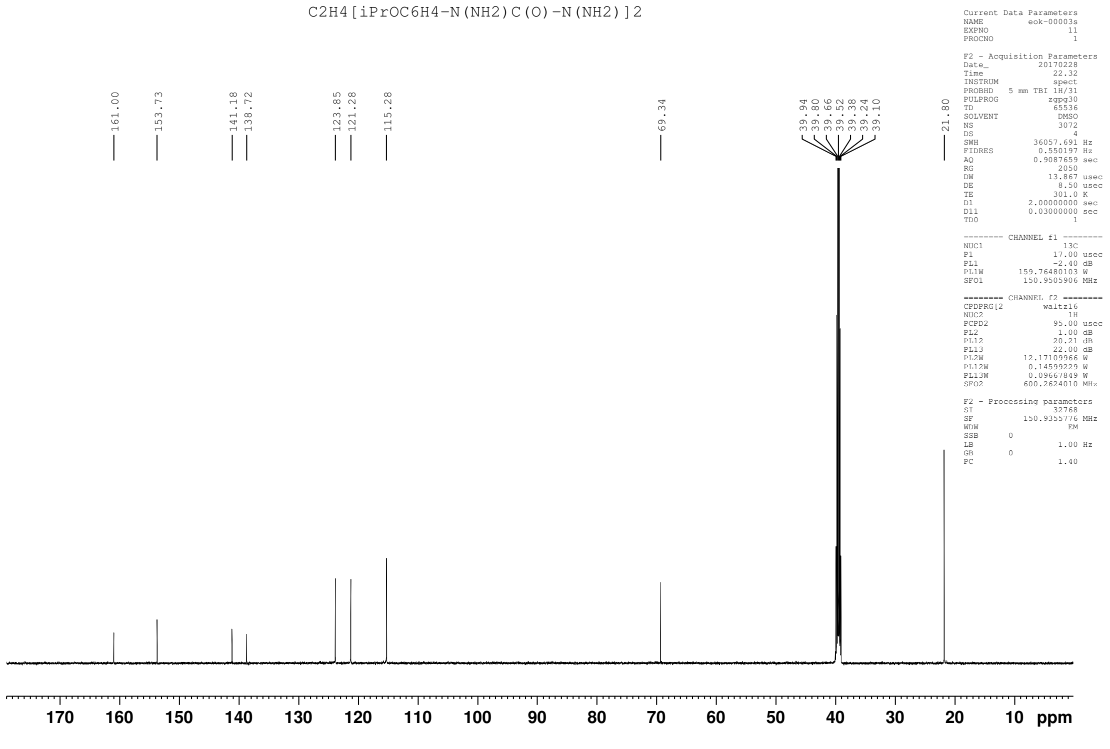

Figure S5. ^{1}H NMR (600 MHz) and $^{13}C\{^{1}H\}$ NMR (151 MHz) **7p** (DMSO-d_6).

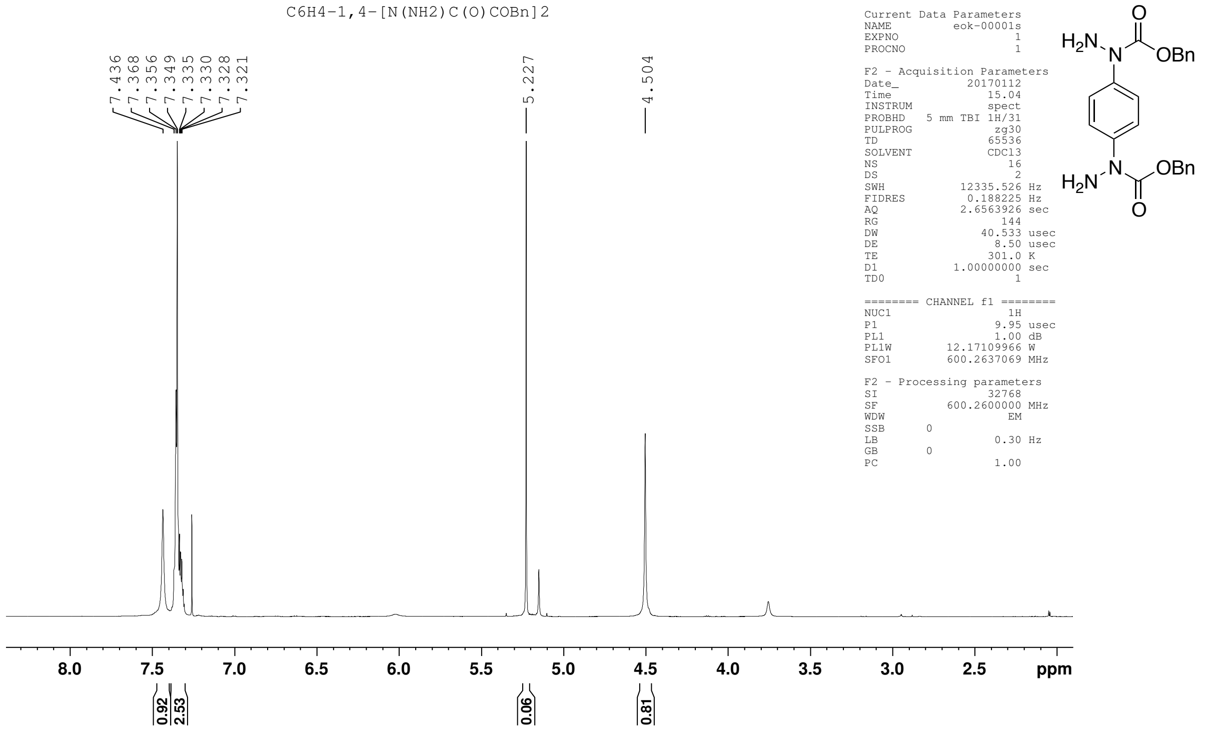

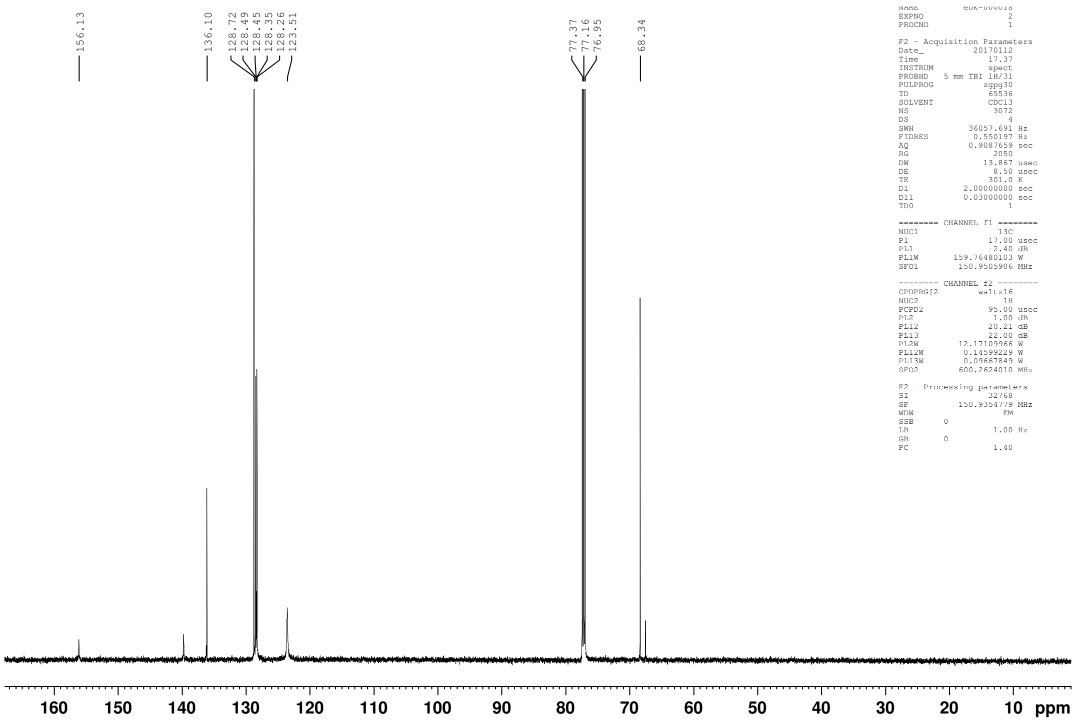

Figure S6. ^{1}H NMR (600 MHz) and $^{13}C\{^{1}H\}$ NMR (151 MHz) of **9p** ($CDCl_3$).

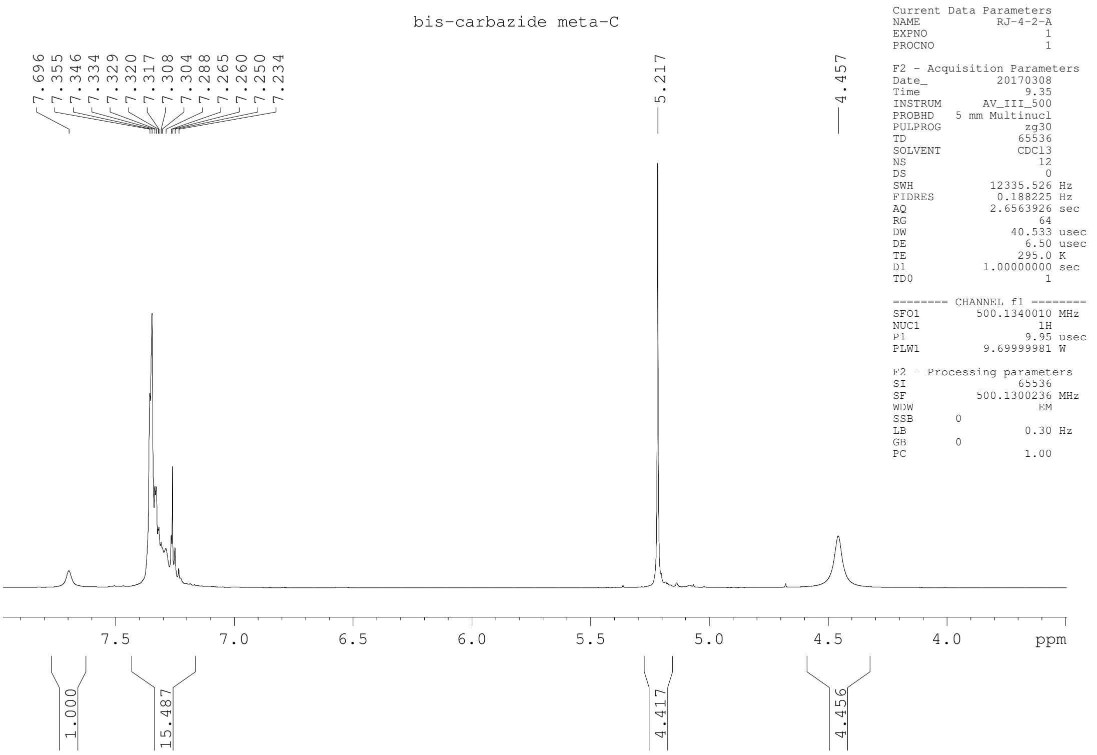

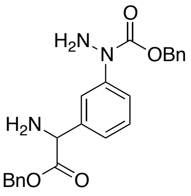

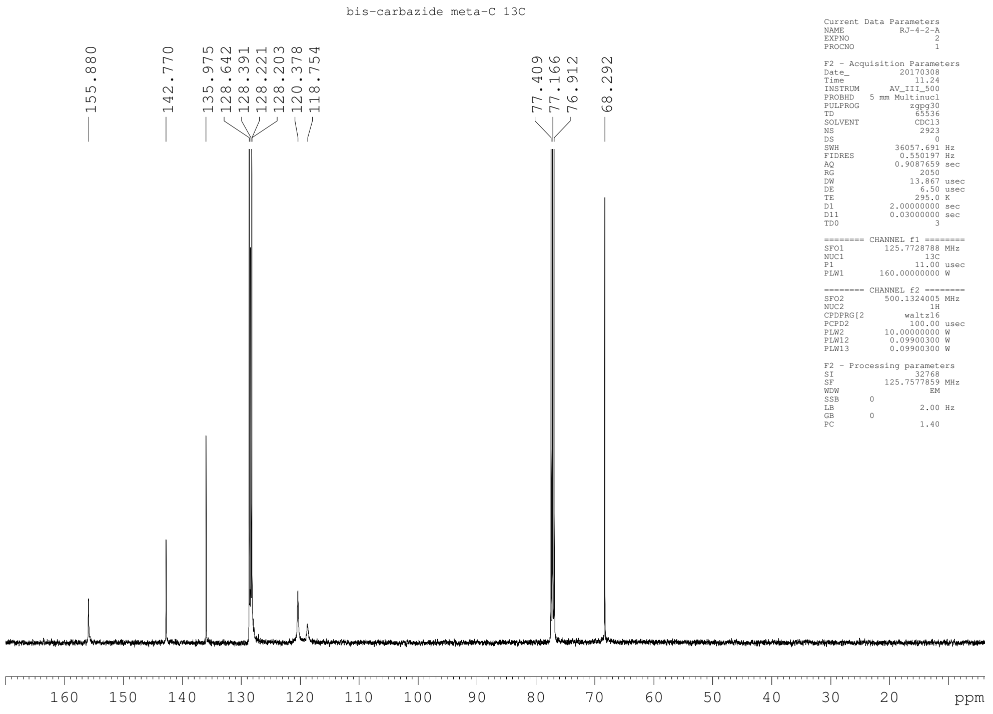

Figure S7. ^{1}H NMR (600 MHz) and ^{13}C{^{1}H} NMR (151 MHz) of **9m** ($CDCl_3$).

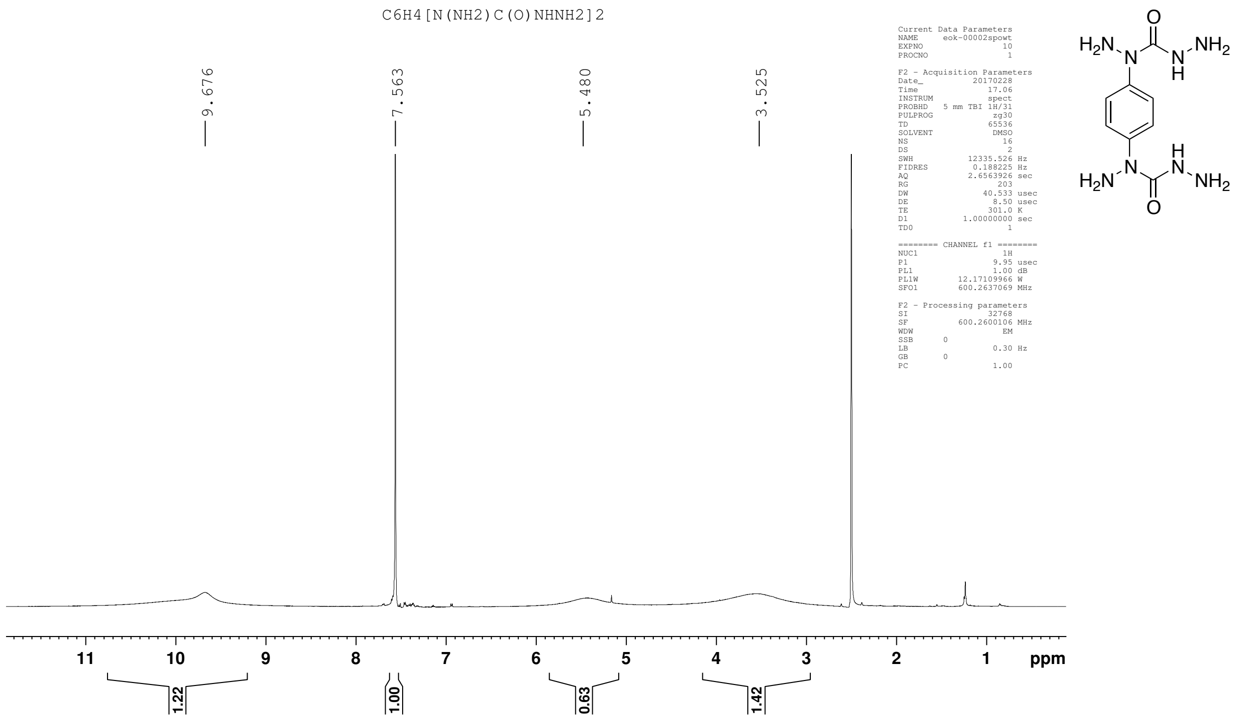

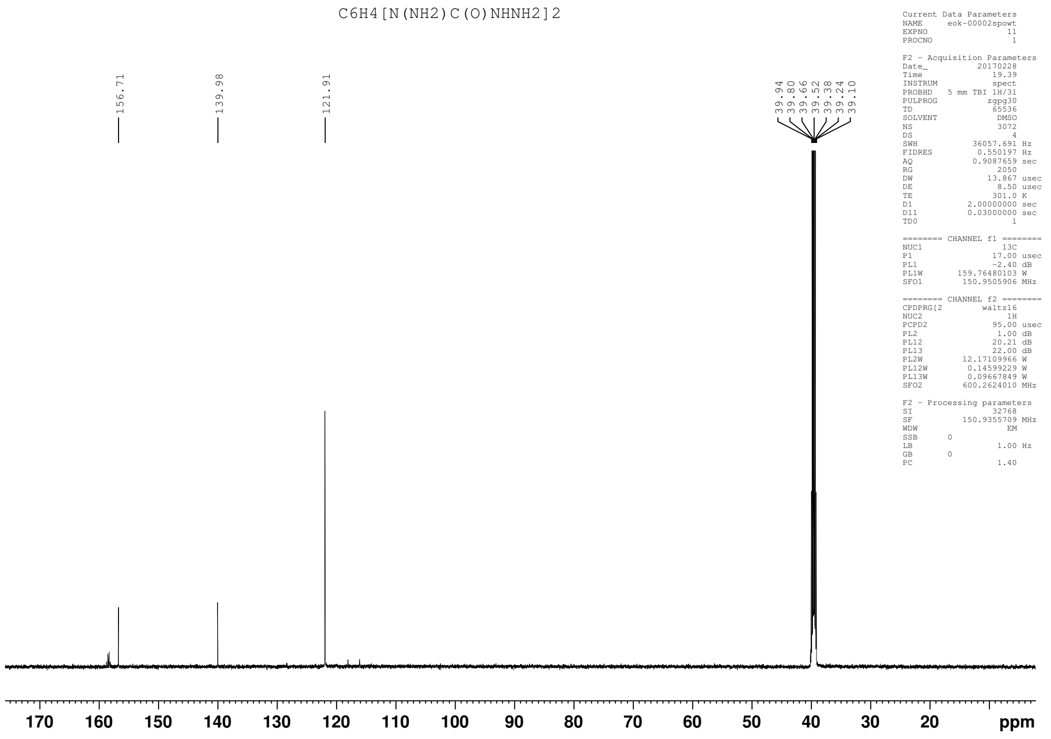

Figure S8. ^{1}H NMR (600 MHz) and ^{13}C{^{1}H} NMR (151 MHz) of **10p** (DMSO-d_6 + CF_3COOD).

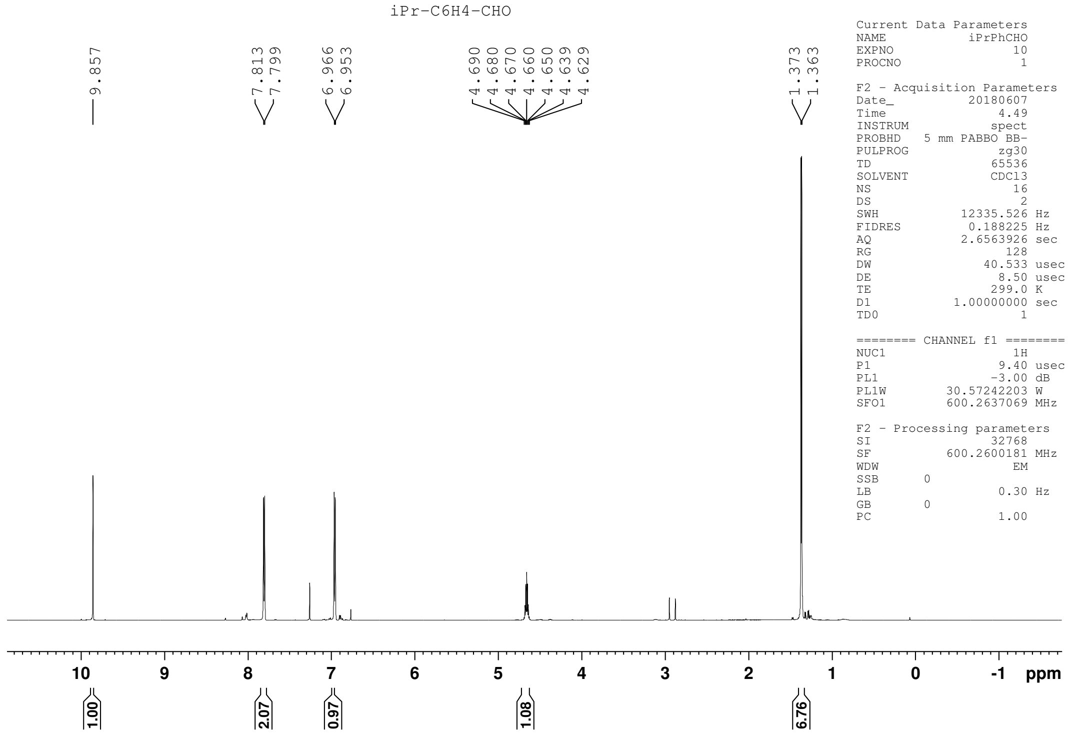

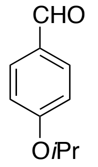

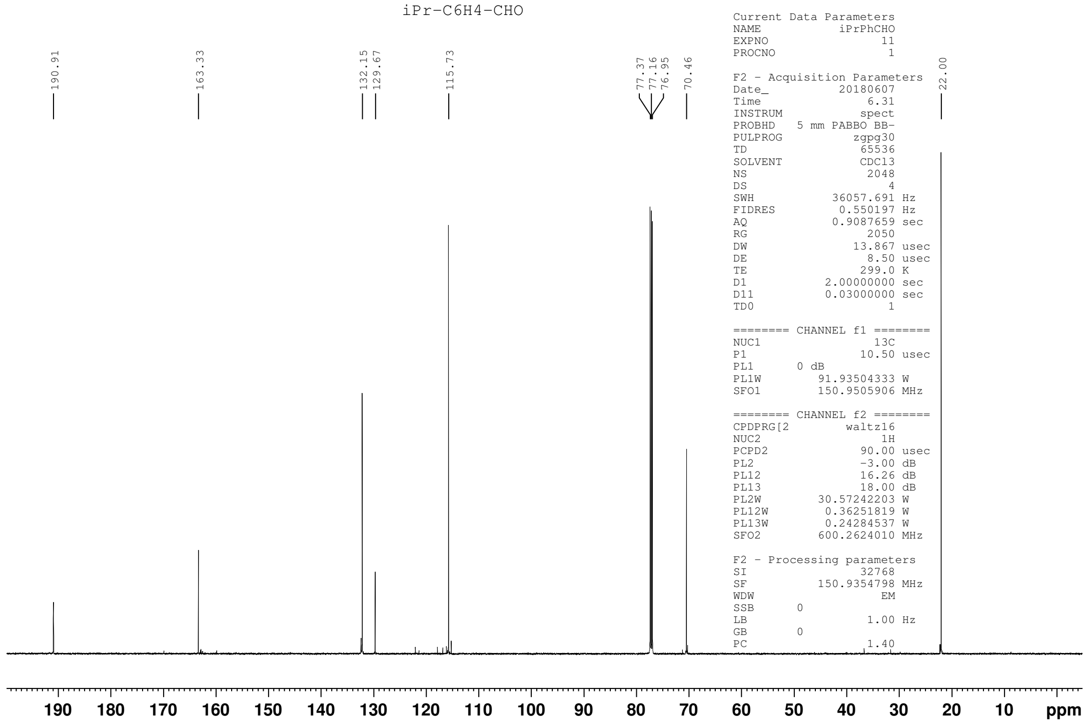

Figure S9. ^{1}H NMR (600 MHz) and ^{13}C{^{1}H} NMR (151 MHz) of **11** ($CDCl_3$).

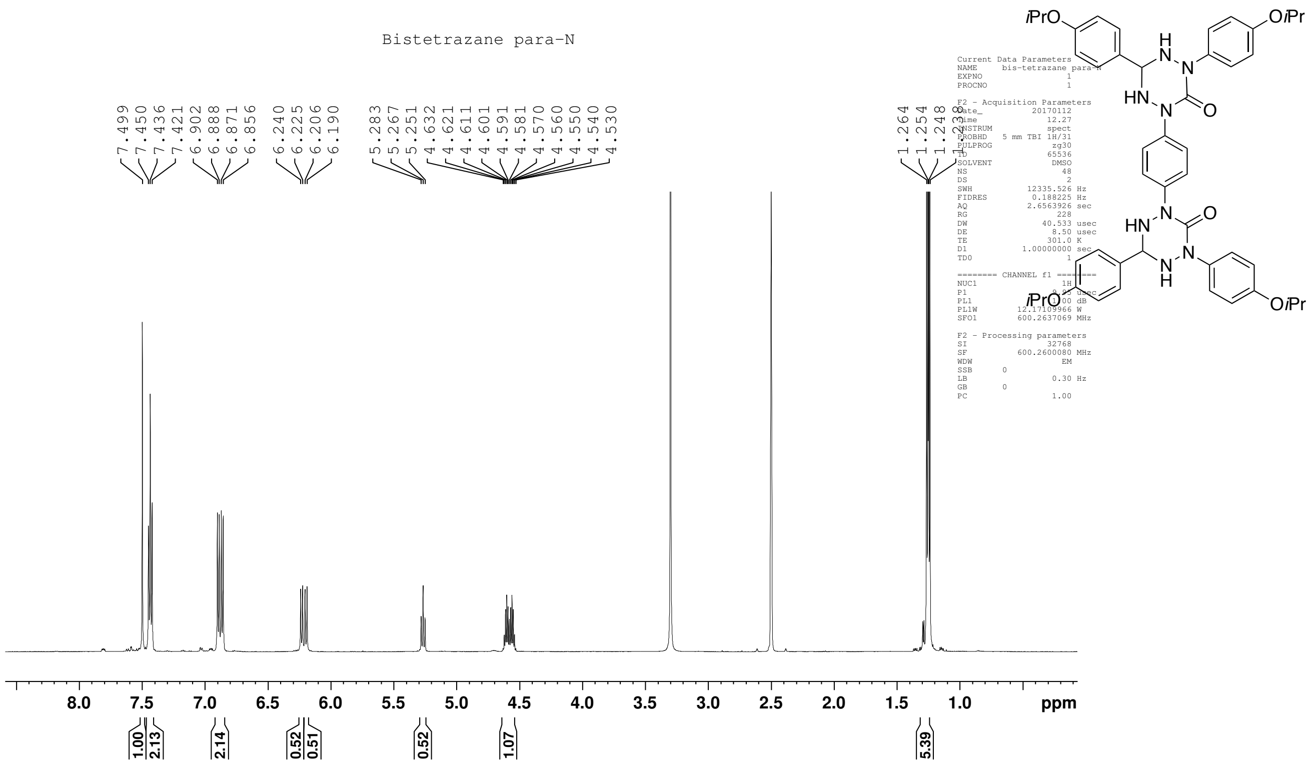

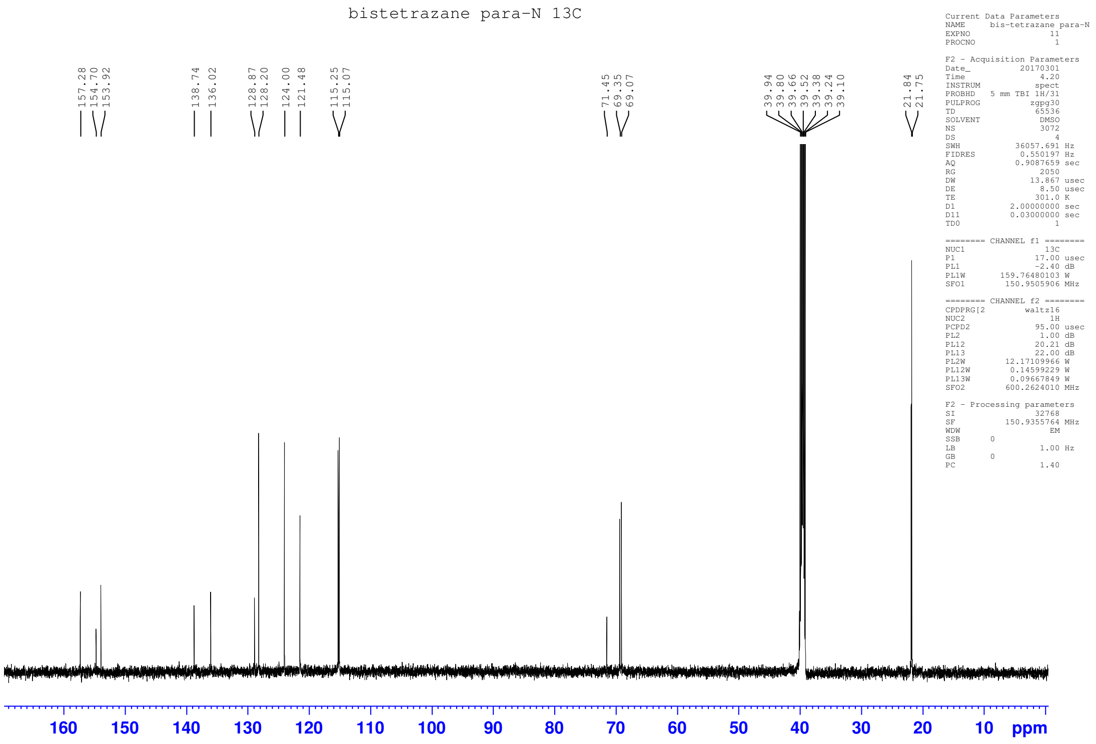

Figure S10. ^{1}H NMR (600 MHz) and $^{13}C\{^{1}H\}$ NMR (151 MHz) of **12p** (DMSO-d_6).

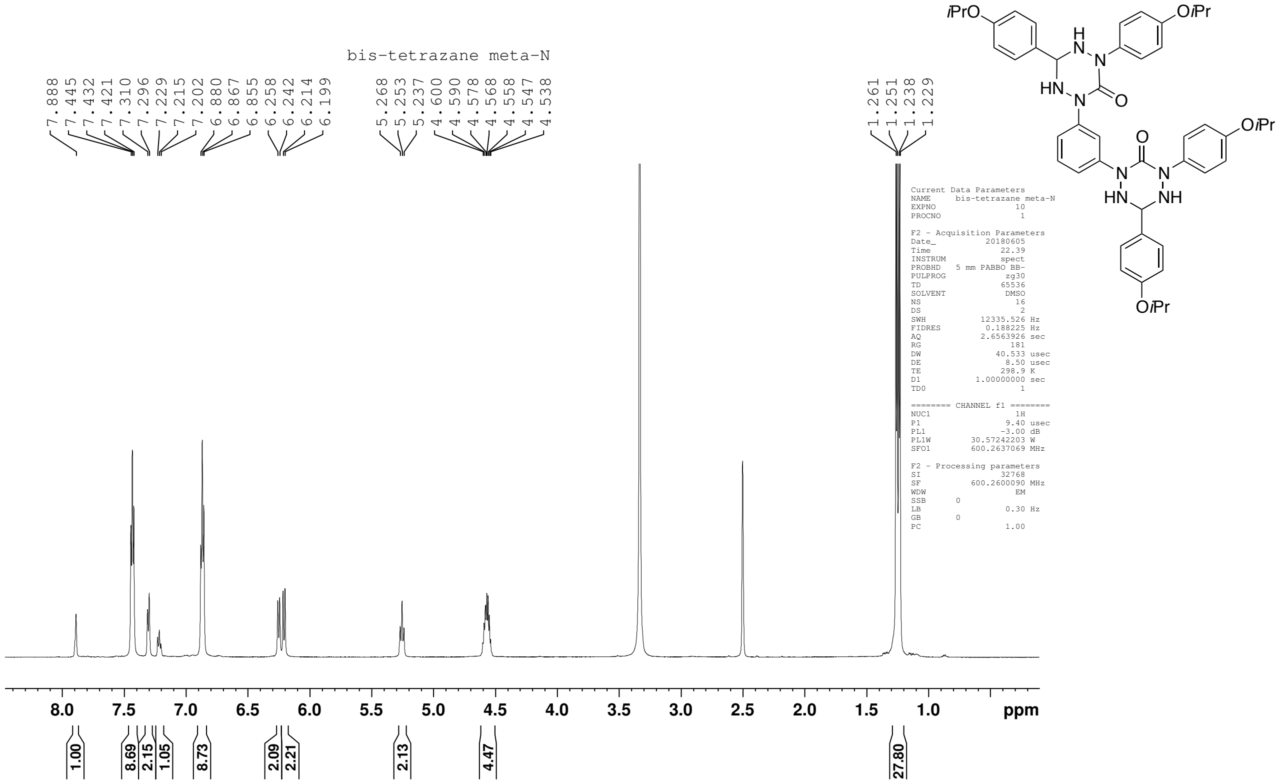

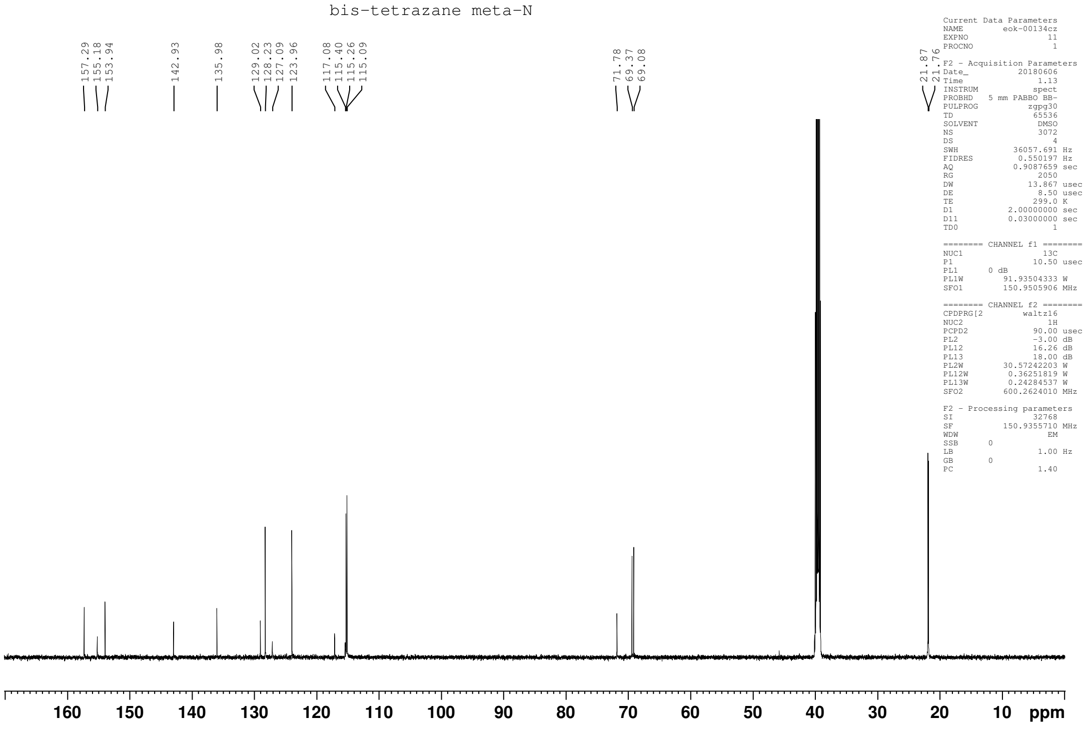

Figure S11. ^{1}H NMR (600 MHz) and ^{13}C{^{1}H} NMR (151 MHz) of **12m** (DMSO-d_6).

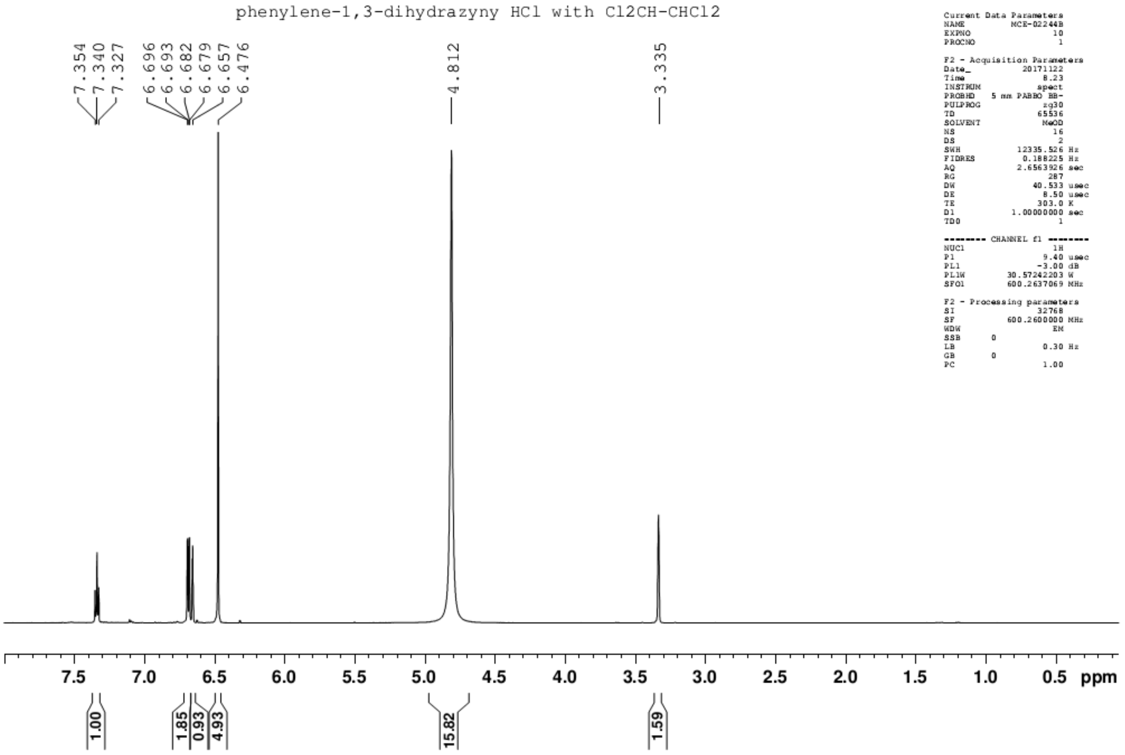

Figure S12. ^{1}H NMR (600 MHz) of **13•HCl** and $(CHCl_2)_2$ (methanol-*d*$_4$).

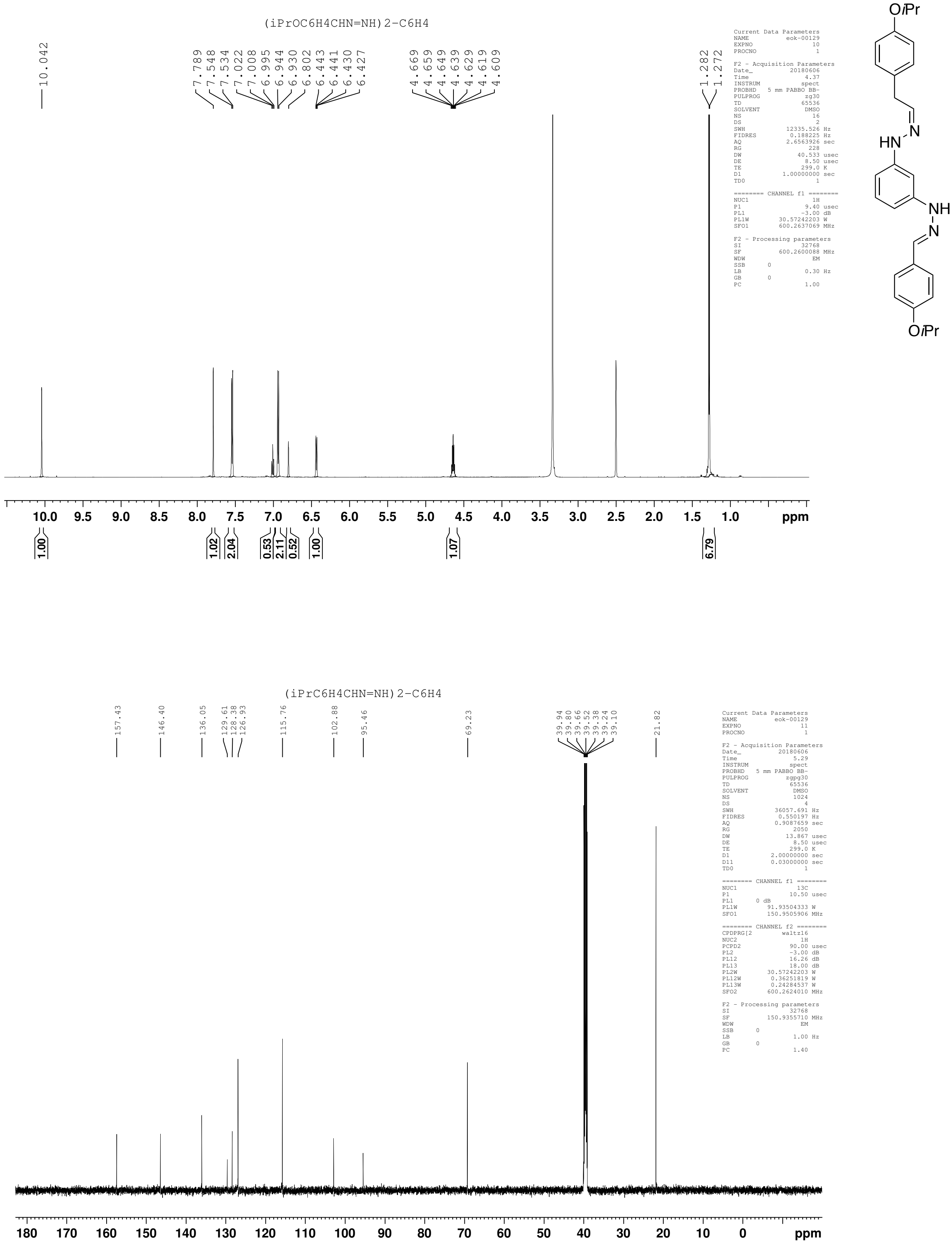

Figure S13. ^{1}H NMR (600 MHz) and ^{13}C{^{1}H} NMR (151 MHz) of **14** (DMSO-d_6).

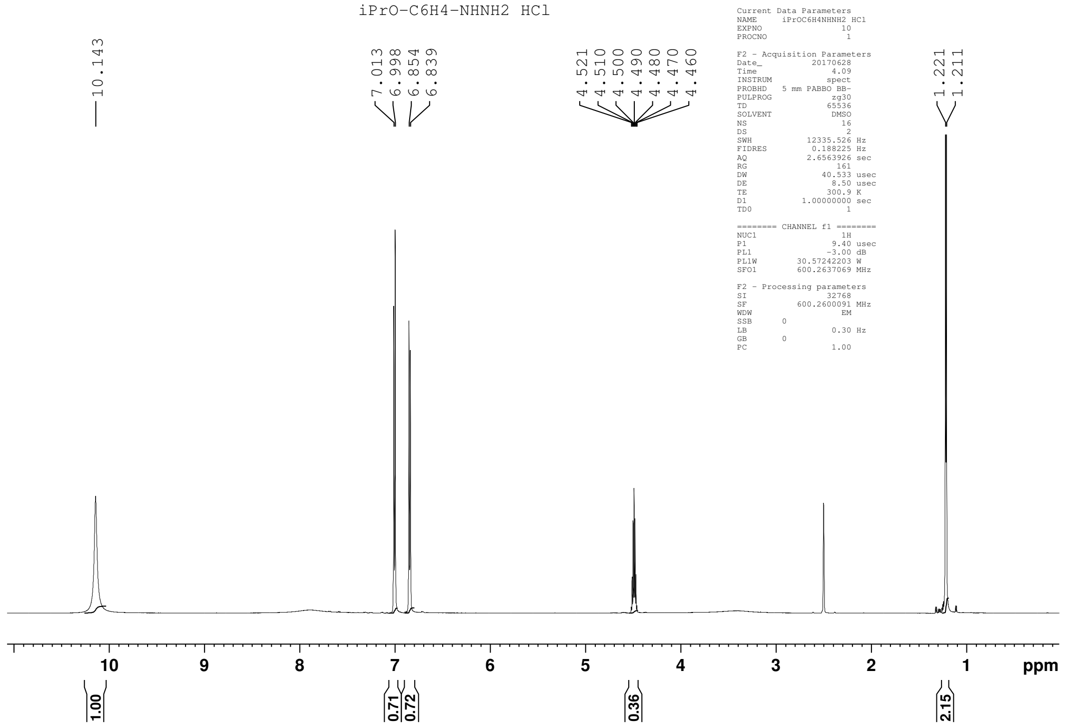

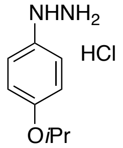

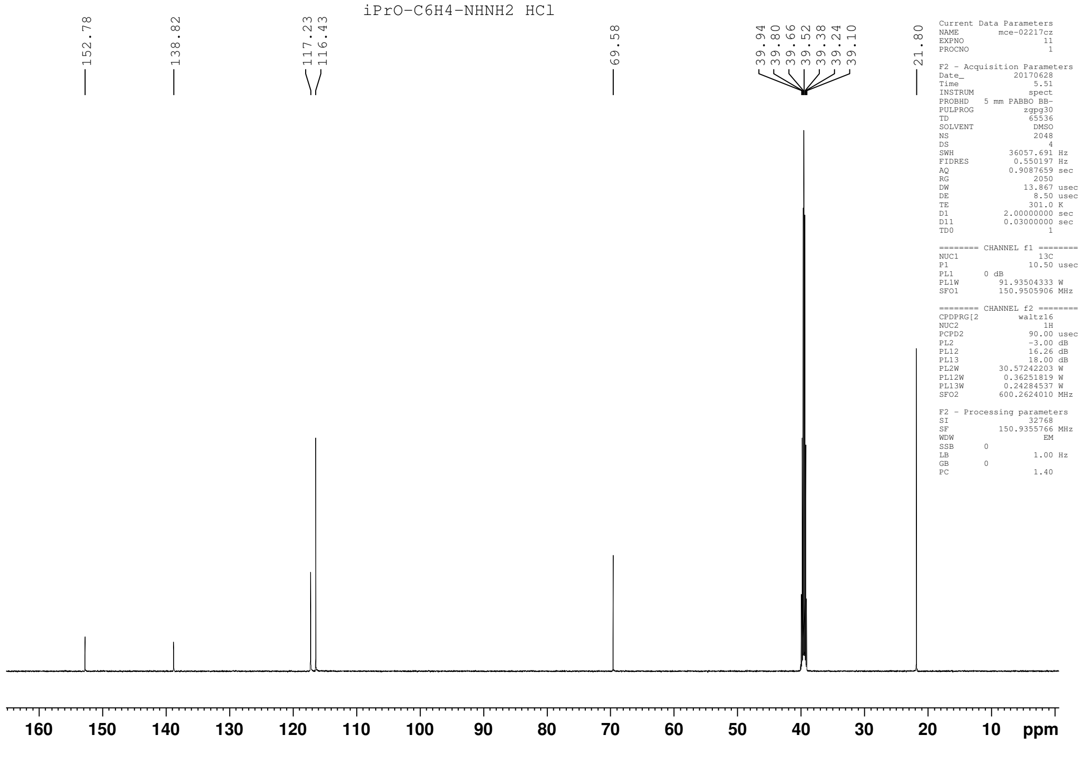

Figure S14. ^{1}H NMR (600 MHz) and ^{13}C{^{1}H} NMR (151 MHz) of **16•HCl** (DMSO-d_6).

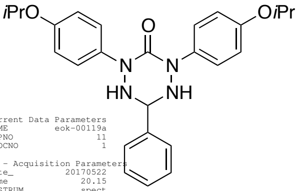

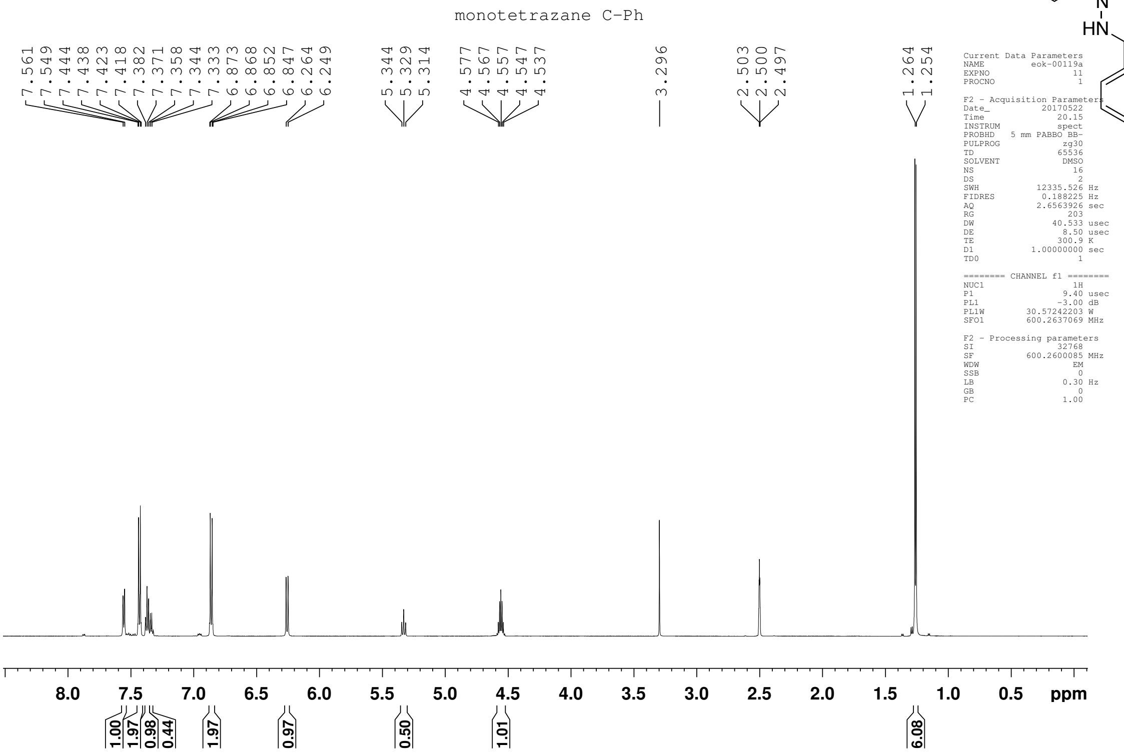

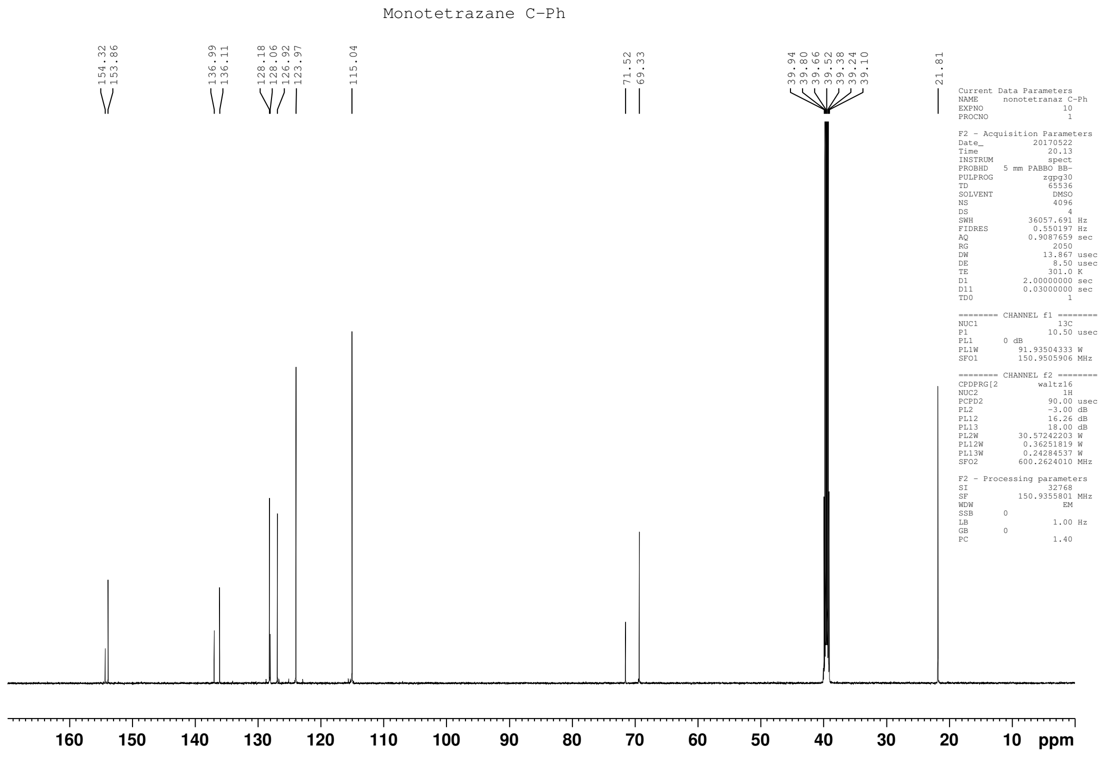

Figure S15. ^{1}H NMR (600 MHz) and $^{13}C\{^{1}H\}$ NMR (151 MHz) of **17** (DMSO-d_6).

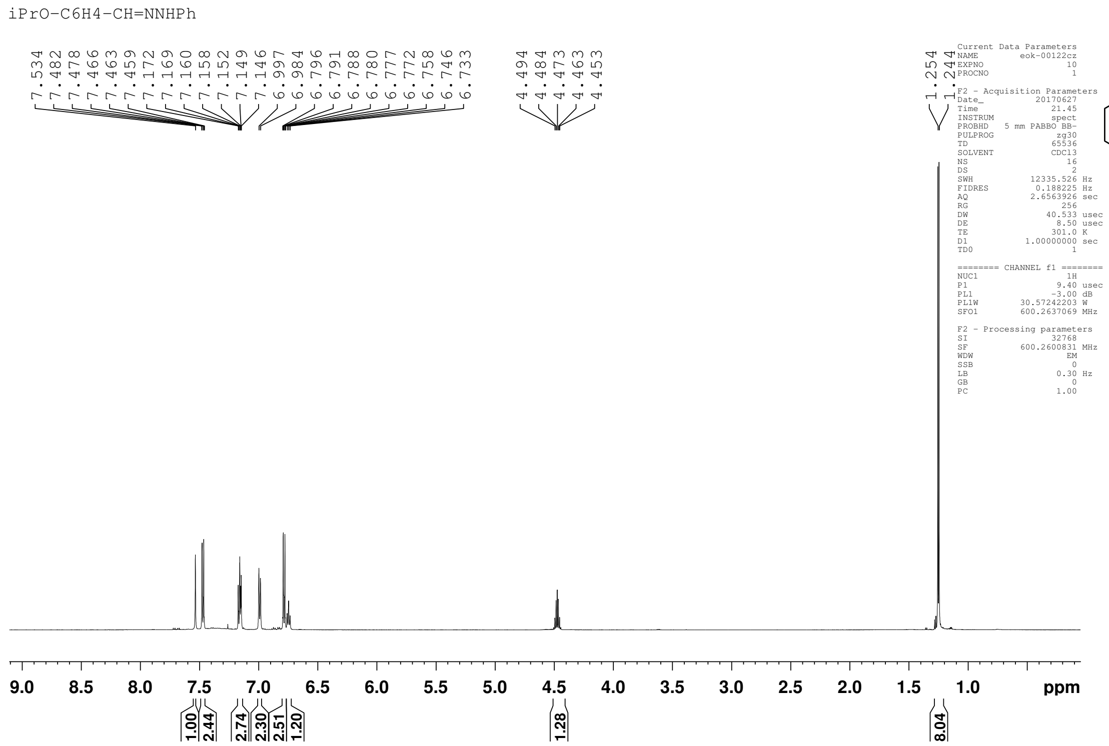

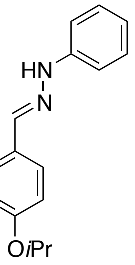

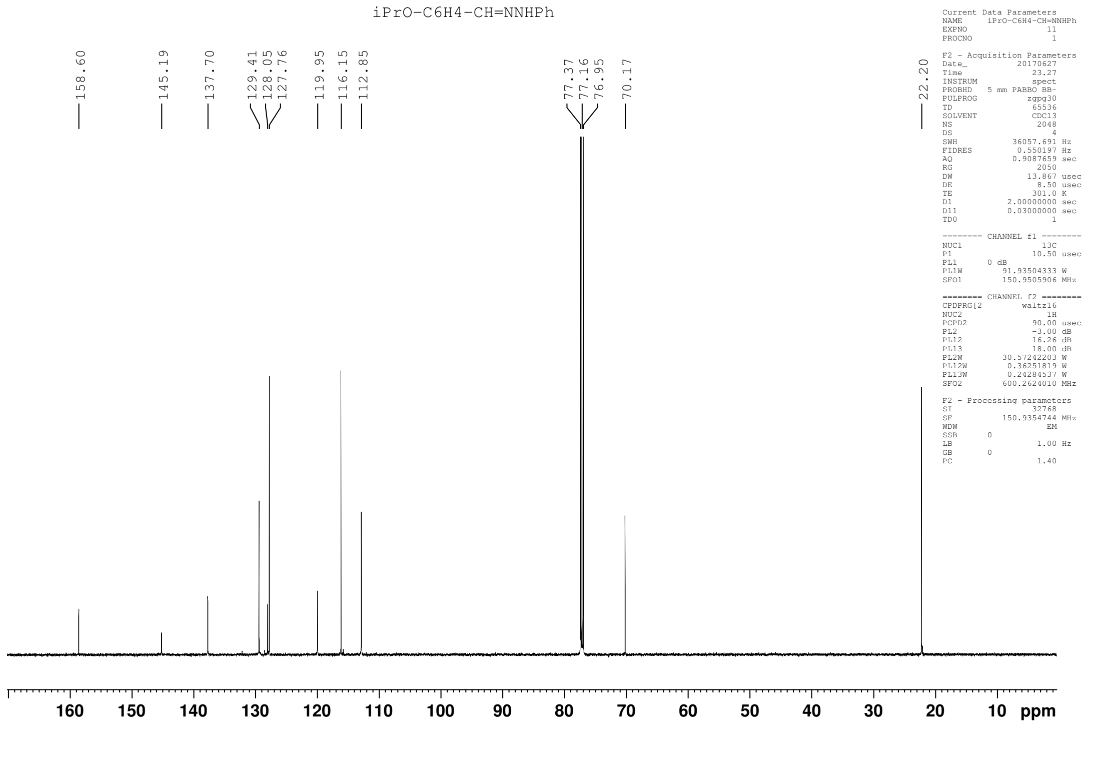

Figure S16. ^{1}H NMR (600 MHz) and ^{13}C{^{1}H} NMR (151 MHz) of **18** ($CDCl_3$).

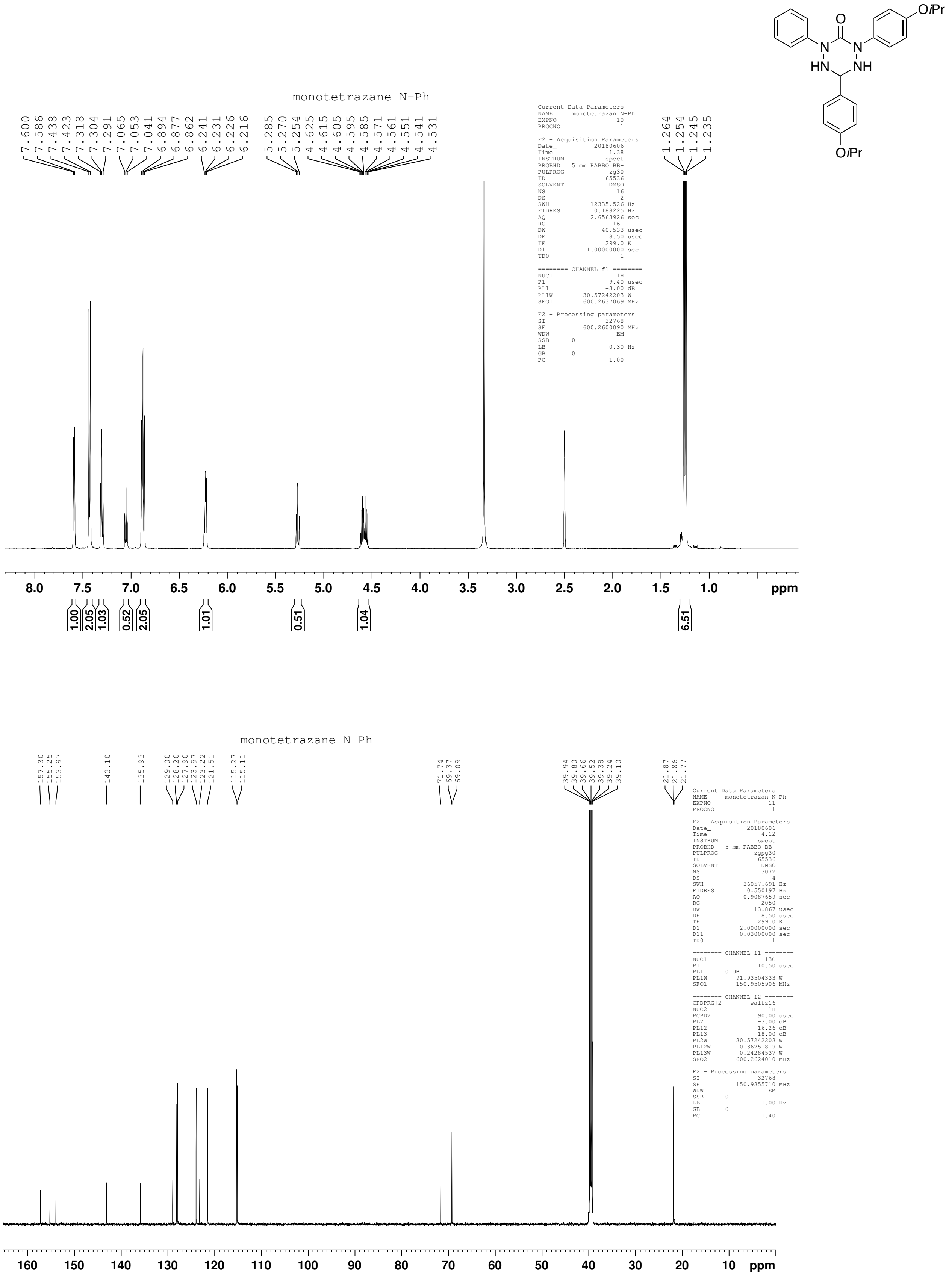

Figure S17. ^{1}H NMR (600 MHz) and ^{13}C{^{1}H} NMR (151 MHz) of **20** (DMSO-d_6).

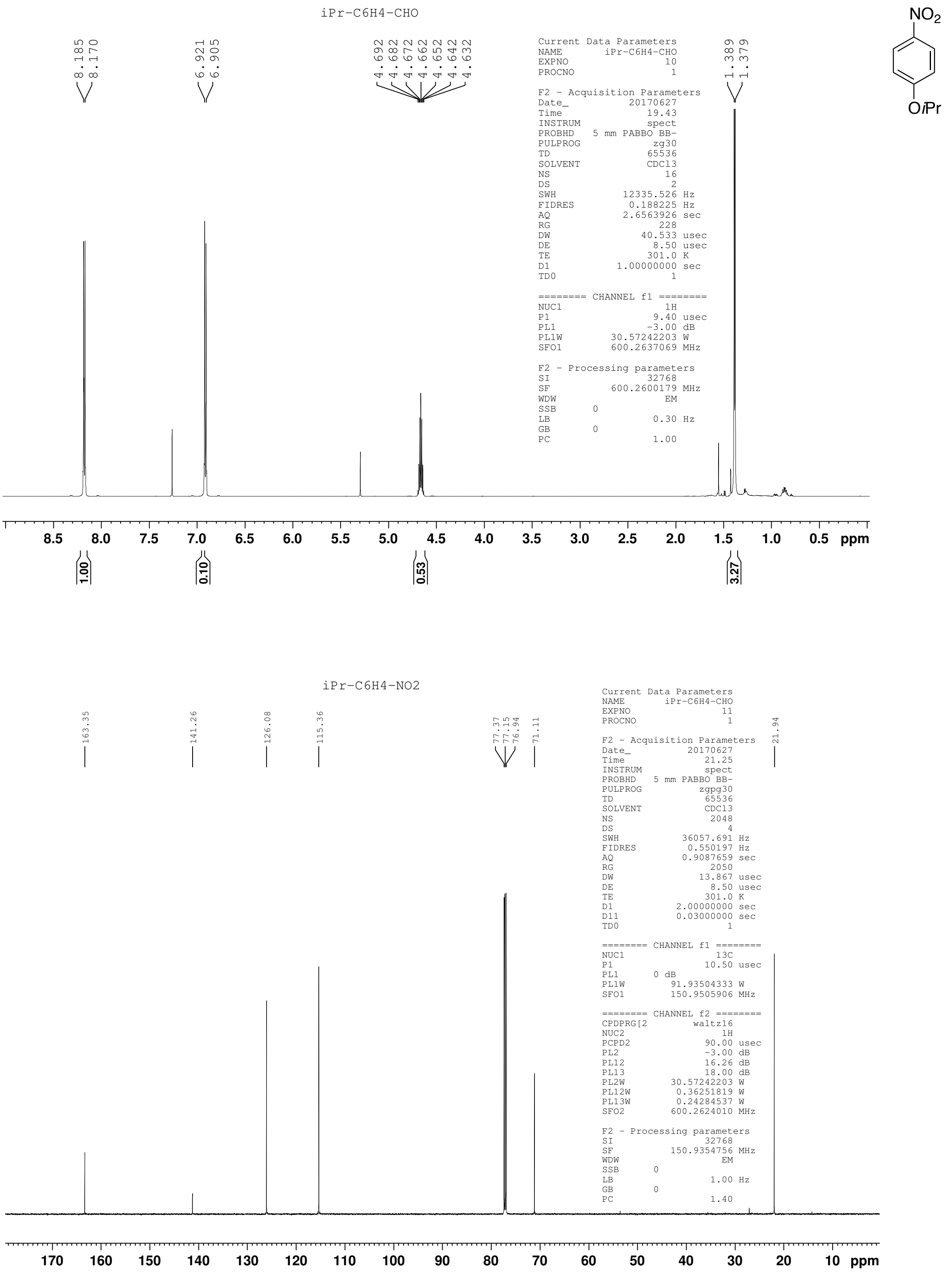

Figure S18. ^{1}H NMR (600 MHz) and $^{13}C\{^{1}H\}$ NMR (151 MHz) of 1-(*i*-propoxy)-4-nitrobenzene ($CDCl_3$).

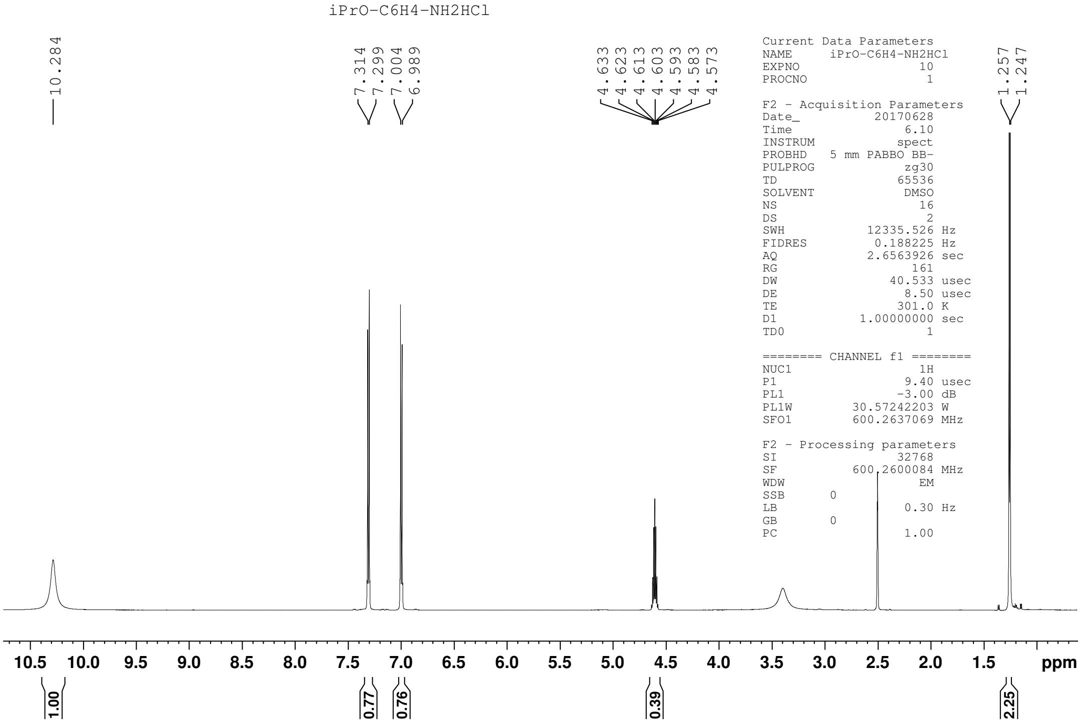

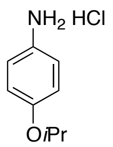

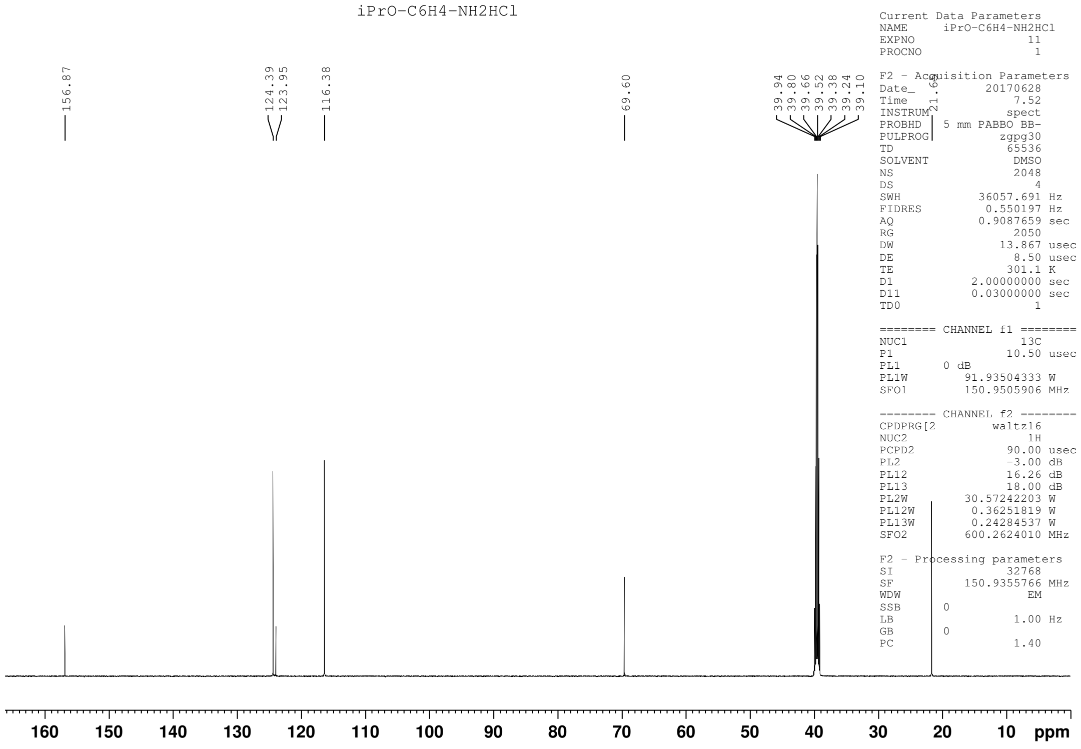

Figure S19. ^{1}H NMR (600 MHz) and ^{13}C{^{1}H} NMR (151 MHz) of 4-(*i*-propoxy)aniline hydrochloride (DMSO-d_6).

3. Additional details of single crystal analysis.

X-Ray data collection and refinement

Good quality single crystals of **1-Np** and **2-C** were selected for X-ray diffraction experiments at 100(2) K. Diffraction data were collected on the Agilent Technologies SuperNova Dual Source diffractometer equipped with an Atlas detector with Cu*K*α radiation (λ = 1.54184 Å) using CrysAlis Pro software.[6] The crystals were positioned at 72 mm from the detector. A total number of 858 and 1656 frames were collected at 1° intervals with a counting time of 1.5−50 s and 1−2 s for **1-Np** and **2-C**, respectively. The lattice parameters were obtained by least-squares fit to the optimized setting angles of the reflections collected by using the CrysAlis CCD software.[6] Data were reduced using the CrysAlis RED program.[6] The multi-scan empirical absorption correction using spherical harmonics, implemented in SCALE3 ABSPACK scaling algorithm, was applied.[6] The structural determination procedure was carried out using the SHELX package.[7] The structures were solved with direct methods and then successive least-square refinement was carried out based on the full-matrix least-squares method on F^2 using the SHELXL program[7] within the Olex2 program.[8] Weighted *R* factors (*wR*) and all goodness-of-fit (GooF) values are based on F^2. Conventional *R* factors are based on *F* with *F* set to zero for negative F^2. The $F_o^2 > 2\sigma(Fo^2)$ criterion was used only for calculating the *R* factors, and it is not relevant to the choice of reflections for the refinement. The *R* factors based on F^2 are about twice larger as those based on *F*. Scattering factors were taken from the International Tables for Crystallography.[9] All H-atoms were positioned geometrically, with C−H equal to 0.93, 0.96 and 0.98 Å for aromatic, methine and methyl H-atoms, respectively, and constrained to ride on their parent atoms with $U_{iso}(H) = xU_{eq}(C)$, where x = 1.2 for the aromatic and methine H-atoms, and x = 1.5 for the methyl H-atoms. The figures for this publication were prepared using Olex2 program.[8] All molecular interactions in crystal were identified using PLATON program.[10] The collected data and refinement parameters are collected in Table S2, while the resulting molecular structures are shown in Figure S20.

CCDC 2270798 and 2270799 contain the supplementary crystallographic data for this paper. These data can be obtained freely via http://www.ccdc.cam.ac.uk/data_request/cif, by e-mailing data_request@ccdc.cam.ac.uk or by contacting directly the Cambridge Crystallographic Data Centre (12 Union Road, Cambridge CB2 1EZ, UK. Fax: +44 1223 336033).

Table S2. Crystallographic data and refinement parameters for **1-Np** and **2-C**.

Identification code	**1-Np**	**2-C**
CCDC	2270799	2270798
Empirical formula	$C_{46}H_{48}N_8O_6$	$C_{26}H_{27}N_4O_3$
Formula weight	808.92	443.51
Temperature/K	100(2)	100(2)
Crystal system	triclinic	Orthorhombic
Space group	*P*-1	*Pbcn*
a/Å	9.7496(7)	25.016(2)
b/Å	12.2597(7)	11.4885(9)
c/Å	18.1423(7)	7.8207(9)
α/°	90.436(4)	90
β/°	101.129(5)	90
γ/°	95.353(5)	90
Volume /Å^3	2117.7(2)	2247.6(4)
Z	2	4
ρ_{calc}g /cm^3	1.269	1.311
μ/mm^{-1}	0.696	0.705
F(000)	856.0	940.0
Crystal size /mm^3	0.39 × 0.15 × 0.12	0.12 × 0.10 × 0.05
Radiation	Cu$K\alpha$ (λ = 1.54184)	Cu$K\alpha$ (λ = 1.54184)
2Θ range for data collection/°	4.966 to 134.156	7.068 to 134.072
Index ranges	$-11 \le h \le 11$, $-14 \le k \le 14$, $-21 \le l \le 13$	$-22 \le h \le 29$, $-12 \le k \le 13$, $-9 \le l \le 9$
Reflections collected	13797	6938
Independent reflections	7572 [R_{int} = 0.0431, R_{sigma} = 0.0812]	2007 [R_{int} = 0.0587, R_{sigma} = 0.0476]
Data/restraints/parameters	7572/0/549	2007/0/154
Goodness-of-fit on F^2	0.993	1.028
Final R indexes [I>=2σ (I)]	R_1 = 0.0570, wR_2 = 0.1250	R_1 = 0.0500, wR_2 = 0.1177
Final R indexes [all data]	R_1 = 0.1085, wR_2 = 0.1479	R_1 = 0.0850, wR_2 = 0.1399
Largest diff. peak/hole / e Å^{-3}	0.32/-0.24	0.24/-0.22

X-Ray data analysis

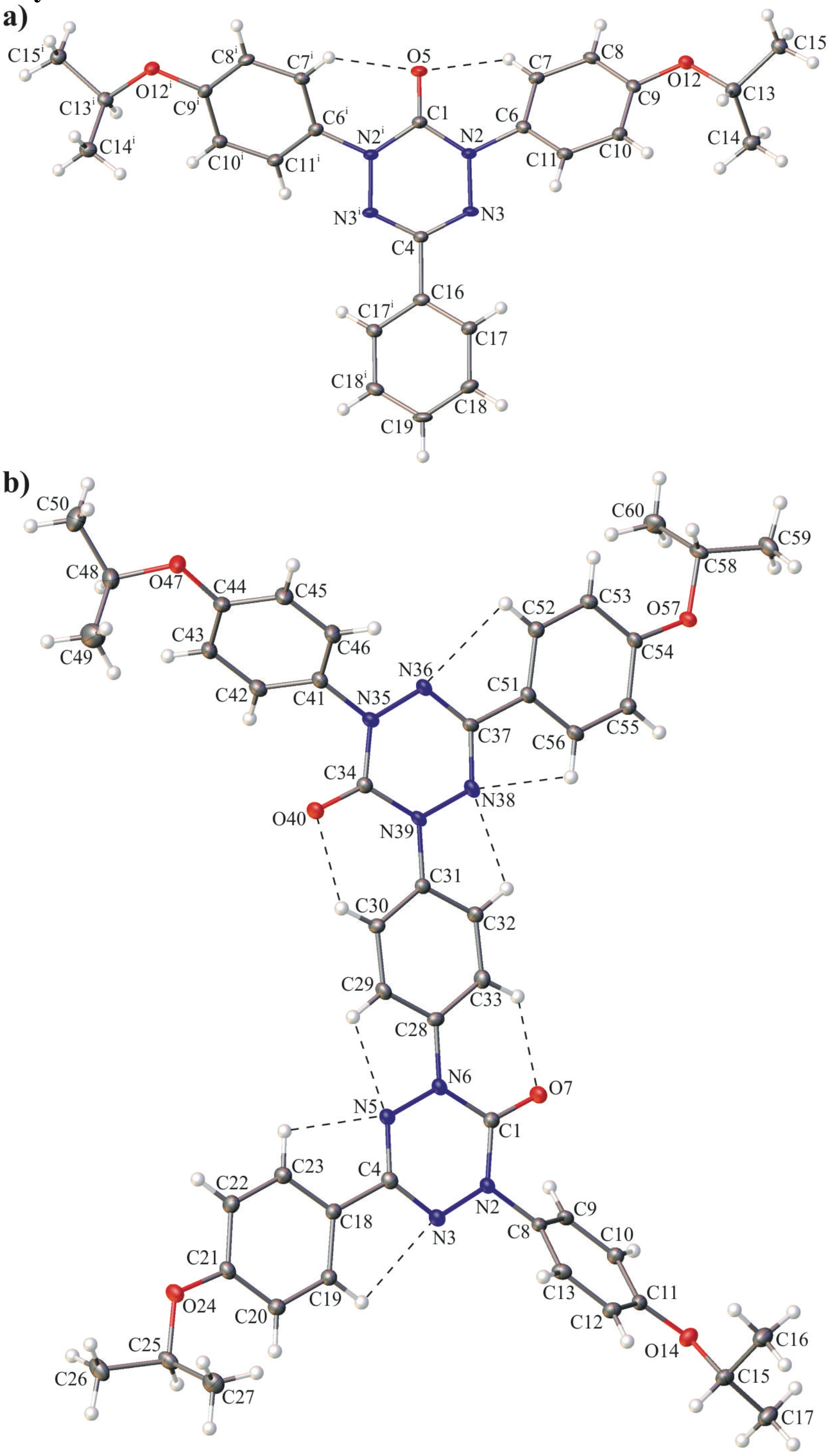

Figure S20. Molecular structure of **2-C** (a) and **1-Np** (b) with crystallographic atom numbering scheme. Displacement ellipsoids are drawn at the 50% probability level. Hydrogen atoms are shown as small spheres of arbitrary radius. The intramolecular C−H···O hydrogen bonds are represented by dashed lines. Symmetry code: (i) $-x + 1$, y, $-z + 3/2$.

Interatomic distances in **1-Np** and **2-C** are listed in Tables S3 and S4, respectively, while close contacts (H bonding, C−H···π, and π – π contacts) are provided in Tables S5–S10.

Table S3. Bond lengths for **1-Np**.

Atom	Atom	Length/Å	Atom	Atom	Length/Å
C(1)	N(2)	1.383(3)	C(31)	N(39)	1.421(3)
C(1)	N(6)	1.391(3)	C(32)	C(33)	1.364(4)
C(1)	O(7)	1.232(3)	C(34)	N(39)	1.394(3)
C(4)	C(18)	1.469(4)	C(34)	N(35)	1.383(3)
C(4)	N(3)	1.330(4)	C(34)	O(40)	1.224(3)
C(4)	N(5)	1.339(3)	C(37)	C(51)	1.485(3)
C(8)	C(9)	1.374(4)	C(37)	N(38)	1.336(3)
C(8)	C(13)	1.393(4)	C(37)	N(36)	1.322(3)
C(8)	N(2)	1.429(3)	C(51)	C(56)	1.393(4)
C(9)	C(10)	1.376(4)	C(51)	C(52)	1.383(4)
C(10)	C(11)	1.399(4)	C(56)	C(55)	1.383(4)
C(11)	C(12)	1.386(4)	C(55)	C(54)	1.395(4)
C(11)	O(14)	1.360(3)	C(54)	C(53)	1.391(4)
C(12)	C(13)	1.386(4)	C(54)	O(57)	1.371(3)
C(15)	C(16)	1.520(4)	C(53)	C(52)	1.394(4)
C(15)	C(17)	1.507(4)	C(58)	C(59)	1.519(4)
C(15)	O(14)	1.449(3)	C(58)	C(60)	1.516(4)
C(18)	C(19)	1.389(4)	C(58)	O(57)	1.449(3)
C(18)	C(23)	1.392(4)	C(41)	C(46)	1.377(4)
C(19)	C(20)	1.391(4)	C(41)	C(42)	1.388(4)
C(20)	C(21)	1.384(4)	C(41)	N(35)	1.438(4)
C(21)	C(22)	1.395(4)	C(46)	C(45)	1.382(4)
C(21)	O(24)	1.364(3)	C(45)	C(44)	1.394(4)
C(22)	C(23)	1.374(4)	C(44)	C(43)	1.375(4)
C(25)	C(26)	1.516(4)	C(44)	O(47)	1.371(4)
C(25)	C(27)	1.510(4)	C(43)	C(42)	1.388(4)
C(25)	O(24)	1.443(3)	C(48)	C(50)	1.515(5)
C(28)	C(29)	1.397(4)	C(48)	C(49)	1.504(5)
C(28)	C(33)	1.407(4)	C(48)	O(47)	1.440(3)
C(28)	N(6)	1.422(3)	N(2)	N(3)	1.378(3)
C(29)	C(30)	1.378(4)	N(5)	N(6)	1.364(3)
C(30)	C(31)	1.399(3)	N(39)	N(38)	1.367(3)
C(31)	C(32)	1.397(4)	N(36)	N(35)	1.382(3)

Table S4. Bond lengths for **2-C**.

Atom	Atom	Length/Å	Atom	Atom	Length/Å
C(1)	N(2)	1.388(3)	C(9)	O(12)	1.369(3)
C(1)	N(2)^{i}	1.388(3)	C(10)	C(11)	1.385(3)
C(1)	O(5)	1.212(4)	C(13)	C(14)	1.522(3)
C(4)	C(16)	1.493(4)	C(13)	C(15)	1.508(3)
C(4)	N(3)	1.328(2)	C(13)	O(12)	1.451(3)
C(4)	N(3)^{i}	1.328(2)	C(16)	C(17)	1.393(3)
C(6)	C(7)	1.394(3)	C(16)	C(17)^{i}	1.393(3)
C(6)	C(11)	1.383(3)	C(17)	C(18)	1.386(3)
C(6)	N(2)	1.438(3)	C(18)	C(19)	1.386(3)
C(7)	C(8)	1.385(3)	C(19)	C(18)^{i}	1.386(3)
C(8)	C(9)	1.386(3)	N(2)	N(3)	1.375(2)
C(9)	C(10)	1.388(3)			

Symmetry code: (i) $-x + 1, y, -z + 3/2$.

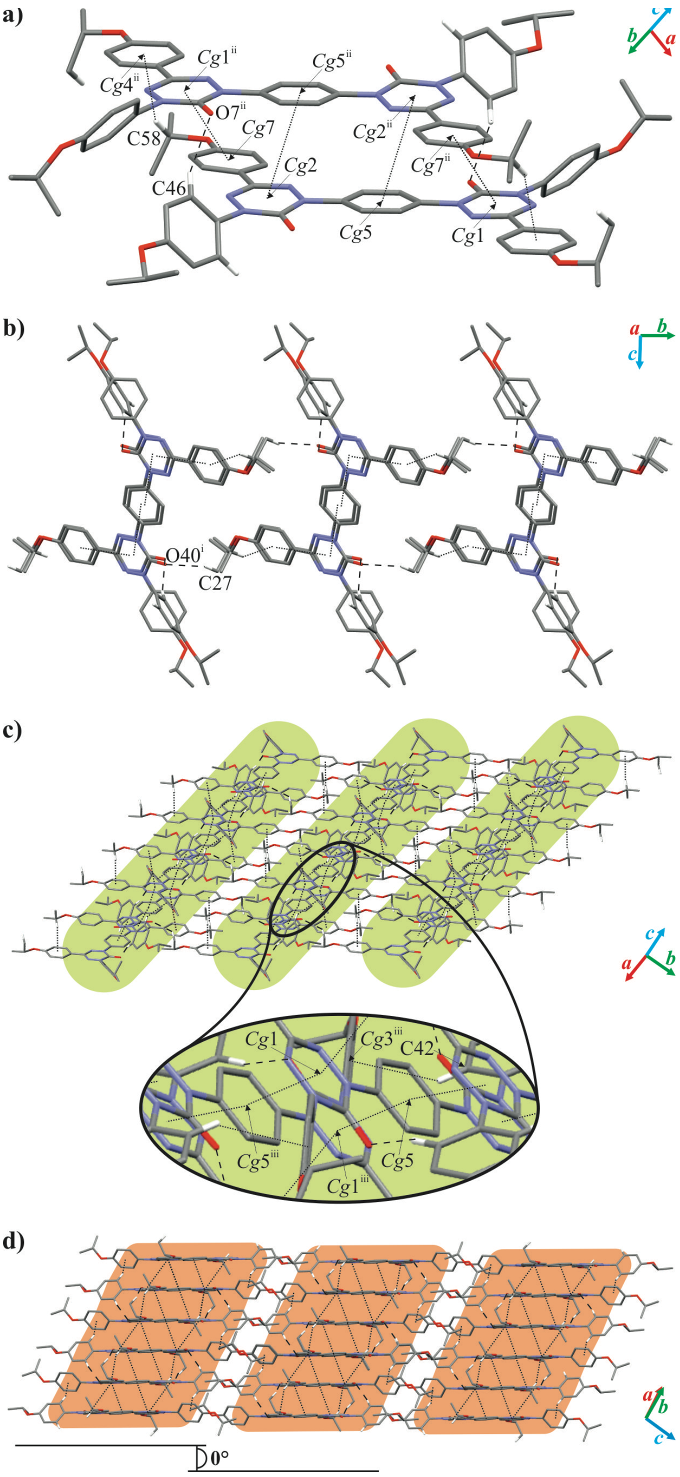

Figure S21. Supramolecular architecture of the crystal of **1-Np**, where: (a) dimer of molecules; (b) complex chain of H-bonded dimers of molecules running along [1-10] direction; (c) 2D-sheet built from stacks of molecules, viewed from the side (single stacks are highlighted in green); (c) general view on arrangement of molecules in the crystal structure (single 2D-sheets are highlighted in beige). The C−H···O hydrogen bonds are represented by dashed lines, while the π-π and C−H···π contacts by dotted lines. The H-atoms not involved in the intermolecular interactions were omitted for clarity. (i) $-x + 1, -y, -z + 1$; (ii) $-x, -y + 1, -z + 1$; (iii) $-x + 1, -y +1, -z + 1$.

Diradical **1-Np** forms discrete dimers of antiparallel oriented molecules connected through weak C−H···O hydrogen bonds involving the H-atoms of the N-bonded *i*-propoxyphenyl substituents and the carbonyl oxygen atoms (Figure S21a, Table S5). The dimers are additionally stabilized by a network of weak π–π and C−H···π contacts (Figure S21b, Tables S6 and S7). Neighboring dimers in the crystal network are held together by weak C−H···O hydrogen bonds between the H-atoms of the *i*-propoxy groups attached to the C-bonded phenyl ring and the carbonyl O-atom forming complex infinite chains running along the [1–10] direction (Figure S21b, Table S5). The phenyl and verdazyl rings in adjacent chains are involved in a network of weak π–π contacts, what results in formation of 2D-sheet spreading along the (001) plane (Figure S21c, Table S7). Adjacent molecules in the sheets are also organized in stacks (Figure S21c) with numerous π–π interactions (Table S7). Interacting rings within the stacks in the crystal of **1-Np** are slightly twisted relative each other. The twist angle ranges from 5.39(12)° and 8.35(12)° and the distance between their geometric centers of gravity, Cg, varies from 3.704(2) Å to 4.090(2) Å (Table S5). The 2D-sheets are additionally stabilized by weak C−H···π contacts between neighboring molecules in adjacent H-bonded complex chains of molecules (Figure S21c, Table S5). A close look at Figure S21d reveals that mean-planes defined by the adjacent molecules of the diradicals in the neighboring sheets are parallel to each other.

Table S5. Hydrogen-bond geometry in the crystal of **1-Np**.

D−H···A	d(D−H) (Å)	d(H···A) (Å)	d(D···A) (Å)	<D−H···A (°)
C19−H19···N3*	0.93	2.49	2.806(3)	100
C23−H23···N5*	0.93	2.43	2.760(3)	101
C27−H27C···O40^{i}	0.96	2.57	3.262(4)	129
C29−H29···N5*	0.93	2.28	2.646(4)	103
C30−H30···O40*	0.93	2.13	2.753(3)	123
C32−H32···N38	0.93	2.28	2.650(4)	103
C33−H33···O7	0.93	2.11	2.742(3)	124
C46−H46···O7^{ii}	0.93	2.40	3.278(4)	157
C52−H52···N36*	0.93	2.53	2.838(3)	100
C56−H56···N38*	0.93	2.40	2.735(3)	101

Symmetry codes: (i) −*x* + 1, −*y*, −*z* + 1; (ii) −*x*, −*y* + 1, −*z* + 1; (*) intramolecular interaction.

Table S6. The geometry of the C−H···π contacts in the crystal of **1-Np**.

D−H	*Cg*I	d(X··· *Cg*I) (Å)	<Y−X···*Cg*I (°)
C42−H42	3^{iii}	3.543(3)	138
C58−H58	4^{ii}	3.702(3)	149

*Cg*3 and *Cg*4 denote geometric centres of gravity of the aromatic rings delineated by the C8−C13 and C18−C23 atoms, respectively (Fig. 1). Symmetry codes: (ii) −*x*, −*y* + 1, −*z* + 1; (iii) −*x* + 1, −*y* + 1, −*z* + 1.

Table S7. The geometry of π–π contacts in the crystal of **1-Np**.

*Cg*I	*Cg*J	*Cg*I···*Cg*J (Å)	Dihedral angle (°)	Interplanar distance (Å)	Offset (Å)
1	5^{iii}	3.704(2)	5.39(12)	3.357(2)	1.565(2)
1	7^{ii}	4.053(2)	6.69(12)	-3.137(2)	2.566(2)
2	5^{ii}	4.090(2)	8.35(12)	-3.507(2)	2.105(2)
5	1^{iii}	3.704(2)	5.39(12)	3.197(2)	1.871(2)
5	2^{ii}	4.090(2)	8.35(12)	-3.191(2)	2.558(2)
7	1^{ii}	4.053(2)	6.69(12)	-3.408(2)	2.194(2)

Symmetry codes: (ii) −*x*, −*y* + 1, −*z* + 1; (iii) −*x* + 1, −*y* + 1, −*z* + 1.
*Cg*1, *Cg*2, *Cg*5 and *Cg*7 denote geometric centres of gravity of the aromatic rings delineated by the C1/N2−N3/C4/N5−N6, C34/N35−N36/C37/N38−N39, C28−C33 and C51−C56 atoms, respectively (Fig. 1).
*Cg*I···*Cg*J is the distance between ring centroids.
The dihedral angle is the angle between the mean planes of *Cg*I and *Cg*J.
The interplanar distance is the perpendicular distance from *Cg*I to ring J.
The offset is the perpendicular distance from ring I to ring J.

In the crystal of **2-C**, neighboring molecules are oriented '*head-to-tail*' forming an infinite chain running along the [010] direction with a weak C−H···O hydrogen bond (Figure S22a, Table S8). The chains are oriented antiparallel and arranged in stacks spreading along the [001] direction, which results in a 2D-sheet parallel to the (100) plane (Figures S22b and S22c). The verdazyl and C-bonded phenyl groups of neighboring molecules between the stacks are involved in a network of weak π–π contacts (Figure S22b, Tables S9 and S10). The planes of the interacting aromatic rings within the stack are inclined to each other by the angle of 18.41(10)° and the distance between their geometrical centers of gravity, Cg, is 3.938(2) Å (Table S10). The entire crystal structure is stabilized by weak C−H···π contacts engaging the H-atoms of the *i*-propoxy groups attached to the N-bonded phenyl rings and these phenyl rings of neighboring molecules in adjacent 2D-sheets (Figure S22c, Table S9). A general view on the crystal structure of **2-CP** along the *b*-direction reveals the characteristic '*herring-bone*' supramolecular architecture, in which the mean-planes of molecules in adjacent sheets are inclined to each other by the angle of *ca*. 144.5° (Figure S22c).

Table S8. Hydrogen-bond geometry in the crystal of **2-C**.

D−H···A	d(D−H) (Å)	d(H···A) (Å)	d(D···A) (Å)	<D−H···A (°)
C7−H7···O5*	0.93	2.36	2.833(3)	111
C19−H19···$O5^{ii}$	0.93	2.34	3.270(4)	180

Symmetry codes: (ii) *x*, *y* + 1, *z*; (*) intramolecular interaction.

Table S9. The geometry of the C−H···π contacts in the crystal of **2-C**.

D−H	*Cg*I	d(X··· *Cg*I) (Å)	<Y−X···*Cg*I (°)
C14−H14B	2^{iii}	3.704(3)	154

*Cg*2 denotes geometric center of gravity of the aromatic ring delineated by the C6−C11 atoms (Fig. 1). Symmetry code: (iii) −*x* + 3/2, −*y* + 1/2, *z* + 1/2.

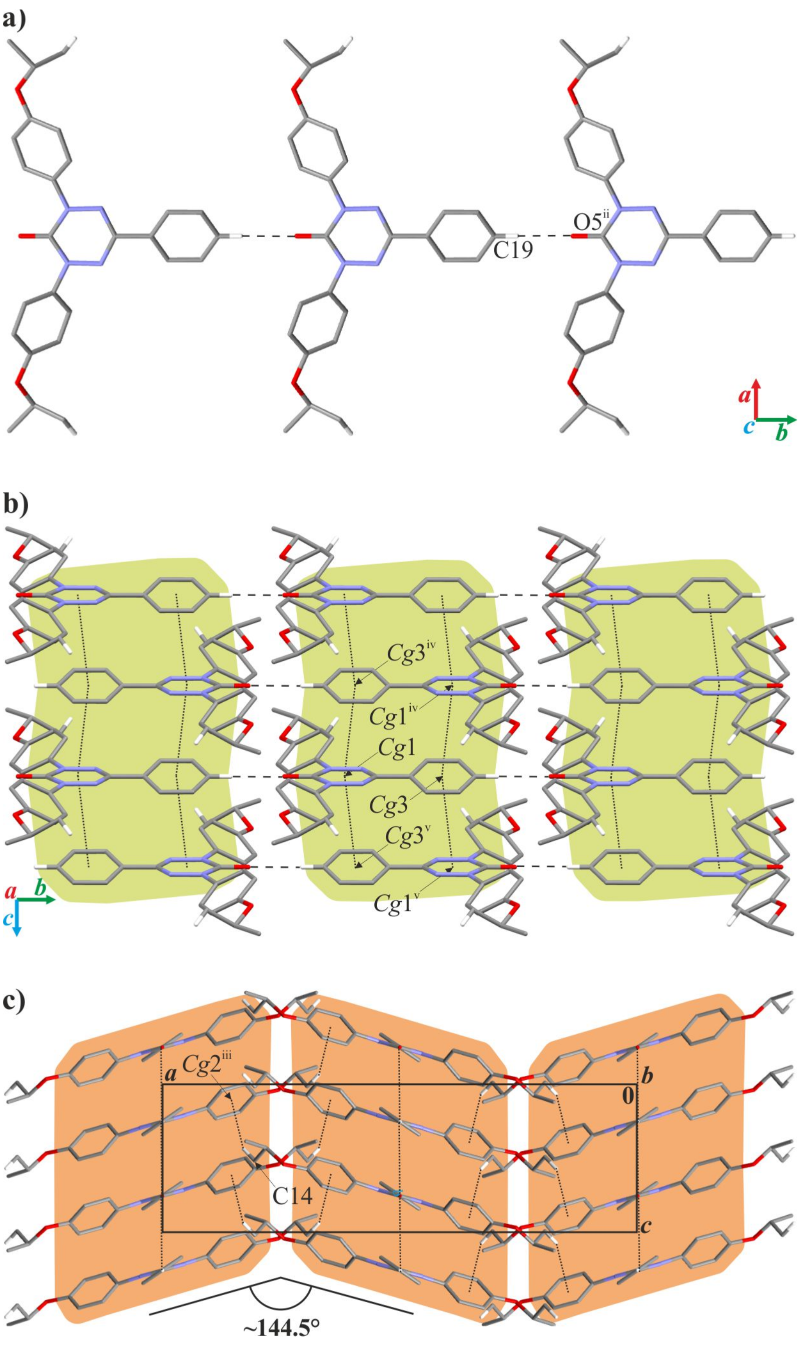

Figure S22. Supramolecular architecture of the crystal of **2-C**, where: (a) H-bonded chain of molecules running along [010] direction; (b) 2D-sheet built from H-bonded stacks of molecules, viewed from the side (single stacks are highlighted in green); (c) general view on characteristic '*herring-bone*' arrangement of molecules, viewed along *b*-direction (single 2D-sheets are highlighted in beige). The C−H···O hydrogen bonds are represented by dashed lines, while the π-π and C−H···π contacts by dotted lines. The H-atoms not involved in the intermolecular interactions were omitted for clarity. Symmetry codes: (ii) *x*, *y* + 1, *z*; (iii) −*x* + 3/2, −*y* + 1/2, *z* + 1/2; (iv) −*x* + 1, −*y*, −*z* + 2; (v) −*x* + 2, −*y* + 1, −*z* + 1.

Table S10. The geometry of π–π contacts in the crystal of **2-C**.

*Cg*I	*Cg*J	*Cg*I···*Cg*J (Å)	Dihedral angle (°)	Interplanar distance (Å)	Offset (Å)
1	3^{iv}	3.938(2)	18.41(10)	-3.237(2)	2.243(2)
1	3^{v}	3.938(2)	18.41(10)	3.237(2)	2.243(2)
3	1^{iv}	3.938(2)	18.41(10)	-3.764(2)	1.158(2)
3	1^{v}	3.938(2)	18.41(10)	3.764(2)	1.158(2)

Symmetry codes: (i) $-x + 1, y, -z + 3/2$; (iv) $-x + 1, -y, -z + 2$; (v) $-x + 2, -y + 1, -z + 1$.
*Cg*1 and *Cg*3 denote geometric centres of gravity of the aromatic rings delineated by the C1/N1−N2/C4/N3^{i}−N2^{i} and C16−C19/C18^{i}−C17^{i} atoms, respectively (Fig. 1).
*Cg*I···*Cg*J is the distance between ring centroids.
The dihedral angle is the angle between the mean planes of *Cg*I and *Cg*J.
The interplanar distance is the perpendicular distance from *Cg*I to ring J.
The offset is the perpendicular distance from ring I to ring J.

4. UV-vis spectroscopy

Electronic absorption spectra for radicals **1** and **2** were recorded in spectroscopic grade CH_2Cl_2 at concentrations in a range 1.5–10×10^{-5} M and fitted to the Beer–Lambert law. Results are shown in Figures S23–S28.

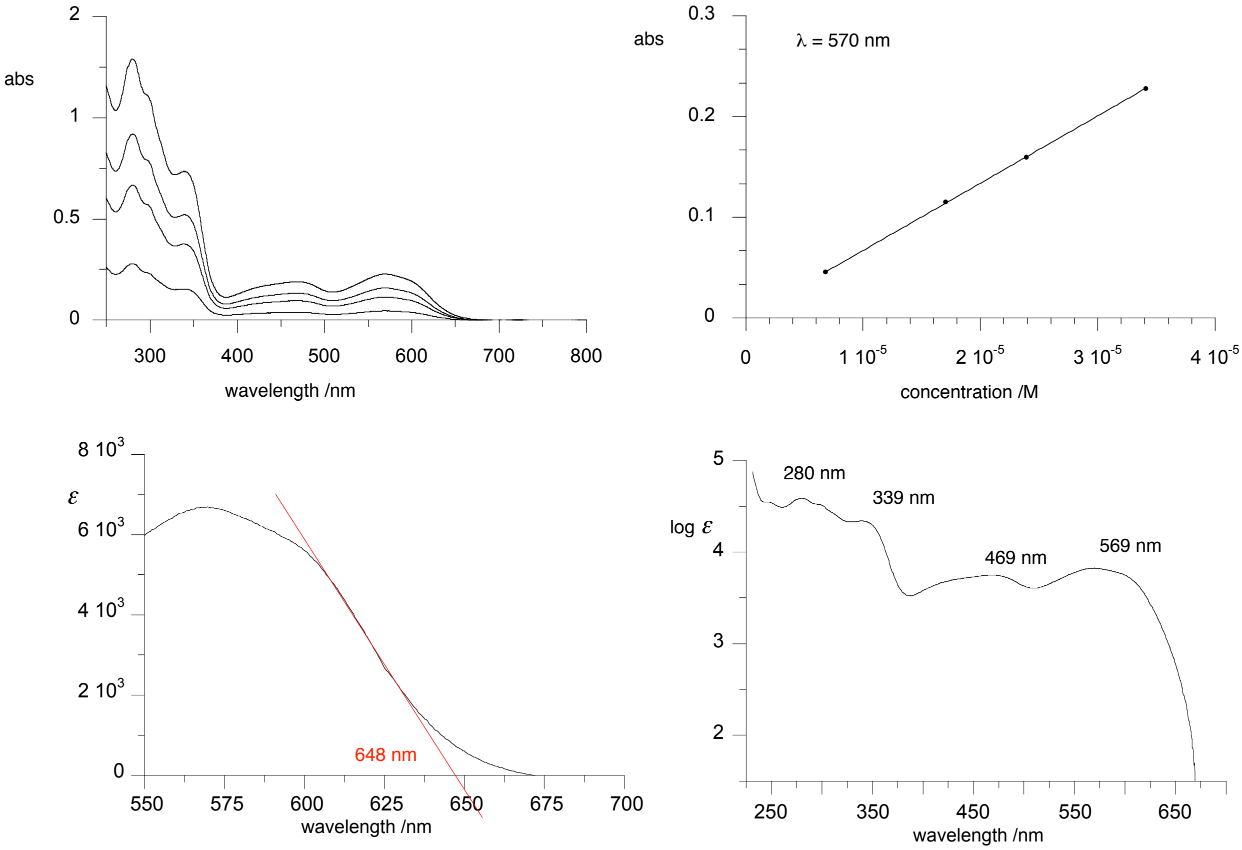

Figure S23. Clockwise: electronic absorption spectra for **1-Cp** in CH_2Cl_2 for four concentrations and determination of molar extinction coefficient ε at λ = 569 nm (best fit function: ε = 6691(14)×conc, r^2 = 0.99993), molar excitation *log* (ε) plot, and onset of absorption.

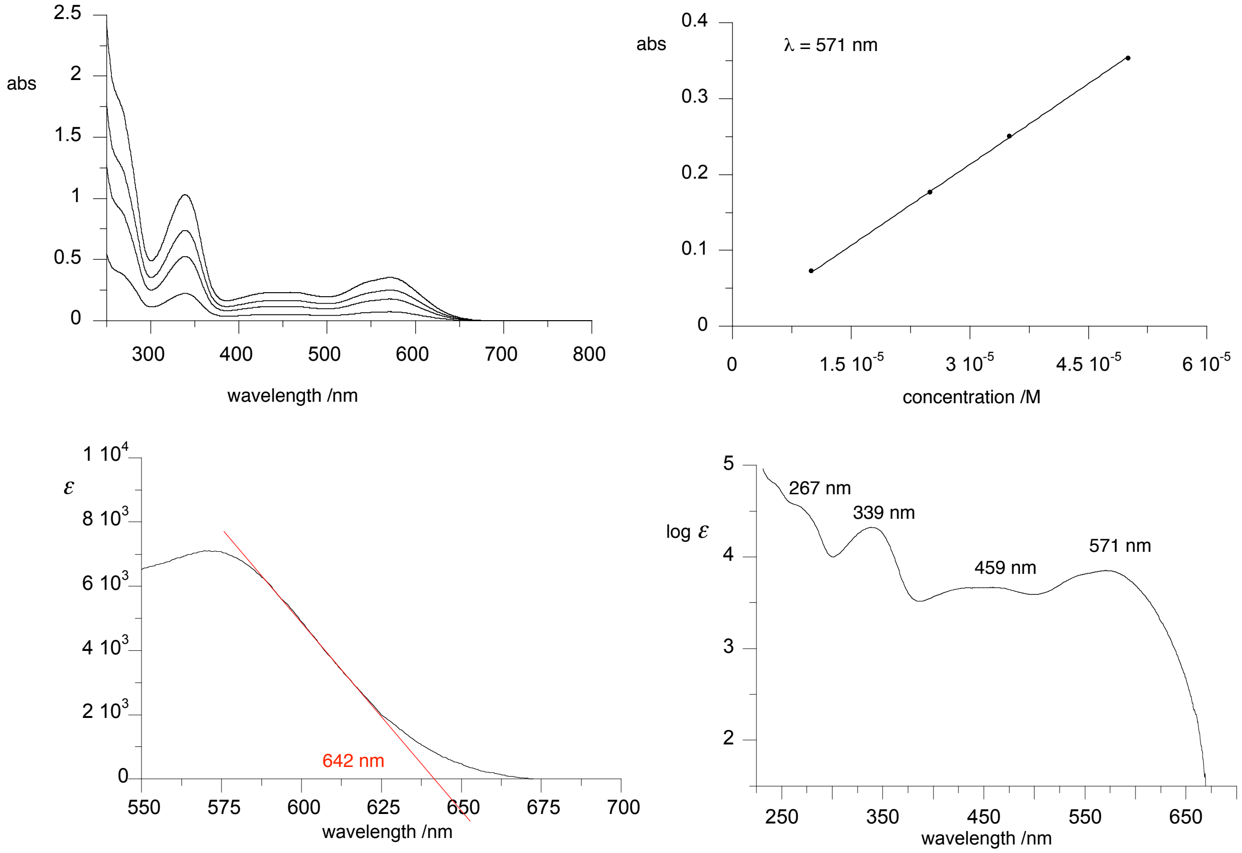

Figure S24. Clockwise: electronic absorption spectra for **1-Cm** in CH_2Cl_2 for four concentrations and determination of molar extinction coefficient ε at λ = 571 nm (best fit function: ε = 7103(33)×conc, r^2 = 0.9997), molar excitation *log* (ε) plot, , and onset of absorption.

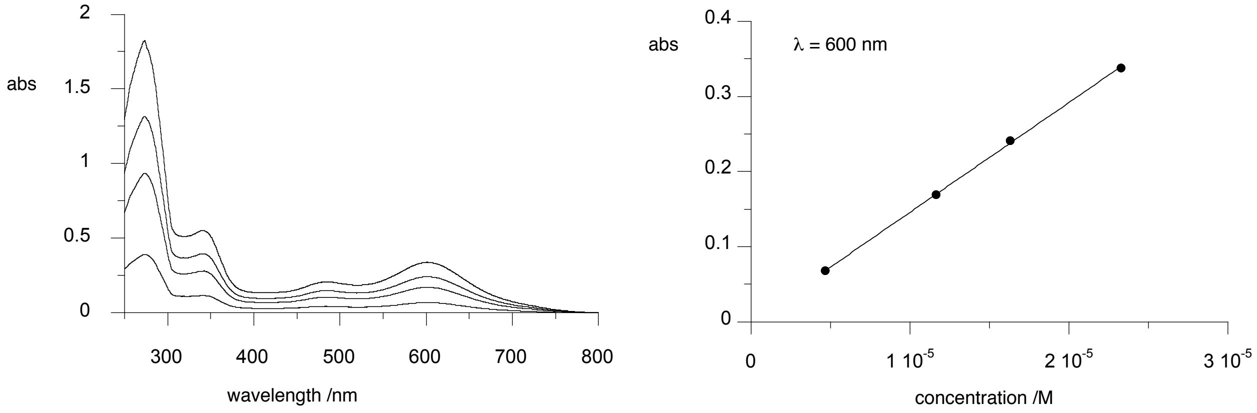

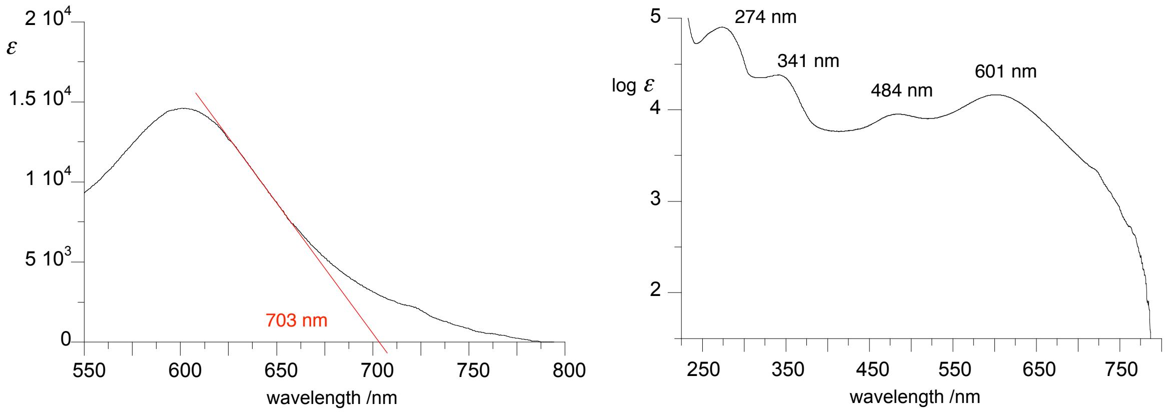

Figure S25. Clockwise: electronic absorption spectra for **1-Np** in CH_2Cl_2 for four concentrations and determination of molar extinction coefficient ε at λ = 601 nm (best fit function: ε = 14580(79)×conc, r^2 = 0.9995), molar excitation *log* (ε) plot, and onset of absorption.

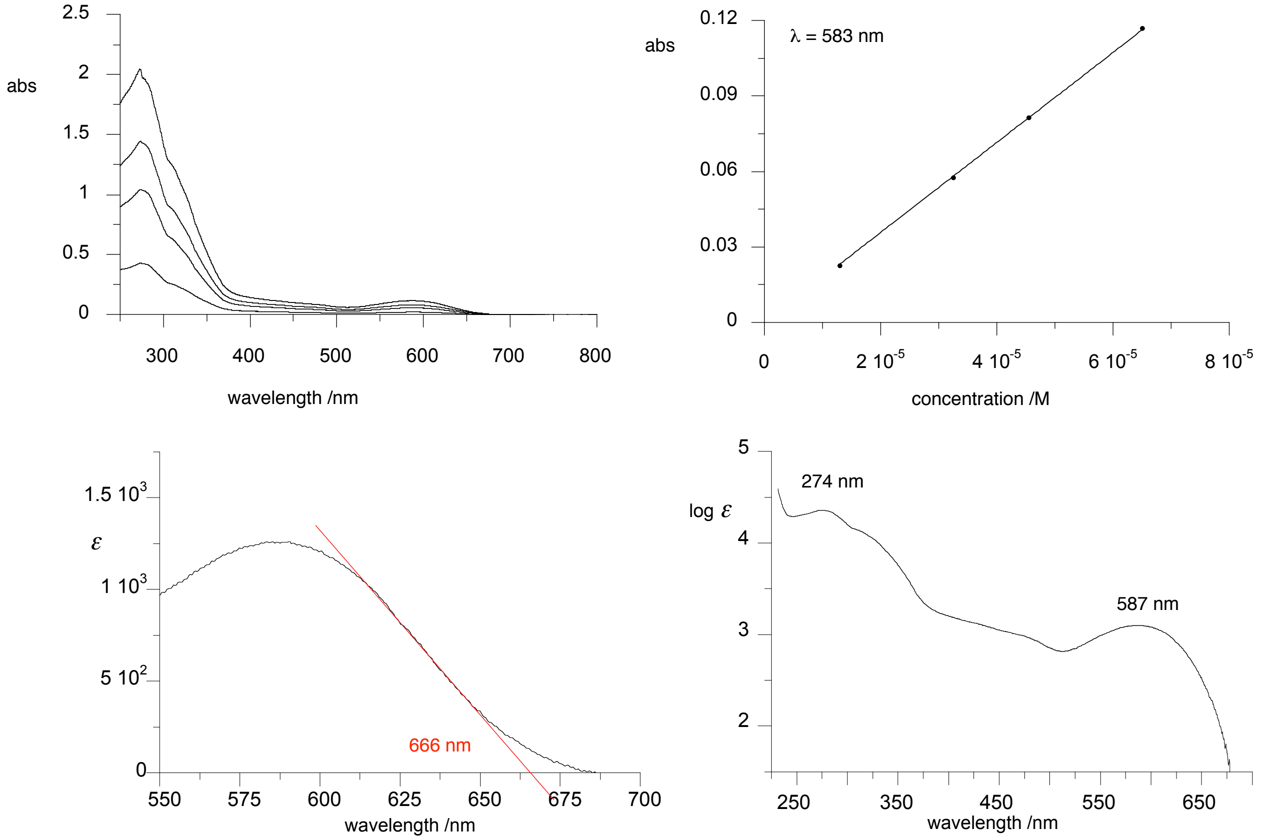

Figure S26. Clockwise: electronic absorption spectra for **1-Nm** in CH_2Cl_2 for four concentrations and determination of molar extinction coefficient ε at λ = 587 nm (best fit function: ε = 1787(8)×conc, r^2 = 0.9997), molar excitation *log* (ε) plot, and onset of absorption.

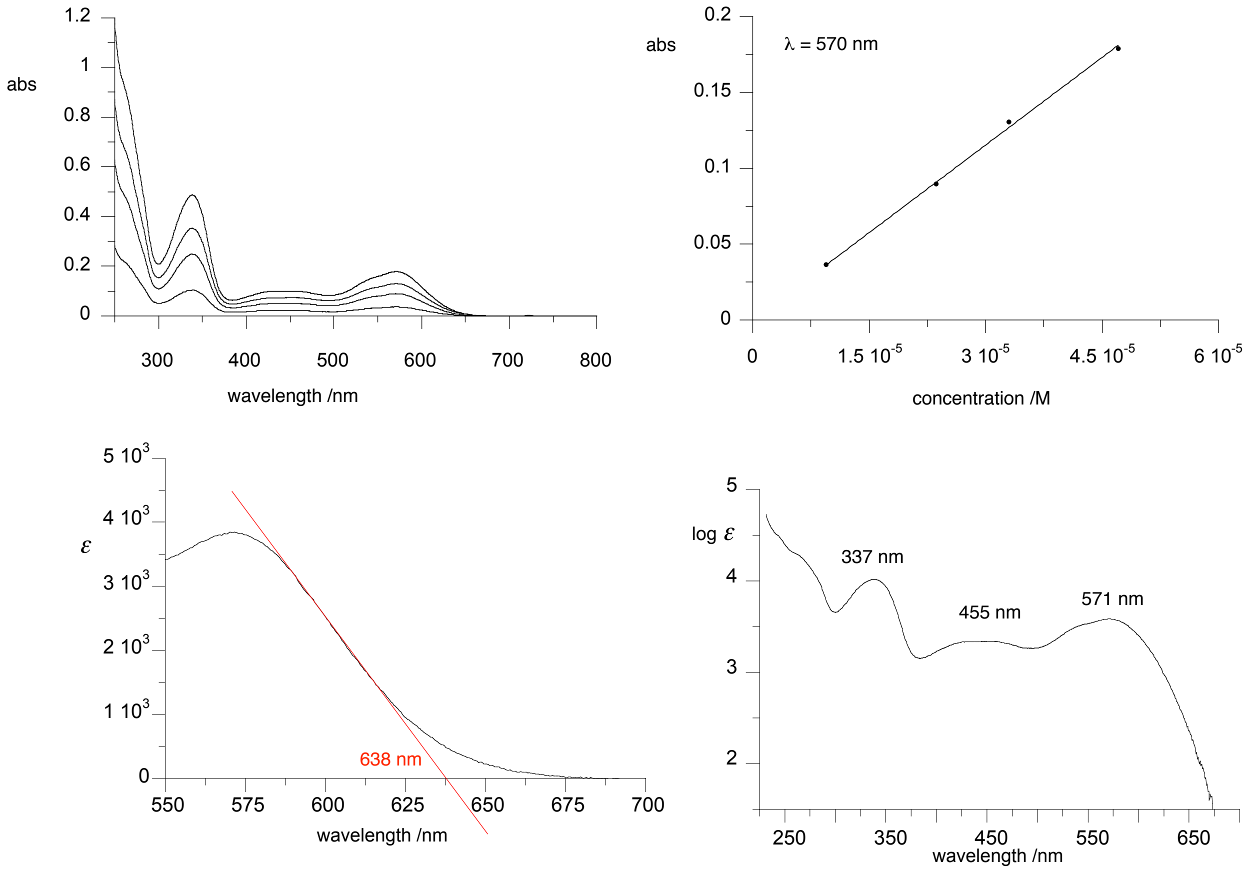

Figure S27. Clockwise: electronic absorption spectra for **2-C** in CH_2Cl_2 for four concentrations and determination of molar extinction coefficient ε at λ = 571 nm (best fit function: ε = 3846(42)×conc, r^2 = 0.998), molar excitation *log* (ε) plot, and onset of absorption.

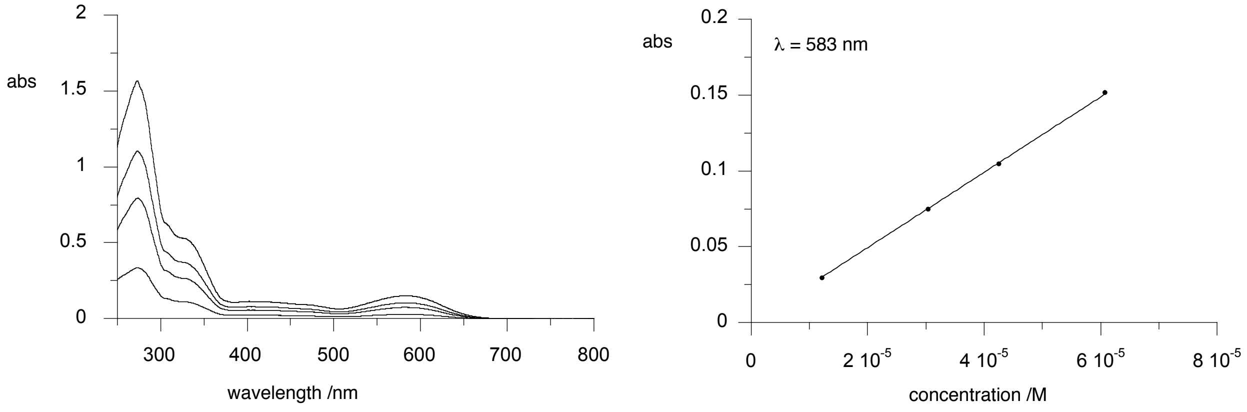

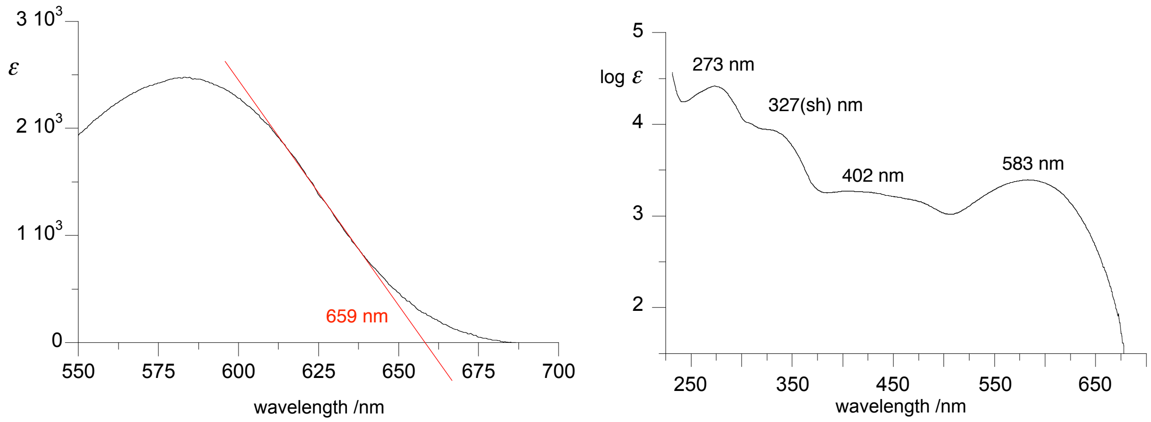

Figure S28. Clockwise: electronic absorption spectra for **2-N** in CH_2Cl_2 for four concentrations and determination of molar extinction coefficient ε at λ = 583 nm (best fit function: ε = 2478(10)×conc, r^2 = 0.9998), molar excitation *log* (ε) plot, and onset of absorption.

5. Electrochemical results

The electrochemical characterization of radicals **1** and **2** was conducted using Metrohm Autolab PGSTAT128N potentiostat/galvanostat instrument. Radicals **1** and **2** were dissolved in dry, spectroscopic grade CH_2Cl_2 (concentration 0.5 mM) in the presence of $[n\text{-}Bu_4N]^+[PF_6]^-$ as an electrolyte (concentration 50 mM) and the resulting solution was degassed by purging with Ar gas for 20 minutes. A three-electrode electrochemical cell was used with glassy carbon disk as the working electrode (ϕ 2 mm, alumina polished), Pt wire as the counter electrode and Ag/AgCl wire as the pseudoreference electrode. All samples were measured without internal reference followed by measurements with added ferrocene as the internal reference with a scan rate of 50 mV s^{-1} (CV) or 5 mV s^{-1} (DPV) at *ca.* 20 °C. The oxidation potential for the Fc/Fc^+ couple (0.46 V vs SCE)[11] was set at 0.0 V. Cyclic voltammetry (CV) measurements were started from 0.0 V in the oxidative direction.

Cyclic voltammetry (CV) and differential pulse voltammetry (DPV) plots are shown in Figures S29–S34, and numerical result are shown in Table 3 in the main text.

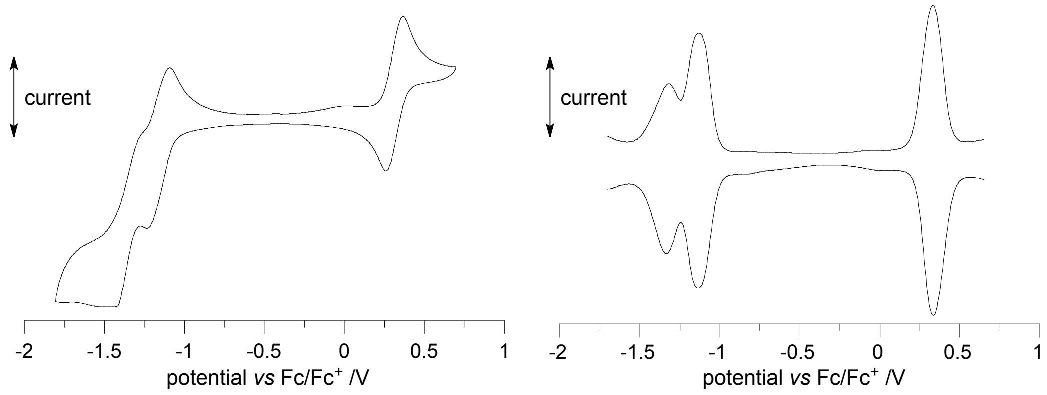

Figure S29. Cyclic and differential pulse voltammograms for **1-Cp**.

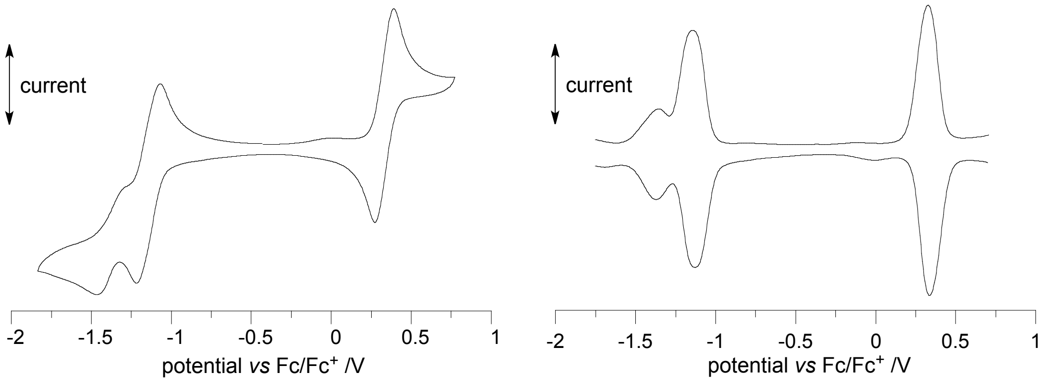

Figure S30. Cyclic and differential pulse voltammograms for **1-Cm**.

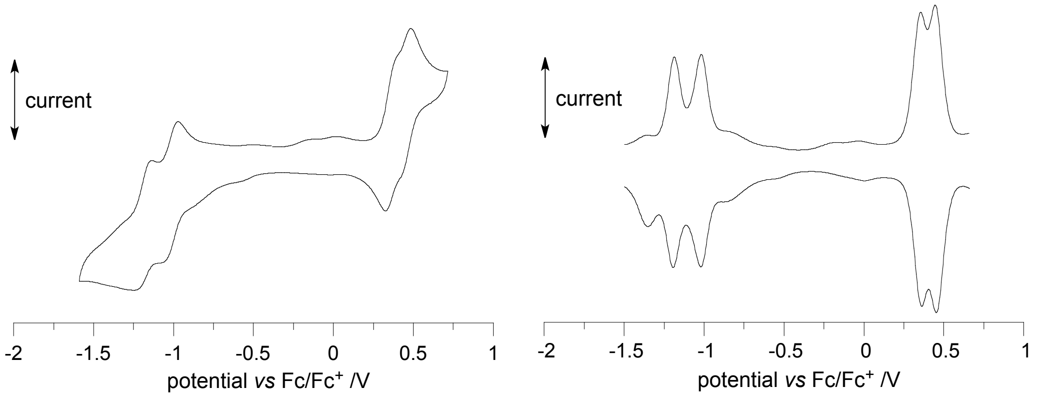

Figure S31. Cyclic and differential pulse voltammograms for **1-Np**.

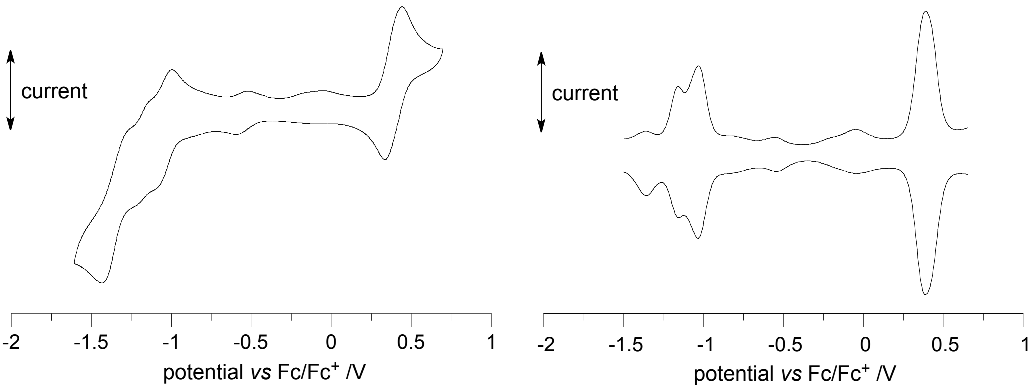

Figure S32. Cyclic and differential pulse voltammograms for **1-Nm**.

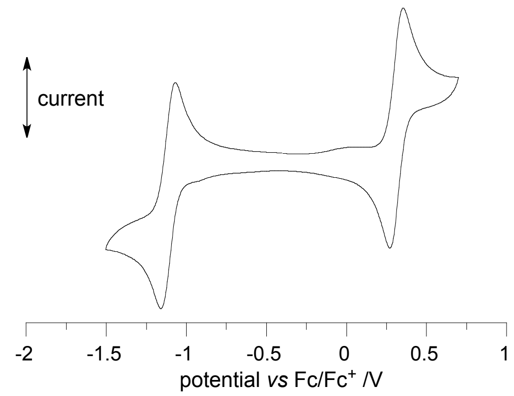

Figure S33. Cyclic voltammogram for **2-C**.

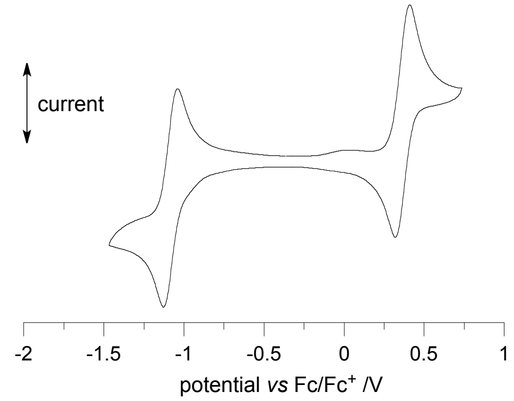

Figure S34. Cyclic voltammogram for **2-N**.

6. EPR spectroscopy

a) liquid solution spectroscopy

EPR spectra for monoradicals **2-C** and **2-N** were recorded on an X-band EMX-Nano EPR spectrometer at room temperature on diluted and degassed solutions in benzene. The microwave power was in a range of 3-12 mW (established with the Power Sweep program below the saturation of the signal) with a modulation frequency of 100 kHz, modulation amplitude of 0.5 G_{pp} and spectral width of 100 G. Accurate *g*-values were obtained using TEMPO as EMX-Nano internal standard. Simulations of the spectra were performed with the EMX-Nano software using DFT results (*vide infra*) as the starting point including all nitrogen atoms. The resulting *hfcc* values were perturbed several times until a global minimum for the fit was achieved. Experimental and simulated spectra are shown in Figures S35 and S36 and resulting *hfcc* are listed in Table S11.

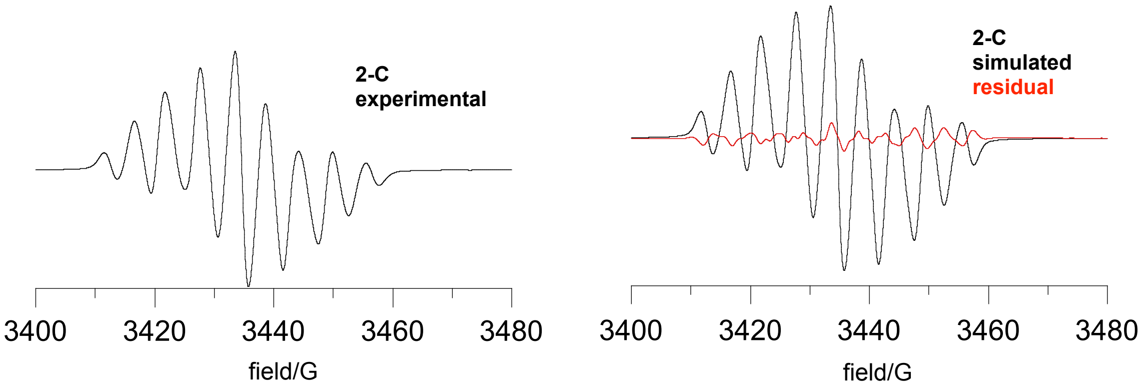

Figure S35. Experimental, simulated and difference spectra for verdazyl radical **2-C**.

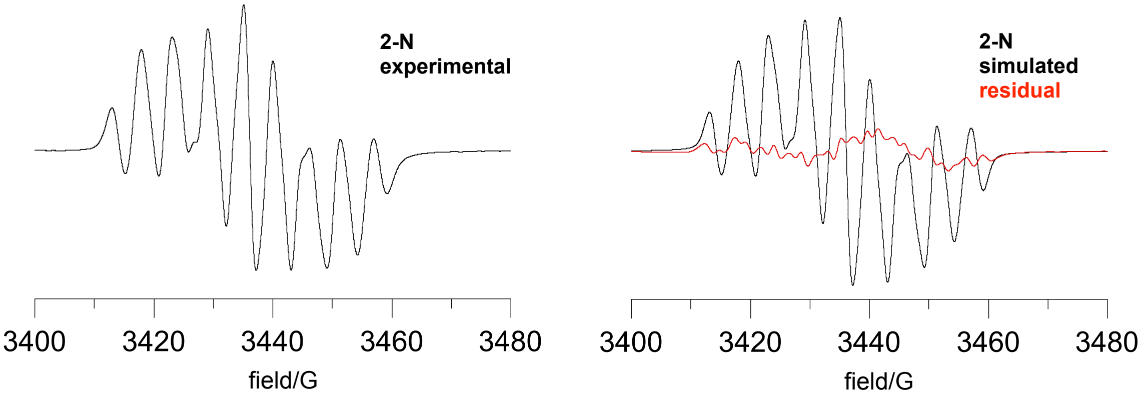

Figure S36. Experimental, simulated and difference spectra for verdazyl radical **2-N**.

Table S11. Hyperfine coupling constants (G) for radicals **2-C** and **2-N**.

radical	$a_{N(1)}$	$a_{N(2)}$	$a_{N(4)}$	$a_{N(5)}$	g^a
**2-C**	6.22	4.71	6.22	4.71	2.0042
**2-N**	6.43	4.77	6.28	4.46	2.0041

[a] Referenced to TEMPO as the internal standard.

b) solid solution spectroscopy

• sample preparation

A solution of polystyrene (0.650–2.085 g, d = 1.04 g cm^{-3}) in dry and distilled CH_2Cl_2 (3 mL) was degassed in vacuum and diradical **1** (0.56–1.57 mg, 0.67–1.88 mmol) was added and mixed till a homogenous mixture was formed. The resulting mixture was degassed in vacuum till complete evaporation of the solvent and formation of a fragile polystyrene film. The film was then dried for 1 h, divided into smaller pieces, placed in EPR tube and tightly packed using a glass rod. The EPR tube containing the sample was filled with argon gas, tightly closed, and variable temperature measurement was performed.

• ***measurements***

Variable temperature EPR spectra for diradicals **1** were recorded on a X-band EMX-Nano spectrometer equipped with a frequency counter typically in a rage 120 – 320 K in degassed solid polystyrene solution (0.88–1.07 mM). At 120 K spectra exhibit patterns consistent with randomly oriented triplets contaminated with a signal from the doublet impurity (the middle singlet). The optimum microwave power for the measurement was determined from the linear portion of the plot of signal intensity (double integral, DI) *vs* square root of microwave power (Figures S37 and S38) at 120 K and 298 K. On the basis of the power sweep plots attenuation 32 dB was selected for measurements. For each temperature three spectra were recorded and averaged. No half-field transition $|\Delta m_s| = 2$ was observed in either of the diradicals. Variable temperature EPR spectra for diradicals **1** are shown in the Figures S39–S42.

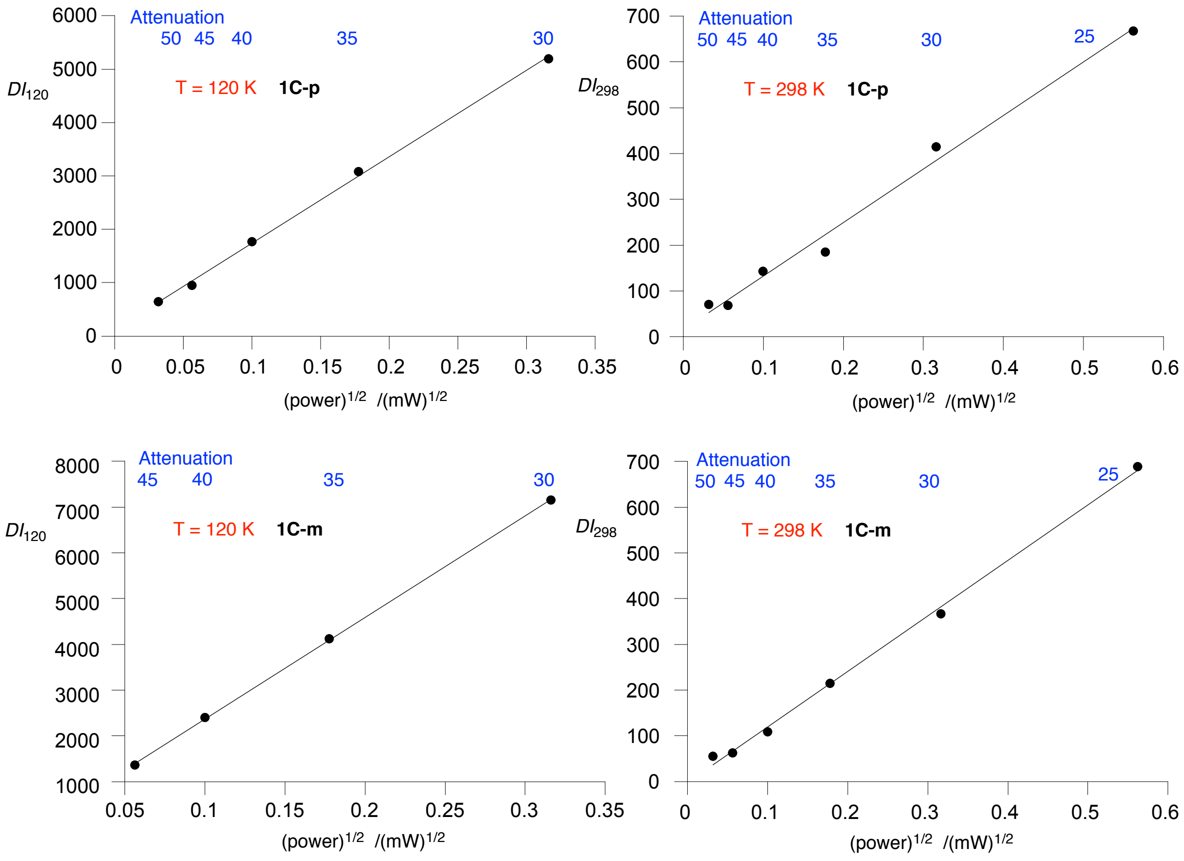

Figure S37. EPR double integral signal intensity (DI) *vs* the square root of microwave power for ~1 mM solutions of radicals **1C** in polystyrene. Top: **1C-p**, best fit line: DI_{120} = 124.2+16186×(power)$^{1/2}$, r^2 = 0.999; DI_{298} = 12.2+1170×(power)$^{1/2}$, r^2 = 0.989; bottom: **1C-m**, best fit line: DI_{120} = 150+22210×(power)$^{1/2}$, r^2 = 0.999; DI_{298} = -2.3+1215×(power)$^{1/2}$, r^2 = 0.997.

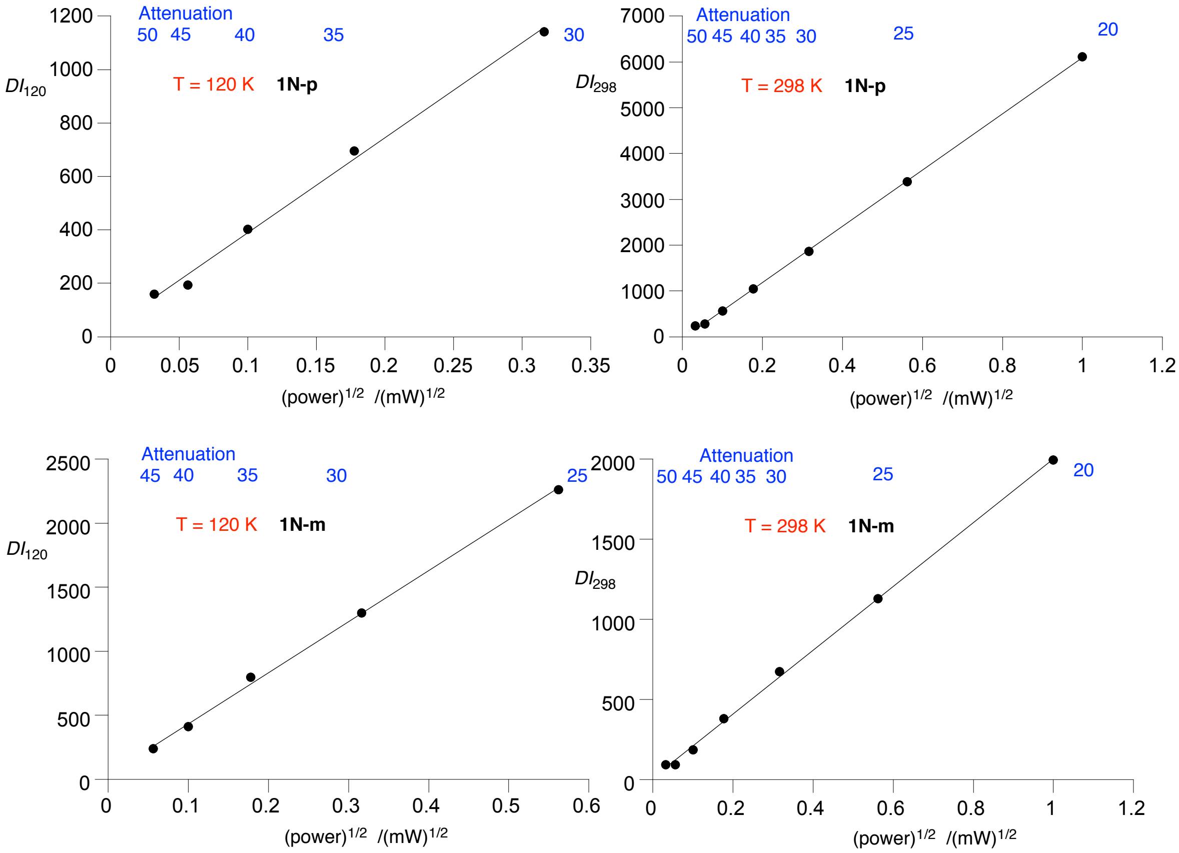

Figure S38. EPR double integral signal intensity (DI) vs the square root of microwave power for ~1 mM solutions of radicals **1N** in polystyrene. Top: **1N-p**, best fit line: DI_{120} = 35.4+3547×(power)$^{1/2}$, r^2 = 0.995; DI_{298} = -37.3+6134×(power)$^{1/2}$, r^2 = 0.999; bottom: **1N-m**, best fit line: DI_{120} = 35.7+3985×(power)$^{1/2}$, r^2 = 0.999; DI_{298} = 11.3+1992×(power)$^{1/2}$, r^2 = 0.999.

• ***spectra analysis and simulation***

EPR spectra were double integrated and the resulting DI intensities were normalized for the intensity at the lowest temperature. The resulting DI_{rel} = DI/DI_T and the DI_{rel}•T product are shown in Tables S12–S15.

Table S12. Double integral and normalized data for **1-Cp**.

Temp /K	DI	DI/DI_{120}	DI_{rel}•T	Temp /K	DI	DI/DI_{120}	DI_{rel}•T
120.3	4388	1.0000	120.34	224.1	2625	0.59822	134.08
124.7	4296	0.97903	122.05	229.4	2577	0.58728	134.74
129.5	4171	0.95055	123.07	234.4	2507	0.57133	133.89
134.2	4068	0.92707	124.42	239.1	2484	0.56609	135.38
139.2	3938	0.89745	124.93	244.1	2454	0.55925	136.52
144.4	3845	0.87625	126.52	249.3	2390	0.54467	135.79

149.2	3734	0.85096	126.94	254.1	2320	0.52871	134.36
154.4	3589	0.81791	126.30	259.1	2290	0.52188	135.24
159.2	3531	0.80469	128.13	264.1	2196	0.50046	132.19
164.2	3454	0.78715	129.21	269.1	2158	0.49180	132.36
169.2	3314	0.75524	127.75	274.3	2154	0.49088	134.66
174.3	3235	0.73724	128.53	279.3	2063	0.47015	131.33
179.2	3161	0.72037	129.07	284.1	2050	0.46718	132.75
184.4	3118	0.71057	131.01	289.1	2023	0.46103	133.30
189.4	2987	0.68072	128.93	294.3	1967	0.44827	131.91
194.4	2939	0.66978	130.19	299.3	1955	0.44553	133.34
199.4	2891	0.65884	131.35	304.1	1914	0.43619	132.65
204.4	2837	0.64654	132.14	309.3	1892	0.43118	133.37
209.1	2761	0.62922	131.59	314.3	1839	0.41910	131.74
214.1	2706	0.61668	132.06	319.1	1834	0.41796	133.38
219.1	2612	0.59526	130.45				

Table S13. Double integral and normalized data for **1-Cm.**

Temp /K	DI	DI/DI_{120}	$DI_{rel}\cdot T$	Temp /K	DI	DI/DI_{120}	$DI_{rel}\cdot T$
120.4	4352	1.0000	120.39	224.1	2639	0.60639	135.90
124.7	4252	0.97702	121.81	229.4	2590	0.59513	136.52
129.3	4127	0.94830	122.58	234.4	2540	0.58364	136.78
134.7	4085	0.93865	126.43	239.1	2476	0.56893	136.06
139.6	3975	0.91337	127.47	244.1	2453	0.56365	137.60
144.2	3919	0.90051	129.87	249.2	2443	0.56135	139.86
149.5	3772	0.86673	129.55	254.1	2384	0.54779	139.21
154.4	3653	0.83938	129.64	259.1	2345	0.53883	139.63
159.2	3558	0.81756	130.12	264.2	2289	0.52597	138.94
164.4	3408	0.78309	128.70	269.4	2220	0.51011	137.40
169.1	3310	0.76057	128.64	274.3	2195	0.50437	138.37
174.2	3235	0.74334	129.47	279.2	2155	0.49517	138.23
179.2	3181	0.73093	130.97	284.1	2109	0.48460	137.68
184.2	3085	0.70887	130.55	289.3	2102	0.48300	139.74
189.2	3038	0.69807	132.05	294.2	2045	0.46990	138.22
194.2	2989	0.68681	133.35	299.3	2033	0.46714	139.83
199.1	2896	0.66544	132.51	304.3	1968	0.45221	137.63

204.2	2852	0.65533	133.80	309.3	1972	0.45312	140.16
209.2	2798	0.64292	134.49	314.1	1952	0.44853	140.89
214.2	2699	0.62017	132.83	319.1	1939	0.44554	142.19
219.2	2693	0.61880	135.61				

Table S14. Double integral and normalized data for **1-Np**.

Temp /K	DI	DI/DI_{119}	$DI_{rel} \cdot T$	Temp /K	DI	DI/DI_{119}	$DI_{rel} \cdot T$
119.2	1067	1.0000	119.18	224.3	1706	1.5989	358.61
124.6	1108	1.0384	129.35	229.3	1656	1.5520	355.81
129.4	1184	1.1097	143.63	234.3	1586	1.4864	348.25
134.4	1259	1.1799	158.58	239.3	1621	1.5192	363.55
139.4	1380	1.2933	180.25	244.3	1655	1.5511	378.88
144.1	1399	1.3112	188.90	249.3	1640	1.5370	383.15
149.1	1559	1.4611	217.86	254.3	1624	1.5220	386.99
154.1	1591	1.4911	229.74	259.3	1645	1.5417	399.73
159.3	1480	1.3871	220.97	264.3	1584	1.4845	392.33
164.1	1604	1.5033	246.64	269.2	1610	1.5089	406.26
169.1	1604	1.5033	254.17	274.2	1601	1.5005	411.47
174.1	1650	1.5464	269.18	279.3	1596	1.4958	417.71
179.3	1646	1.5426	276.60	284.3	1586	1.4864	422.54
184.3	1588	1.4883	274.24	289.2	1567	1.4686	424.78
189.3	1645	1.5417	291.84	294.1	1621	1.5192	446.74
194.3	1666	1.5614	303.37	299.3	1535	1.4386	430.51
199.3	1586	1.4864	296.20	304.1	1545	1.4480	440.27
204.3	1648	1.5445	315.51	309.3	1517	1.4217	439.69
209.3	1652	1.5483	323.98	314.1	1552	1.4545	456.83
214.1	1609	1.5080	322.80	319.1	1565	1.4667	467.98
219.1	1656	1.5520	340.00				

Table S15. Double integral and normalized data for **1-Nm**.

Temp /K	DI	DI/DI_{119}	$DI_{rel} \cdot T$	Temp /K	DI	DI/DI_{119}	$DI_{rel} \cdot T$
120.7	1847	1.0000	120.75	224.2	1084	0.58690	131.56
124.3	1737	0.94044	116.87	229.2	1131	0.61234	140.32

129.3	1773	0.95994	124.10	234.1	1072	0.58040	135.88
134.3	1795	0.97185	130.52	239.1	1018	0.55116	131.80
139.2	1751	0.94802	131.98	244.1	1054	0.57066	139.32
144.2	1705	0.92312	133.11	249.2	976.4	0.52864	131.71
149.4	1628	0.88143	131.72	254.1	969.7	0.52501	133.43
154.1	1549	0.83866	129.27	259.1	995.1	0.53877	139.61
159.2	1448	0.78397	124.80	264.2	954.9	0.51700	136.58
164.2	1472	0.79697	130.83	269.4	997.9	0.54028	145.53
169.2	1422	0.76990	130.25	274.1	934.9	0.50617	138.75
174.2	1367	0.74012	128.90	279.1	896.8	0.48554	135.53
179.2	1330	0.72009	129.01	284.1	922.2	0.49930	141.86
184.2	1358	0.73525	135.40	289.3	861.4	0.46638	134.93
189.4	1317	0.71305	135.04	294.1	893.2	0.48360	142.24
194.3	1249	0.67623	131.42	299.4	840.8	0.45522	136.28
199.4	1240	0.67136	133.88	304.1	921.4	0.49886	151.72
204.4	1198	0.64862	132.57	309.2	833.9	0.45149	139.58
209.2	1198	0.64862	135.67	314.3	831.5	0.45019	141.51
214.1	1167	0.63184	135.30	319.1	828.0	0.44829	143.06
219.2	1162	0.62913	137.89				

The singlet-triplet energy gap $\Delta E_{S\text{-}T} = 2J$ for each diradical was estimated by fitting experimental VT EPR data points (DI_{rel} *vs* T, Tables S12–S15) to the Bleaney-Bowers equation[12] (eq S1).

$$\chi = \frac{Ng^2\mu_B^2}{kT}\left(\frac{2}{3+e^{-\frac{2J}{kT}}}\right) \qquad \text{(eq S1)}$$

It was assumed that each diradical contains 5% of a monoradical impurity. Therefore, for numeral fitting to the eq S1, a two-parameter equation S2 was used:

$$DI_{rel} = {}^{m1}/_{T}\left(\frac{2}{3+e^{-\frac{m2}{T}}}\right) + 0.025 \times {}^{m1}/_{T} \qquad \text{(eq S2)}$$

The resulting singlet-triplet energy gaps estimated from VT-EPR data fitting are shown in Figure S39–S42 and listed in Table S16.

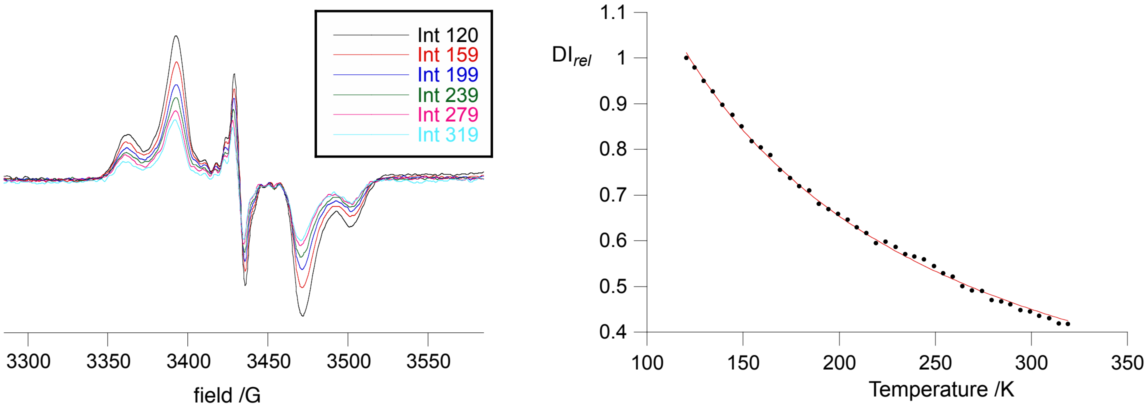

Figure S39. Determination of ΔE_{ST} for 0.94 mM diradical **1-Cp** in polystyrene. Left: variable temperature spectra at 32 dB in the temperature range 120–318 K. Right: plot of DI_{rel} *vs* *T*. in the temperature range 120–319 K. Red line represents the best fitting function (eq. S2) with the following parameters: $m1 = 273(1)$, $m2 = 2J/k_B = -67(3)$ K, $r^2 = 0.998$.

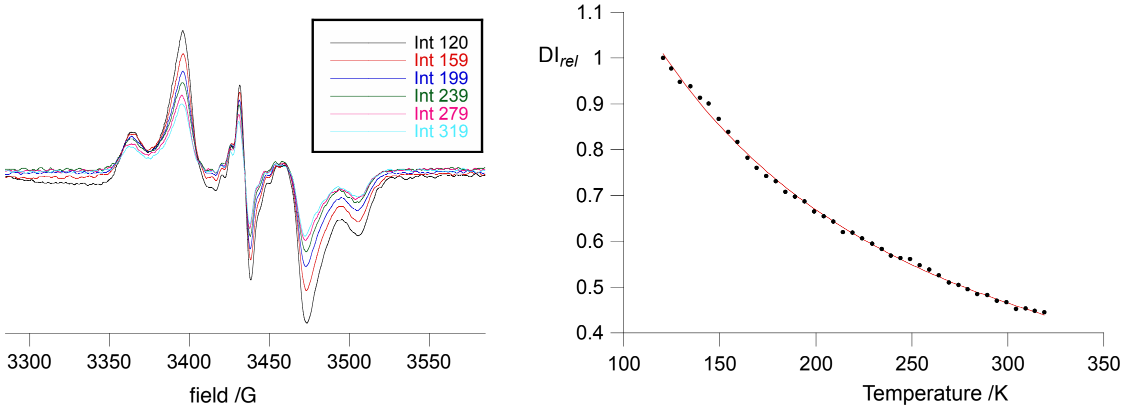

Figure S40. Determination of ΔE_{ST} for 0.88 mM diradical **1-Cm** in polystyrene. Left: variable temperature spectra at 32 dB in the temperature range 120–318 K. Right: a plot of DI_{rel}•T *vs* *T* in the temperature range 120–318 K. The red line represents the best fitting function (eq. S2) with the following parameters: $m1 = 286(1)$, $m2 = 2J/k_B = -83(3)$ K, $r^2 = 0.998$.

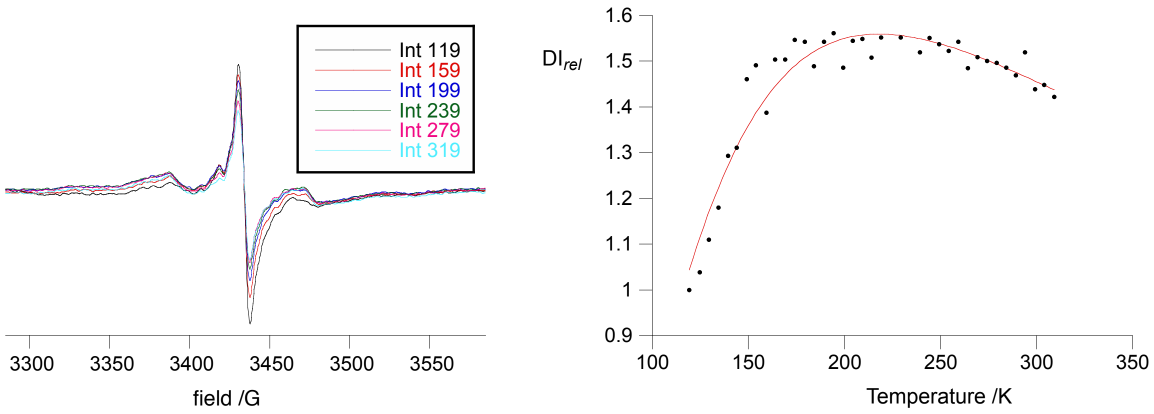

Figure S41. Determination of ΔE_{ST} for 1.2 mM diradical **1-Np** in polystyrene. Left: variable temperature spectra at 32 dB in the temperature range 120–315 K. Right: a plot of DI_{rel} *vs* *T* in the temperature range 120–310 K. The red line represents the best fitting function (eq. S2) with the following parameters: $m1 = 1355(15)$, $m2 = 2J/k_B = -350(3)$ K, $r^2 = 0.96$.

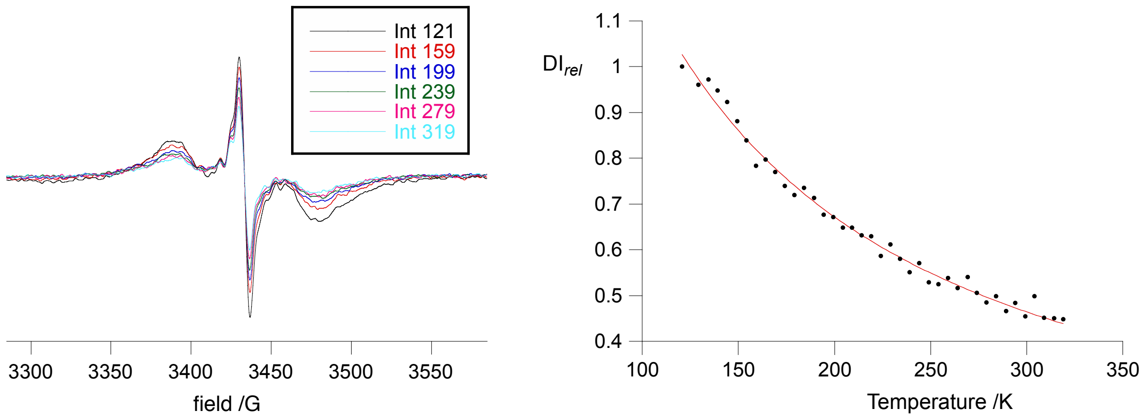

Figure S42. Determination of ΔE_{ST} for 1.02 mM diradical **1-Nm** in polystyrene. Left: variable temperature spectra at 32 dB in the temperature range 119–317 K. Right: a plot of DI$_{rel}$ *vs* T in the temperature range 119–317 K. The red line represents the best fitting function (eq. S2) with the following parameters: $m1 = 283(4)$, $m2 = 2J/k_B = -74(7)$ K, $r^2 = 0.989$.

Table S16. The singlet-triplet energy gap $\Delta E_{S\text{-}T}$ (2J) for diradicals **1** determined by fitting to the Bleaney-Bowers equation eq S1.

	Matrix	$\Delta E_{S\text{-}T}$ /kcal mol^{-1}	$\lvert D/hc \rvert$ $\times 10^{-3}$ /cm^{-1}	$\lvert E/hc \rvert$ $\times 10^{-4}$ /cm^{-1}
**1-Cp**	PS	-0.133(6)	6.28	1.12
**1-Cm**	PS	-0.164(6)	6.34	1.05
**1-Np**	PS	-0.693(6)	7.11	6.04
**1-Nm**	PS	-0.14(1)	5.75	6.00

Simulation of triplet EPR spectra for diradicals **1** was conducted using the *pepper* module in *EasySpin* (Matlab).[13] and results are shown in Figures S43–S46. Assuming an isotropic g value. The resulting absolute values of zero field splitting parameters (*zfp*), $|D/hc|$ and $|E/hc|$, are shown in Table S16. Assuming a point dipole approximation, the mean distance between the spin centers was estimated using equation S3.

$$r = ((D/\mathrm{g})\times 7.19\times 10^{-5})^{-1/3} \qquad \text{(eq S3)}$$

where D (in gauss) is the fitting parameter in the simulated EPR spectrum.

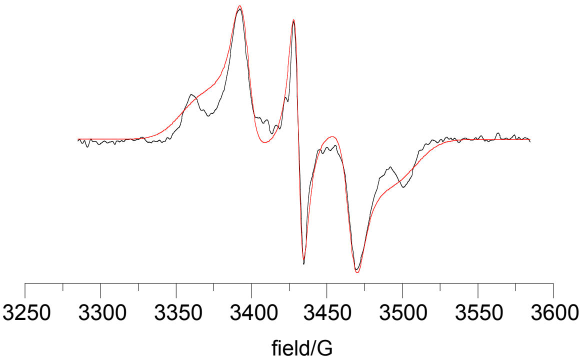

Figure S43. A complete set of fitting parameters for EPR spectrum of 0.94 mM diradical **1-Cp** in polystyrene (295 K, ν = 9.644 GHz). Simulation $|\Delta m_S| = 1$ region (*pepper*, *EasySpin*, rmsd = 0.0549572): Component A, weight = 1.0000, $S = 1$, D = 188.081 MHz, E = -3.37185 MHz, g_{iso} = 2.0081; H-strain (MHz): H_x = 29.6594, H_y = 156.438, H_z = 89.1412; component B, S =1/2, weight = 0.2331, g_{iso}= 2.00822.

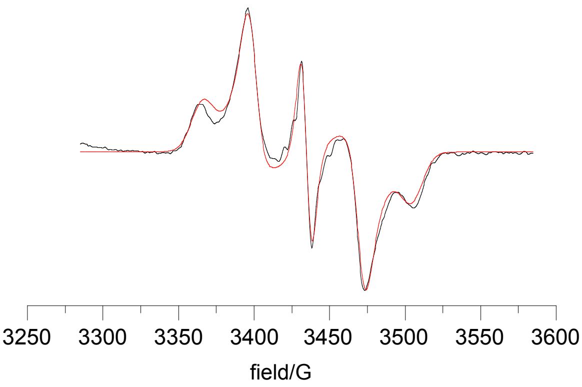

Figure S44. A complete set of fitting parameters for EPR spectrum of 0.88 mM diradical **1-Cm** in polystyrene (120 K, ν = 9.644 GHz). Simulation $|\Delta m_S| = 1$ region (*pepper*, *EasySpin*, rmsd = 0.0361397): Component A, weight = 1.0000, $S = 1$, D = 190.105 MHz, E = -3.14098 MHz, g_{iso} = 2.00587, H-strain (MHz): H_x = 28.9283, H_y = 129.528, H_z = 47.5403; component B, S =1/2, weight = 0.133898, g_{iso} = 2.00603.

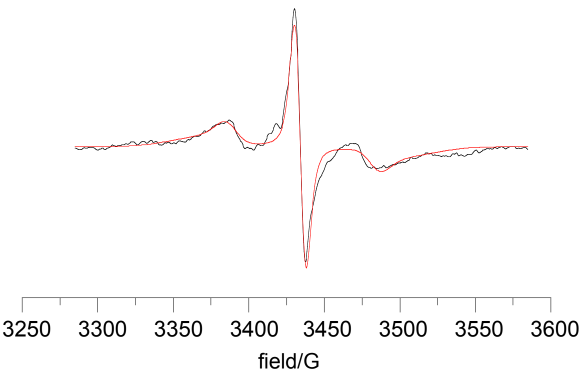

Figure S45. A complete set of fitting parameters for EPR spectrum of 1.08 mM diradical **1-Np** in polystyrene (295 K, ν = 9.644 GHz). Simulation $|\Delta m_S| = 1$ region (*pepper*, *EasySpin*, rmsd = 0.0364238): Component A, weight = 1.0000, $S = 1$, D = 213.006 MHz, E = -18.1163 MHz, g_{iso} = 2.00537, H-strain (MHz): H_x = 39.552, H_y = 207.4, H_z = 120.39; component B, S =1/2, weight = 0.546376, g_{iso} = 2.00638.

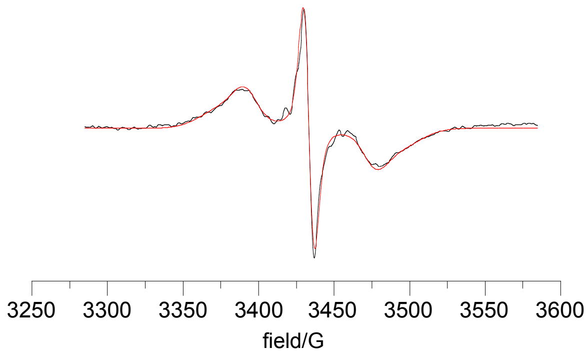

Figure S46. A complete set of fitting parameters for the EPR spectrum of 1.02 mM diradical **1-Nm** in polystyrene (121 K, ν = 9.644 GHz). Simulation $|\Delta m_S| = 1$ region (*pepper, EasySpin*, rmsd = 0.0225999): Component A, weight = 1.0000, S = 1, D = 172.244 MHz, E = -17.9929 MHz, g_{iso} = 2.00632; H-strain (MHz): H_x = 48.2758, H_y = 169.984 H_z = 85.4942; component B, S =1/2, weight = 0.370078, g_{iso} = 2.00691.

7. Magnetization measurements and data analysis

Magnetic susceptibility of polycrystalline samples of diradicals **1** and monoradicals **2** was measured in a polycarbonate capsule fitted in a plastic straw as a function of temperature in cooling (300 K→ 2 K) and then in heating (2 K→ 400 K) modes with 3 min of temperature stabilization at each temperature (0.2 K increment in a range 2–10 K, 1 K increment in a range 11-49 K, and 5 K increment in a range 50-300) at 0.60 T, using a SQUID magnetometer (Quantum Design MPMS-XL-7T). Measurements of magnetization M *vrs* field H (0 T → 7 T) were conducted at 2 K. No significant differences in magnetic susceptibility were observed for data collected in heating and cooling modes in the range 2–300 K. Above 300 K abrupt changes in magnetic susceptibilities were observed in samples **2-N** (380 K), presumably due to a Cr–Cr transition. Analysis was conducted for data obtained on the first heating run.

The magnetic effect of the capsule (scaled by the mass of the capsule) was subtracted from the raw data for the sample using the following method. For a given temperature T and magnetic field B magnetization $M(B,T)$ was measured. Raw data collected by SQUID magnetometer at each such a point are the electric signals $U(x)$ measured as a function of sample position x in the SQUID pick-up coil (so called second-order gradiometer, Figure S47). The raw signal of empty polycarbonate capsule $U_C(x)$ was measured independently as a function of temperature and magnetic field. For each measured raw data point $U(x)$, signal of the empty polycarbonate capsule, $U_C(x)$, was subtracted. The resulting difference of signals, $U(x) - U_C(x)$, was fitted to an analytical function provided by Quantum Design MPMS (Application Note 1014-213 https://www.qdusa.com/sitedocs/appNotes/mpms/1014-213.pdf), which gave a

magnetic moment value *M*. This procedure was applied for each experimental data point to obtain *M(T)* and *M(B)* values and executed using an algorithm written in MatLab program (version R2019a). The corrected data of magnetization of the sample was used for further analysis.

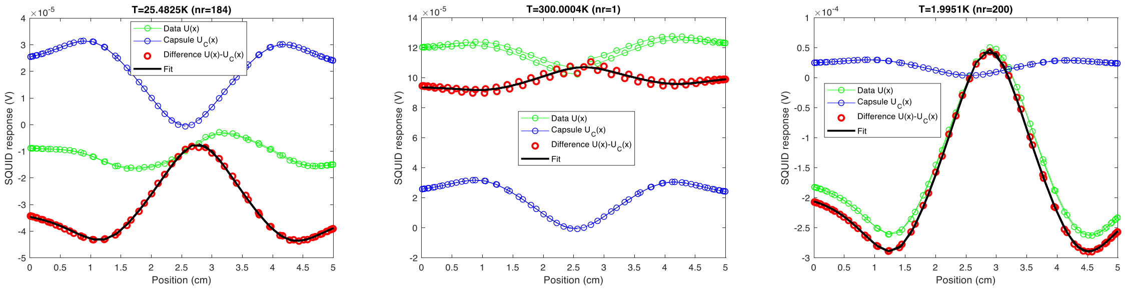

Figure S47. Examples of analysis of raw data: a) *U(x)* < $U_C(x)$, b) *U(x)* ~ $U_C(x)$ (magnetic moment crosses 0 line); c) *U(x)* > $U_C(x)$.

Analysis of magnetization data obtained for all samples as described above was performed by calculating molar total magnetic susceptibility, χ_{tot}. Only for **1-Cp**, **1-Np** and **2-N** the data was analyzed further by determining the diamagnetic correction, χ_{dia}, and to calculate the paramagnetic component, χ_{p}, of the magnetic susceptibility. For **1-Cp** and **2-N** and the diamagnetic correction (sample and the sample holder) was estimated from the linear portion of high temperature $\chi_{\mathrm{tot}} \bullet T$ *vs* *T* plot assuming ideal paramagnetic behavior of the sample and using the Curie law (eq 4).

$$\chi \bullet T = (\chi_p + \chi_{dia}) \bullet T = C + \chi_{dia} \bullet T \quad \text{(eq 4)}$$

where $C = 0.375$ cm^{3}mol^{-1}K for an ideal paramagnet.

For **1-Np** the diamagnetic correction was established from the low temperature plot assuming strong antiferromagnetic interactions and hence $\chi_{\mathrm{p}} = 0$.

Diradicals **1-Cp** and **1-Np** were treated as dimers of monoradicals in a mean magnetic filed. Therefore, analysis of the χ_{p}(T) curves was performed using the Bleaney-Bowers model[12] (eq S5) on the basis of the basis of Heisenberg Hamiltonian for two spins S = 1/2, $\hat{H} = -2J\hat{S}_1 \cdot \hat{S}_2$:

$$\chi T = \frac{Ng^2\mu_B^2}{k}\left(\frac{2}{3+e^{-\frac{2J}{kT}}}\right) \quad \text{(eq S5)}$$

Attempts at modeling magnetic data of monoradical **2-C** with the Bonner-Fischer[14] model (eq S6) for regular antiferromagnetic chain of spins S = ½ were unsuccessful due to curvature of

the χ_p(T) plots above 100 K.

$$\chi_{BF}(T) = \frac{N_A g^2 \mu_B^2}{k_B T} \frac{0.25+0.074975x+0.075235x^2}{1+0.9931x+0.172135x^2+0.757825x^3} \quad \text{(eq S6)}$$

where

$$x = |2J|/k_B T$$

a) Diradical **1-Cp**

A microcrystalline sample of diradical **1-Cp** (m = 19.10 mg, 2.361×10^{-5} mol, M_w = 808.92 g mol^{-1}) was analyzed at 0.60 T. The total magnetic susceptibility χ_{tot} was calculated per mole of spins and χ_{tot}(T) and $\chi_{tot}T$(T) plots are shown in Figures S48 and S49, respectively.

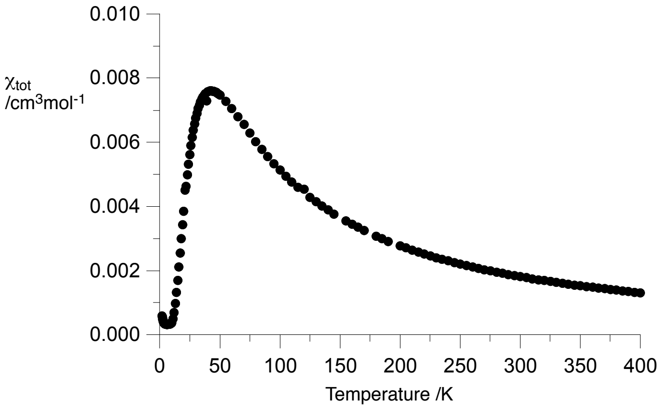

Figure S48. Total magnetic susceptibility χ_{tot} (per spin) *vs* temperature of **1-Cp**.

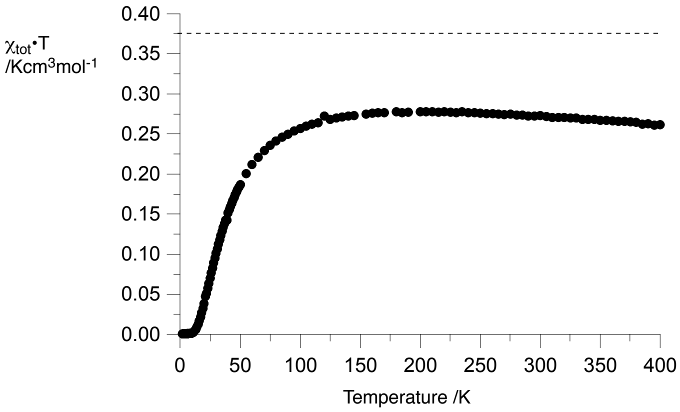

Figure S49. A plot of χ_{tot}•T (per spin) *vs* temperature for **1-Cp**. The dotted line marks χ_p•T = 0.375 cm^3 mol^{-1} K for an ideal paramagnet.

Diamagnetic correction was determined from the high temperature portion of the χ_{tot}•T(T) plot assuming saturation of the magnetization. Results are shown in Figure S50.

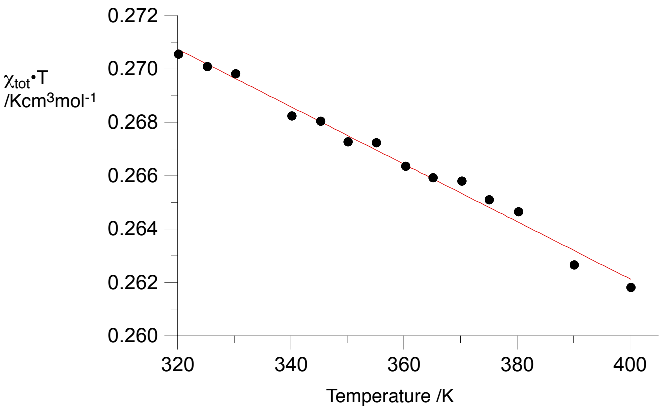

Figure S50. High temperature portion of the of the χ_{tot}•T (per spin) *vs* temperature plot for **1-Cp**. Best fit line: χ_{tot}•T = 0.305(1) – 1.08(4)10^{-4}×T, r^2 = 0.987.

The established lower estimate of the diamagnetic correction was used to derive χ_p. A χ_p•T(T) plot is shown in Figure S51 and fitted to the Bleaney-Bowers model (eq S5).

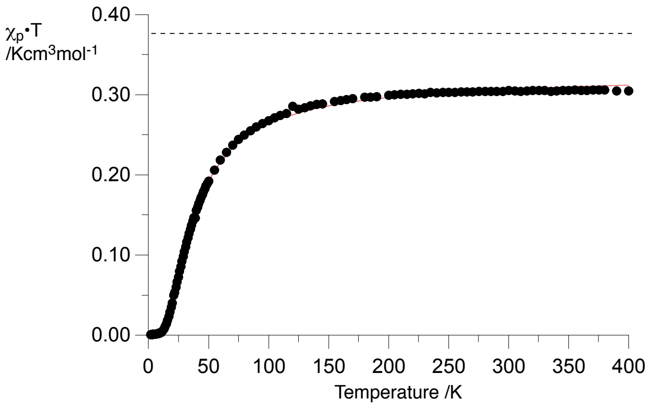

Figure S51. A plot of χ_p•T (per spin) *vs* temperature for **1-Cp** after applying the diamagnetic correction of χ_{dia} = – 1.08(4)10^{-4} cm^3 mol^{-1} determined in Figure S48. The dotted line marks χ_p•T = 0.375 cm^3 mol^{-1} K for an ideal paramagnet. The red line represents fitting to the Bleaney-Bowers model (eq S5) with the $2J/k_B$ = -66.9(2) K (r^2 = 0.9995).

<u>*b*) Diradical **1-Cm**</u>

A microcrystalline sample of diradical **1-Cm** (m = 7.14 mg, 0.883×10^{-5} mol, M_w = 808.92 g mol^{-1}) was analyzed at 0.60 T. The total magnetic susceptibility χ_{tot} was calculated per mole of spins and χ_{tot}(T) and $\chi_{tot}T$(T) plots are shown in Figures S52 and S53, respectively.

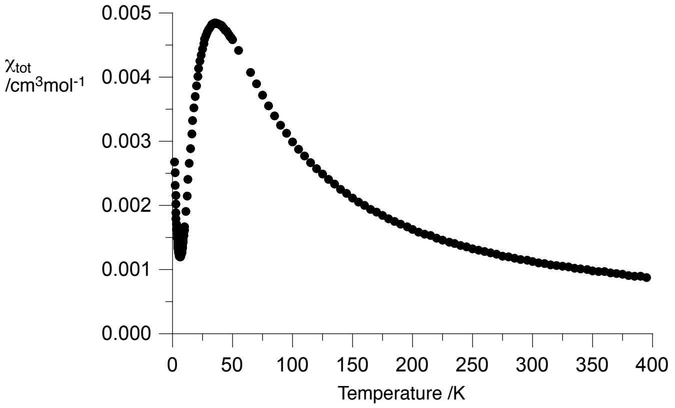

Figure S52. Total magnetic susceptibility χ_{tot} (per spin) *vs* temperature of **1-Cm**.

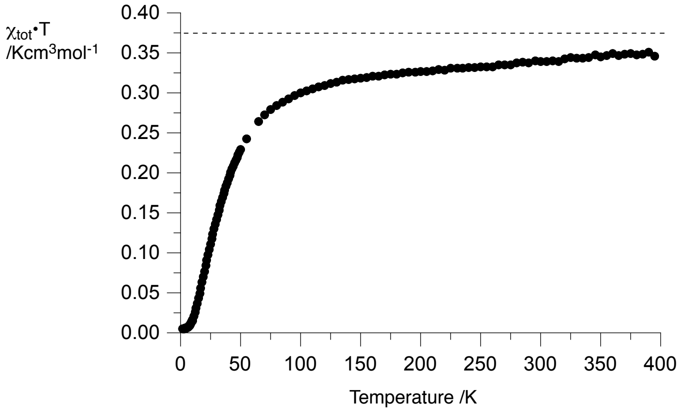

Figure S53. A plot of χ_{tot}•T (per spin) *vs* temperature for **1-Cm**. The dotted line marks χ_p•T = 0.375 cm^3 mol^{-1} K for an ideal paramagnet.

c) Diradical **1-Np**

A microcrystalline sample of diradical **1-Np** (*m* = 3.89 mg, 0.481×10^{-5} mol, M_w = 808.92 g mol^{-1}) was analyzed at 0.60 T. The total magnetic susceptibility χ_{tot} was calculated per mole of spins and χ_{tot}(T) and $\chi_{tot}T$(T) plots are shown in Figures S54 and S55, respectively.

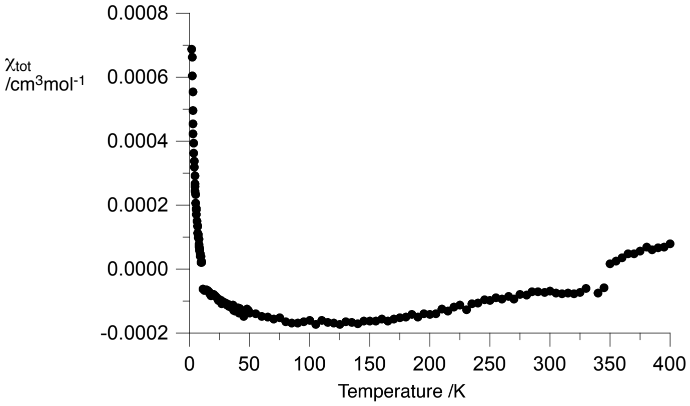

Figure S54. Total magnetic susceptibility χ_{tot} (per spin) *vs* temperature of **1-Np**.

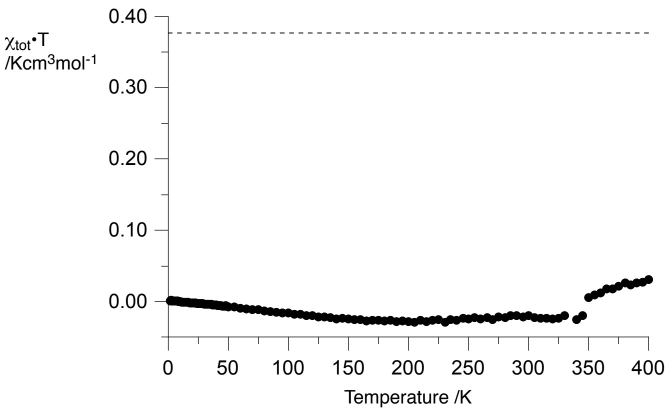

Figure S55. A plot of χ_{tot}•T (per spin) *vs* temperature for **1-Np**. The dotted line marks χ_p•T = 0.375 cm^3 mol^{-1} K for an ideal paramagnet.

Diamagnetic correction was determined from fitting the low temperature portion of the χ_{tot}•T(T) data in the range 2–75 K to a linear function, as shown in Figure S56. Using the diamagnetic correction of χ_{dia} = -1.76(1)×10^{-4} cm^3 mol^{-1} determined in Figure S56 was used to determine χ_p. The resulting full χ_p•T vs T plot is shown in Figure S57, while the fit of the 2-300 K portion of the plot to the Bleaney-Bowers model (eq S5) in Figure S58.

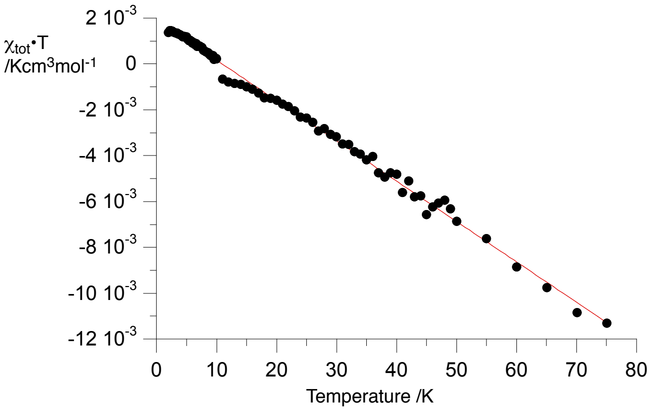

Figure S56. A plot of χ_{tot}•T (per spin) *vs* temperature for **1-Np**. The dotted line marks χ_p•T = 0.375 cm^3 mol^{-1} K for an ideal paramagnet. χ_{tot}•T = 0.00193(3) – 0.000176(1)•T, r^2 = 0.996.

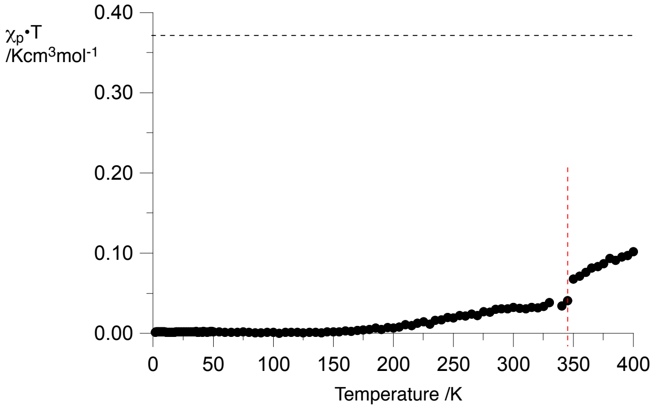

Figure S57. Plot of χ_p•T (per spin) *vs* temperature for **1-Np** after applying diamagnetic correction χ_{dia} = -1.76(1)×10^{-4} cm^3 mol^{-1} determined in Figure S54. The horizontal dotted line marks χ_p•T = 0.375 cm^3 mol^{-1} K for an ideal paramagnet. The red vertical line marks abrupt change.

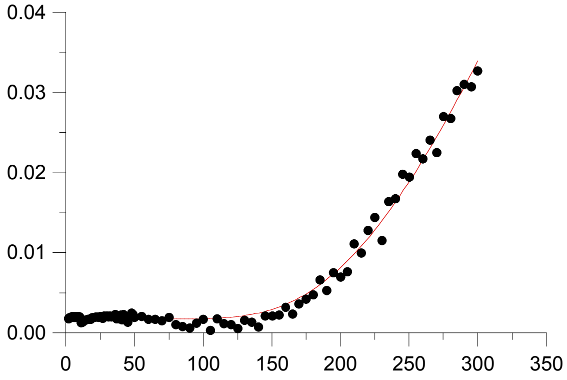

Figure S58. A plot of χ_p•T (per spin) *vs* temperature for **1-Np** after applying the diamagnetic correction of χ_{dia} = – 1.76(1)10^{-4} cm^3 mol^{-1} determined in Figure S54. The red line represents fitting to the Bleaney-Bowers model (eq S5) with the $2J/k_B$ = -1018(18) K (r^2 = 0.990).

d) Diradical **1-Nm**

A microcrystalline sample of verdazyl derivative **1-Nm** (m = 17.435 mg, 2.155×10^{-5} mol, M_w = 808.92 g mol^{-1}) was analyzed at 0.60 T. The total magnetic susceptibility χ_{tot} was calculated per mole of spins and χ_{tot}(T) and $\chi_{tot}T$(T) plots are shown in Figures S59 and S60, respectively.

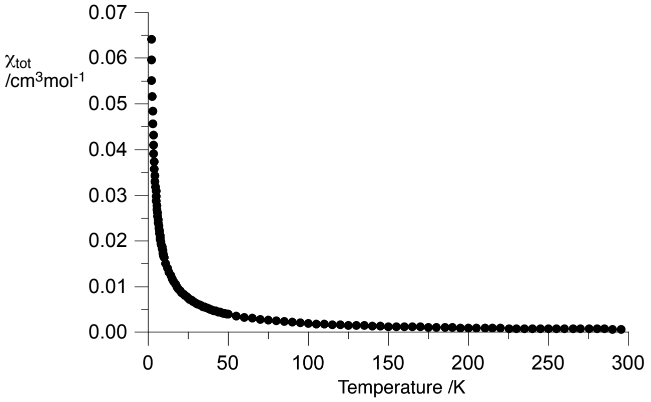

Figure S59. Total magnetic susceptibility χ_{tot} (per spin) *vs* temperature of **1-Nm**.

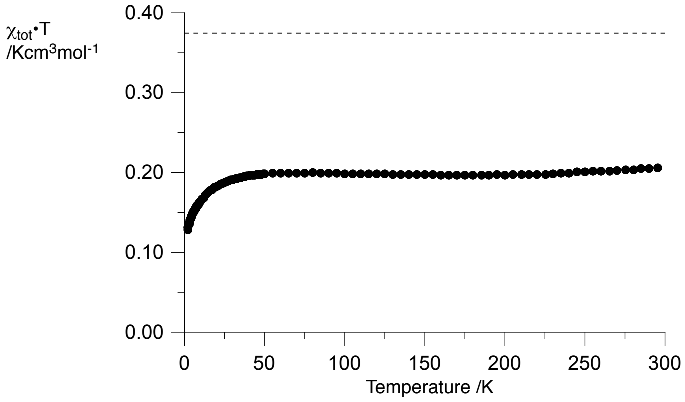

Figure S60. A plot of χ_{tot}•T (per spin) *vs* temperature for **1-Nm**. The dotted line marks χ_p•T = 0.375 cm^3 mol^{-1} K for an ideal paramagnet.

e) Radical **2-C**

A microcrystalline sample of verdazyl derivative **2-C** (m = 14.22 mg, 3.206×10^{-5} mol, M_w = 443.52 g mol^{-1}) was analyzed at 0.60 T. The total molar magnetic susceptibility χ_{tot}(T) and $\chi_{tot}T$(T) plots are shown in Figures S61 and S62, respectively.

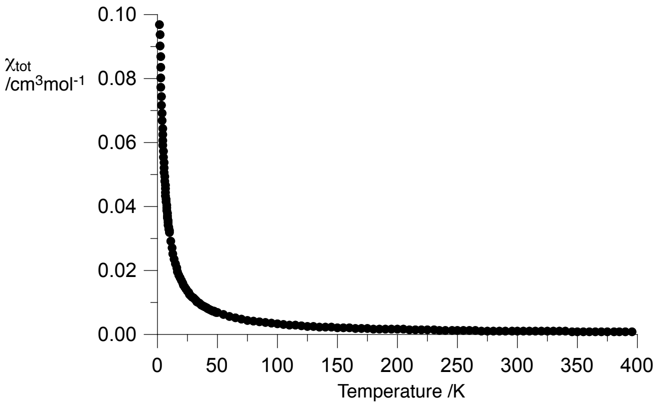

Figure S61. Total molar magnetic susceptibility χ_{tot} *vs* temperature of **2-C**.

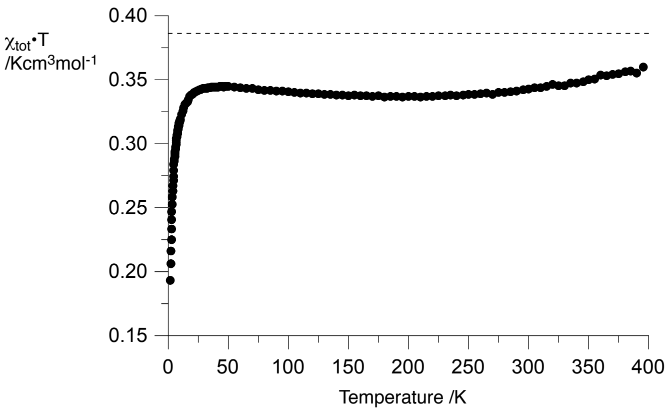

Figure S62. A plot of χ_{tot} •T *vs* temperature of **2-C**. The dotted line marks χ_p•T = 0.375 cm^3 mol^{-1} K for an ideal paramagnet.

f) Radical **2-N**

A microcrystalline sample of monoradical **2-N** (m = 29.495 mg, 6.650×10^{-5} mol, M_w = 443.52 g mol^{-1}) was analyzed at 0.60 T. The total molar magnetic susceptibility χ_{tot}(T) and $\chi_{tot}T$(T) plots are shown in Figures S63 and S64, respectively.

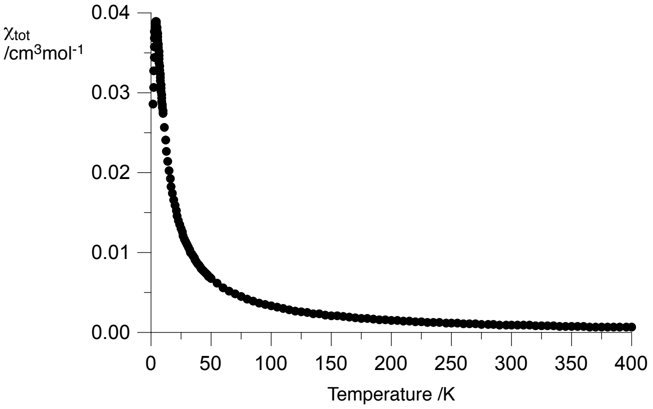

Figure S63. Total molar magnetic susceptibility χ_{tot} *vs* temperature of **2-N**.

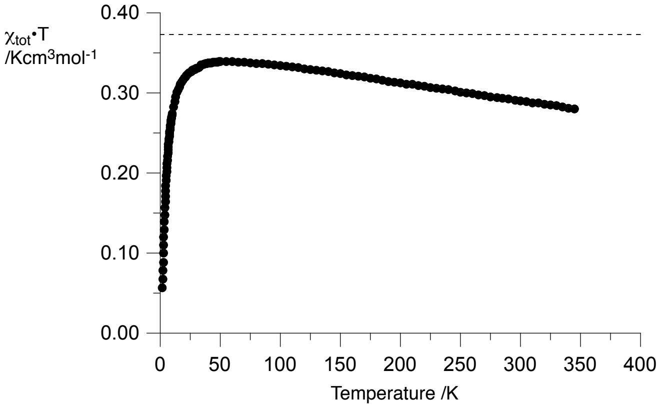

Figure S64. A plot of χ_{tot}•T *vs* temperature of **2-N**. The dotted line marks χ_p•T = 0.375 cm^3 mol^{-1} K for an ideal paramagnet.

Diamagnetic correction was determined by a linear fit of high temperature portion of the χ_{tot}•T(T) plot (Figure S65).

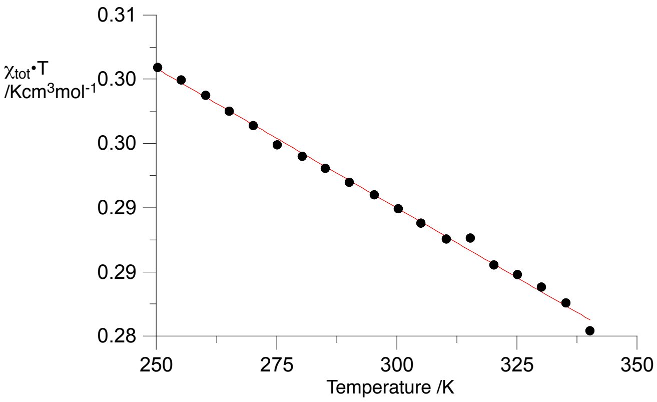

Figure S65. High temperature portion of the of the χ_{tot}•T (per spin) *vs* temperature plot for **2-N**. Best fit line: χ_{tot}•T = 0.355(1) – 0.000217(3)•T, r^2 = 0.996.

The diamagnetic correction of χ_{dia} = -2.17(3)×10^{-4} cm^3 mol^{-1} determined in Figure S65 was used to determine χ_p. The resulting full χ_p•T *vs* T plot is shown in Figure S66.

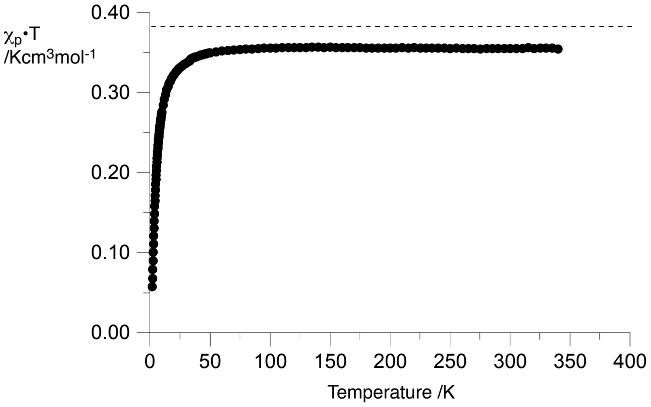

Figure S66. A plot of χ_p•T *vs* temperature for **2-N** after applying the diamagnetic correction of χ_{dia} = – 2.17(3)10^{-4} cm^3 mol^{-1} determined in Figure S63. The dotted line marks χ_p•T = 0.375 cm^3 mol^{-1} K for an ideal paramagnet.

8. Computational details

Quantum-mechanical calculations were carried out using Gaussian 09 suite of programs.[15] Geometry optimizations for model open-shell singlet (using the broken symmetry approach, **1-OSS**), triplet (**1-T**) and the closed-shell analogue (**1-CS**) were undertaken at the (U)B3LYP/6-31G(2d,p) level of theory in vacuum using tight convergence limits without symmetry constraints. For the purpose of computational analysis, the *i*-PrO groups were replaced with MeO in the model compounds. Vibrational frequency calculations were used to characterize the nature of the stationary points and to obtain thermodynamic parameters. Zero-point energy (ZPE) corrections were scaled[16] by 0.9806 and results are shown in Table S15.

□□□ *$\Delta E_{S\text{-}T}$* ***energy gap calculations***

Singlet – triplet energy gaps for diradicals were determined using the Yamaguchi procedure[17-19] (eq S7):

$$\Delta E_{S-T} = 2J = 2\frac{E_{BS}-E_T}{\langle S^2\rangle_T-\langle S^2\rangle_{BS}} \qquad \text{eq. S7}$$

where the SCF energies of the triplet (E_T) and broken symmetry singlet (E_{BS}) corrected for ZPE and total spin angular momenta $\langle S^2\rangle$ before spin annihilation. The SCF energy and angular momenta were obtained in single point calculations at the UB3LYP/6-311+G(2d,p) //UB3LYP/6-31G(2d,p) level of theory in benzene dielectric medium. The solvation was implemented with the PCM model[20] using the "SCRF(solvent=Benzene)" keyword.

The results are shown in Table S17.

Table S17. DFT results for model diradicals **1'**.

Diradical	E_{SCF} [a] /Ha	<S^2>[a]	ZPE [b]	ΔE_{ST} [c] /kcal mol^{-1}	ΔH_{rel} [d] /kcal mol^{-1}
**1'-Cm**					
**OSS** *d*	-2357.205572	1.040579	0.648729		0.0
**T**	-2357.205800	2.0458	0.648737	0.23	-0.13
**CS**	-2357.1707604		0.649023		24.63
**1'-Cp**					
**OSS** *d*	-2357.206983	1.048612	0.648771		0.0
**T**	-2357.206322	2.039466	0.648753	-0.81	0.45
**CS**	-2357.171983		0.649141		24.21
**1'-Nm**					
**OSS** *d*	-2357.2069704	1.042519	0.64869		0.0
**T**	-2357.207179	2.046073	0.648695	0.25	-0.15
**CS**	-2357.1719558		0.648524		24.56
**1'-Np**					
**OSS** *d*	-2357.2080475	1.016105	0.648792		0.0
**T**	-2357.20722	2.042651	0.64868	-0.87	0.53
**CS**	-2357.1883321		0.649766		13.93

[a] Obtained with the (U)B3LYP/6-311+G(2d,p)//B3LYP/6-31G(d,p) method in benzene dielectric medium. [b] Obtained with the (U)B3LYP/6-31G(d,p) method in vacuum. [c] Adiabatic energy gap calculated with the Yamaguchi protocol, ref. [17-19] using SCF energies with ZPE correction. [d] Calculates as a difference of SCF energies with the enthalpic correction.

b) exchange interaction energy in XRD structures

The effective spin-spin exchange interaction J_{DFT} for experimental geometries of **2-C** and **1-Np** was determined using the Yamaguchi procedure[17-19] (eq S7, above). Energies were obtained by single point calculations for a molecule of **1-Np** and two selected pairs of molecules at crystallographically determined coordinates at the UB3LYP/6-311++G(2d,p) level of theory.[21] The input geometries and calculated exchange interaction energies for **2-C** and **1-Np** are shown in Figures S67–S69.

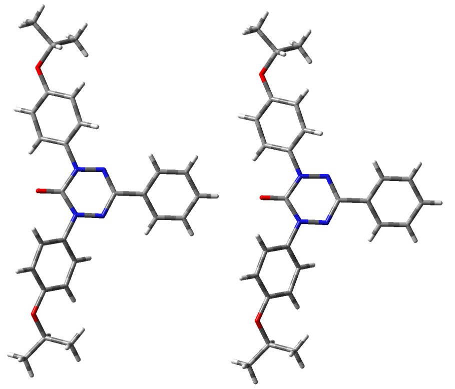

Figure S67. A pair of molecules **2-C** forming an infinite chain –C=O···H-Ph–

J_{ST} = –0.04 cm^{-1}

$2J$ = -0.22 cal mol^{-1}

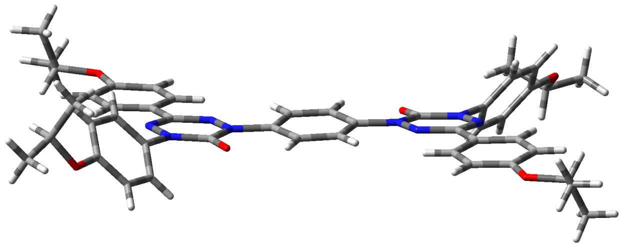

Figure S68. Single molecule of **1-Np** in XRD coordinates.

J_{ST} = -478.93 cm^{-1}

$2J$ = -2.74 kcal mol^{-1}

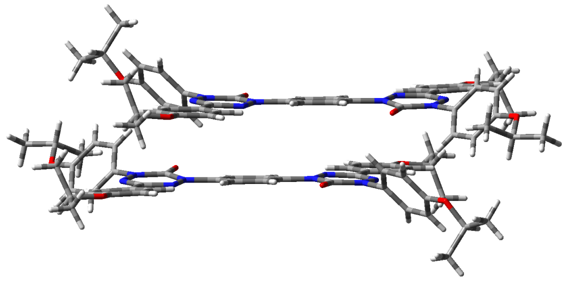

Figure S69. The close pair of molecules of **1-Np** in XRD coordinates.

J_{ST} = -483.9 cm^{-1}

$2J$ = -2.77 kcal mol^{-1}

c) diradicaloid character

Diradical character index *y* was calculated using the occupancy *n* of the lowest unoccupied natural orbital (LUNO) using the Yamaguchi equation:[17]

$$y = 1 - \frac{2T}{1+T^2} \quad \text{eq S8}$$

where *T* is calculated using the occupation number *n* of natural orbitals derived with the UHF method

$$T = \frac{n_{HONO} - n_{LUNO}}{2} \quad \text{eq S9}$$

y = 0 for the closed-shell and *y* = 1 for the pure singlet diradical. The occupancy numbers n_{HOMO} and n_{LUMO} were obtained from natural orbital population analysis (pop=no) using the broken symmetry UHF/6-311G(d,p) // UB3LYP/6-31G(2d,p) method in gas phase.

d) electronic excitation data

Electronic excitation energies in CH_2Cl_2 dielectric medium were obtained for diradicals **1** at the UB3LYP/6-311+G(2d,p)//UB3LYP/6-31G(2d,p) level using the time-dependent DFT method[22]

supplied in the Gaussian package. Solvation models in both types of calculations were implemented with the PCM model[20] using the SCRF(solvent=CH_2Cl_2) keyword. Energies of FMOs involved in the low energy transitions are listed in Table S18.

Table S18. Calculated energies for selected MOs.[a]

Radical	α-HOMO-1 /eV	α-HOMO /eV	α-LUMO /eV	α-LUMO+1 /eV	β-HOMO-1 /eV	β-HOMO /eV	β-LUMO /eV	β-LUMO+1 /eV
**2'-C**	-5.923	-5.001	-1.206	-0.293	-5.765	-5.765	-2.740	-1.042
**1'-Cm**	-5.011	-5.009	-1.399	-1.045	-5.812	-5.748	-2.762	-2.752
**1'-Cp**	-5.751	-5.021	-2.755	-1.592	-5.751	-5.021	-2.755	-1.592
**2'-N**	-5.896	-5.077	-1.062	-0.400	-6.139	-5.668	-2.759	-0.914
**1'-Nm**	-5.152	-5.067	-1.107	-1.078	-5.699	-5.671	-2.806	-2.793
**1'-Np**	-5.693	-5.103	-2.813	-1.081	-5.693	-5.103	-2.813	-1.081

[a] Obtained with the UB3LYP/6-311+G(2d,p) // UB3LYP/6-31G(2d,p) method in CH_2Cl_2 dielectric medium.

e) partial output from TD-DFT calculation

```
TD-DFT in CH2Cl2 at the UB3LYP/6-311+G(2d,p)//UB3LYP/6-31G(2d,p) level of theory
```

1'-Cm-T

```
Excited State 1:  3.036-A  2.1817 eV  568.30 nm  f=0.0614  <S**2>=2.054
    183A -> 184A       0.14188
    180B -> 183B       0.58965
    181B -> 182B       0.75112
 This state for optimization and/or second-order correction.
 Total Energy, E(TD-HF/TD-KS) =  -2356.55832391
 Copying the excited state density for this state as the 1-particle RhoCI
density.

 Excited State 2:  3.036-A  2.1960 eV  564.59 nm  f=0.2092 <S**2>=2.055
    182A -> 184A       0.14831
    180B -> 182B       0.60333
    181B -> 183B       0.73657

 Excited State 3:  3.050-A  2.6151 eV  474.11 nm f=0.0137  <S**2>=2.076
    182A -> 184A      -0.41181
    183A -> 185A      -0.25557
    170B -> 182B      -0.15221
    171B -> 183B      -0.10731
    176B -> 182B       0.12131
    177B -> 183B       0.32873
    178B -> 183B      -0.16162
    180B -> 182B       0.61778
```

```
   181B -> 183B      -0.37557

 Excited State 4:  3.041-A  2.6198 eV  473.27 nm  f=0.0375 <S**2>=2.062
   182A -> 185A      -0.27532
   183A -> 184A      -0.44010
   170B -> 183B      -0.16472
   171B -> 182B      -0.12018
   176B -> 183B       0.14612
   177B -> 182B       0.34414
   178B -> 182B      -0.14502
   180B -> 183B       0.59576
   181B -> 182B      -0.33208

 Excited State 5:  3.058-A 2.6612 eV  465.90 nm  f=0.0851  <S**2>=2.088
   182A -> 185A      -0.23075
   183A -> 184A      -0.44536
   176B -> 183B       0.15555
   177B -> 182B       0.16107
   178B -> 182B       0.47434
   179B -> 183B       0.45373
   180B -> 183B      -0.28654
   181B -> 182B       0.36436

 Excited State 6:  3.064-A 2.6752 eV  463.46 nm  f=0.0142  <S**2>=2.098
   182A -> 184A       0.50833
   183A -> 185A       0.31407
   170B -> 182B       0.10084
   176B -> 182B      -0.22712
   177B -> 183B      -0.18376
   178B -> 183B      -0.24032
   179B -> 182B      -0.28339
   180B -> 182B       0.35283
   181B -> 183B      -0.45916

 Excited State 7:  3.066-A 2.7066 eV  458.09 nm  f=0.0363  <S**2>=2.100
   182A -> 185A       0.20033
   183A -> 184A       0.25397
   176B -> 183B      -0.15315
   178B -> 182B       0.46554
   179B -> 183B       0.51896
   180B -> 183B       0.40188
   181B -> 182B      -0.39287

 Excited State 8:  3.090-A 2.7174 eV  456.26 nm  f=0.0215  <S**2>=2.137
   182A -> 184A       0.13874
   178B -> 183B       0.58872
   179B -> 182B       0.64626
   180B -> 182B       0.27060
   181B -> 183B      -0.24339
```

1′-Cp-OSS

```
Excited State 1:  1.266-A  1.9486 eV  636.26 nm  f=0.0000  <S**2>=0.150
   182A -> 183A      -0.70668
   182B -> 183B       0.70670
 This state for optimization and/or second-order correction.
 Total Energy, E(TD-HF/TD-KS) =  -2356.56773669
 Copying the excited state density for this state as the 1-particle RhoCI
density.

 Excited State2:  1.265-A  1.9493 eV  636.04 nm  f=0.0022  <S**2>=0.150
```

```
   182A -> 183A       0.70668
   182B -> 183B       0.70666

 Excited State 3:  2.284-A 2.1766 eV  569.63 nm  f=0.2430  <S**2>=1.054
   180A -> 183A       0.14034
   181A -> 183A       0.64646
   182A -> 184A      -0.17337
   180B -> 183B       0.14034
   181B -> 183B       0.64647
   182B -> 184B       0.17337

 Excited State 4:  2.276-A  2.1778 eV  569.32 nm  f=0.0027 <S**2>=1.045
   180A -> 183A       0.14776
   181A -> 183A       0.64661
   182A -> 184A       0.16956
   180B -> 183B      -0.14776
   181B -> 183B      -0.64660
   182B -> 184B       0.16956

 Excited State 5:  2.293-A 2.5487 eV  486.46 nm  f=0.0548  <S**2>=1.064
   172A -> 183A      -0.12820
   178A -> 183A       0.22037
   180A -> 183A      -0.19015
   181A -> 183A      -0.14006
   182A -> 184A      -0.57284
   182A -> 185A       0.15360
   172B -> 183B       0.12819
   178B -> 183B       0.22037
   180B -> 183B      -0.19014
   181B -> 183B      -0.14006
   182B -> 184B       0.57285
   182B -> 185B      -0.15360

 Excited State 6:  2.280-A 2.5494 eV  486.33 nm  f=0.0010  <S**2>=1.049
   172A -> 183A      -0.12592
   178A -> 183A       0.22026
   180A -> 183A      -0.18962
   181A -> 183A      -0.13354
   182A -> 184A       0.57501
   182A -> 185A      -0.15361
   172B -> 183B      -0.12593
   178B -> 183B      -0.22027
   180B -> 183B       0.18963
   181B -> 183B       0.13354
   182B -> 184B       0.57499
   182B -> 185B      -0.15361

 Excited State 7:  2.352-A  2.6950 eV  460.06 nm f=0.2254  <S**2>=1.133
   179A -> 183A      -0.68765
   179B -> 183B       0.68765

 Excited State 8:  2.482-A 2.7062 eV  458.14 nm  f=0.0000  <S**2>=1.290
   178A -> 184A       0.12872
   179A -> 183A       0.65867
   181A -> 184A       0.14315
   178B -> 184B       0.12872
   179B -> 183B       0.65867
   181B -> 184B       0.14315
```

1′-Nm-T

```
Excited State 1:  3.023-A 2.0410 eV  607.47 nm  f=0.1469  <S**2>=2.034
    182A -> 184A       0.10096
    183A -> 185A      -0.11225
    180B -> 183B       0.64168
    181B -> 182B       0.72608
 This state for optimization and/or second-order correction.
 Total Energy, E(TD-HF/TD-KS) =  -2356.56511946
 Copying the excited state density for this state as the 1-particle RhoCI
density.

 Excited State 2:  3.025-A 2.0634 eV  600.88 nm  f=0.0001  <S**2>=2.038
    182A -> 185A      -0.10938
    180B -> 182B       0.67471
    181B -> 183B       0.69202

 Excited State 3:  3.041-A 2.5065 eV  494.66 nm  f=0.0024  <S**2>=2.062
    177B -> 183B      -0.10758
    180B -> 182B       0.70730
    181B -> 183B      -0.68605

 Excited State 4:  3.035-A 2.5121 eV  493.55 nm  f=0.0013  <S**2>=2.053
    180B -> 183B       0.74092
    181B -> 182B      -0.65669

 Excited State 5:  3.065-A 2.5715 eV  482.15 nm  f=0.1023  <S**2>=2.098
    182A -> 184A       0.12339
    183A -> 185A      -0.12387
    170B -> 182B      -0.28032
    171B -> 183B       0.25457
    176B -> 183B      -0.17423
    177B -> 182B       0.16693
    178B -> 183B       0.58121
    179B -> 182B       0.61151

 Excited State 6:  3.065-A 2.5722 eV  482.03 nm  f=0.0254  <S**2>=2.098
    182A -> 185A      -0.11412
    183A -> 184A       0.11782
    170B -> 183B      -0.27488
    171B -> 182B       0.26790
    176B -> 182B      -0.19323
    177B -> 183B       0.17345
    178B -> 182B       0.60320
    179B -> 183B       0.57911
    181B -> 183B      -0.10673

 Excited State 7:  3.068-A 2.7622 eV  448.86 nm  f=0.0034  <S**2>=2.103
    182A -> 185A      -0.18493
    183A -> 184A       0.23193
    170B -> 183B       0.43684
    171B -> 182B      -0.43086
    173B -> 182B      -0.10880
    176B -> 182B       0.35211
    177B -> 183B      -0.40482
    178B -> 182B       0.30943
    179B -> 183B       0.26358

 Excited State 8:  3.054-A 2.7644 eV  448.50 nm  f=0.0825  <S**2>=2.082
    182A -> 184A       0.16391
    183A -> 185A      -0.17103
    170B -> 182B       0.48720
    171B -> 183B      -0.44876
```

```
    173B -> 183B      -0.11466
    176B -> 183B       0.34181
    177B -> 182B      -0.38856
    178B -> 183B       0.29134
    179B -> 182B       0.28624
```

1′-Np-OSS

```
 Excited State 1:  1.479-A 1.8150 eV  683.10 nm  f=0.0001  <S**2>=0.297
    181A -> 183A       0.16429
    182A -> 183A       0.67882
    181B -> 183B       0.16429
    182B -> 183B       0.67882
 This state for optimization and/or second-order correction.
 Total Energy, E(TD-HF/TD-KS) =  -2356.57393026
 Copying the excited state density for this state as the 1-particle RhoCI
density.

 Excited State 2:  1.645-A 1.8789 eV  659.87 nm  f=0.3611  <S**2>=0.427
    181A -> 183A      -0.32088
    182A -> 183A      -0.62047
    181B -> 183B       0.32088
    182B -> 183B       0.62047

 Excited State 3:  2.165-A 2.1102 eV  587.53 nm  f=0.0080  <S**2>=0.921
    180A -> 183A       0.15751
    181A -> 183A       0.64685
    182A -> 183A      -0.15871
    182A -> 184A       0.10899
    180B -> 183B       0.15744
    181B -> 183B       0.64633
    182B -> 183B      -0.15842
    182B -> 184B       0.10905

 Excited State 4:  2.035-A 2.1124 eV  586.94 nm  f=0.0165  <S**2>=0.785
    181A -> 183A      -0.60103
    182A -> 183A       0.33110
    181B -> 183B       0.60159
    182B -> 183B      -0.33123

 Excited State 5:  2.327-A 2.5717 eV  482.12 nm  f=0.1029  <S**2>=1.103
    172A -> 183A       0.21318
    174A -> 183A      -0.10191
    179A -> 183A       0.60990
    182A -> 184A      -0.14885
    172B -> 183B       0.21313
    174B -> 183B      -0.10188
    179B -> 183B       0.60973
    182B -> 184B       0.14889

 Excited State 6:  2.317-A 2.5753 eV  481.43 nm  f=0.0160  <S**2>=1.092
    172A -> 183A      -0.21995
    174A -> 183A       0.10589
    179A -> 183A      -0.61318
    182A -> 184A      -0.13864
    172B -> 183B       0.22001
    174B -> 183B      -0.10592
    179B -> 183B       0.61334
    182B -> 184B      -0.13860
```

```
 Excited State 7:  2.348-A 2.7309 eV  454.01 nm  f=0.0036  <S**2>=1.128
    172A -> 183A       0.11399
    177A -> 183A       0.17006
    178A -> 183A       0.13287
    179A -> 183A      -0.12398
    180A -> 183A       0.61094
    181A -> 183A      -0.13226
    172B -> 183B      -0.11399
    177B -> 183B       0.17006
    178B -> 183B      -0.13287
    179B -> 183B       0.12397
    180B -> 183B       0.61093
    181B -> 183B      -0.13226

 Excited State 8:  2.263-A 2.7669 eV  448.10 nm  f=0.0538  <S**2>=1.030
    177A -> 183A      -0.14766
    179A -> 183A       0.14634
    180A -> 183A      -0.65585
    177B -> 183B       0.14766
    179B -> 183B       0.14634
    180B -> 183B       0.65586

 Excited State 9:  2.313-A 2.7869 eV  444.88 nm  f=0.0377  <S**2>=1.088
    170A -> 183A       0.10561
    171A -> 183A       0.18093
    172A -> 183A       0.46990
    173A -> 183A       0.11866
    174A -> 183A      -0.23635
    176A -> 183A       0.10221
    177A -> 183A       0.20632
    179A -> 183A      -0.24243
    180A -> 183A      -0.12965
    170B -> 183B       0.10560
    171B -> 183B      -0.18089
    172B -> 183B       0.46985
    173B -> 183B       0.11864
    174B -> 183B      -0.23632
    176B -> 183B      -0.10220
    177B -> 183B      -0.20630
    179B -> 183B      -0.24241
    180B -> 183B       0.12962
```

2′-C

```
Excited State 1:  2.029-B  2.1841 eV  567.66 nm  f=0.1378  <S**2>=0.779
    102A ->103A       -0.16339
     95B ->102B        0.10492
     99B ->102B       -0.13726
    101B ->102B        0.95310
 This state for optimization and/or second-order correction.
 Total Energy, E(TD-HF/TD-KS) =  -1294.37040665
 Copying the excited state density for this state as the 1-particle RhoCI
density.

 Excited State 2:  2.036-B 2.6747 eV  463.55 nm  f=0.0112  <S**2>=0.786
    100A ->103A       -0.13364
    102A ->103A        0.73953
     95B ->102B        0.34593
     99B ->102B        0.48800
    101B ->102B        0.15870

 Excited State 3:  2.128-A 2.7161 eV  456.47 nm  f=0.0757  <S**2>=0.882
```

```
    102A ->108A        0.11354
    100B ->102B        0.96780

 Excited State 4:  2.046-B 2.8171 eV  440.11 nm  f=0.0106  <S**2>=0.797
    102A ->103A       -0.41544
     92B ->102B       -0.14009
     95B ->102B        0.82071
     97B ->102B        0.25882
    101B ->102B       -0.14918

 Excited State 5:  2.050-B 3.1148 eV  398.05 nm  f=0.0280  <S**2>=0.801
    102A ->103A       -0.43516
     95B ->102B       -0.25305
     99B ->102B        0.83858

 Excited State 6:  3.365-A 3.3529 eV  369.78 nm  f=0.0025  <S**2>=2.581
     98A ->107A        0.24942
     99A ->103A        0.44082
    101A ->103A       -0.36363
     98B ->102B       -0.25940
     98B ->107B       -0.24772
     99B ->103B       -0.40365
     99B ->104B        0.10892
    100B ->102B       -0.12895
    101B ->103B        0.47927

 Excited State 7:  2.079-B 3.5274 eV  351.49 nm  f=0.0016  <S**2>=0.830
    102A ->106A       -0.12938
     95B ->102B       -0.27856
     97B ->102B        0.93656
```

2′-N

```
Excited State 1:  2.014-A  2.0690 eV  599.24 nm  f=0.0673  <S**2>=0.764
    102A ->103A        0.15138
     94B ->102B       -0.10492
    101B ->102B        0.96640
 This state for optimization and/or second-order correction.
 Total Energy, E(TD-HF/TD-KS) =  -1294.37544072
 Copying the excited state density for this state as the 1-particle RhoCI
density.

 Excited State 2:  2.066-A 2.5973 eV  477.36 nm  f=0.0566  <S**2>=0.817
    102A ->103A        0.24306
     95B ->102B        0.40266
     99B ->102B        0.19733
    100B ->102B        0.83432

 Excited State 3:  2.078-A 2.7884 eV  444.65 nm  f=0.0350  <S**2>=0.830
    102A ->103A       -0.13132
     93B ->102B        0.13030
     95B ->102B        0.72737
     96B ->102B       -0.12471
     98B ->102B        0.10765
     99B ->102B        0.42867
    100B ->102B       -0.42253

 Excited State 4:  2.080-A 2.9669 eV  417.89 nm  f=0.0090  <S**2>=0.831
    101A ->103A       -0.11836
    102A ->103A        0.87107
    102A ->104A       -0.10915
```

```
    102A ->109A         0.11769
     95B ->102B        -0.11985
     99B ->102B         0.23418
    100B ->102B        -0.26558
    101B ->102B        -0.12257

 Excited State  5:   2.424-A 3.1296 eV  396.16 nm  f=0.0423  <S**2>=1.219
    101A ->103A        -0.24175
    102A ->103A        -0.27438
    102A ->104A        -0.13125
     95B ->102B        -0.40378
     98B ->102B        -0.10916
     99B ->102B         0.68863
     99B ->104B         0.10211
    100B ->102B         0.15778
    101B ->102B         0.10058
    101B ->103B         0.24330

 Excited State  6:   3.256-A 3.2791 eV  378.10 nm  f=0.0087  <S**2>=2.400
     97A ->107A         0.14695
     99A ->107A        -0.11701
    100A ->103A        -0.16995
    101A ->103A        -0.50991
    101A ->104A        -0.14809
     95B ->102B         0.12614
     97B ->102B         0.14533
     97B ->107B        -0.16036
     99B ->102B        -0.34041
    100B ->103B         0.18123
    101B ->103B         0.54110
    101B ->104B         0.18865

 Excited State  7:   2.093-A 3.4576 eV  358.58 nm  f=0.0022  <S**2>=0.845
    102A ->106A        -0.10094
     95B ->102B        -0.16568
     98B ->102B         0.96469
```

f) contours of MOs involved in low energy excitation in model 1' and 2'.

FMO contours and energies relevant to low energy electronic excitation were obtained at the UB3LYP/6-311+G(2d,p) // UB3LYP/6-31G(2d,p) level of theory in CH_2Cl_2 dielectric medium and are shown in Figure S70.

1'-Cm-T

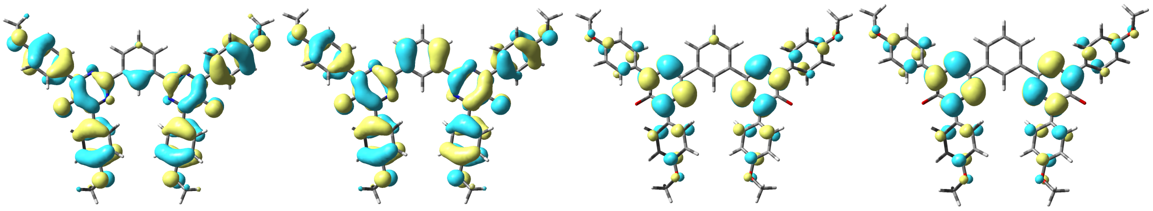

β-HOMO-1 E_{MO} = -5.812 eV

β-HOMO E_{MO} = -5.748 eV

β-LUMO E_{MO} = -2.762 eV

β-LUMO+1 E_{MO} = -2.752 eV

1'-Cp-OSS

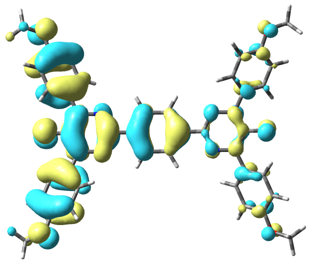

α-HOMO-1
E_{MO} = -5.751 eV

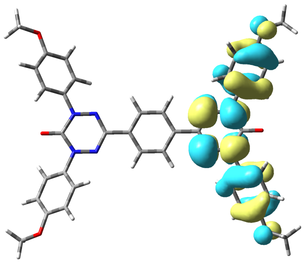

α-HOMO
E_{MO} = -5.021 eV

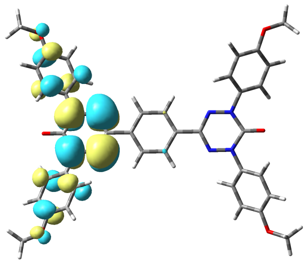

α-LUMO
E_{MO} = -2.755 eV

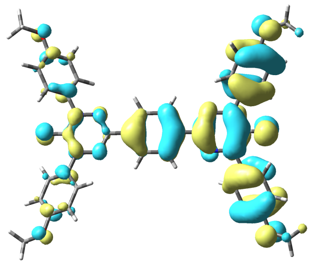

β-HOMO-1
E_{MO} = -5.751 eV

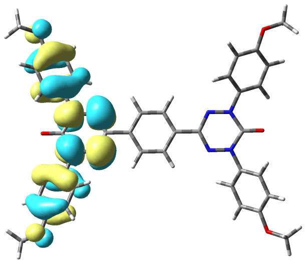

β-HOMO
E_{MO} = -5.021 eV

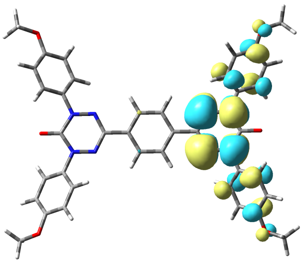

β-LUMO
E_{MO} = -2.755 eV

1'-Nm-T

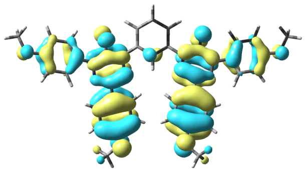

β-HOMO-1
E_{MO} = -5.699 eV

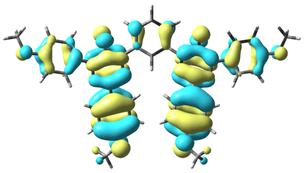

β-HOMO
E_{MO} = -5.671 eV

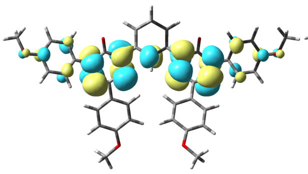

β-LUMO
E_{MO} = -2.806 eV

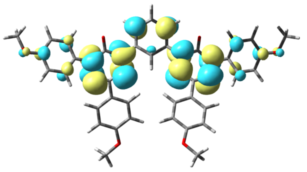

β-LUMO+1
E_{MO} = -2.793 eV

1'-Np-OSS

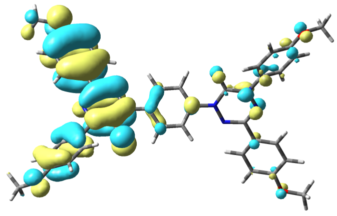

α-HOMO-1
E_{MO} = -5.693 eV

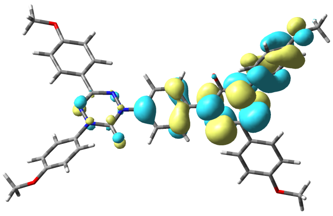

α-HOMO
E_{MO} = -5.103 eV

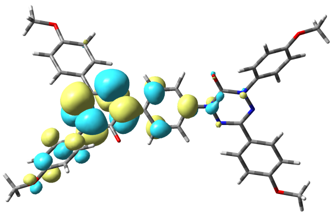

α-LUMO
E_{MO} = -2.813 eV

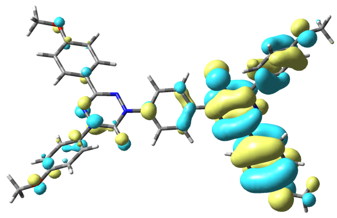

β-HOMO-1
E_{MO} = -5.693 eV

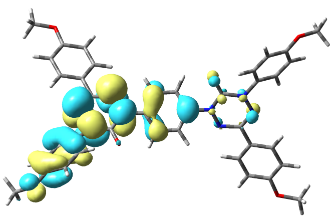

β-HOMO
E_{MO} = -5.103 eV

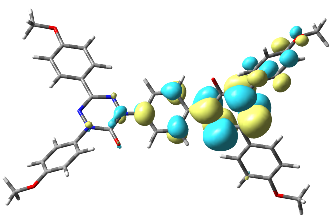

β-LUMO
E_{MO} = -2.813 eV

2'-C

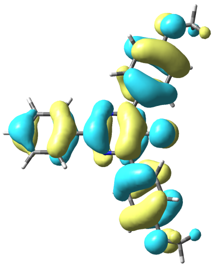

β-HOMO
E_{MO} = -6.174 eV

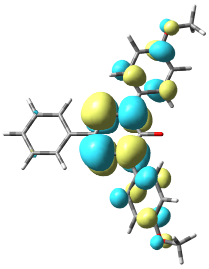

β-LUMO
E_{MO} = -2.740 eV

2'-N

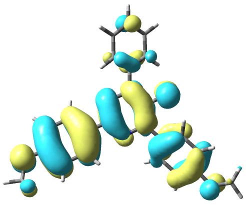

β-HOMO
E_{MO} = -5.668 eV

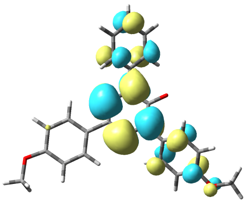

β-LUMO
E_{MO} = -2.759 eV

Figure S70. FMO contours and energies obtained at the UB3LYP/6-311+G(2d,p) // UB3LYP/6-31G(2d,p) level of theory in CH_2Cl_2 dielectric medium.

9. Archive for DFT calculations for model compounds (full geometry optimization)

1'-Cp-OSS

1\1\GINC-LOCALHOST\FOpt\UB3LYP\6-31G(2d,p)\C38H32N8O6\PIOTR\18-Dec-201
6\0\\#P UB3LYP/6-31G(2d,p) Fopt(tight) guess(mix, always) #P geom(noan
gle, nodistance) fcheck freq(noraman, readIso)\\1,4-Bis-[1,5-bis(4-MeO
phenyl)-6-oxoverdazl-3-yl]benzene, C1\\0,1\O,6.8356438507,0.0000039178
,0.0000003781\C,5.6218474266,0.0000019834,0.0000001507\N,4.8542797083,
-1.1655285946,-0.0874243935\N,3.4912981763,-1.1823934713,-0.1073267738
\C,2.8912017883,-0.0000022851,0.0000001942\N,3.4912944794,1.1823913856
,0.107327405\N,4.8542763941,1.1655305014,0.0874250668\C,5.4726579389,2
.4458822535,0.2606331485\C,6.6525446092,2.7921674894,-0.3956800891\C,7
.2028403647,4.0613092266,-0.2268375084\C,6.5733322828,4.9967346577,0.5
978594306\C,5.385451128,4.6457513123,1.2517602935\C,4.8410073641,3.384
7361369,1.0856332965\C,1.4084428297,-0.0000036533,-0.000000061\C,0.693
8406086,1.2051992007,-0.0005556326\C,-0.6938397899,1.2051986131,0.0005
562898\C,-1.4084410221,-0.0000048536,-0.0000003016\C,-0.6938387839,-1.
2052074497,-0.0005567669\C,0.6938415793,-1.2052068621,0.0005553327\C,5
.4726649327,-2.4458782391,-0.2606323094\C,4.8410173454,-3.3847336483,-
1.0856324786\C,5.3854645957,-4.6457471757,-1.2517596601\C,6.5733464679
,-4.9967274233,-0.5978584697\C,7.2028514649,-4.0613004256,0.2268390952
\C,6.6525519979,-2.7921602952,0.3956815297\O,7.0261586502,-6.257642520
6,-0.8291102106\O,7.0261407826,6.2576509959,0.8291110228\C,-2.89119997
89,-0.0000047814,-0.0000003913\C,-5.6218456137,-0.0000028963,0.0000001
632\N,-3.4912954452,-1.1823963789,0.1073254806\N,-3.4912935904,1.18238
8724,-0.1073260236\N,-4.8542756318,1.1655265561,-0.08742326\N,-4.85427
68476,-1.1655327412,0.0874235144\O,-6.8356420376,-0.0000022482,0.00000
03995\C,-5.4726581759,2.4458781919,-0.2606294569\C,-4.841008405,3.3847

335444,-1.0856286657\C,-6.6525448038,2.7921619559,0.3956847703\C,-5.38
54530669,4.6457485937,-1.2517538895\C,-7.2028413751,4.0613035536,0.226
8438713\C,-6.5733342408,4.9967303418,-0.5978522243\C,-5.472661086,-2.4
458829021,0.2606302836\C,-4.8410127951,-3.3847387556,1.0856292877\C,-6
.6525481083,-2.7921649114,-0.3956835208\C,-5.3854591716,-4.6457527926,
1.2517552675\C,-7.2028467815,-4.0613055679,-0.2268424053\C,-6.57334095
02,-4.996733116,0.5978539255\O,-7.0261522783,-6.257648798,0.8291043768
\O,-7.0261436411,6.2576466269,-0.8291021206\C,8.2225786132,-6.66529409
63,-0.1889561794\C,8.2225597154,6.6653059207,0.188957249\C,-8.22256259
1,6.6653000864,-0.1889474625\C,-8.2225720594,-6.6653003797,0.188949992
4\H,3.9198648823,3.1146620724,1.585187487\H,4.9081482481,5.3797031627,
1.8908402867\H,8.1191359128,4.3048085117,-0.7484412447\H,7.1489524405,
2.0758390949,-1.0331299774\H,1.237791623,-2.1415168648,0.0043175659\H,
-1.237788033,-2.1415178822,-0.0043198042\H,-1.2377897691,2.1415088659,
0.0043192695\H,1.2377897979,2.1415098889,-0.0043177961\H,7.1489574916,
-2.0758308259,1.0331319548\H,8.1191473894,-4.3047972765,0.7484433457\H
,4.9081640284,-5.3797002343,-1.8908399495\H,3.919874363,-3.1146621573,
-1.5851868005\H,-3.9198658797,3.1146606295,-1.5851834958\H,-7.14895186
59,2.0758325062,1.0331340676\H,-4.9081508498,5.3797015192,-1.890833135
9\H,-8.1191368347,4.3048016324,0.7484482977\H,-3.9198699123,-3.1146672
742,1.5851837014\H,-7.1489542838,-2.0758350295,-1.0331329562\H,-4.9081
580167,-5.3797062573,1.8908346584\H,-8.1191427166,-4.3048024171,-0.748
4466637\H,8.3992806197,-7.6936595472,-0.5070362018\H,9.0739579673,-6.0
424740944,-0.4904912103\H,8.1253726144,-6.6368619034,0.9034635744\H,8.
3992585989,7.6936719787,0.5070370581\H,9.0739408428,6.0424885396,0.490
4926587\H,8.1253541252,6.6368731813,-0.903462526\H,-8.1253566405,6.636
8660824,0.9034722426\H,-8.3992621847,7.6936664246,-0.5070259633\H,-9.0
739434581,6.0424825855,-0.490483341\H,-8.1253661629,-6.6368668731,-0.9
034697416\H,-8.3992733905,-7.6936663054,0.5070288634\H,-9.0739517706,-
6.0424812402,0.4904858154\\Version=ES64L-G09RevD.01\State=1-A\HF=-2356
.6228027\S2=1.04976\S2-1=0.\S2A=0.400247\RMSD=3.897e-09\RMSF=7.375e-08
\Dipole=0.,-0.0000053,0.0000004\Quadrupole=23.8751228,6.6243815,-30.49
95043,-0.0000117,-0.0000004,0.0000227\PG=C01 [X(C38H32N8O6)]\\@

1′-Cp-T

1\1\GINC-LOCALHOST\FOpt\UB3LYP\6-31G(2d,p)\C38H32N8O6(3)\PIOTR\17-Dec-
2016\0\\#P UB3LYP/6-31G(2d,p) Fopt(tight) #P geom(noangle, nodistance)
fcheck freq(noraman, readIso)\\1,4-Bis-[1,5-bis(4-MeOphenyl)-6-oxover
dazl-3-yl]benzene, C1\\0,3\O,6.836222427,0.0000027788,0.0000005495\C,5
.6223814997,0.0000028257,0.0000001743\N,4.8547434546,-1.1650005773,-0.
0937755369\N,3.4916980055,-1.181543428,-0.1140595084\C,2.8919775238,0.
0000031018,0.0000000489\N,3.4916984473,1.1815490811,0.114059762\N,4.85
47435089,1.165006042,0.0937762664\C,5.4728727945,2.4444656412,0.273836
4571\C,6.6536040479,2.7940142964,-0.3793033654\C,7.2034911755,4.062424
1371,-0.2037807608\C,6.5728217105,4.99387167,0.6245257713\C,5.38415772
09,4.6396355125,1.2752486179\C,4.8400462168,3.3793943028,1.1024526683\
C,1.4082361374,0.0000028707,-0.0000002437\C,0.6939684127,1.2050179887,
-0.0006040084\C,-0.6939675872,1.2050173891,0.0006040275\C,-1.408234272
8,0.0000016558,-0.000000805\C,-0.6939665853,-1.2050136712,-0.000605358
3\C,0.6939694892,-1.2050130716,0.0006032772\C,5.4728724366,-2.44446076
94,-0.2738351586\C,4.8400457068,-3.3793896155,-1.1024515041\C,5.384157
1057,-4.6396310715,-1.2752468518\C,6.5728209286,-4.9938672239,-0.62452
36559\C,7.2034904512,-4.0624196693,0.2037828095\C,6.6536036343,-2.7940
096073,0.3793051486\O,7.0251507683,-6.2537166248,-0.8623608597\O,7.025
1517924,6.2537210254,0.8623634114\C,-2.8919756579,0.0000005777,-0.0000
010209\C,-5.6223796283,-0.0000020093,-0.0000011813\N,-3.4916951289,-1.
1815465478,0.1140574096\N,-3.4916975908,1.1815462286,-0.1140591415\N,-
4.8547426675,1.1650020603,-0.0937754128\N,-4.8547405619,-1.1650047757,
0.0937736757\O,-6.8362205575,-0.0000032923,-0.0000011482\C,-5.47287290
22,2.4444615512,-0.2738332761\C,-4.8400469281,3.3793921967,-1.10244771
75\C,-6.6536044383,2.7940082524,0.37930718\C,-5.384159305,4.6396333192

,-1.2752414112\C,-7.2034923852,4.0624180661,0.2037868664\C,-6.57282353
68,4.9938675126,-0.6245179806\C,-5.4728686396,-2.4444654814,0.27383256
8\C,-4.8400414028,-3.3793943345,1.1024485109\C,-6.6535995366,-2.794014
6626,-0.3793079947\C,-5.3841520087,-4.6396362574,1.2752431155\C,-7.203
4856122,-4.0624251301,-0.2037863829\C,-6.5728155765,-4.993872762,0.624
5196052\O,-7.0251446993,-6.2537225781,0.8623560936\O,-7.0251544378,6.2
537169862,-0.8623533239\C,8.2224422747,-6.6645474016,-0.2258716007\C,8
.2224435304,6.6645516105,0.225874395\C,-8.222446392,6.664545729,-0.22
58635118\C,-8.2224359141,-6.6645536935,0.2258664749\H,3.918287499,3.10
68637685,1.5995059618\H,4.905965772,5.3704871428,1.9172119878\H,8.1204
352249,4.3084968704,-0.7230386005\H,7.1509233049,2.0807924565,-1.01949
57413\H,1.237924865,-2.141333255,0.004348083\H,-1.2379211598,-2.141334
3153,-0.004350891\H,-1.2379230025,2.1413373791,0.0043494318\H,1.237923
033,2.1413384444,-0.0043485108\H,7.1509228796,-2.0807876009,1.01949744
89\H,8.1204343214,-4.308492446,0.7230409012\H,4.9059651367,-5.37048276
43,-1.9172101513\H,3.9182870656,-3.1068588915,-1.5995051897\H,-3.91828
80325,3.106863186,-1.5995015042\H,-7.1509232442,2.0807849491,1.0194982
82\H,-4.9059678419,5.3704864246,-1.9172034517\H,-8.1204365978,4.308489
2225,0.7230451667\H,-3.918282949,-3.1068633073,1.5995023938\H,-7.15091
91581,-2.0807925807,-1.0194999133\H,-4.905959625,-5.3704880174,1.91720
60407\H,-8.1204292978,-4.3084982044,-0.7230446551\H,8.3986179715,-7.69
13713008,-0.5491787655\H,9.0734336074,-6.0403273587,-0.5255953542\H,8.
1267772588,-6.6414076241,0.8668072862\H,8.3986193765,7.6913754373,0.54
91817164\H,9.0734346461,6.0403313155,0.5255982659\H,8.1267786774,6.641
4119469,-0.8668045101\H,-8.1267814966,6.6414040208,0.8668153505\H,-8.3
986228618,7.6913699992,-0.5491689028\H,-9.0734371527,6.0403254366,-0.5
255885104\H,-8.1267707775,-6.6414132874,-0.8668123856\H,-8.3986110644,
-7.6913778617,0.5491730888\H,-9.0734276263,-6.0403342821,0.5255904683\
\Version=ES64L-G09RevD.01\State=3-A\HF=-2356.6225181\S2=2.039967\S2-1=
0.\S2A=2.000868\RMSD=7.029e-09\RMSF=2.507e-07\Dipole=0.,-0.0000226,0.0
00012\Quadrupole=23.9193915,6.6983493,-30.6177408,0.0000188,-0.0000335
,0.0000197\PG=C01 [X(C38H32N8O6)]\\@

1′-Cp-CS

1\1\GINC-LOCALHOST\FOpt\RB3LYP\6-31G(2d,p)\C38H32N8O6\PIOTR\01-Jun-202
3\0\\#P B3LYP/6-31G(2d,p) Fopt(tight) #P geom(noangle, nodistance) fch
eck freq(noraman, readIso)\\1,4-Bis-[1,5-bis(4-MeOphenyl)-6-oxoverdazl
-3-yl]benzene, C1\\0,1\O,6.8299474894,0.0000020061,-0.0000000352\C,5.6
154781816,0.0000018753,0.0000001379\N,4.8488481691,-1.1687711266,-0.07
22427645\N,3.4832666574,-1.1839651958,-0.0900658006\C,2.8892348511,0.0
000013411,0.000000214\N,3.4832659671,1.1839684514,0.0900661695\N,4.848
847722,1.1687747366,0.0722427814\C,5.4616447643,2.4468934896,0.2283029
609\C,6.6909067985,2.7675295291,-0.354037904\C,7.2279459809,4.04355888
41,-0.204629258\C,6.5429473772,5.0163173176,0.5280836959\C,5.308868474
1,4.6937171396,1.1082567263\C,4.7733226295,3.4283510288,0.9588868404\C
,1.4066059826,0.0000009998,0.0000003895\C,0.6944134201,1.2054088056,-0
.0000487983\C,-0.6944139313,1.2054084405,0.0000508182\C,-1.4066057785,
0.0000002651,0.000000691\C,-0.6944132071,-1.2054075214,-0.0000495615\C
,0.6944141346,-1.2054071575,0.0000497298\C,5.4616459615,-2.4468894393,
-0.2283026555\C,4.7733245996,-3.4283478045,-0.9588859634\C,5.308871164
7,-4.693713622,-1.1082551105\C,6.5429501714,-5.0163127475,-0.528081842
5\C,7.2279482393,-4.0435534048,0.2046303296\C,6.6909082336,-2.76752431
84,0.3540381311\O,6.9807183154,-6.2857433657,-0.7344753176\O,6.9807147
915,6.2857480442,0.7344780411\C,-2.8892346405,-0.000000174,0.000000964
\C,-5.6154779724,-0.0000009251,0.0000035901\N,-3.4832655502,-1.1839674
449,0.0900657454\N,-3.4832666243,1.1839663801,-0.0900635711\N,-4.84884
81152,1.1687722112,-0.0722392442\N,-4.8488473055,-1.1687738954,0.07224
33012\O,-6.829947276,-0.0000011534,0.0000030309\C,-5.4616457956,2.4468
906582,-0.2282985426\C,-4.7733252716,3.4283482962,-0.9588836053\C,-6.6
909068824,2.7675264873,0.3540442959\C,-5.3088716342,4.6937142089,-1.10
82527966\C,-7.2279466372,4.0435557024,0.2046365454\C,-6.5429495411,5.0

163142416,-0.5280776352\C,-5.4616434762,-2.4468933908,0.2283010499\C,-
4.7733196244,-3.4283520729,0.958881776\C,-6.6909055725,-2.7675294246,-
0.3540397923\C,-5.3088638935,-4.6937191713,1.1082488592\C,-7.227943225
7,-4.0435597281,-0.2046338149\C,-6.542942946,-5.0163192121,0.528076131
8\O,-6.9807087121,-6.2857509935,0.7344674933\O,-6.9807176076,6.2857448
648,-0.7344713076\C,8.2225172081,-6.6655709466,-0.1668956995\C,8.22251
35507,6.6655767648,0.1668988789\C,-8.222515318,6.6655733308,-0.1668896
877\C,-8.22250746,-6.6655796228,0.1668882578\H,3.8187333177,3.18053718
45,1.402692819\H,4.7887126288,5.4550011641,1.6782876745\H,8.1795609337
,4.2639735699,-0.6703741075\H,7.2307601715,2.02690314,-0.9230510469\H,
1.2397168861,-2.14071276,0.0048297909\H,-1.2397154754,-2.1407134677,-0
.0048302077\H,-1.2397166857,2.1407140408,0.0048317208\H,1.2397156868,2
.1407147593,-0.0048289185\H,7.2307612122,-2.0268972463,0.9230507284\H,
8.179563386,-4.26396722,0.6703752245\H,4.7887157996,-5.4549983327,-1.6
782855517\H,3.818735219,-3.1805349219,-1.40269206\H,-3.8187367897,3.18
05346875,-1.4026912488\H,-7.2307591223,2.0269001322,0.9230584765\H,-4.
7887169857,5.4549982875,-1.6782847608\H,-8.1795608385,4.2639702463,0.6
703830249\H,-3.8187300909,-3.1805383643,1.402687383\H,-7.2307601431,-2
.0269023646,-0.9230508959\H,-4.7887066322,-5.4550041158,1.6782772703\H
,-8.1795582679,-4.2639743567,-0.6703785248\H,8.3781036847,-7.706855800
8,-0.4520679954\H,9.0470871632,-6.0564582789,-0.5574105832\H,8.2054517
769,-6.5891638706,0.9273163523\H,8.3780993418,7.7068615286,0.452071890
9\H,9.0470838174,6.0564643546,0.5574135066\H,8.2054483559,6.5891703995
,-0.9273132295\H,-8.2054478741,6.5891671106,0.9273223953\H,-8.37810193
57,7.7068580185,-0.452062521\H,-9.0470862193,6.0564606547,-0.557402557
5\H,-8.2054431998,-6.5891702868,-0.9273236537\H,-8.3780918326,-7.70686
53307,0.4520585839\H,-9.0470781245,-6.0564692127,0.557405163\\Version=
ES64L-G09RevD.01\State=1-A\HF=-2356.5873235\RMSD=3.917e-09\RMSF=3.608e
-07\Dipole=0.0000016,-0.0000008,0.0000019\Quadrupole=28.7920736,5.9976
534,-34.789727,0.0000279,-0.0000174,0.0000339\PG=C01 [X(C38H32N8O6)]\\

1′-Cm-OSS

1\1\GINC-LOCALHOST\FOpt\UB3LYP\6-31G(2d,p)\C38H32N8O6\PIOTR\14-Dec-201
6\0\\#P UB3LYP/6-31G(2d,p) Fopt(tight) guess(mix, always) #P geom(noan
gle, nodistance) fcheck freq(noraman, readIso)\\1,3-Bis-[1,5-bis(4-MeO
phenyl)-6-oxoverdazl-3-yl]benzene, OS singlet\\0,1\O,5.85624651,0.6976
47419,-0.0095782328\C,4.7998137908,0.1001688668,-0.0241179597\N,3.5557
67093,0.7351535171,-0.0900641643\N,2.365034764,0.0725802935,-0.0884377
51\C,2.4300049001,-1.2545315533,-0.0520901739\N,3.5368798903,-1.989639
923,-0.0093805441\N,4.7103103452,-1.2948268978,0.0207689781\C,5.890753
544,-2.105799364,0.0027851794\C,7.0347998207,-1.7644939325,0.735066020
1\C,8.1396309601,-2.5988854695,0.7147683025\C,8.127809213,-3.786053662
7,-0.0279955701\C,6.9839087267,-4.1291682044,-0.7533723499\C,5.8743687
894,-3.2880296275,-0.7339812026\C,1.1361159163,-1.9851272928,-0.064932
4766\C,1.1035687242,-3.3848362054,-0.0817499111\C,-0.1180185565,-4.050
4641358,-0.1051567031\C,-1.3140136661,-3.3394953695,-0.1068497525\C,-1
.2945156369,-1.9397096987,-0.0795088452\C,-0.0664024232,-1.2740489044,
-0.061362546\C,3.4437659465,2.1635609354,-0.0959559501\C,2.3349050212,
2.7459793881,0.5323750492\C,2.1612731524,4.1184472702,0.5128589486\C,3
.0927339738,4.9418587859,-0.1326456308\C,4.1980682767,4.3623930473,-0.
7599217584\C,4.3706627686,2.979540334,-0.7437777011\O,2.8335838583,6.2
758820169,-0.0918841351\O,9.2658753474,-4.5283972078,0.0230871884\C,-2
.560354325,-1.1614963669,-0.0685517704\C,-4.8781689553,0.2805391278,-0
.0524114611\C,-6.0503147399,-1.8814208789,-0.1481645821\C,-6.078299850
8,-3.0860703642,0.5509765996\C,-7.1804636732,-1.4749340801,-0.86864852
57\C,-7.2183422297,-3.8855890306,0.5445138949\C,-8.3155504486,-2.26791
75909,-0.8739951786\C,-8.3482828571,-3.4775190208,-0.1690126792\C,-3.4
464261122,2.2888260804,0.0835926009\C,-2.3163379245,2.8491501923,-0.52
67516683\C,-4.342729202,3.1178626956,0.7573661881\C,-2.0918416432,4.21
29223483,-0.4641074024\C,-4.1188826904,4.4921520741,0.816967654\C,-2.9
924280223,5.0496253574,0.2075877341\N,-3.6937568619,-1.8532543531,-0.1

337430367\N,-2.446133734,0.160480593,0.0095527671\N,-3.6114229003,0.86
64621926,0.0327669349\N,-4.8405403641,-1.1147106445,-0.1412891959\O,-5
.9116594561,0.9170257278,-0.0474853988\O,-2.6838674998,6.373720258,0.2
087206542\O,-9.513119821,-4.1750783073,-0.2427829876\C,-3.563204251,7.
2628969503,0.8741775282\C,3.7457547489,7.1522974528,-0.7294899939\C,9.
3074657435,-5.7376843341,-0.713392013\C,-9.6000409989,-5.4045952531,0.
4552419764\H,4.9836981298,-3.5520047668,-1.2893605943\H,6.9433730927,-
5.039189709,-1.3375914195\H,9.0318144978,-2.3499464666,1.2777242712\H,
7.0587388153,-0.8504634149,1.3096552833\H,-0.0463230865,-0.1939710067,
-0.0443261953\H,-2.2650097033,-3.8567823065,-0.1305187853\H,-0.1381880
52,-5.1353791073,-0.1222700762\H,2.0346715221,-3.9375937343,-0.0749592
882\H,5.2297013009,2.544547936,-1.2314947428\H,4.9328196686,4.97127197
43,-1.27043462\H,1.3070231041,4.5776807602,0.9970690596\H,1.6114820044
,2.1115316806,1.0266692103\H,-5.1983753147,-3.4003297201,1.0972340574\
H,-7.1700873703,-0.5429950789,-1.4141390628\H,-7.2119898844,-4.8144438
62,1.0997464088\H,-9.1975409258,-1.9683813035,-1.4282985458\H,-1.61671
01908,2.2041508937,-1.0412307418\H,-5.2176118687,2.6999360409,1.231657
0669\H,-1.2208467417,4.65512839,-0.9341282678\H,-4.8307904978,5.111522
5211,1.3468582702\H,-4.5669706389,7.2436083851,0.431792399\H,-3.637670
4852,7.0316382206,1.9441096061\H,-3.1351650403,8.2586913474,0.75117128
39\H,3.3549354288,8.1589380319,-0.5749273373\H,4.74784263,7.08179755,-
0.2884987632\H,3.8122267255,6.9522575747,-1.8062193196\H,10.2956254532
,-6.1645780077,-0.5366755251\H,8.5420832883,-6.4454348457,-0.371142604
7\H,9.1779165374,-5.5600813095,-1.7882837169\H,-10.6032772669,-5.78869
72157,0.2657441965\H,-8.8612857913,-6.1291569782,0.0904038537\H,-9.464
6060412,-5.2658943444,1.5351205034\\Version=ES64L-G09RevD.01\State=1-A
\HF=-2356.6216349\S2=1.041219\S2-1=0.\S2A=0.331416\RMSD=5.807e-09\RMSF
=4.224e-07\Dipole=-0.0068274,-0.367228,-0.0057918\Quadrupole=-7.782341
7,29.8407506,-22.0584089,0.9384415,-15.1162527,1.0997786\PG=C01 [X(C38
H32N8O6)]\\

1′-Cm-T

1\1\GINC-LOCALHOST\FOpt\UB3LYP\6-31G(2d,p)\C38H32N8O6(3)\PIOTR\14-Dec-
2016\0\\#P UB3LYP/6-31G(2d,p) Fopt(tight) geom(noangle, nodistance) fc
heck #P freq(noraman, readIso)\\1,3-Bis-[1,5-bis(4-MeOphenyl)-6-oxover
dazl-3-yl]benzene, triplet\\0,3\O,5.8563915578,0.6970248212,-0.0078308
61\C,4.7998145039,0.0998550407,-0.0226423779\N,3.5559399037,0.73521012
83,-0.0877366109\N,2.3650081215,0.0729970942,-0.086163677\C,2.42964348
14,-1.2542951779,-0.0512519172\N,3.5365552263,-1.9897440984,-0.0096651
653\N,4.7100533754,-1.2951937329,0.0210630474\C,5.8904022669,-2.106349
5595,0.0022010286\C,7.034313828,-1.766151517,0.7351735393\C,8.13904740
74,-2.6006571734,0.7140319226\C,8.1272075715,-3.7868626249,-0.03026680
79\C,6.9834228176,-4.1288739404,-0.7563405599\C,5.8739994499,-3.287587
174,-0.736119734\C,1.1361683909,-1.9844752032,-0.0644926848\C,1.103605
4208,-3.3843584535,-0.0812774698\C,-0.1180103281,-4.0499388105,-0.1051
372049\C,-1.3140353552,-3.3390021011,-0.1072878154\C,-1.2945466454,-1.
9390426254,-0.0799164705\C,-0.066389505,-1.2732728093,-0.0613468668\C,
3.4443981193,2.1636930313,-0.0924041976\C,2.3362981285,2.7460155534,0.
5373092158\C,2.1631553661,4.1185690386,0.5189781973\C,3.0943931013,4.9
421256923,-0.1266453726\C,4.1989837655,4.3627367094,-0.7552983232\C,4.
3710437281,2.9798003325,-0.7403840925\O,2.8357844446,6.2762271963,-0.0
846347573\O,9.2651523651,-4.5294490065,0.0201146139\C,-2.5599871088,-1
.1612473484,-0.0693738232\C,-4.8781838159,0.2802718915,-0.0538921773\C
,-6.0499871611,-1.8820031807,-0.1475935804\C,-6.077914948,-3.085713379
,0.5531289626\C,-7.1800447755,-1.4766052728,-0.8687995491\C,-7.2178452
269,-3.8854126442,0.5474919375\C,-8.3150374664,-2.2697349244,-0.873305
2326\C,-8.3477134348,-3.4784258781,-0.1667618301\C,-3.4470525306,2.289
0938483,0.0800449138\C,-2.3177233668,2.8493886229,-0.5316809803\C,-4.3
431008676,3.1182495863,0.7539728399\C,-2.0937093016,4.2133014765,-0.47
02242245\C,-4.1197816352,4.4926810869,0.8123420969\C,-2.9940684186,5.0
501431427,0.2015860202\N,-3.6934394057,-1.8533790898,-0.1334586912\N,-

2.4460936285,0.1609674979,0.0073021856\N,-3.6115957204,0.8665989649,0.
0304448639\N,-4.8402999088,-1.1150780675,-0.1415900536\O,-5.9118304721
,0.9164628998,-0.049247587\O,-2.686042351,6.3743749855,0.2014677954\O,
-9.5124374723,-4.1762570267,-0.2398382809\C,-3.5651667392,7.263668471,
0.8670379378\C,3.7477462071,7.1527657339,-0.7223590426\C,9.3066973438,
-5.7378044573,-0.7178906844\C,-9.5992778806,-5.404892402,0.4597425669\
H,4.9834066067,-3.5507296727,-1.2920280958\H,6.9428818078,-5.038137466
7,-1.3417388163\H,9.0311472995,-2.3525689451,1.2774952149\H,7.05824589
47,-0.8528582302,1.3109468872\H,-0.0463101331,-0.1932045752,-0.0443139
232\H,-2.2650295648,-3.856244498,-0.1313323201\H,-0.1381798911,-5.1348
571106,-0.1222477492\H,2.0347078504,-3.9370831228,-0.0741070419\H,5.22
95133936,2.5448565947,-1.229158758\H,4.9335460934,4.9717341752,-1.2659
426855\H,1.3094528117,4.5777600888,1.0041875879\H,1.6130315537,2.11144
30606,1.0316815043\H,-5.1980347689,-3.3991539331,1.099937646\H,-7.1696
927254,-0.5453644695,-1.4154947712\H,-7.2114564078,-4.8135493171,1.103
9239382\H,-9.1969786427,-1.9710366411,-1.4281386091\H,-1.6182558866,2.
2042689984,-1.046235813\H,-5.2174165367,2.7003215517,1.2293208586\H,-1
.2232593967,4.6555124912,-0.9412432687\H,-4.8314969824,5.1121629368,1.
3423618382\H,-4.5692918149,7.2436985722,0.4254949741\H,-3.6386687833,7
.0331111012,1.9371890496\H,-3.137611842,8.2595441697,0.7430030004\H,3.
357415741,8.1594386424,-0.5667694971\H,4.750169614,7.0815990633,-0.282
235448\H,3.8132754492,6.9534631295,-1.7992838632\H,10.2947443878,-6.16
50947313,-0.5415007655\H,8.5411171044,-6.4458578077,-0.3767096634\H,9.
1774142844,-5.5588190896,-1.7925857722\H,-10.6024178857,-5.7894030701,
0.2705624851\H,-8.8603397447,-6.1297992445,0.0959600363\H,-9.464053028
,-5.2647944382,1.5394676691\\Version=ES64L-G09RevD.01\State=3-A\HF=-23
56.6218121\S2=2.046728\S2-1=0.\S2A=2.001185\RMSD=2.506e-09\RMSF=4.136e
-07\Dipole=-0.0068307,-0.3676239,-0.0058051\Quadrupole=-7.7911419,29.8
230179,-22.0318761,0.9385144,-15.1350777,1.0993116\PG=C01 [X(C38H32N8O
6)]\\@

1′-Cm-CS

1\1\GINC-GAUSIANDELL\FOpt\RB3LYP\6-31G(2d,p)\C38H32N8O6\PKASZYNSKI\01-
Jun-2023\0\\#P B3LYP/6-31G(2d,p) Fopt(tight) #P geom(noangle, nodistan
ce) fcheck freq(noraman, readIso)\\1,3-Bis-[1,5-bis(4-MeOphenyl)-6-oxo
verdazl-3-yl]benzene, CS\\0,1\O,5.8554310363,0.6973638651,0.0271102684
\C,4.7936391715,0.108338221,0.0068052019\N,3.5533083723,0.7485468441,-
0.0301816784\N,2.3566729822,0.0836551588,-0.0377728391\C,2.4255342036,
-1.2416427528,-0.0361535582\N,3.5278106069,-1.9808449841,-0.0203609484
\N,4.7028372869,-1.2921384734,0.0147501192\C,5.8751059249,-2.103150275
3,-0.0420717393\C,7.0653648417,-1.7524386435,0.6142350468\C,8.15865901
48,-2.598741584,0.5578767801\C,8.0971918781,-3.8076471895,-0.147117773
5\C,6.9108322612,-4.1607647413,-0.7969275214\C,5.810749264,-3.31167941
4,-0.7391254203\C,1.1357880387,-1.9788848768,-0.0574092754\C,1.1038911
989,-3.3782583413,-0.0744224413\C,-0.1178980724,-4.0439254535,-0.10501
20822\C,-1.3140894432,-3.3326838015,-0.1138982283\C,-1.2939540969,-1.9
332507231,-0.0867951818\C,-0.0662965944,-1.2682865021,-0.0612610423\C,
3.4404515828,2.1695351891,0.0038702271\C,2.2724504723,2.7283932058,0.5
499108504\C,2.0934527581,4.098746583,0.5701726358\C,3.071977451,4.9519
254965,0.0422365012\C,4.2327341687,4.4003024978,-0.5056763361\C,4.4162
022348,3.0201418826,-0.5260470277\O,2.8004076721,6.2813520696,0.110834
9363\O,9.2297939579,-4.5579888905,-0.1379701326\C,-2.555401166,-1.1482
878594,-0.0840574289\C,-4.8716801104,0.2894415955,-0.0830766875\C,-6.0
346065688,-1.8807738653,-0.1032673167\C,-6.0156118726,-3.1122208842,0.
5553263711\C,-7.2106257467,-1.465571434,-0.7475028036\C,-7.1465204382,
-3.9212404245,0.5869913632\C,-8.3346459526,-2.2720186958,-0.7171972249
\C,-8.3185574044,-3.5039845337,-0.0506767705\C,-3.4428357067,2.2978144
209,-0.0159936104\C,-2.2545540758,2.8298099408,-0.5448383222\C,-4.3866
148938,3.1669739012,0.5410304979\C,-2.0247462047,4.1925229192,-0.52201
97858\C,-4.1519752207,4.5393197422,0.5640465865\C,-2.9712007799,5.0644
170264,0.0331068762\N,-3.6843753035,-1.8451545025,-0.1224868952\N,-2.4

373408671,0.1728264363,-0.0407239043\N,-3.6084379688,0.8816343437,-0.0
266629002\N,-4.8329775294,-1.1124959612,-0.1351945174\O,-5.9108385117,
0.9178634301,-0.0841923319\O,-2.6503772276,6.3843347816,0.0062691518\O
,-9.478254042,-4.2110600271,-0.0827983314\C,-3.5733401165,7.3077137906
,0.5569734137\C,3.757367499,7.1866881684,-0.4110451867\C,9.2202535101,
-5.7912960546,-0.8356749492\C,-9.514958914,-5.4652393253,0.5756974375\
H,4.8895048619,-3.5825067489,-1.2373624519\H,6.8311569553,-5.085894236
4,-1.3528048156\H,9.0820615194,-2.3423083972,1.0643338305\H,7.13000319
54,-0.8251938126,1.1621047723\H,-0.0462225944,-0.1885079587,-0.0442410
712\H,-2.2649446442,-3.849742469,-0.1441502002\H,-0.1380679749,-5.1288
627736,-0.1221134228\H,2.034858102,-3.9309895761,-0.0610331089\H,5.317
2476723,2.6115479261,-0.9547203822\H,5.0046485033,5.0310412584,-0.9269
446453\H,1.1969101762,4.5345765972,0.9962440623\H,1.5137294194,2.07325
13509,0.9543513416\H,-5.1053601215,-3.4326572646,1.0442446413\H,-7.240
4418778,-0.5197644748,-1.2658487711\H,-7.1016037993,-4.8657456597,1.11
33986504\H,-9.247584346,-1.9656202504,-1.2147837811\H,-1.5204647304,2.
1599946083,-0.9701627417\H,-5.3024726142,2.7788418277,0.957144425\H,-1
.1123776555,4.607944074,-0.9346723632\H,-4.9001654113,5.184721025,1.00
54292964\H,-4.5354640513,7.2780733326,0.0307497805\H,-3.741561308,7.12
11014099,1.6248817663\H,-3.1245440652,8.2938879295,0.430894201\H,3.345
4538404,8.1843961416,-0.2536945389\H,4.7174176861,7.1047512221,0.11341
866\H,3.9191623617,7.0276922026,-1.4844013807\H,10.2131274452,-6.22202
26543,-0.6998018409\H,8.4690310233,-6.4791381028,-0.4281690185\H,9.032
623074,-5.6468146557,-1.9068429951\H,-10.5230728144,-5.8542787718,0.42
69014961\H,-8.7895761228,-6.1673407311,0.1462834174\H,-9.3227179848,-5
.3616502843,1.6507759268\\Version=ES64L-G09RevD.01\State=1-A\HF=-2356.
5866008\RMSD=1.020e-09\RMSF=3.550e-07\Dipole=-0.0053661,-0.2887239,-0.
0045519\Quadrupole=-5.0568929,31.1620028,-26.1051098,0.8898297,-13.692
8337,1.1577889\PG=C01 [X(C38H32N8O6)]\\

1′-Np-OSS

1\1\GINC-LOCALHOST\FOpt\UB3LYP\6-31G(2d,p)\C38H32N8O6\PIOTR\21-Dec-201
6\0\\#P UB3LYP/6-31G(2d,p) Fopt(tight) guess(mix, always) #P geom(noan
gle, nodistance) fcheck freq(noraman, readIso)\\1,4-Bis-[3,5-bis(4-MeO
phenyl)-6-oxoverdazl-1-yl]benzene, singlet\\0,1\N,4.873352273,-1.45553
94207,-0.0847091274\N,3.4506401466,0.8592679182,-0.0270929951\C,5.4809
426176,2.126129693,0.0810463676\C,6.8686164052,2.1997626431,-0.0643885
375\C,7.5378167419,3.4195187088,-0.0048315729\C,6.8145053869,4.5974053
603,0.204494133\C,5.4223874015,4.5345222448,0.3521458693\C,4.76757568
46,3.3172069876,0.2907164029\C,1.391116123,-0.269817835,-0.1506367849\
C,0.8227474074,0.846447251,-0.7732607956\C,-0.5540664666,0.9886872888,
-0.8574782502\C,-1.3904312488,0.0096094382,-0.3092830629\C,-0.82206648
28,-1.1005833857,0.3241083433\C,0.5547493811,-1.2457973368,0.403052239
4\C,5.709304558,-2.6184063707,-0.0380318359\C,5.429340722,-3.762885095
8,-0.7951482651\C,6.2848376925,-4.8502460174,-0.7410223005\C,7.4324512
672,-4.8206733616,0.0611317605\C,7.7152311177,-3.6753919559,0.81001063
85\C,6.8532740587,-2.5831482101,0.7562962699\O,8.1987268996,-5.9433968
327,0.0408260094\O,7.3610139318,5.8396079364,0.2799753656\N,-3.4503928
75,-0.469563484,0.7196397394\N,-4.8723207085,0.6783999139,-1.291843005
7\C,-5.7079222963,1.31976482,-2.2632726349\C,-5.4271480998,1.264151021
1,-3.6342246617\C,-6.8523627429,1.9808768407,-1.8227575942\C,-6.282322
8223,1.8728150043,-4.5371990859\C,-7.7139957854,2.5917128833,-2.730127
1058\C,-7.4304102407,2.54356421,-4.0975021389\C,-5.48116822,-1.0331948
289,1.8585129009\C,-4.7683089217,-1.4698293452,2.9866533771\C,-6.86878
13679,-1.1962020135,1.8454710513\C,-5.4235506083,-2.0473425597,4.05975
41209\C,-7.538411376,-1.7765852572,2.9196832867\C,-6.8156031641,-2.206
685908,4.0363637988\O,-7.3625579545,-2.7850166149,5.1380943628\O,-8.19
63091122,3.1068613652,-5.0691985206\C,9.3677894375,-5.968118192,0.840
4592224\C,8.7653085669,5.9627876801,0.1392225175\C,-9.365826759,3.803
5361482,-4.6772738319\C,-8.7668107077,-2.9696592984,5.169864053\N,2.8
148212072,-0.3472547613,-0.0634514606\N,-2.8141615604,0.1238155103,-0.

331262535\N,-5.5380969887,0.1385867443,-0.2324778569\N,5.5386938772,-0
.2694815546,0.0018825229\C,-3.4810765614,0.721634078,-1.4085715303\C,3
.4821615178,-1.5782487983,-0.1089149395\C,-4.7790051557,-0.4162846015,
0.7103310194\C,4.7792399974,0.8241650986,0.0144544794\O,-2.9097724439,
1.2257596999,-2.3531949583\O,2.9112360126,-2.6476215774,-0.1666242023\
H,3.6920259819,3.2742584774,0.4104329367\H,4.8782172224,5.4576311506,0
.5170177371\H,8.6132487336,3.4395299572,-0.1259139621\H,7.4297444095,1
.2884809072,-0.2320791932\H,0.9749975138,-2.114486208,0.8850060244\H,-
1.4693975512,-1.8475147167,0.7623270399\H,-0.9743095575,1.8504521033,-
1.3517101328\H,1.4700684678,1.6081362993,-1.1853102286\H,7.0708698703,
-1.6911643558,1.3293647101\H,8.5937750566,-3.6202029571,1.4395367735\H
,6.0826328195,-5.7428264475,-1.3217715107\H,4.5460345115,-3.8019764869
,-1.4151082841\H,-4.5434702689,0.7541493256,-3.9879480838\H,-7.0705801
141,2.0096630443,-0.7630674899\H,-6.0794916285,1.8377599739,-5.6013827
768\H,-8.5929219274,3.1015730353,-2.3576759164\H,-3.6928148597,-1.3448
092102,3.0124655137\H,-7.4295167931,-0.8683668275,0.9785690513\H,-4.87
97752139,-2.3837151019,4.9353086856\H,-8.6137795001,-1.8909059734,2.87
35363466\H,9.8208949855,-6.9476300751,0.6819140055\H,10.0790566511,-5.
188572314,0.5400303741\H,9.1309116041,-5.8477988011,1.9049206618\H,8.9
854277382,7.027036356,0.2336463293\H,9.2993448845,5.4124979026,0.92381
27413\H,9.1050194089,5.6095148591,-0.8422844032\H,-9.1296040604,4.6522
513392,-4.0234037913\H,-9.8185216839,4.1745425238,-5.5977728609\H,-10.
077172828,3.1428650625,-4.1660592751\H,-9.1058385536,-3.6268847938,4.3
595087317\H,-8.9873295655,-3.4395957412,6.12930385\H,-9.3011228288,-2.
0137046701,5.104620223 4\\Version=ES64L-G09RevD.01\State=1-A\HF=-2356.6
241583\S2=1.018572\S2-1=0.\S2A=0.347532\RMSD=4.719e-09\RMSF=5.908e-07\
Dipole=-0.0002525,0.3810889,0.6757773\Quadrupole=56.3260183,-23.270012
6,-33.0560058,-5.4990276,3.1369208,-8.096222\PG=C01 [X(C38H32N8O6)]\\

1′-Np-T

1\1\GINC-LOCALHOST\FOpt\UB3LYP\6-31G(2d,p)\C38H32N8O6(3)\PIOTR\21-Dec-
2016\0\\#P UB3LYP/6-31G(2d,p) Fopt(tight) geom(noangle, nodistance) fc
heck #P freq(noraman, readIso)\\1,4-Bis-[3,5-bis(4-MeOphenyl)-6-oxover
dazl-1-yl]benzene, triplet\\0,3\N,4.8773262598,-1.4534917181,-0.151469
7598\N,3.4446658065,0.8541339295,-0.0019262523\C,5.4705291762,2.120377
2551,0.1767666627\C,6.8593776581,2.2028200491,0.0483622837\C,7.5251109
374,3.420209076,0.166856192\C,6.7970321871,4.5865271638,0.4195907483\C
,5.4035668344,4.5147832343,0.5500461048\C,4.7522304808,3.300063315,0.4
299979079\C,1.3889964663,-0.2789739591,-0.1795110407\C,0.8188854487,0.
8337238792,-0.802713165\C,-0.5599291723,0.9724169127,-0.8808358602\C,-
1.3882915147,-0.010363183,-0.3320508835\C,-0.8181830129,-1.1192057343,
0.2979871185\C,0.5606310012,-1.2573703599,0.3770475166\C,5.7166733413,
-2.614247189,-0.144115293\C,5.4438026913,-3.7309946905,-0.944317713 7\C
,6.3029031964,-4.8167299463,-0.9267979012\C,7.4474187148,-4.8130062914
,-0.1196745412\C,7.7228310758,-3.6955764446,0.6727364196\C,6.857195358
8,-2.6051219996,0.6561617349\O,8.2182965512,-5.931173333,-0.1793578164
\O,7.3397231214,5.8252702254,0.554934565\N,-3.4444339849,-0.4453686323
,0.7282759264\N,-4.8762522321,0.6202142169,-1.3246340472\C,-5.71522397
05,1.2268345823,-2.3146085766\C,-5.4415214964,1.1200011198,-3.68412718
48\C,-6.8562154613,1.9065569627,-1.8933801134\C,-6.3002771041,1.696496
17,-4.6046563411\C,-7.7215041092,2.4846827574,-2.8184407292\C,-7.44526
43145,2.3848704413,-4.184510153\C,-5.4708137199,-0.948305339,1.9031225
354\C,-4.7530475133,-1.3417699987,3.0440586061\C,-6.8596143307,-1.1012
980811,1.9064263478\C,-5.4048499353,-1.8677801707,4.1452678546\C,-7.52
58137747,-1.6300241196,3.0091071646\C,-6.7982625948,-2.0169940003,4.13
83525306\O,-7.3414363518,-2.5423099115,5.2680964247\O,-8.2157416057,2.
9121104237,-5.1726876219\C,9.386698975,-5.980081047,0.6202023302\C,8.7
452935663,5.957123462,0.4360326186\C,-9.3845942728,3.6212373473,-4.801
5071213\C,-8.7469804249,-2.712743417,5.3185891161\N,2.8162297891,-0.35
45104674,-0.0938687315\N,-2.8155491169,0.1015410533,-0.3532037533\N,-5
.5351523971,0.1302427058,-0.2379280811\N,5.5357553686,-0.269827591,-0.

0080812896\C,-3.4831372806,0.6517707553,-1.4507370973\C,3.4842746847,-
1.5781856172,-0.1905251524\C,-4.7721178561,-0.3861176597,0.7252665535\
C,4.7723323802,0.8213338446,0.0479912621\O,-2.914621883,1.1121164584,-
2.4190207007\O,2.9161704133,-2.6451536088,-0.2979392129\H,3.6755828386
,3.2499078542,0.5365191196\H,4.8556842141,5.4289728014,0.7488343055\H,
8.6016393226,3.4475502516,0.0575060559\H,7.4243115944,1.3006815945,-0.
1525802647\H,0.9870058184,-2.1239155902,0.8585300191\H,-1.4634742643,-
1.8681121353,0.7365813484\H,-0.9862994835,1.8326678341,-1.3734800201\H
,1.4641675134,1.5967551609,-1.2162604016\H,7.069066022,-1.7347750917,1
.2635848057\H,8.598360301,-3.6610963538,1.3078377599\H,6.1062862046,-5
.6876492441,-1.5413817199\H,4.5632942377,-3.7497738352,-1.5691150955\H
,-4.5606425569,0.5953513912,-4.022970225\H,-7.0687248018,1.9759374258,
-0.8344268281\H,-6.1030169308,1.6212791738,-5.6678133056\H,-8.59741480
81,3.010027517,-2.4608272714\H,-3.6764457399,-1.2243117071,3.056879967
7\H,-7.4241370586,-0.8066476838,1.0301401538\H,-4.8573814226,-2.170515
1209,5.0307419668\H,-8.6022873738,-1.7380983333,2.9752991293\H,9.84565
05204,-6.9500856251,0.4243407333\H,10.0936375974,-5.185336683,0.351053
6806\H,9.148264635,-5.9026150166,1.6883141567\H,8.9619735167,7.0163092
814,0.5815075638\H,9.2715005213,5.3723587537,1.2007180993\H,9.09702647
63,5.6497221937,-0.5566238228\H,-9.1468085577,4.4952473299,-4.18241156
52\H,-9.843114414,3.9553855179,-5.7331784441\H,-10.0916354585,2.979465
4908,-4.2610966441\H,-9.0980360426,-3.4032468339,4.5417085544\H,-8.964
0905802,-3.1363759864,6.3001115958\H,-9.2734408249,-1.755981282,5.2135
505714\\Version=ES64L-G09RevD.01\State=3-A\HF=-2356.6231833\S2=2.04330
7\S2-1=0.\S2A=2.000976\RMSD=7.154e-09\RMSF=8.027e-07\Dipole=-0.0002575
,0.4053437,0.7188141\Quadrupole=56.4009956,-23.4737789,-32.9272167,-5.
8941678,3.3598562,-7.8216659\PG=C01 [X(C38H32N8O6)]\\

1′-Np-CS

1\1\GINC-LOCALHOST\FOpt\RB3LYP\6-31G(2d,p)\C38H32N8O6\PIOTR\01-Jun-202
3\0\\#P B3LYP/6-31G(2d,p) Fopt(tight) #P geom(noangle, nodistance) fch
eck freq(noraman, readIso)\\1,4-Bis-[3,5-bis(4-MeOphenyl)-6-oxoverdazl
-1-yl]benzene, CS singlet\\0,1\N,4.8557402383,-1.4423880244,0.34461086
47\N,3.4716954286,0.7657680266,-0.3861053708\C,5.5147219307,1.97466057
71,-0.6866438261\C,6.8947301115,1.9697831628,-0.9040882832\C,7.5771681
554,3.1346361235,-1.2446680212\C,6.8748098065,4.3369022085,-1.37249310
95\C,5.4909551667,4.3532028143,-1.155388291\C,4.8229561108,3.188547693
9,-0.8187289772\C,1.4108142708,-0.2897431349,-0.120001827\C,0.83787996
25,0.8423451526,-0.7533116494\C,-0.5209567004,1.0048809943,-0.86219014
78\C,-1.4101409076,0.0461212388,-0.3104837857\C,-0.8372097083,-1.08140
15287,0.3309219543\C,0.5216366568,-1.2582283671,0.4144589278\C,5.68088
48504,-2.5343398491,0.7701571061\C,5.3827837367,-3.8660179061,0.452324
6556\C,6.2360970611,-4.8781117684,0.8573331766\C,7.3977362481,-4.58916
99199,1.5844330462\C,7.6980335432,-3.2601425726,1.8956474686\C,6.84159
19184,-2.2428204089,1.4851530598\O,8.1590697815,-5.6587508411,1.934928
8875\O,7.4358923306,5.5312390641,-1.6998558872\N,-3.4712126548,-0.7284
093877,0.45386751\N,-4.8549585273,1.0389858407,-1.0584294492\C,-5.6799
917696,1.9678709593,-1.7731354453\C,-5.3812702859,2.3850307825,-3.0769
726977\C,-6.8412074108,2.4284978774,-1.1543909875\C,-6.2344858346,3.25
50121507,-3.7339851546\C,-7.6975526483,3.3058777844,-1.8130407528\C,-7
.3966398778,3.7273298468,-3.1111841027\C,-5.514460037,-1.6117635831,1.
331664771\C,-4.8230145988,-2.3526603183,2.302480823\C,-6.8943406112,-1
.7957633615,1.2141814864\C,-5.4911993731,-3.2435865961,3.1245290974\C,
-7.5769621373,-2.6901515246,2.0343622051\C,-6.8749228428,-3.4213863189
,2.9974441417\O,-7.436206153,-4.3196804155,3.8497640919\O,-8.157825239
,4.5804381857,-3.8455426894\C,9.3427813068,-5.4215631357,2.6766826884\
C,8.8331805411,5.5737219286,-1.9290422329\C,-9.3420431016,5.0920676015
,-3.2594597246\C,-8.8333753823,-4.538238933,3.7667229007\N,2.799532039
7,-0.3616927228,-0.0309917195\N,-2.7988864131,0.1590651157,-0.32680141
13\N,-5.5491084591,0.1825343691,-0.2450469826\N,5.5496982234,-0.302978
0252,0.0329839327\C,-3.4806669443,1.113975396,-1.1481362138\C,3.481476

4778,-1.5584029345,0.3615708035\C,-4.8028838908,-0.6529799554,0.453556
0846\C,4.8033418102,0.7269078094,-0.3209490269\O,-2.9006173368,1.90572
49155,-1.8617943895\O,2.9015818553,-2.5789604188,0.6694953494\H,3.7545
167389,3.209515848,-0.6436239682\H,4.9634458509,5.2951709871,-1.254757
4598\H,8.6458964426,3.0929945493,-1.4110172943\H,7.4378399225,1.037199
5978,-0.8099642063\H,0.9079860621,-2.136048782,0.9011633662\H,-1.49294
29862,-1.8256826177,0.7555007413\H,-0.9073012375,1.8754460272,-1.36176
00924\H,1.4936102701,1.5909912912,-1.1701500713\H,7.0736042638,-1.2108
965383,1.7130421582\H,8.5875959233,-3.0032068053,2.4558400899\H,6.0207
609994,-5.9127373298,0.6164610961\H,4.4895938824,-4.1069060354,-0.1042
007045\H,-4.48767959,2.0337274149,-3.5705489343\H,-7.073689653,2.08949
25422,-0.1535626495\H,-6.0186712703,3.5843003893,-4.743852667\H,-8.587
5229051,3.6520174201,-1.3038322719\H,-3.7546835536,-2.2133189624,2.411
6457084\H,-7.4372000252,-1.2328555457,0.4645294351\H,-4.9639404825,-3.
8158437036,3.8794888614\H,-8.6455803753,-2.8113035354,1.9120367191\H,9
.7912623115,-6.4007148488,2.8496644471\H,10.0492249563,-4.7940735146,2
.1191955389\H,9.1251838959,-4.9481657573,3.6420917179\H,9.0675495784,6
.6111954667,-2.1714649088\H,9.3977633233,5.2746807751,-1.0371213801\H,
9.1239643093,4.9317996157,-2.7699034979\H,-9.1251589732,5.6733203912,-
2.3547085075\H,-9.7903029263,5.7465834279,-4.0081039458\H,-10.04837003
77,4.2901019685,-3.0113646669\H,-9.123463015,-4.9257384684,2.782161874
3\H,-9.0679431499,-5.2825761272,4.5289492839\H,-9.3983761975,-3.620448
9274,3.971997682\\Version=ES64L-G09RevD.01\State=1-A\HF=-2356.6044305\
RMSD=2.151e-09\RMSF=1.280e-07\Dipole=-0.0001726,0.2600115,0.4610635\Qu
adrupole=55.3251501,-22.7261749,-32.5989753,-4.7157132,2.6946506,-8.16
74643\PG=C01 [X(C38H32N8O6)]\\

1′-Nm-OSS

1\1\GINC-LOCALHOST\FOpt\UB3LYP\6-31G(2d,p)\C38H32N8O6\PIOTR\29-May-202
3\0\\#P UB3LYP/6-31G(2d,p) Fopt(tight) guess(mix, always) #P geom(noan
gle, nodistance) fcheck freq(noraman, readIso)\\1,3-Bis-[1,3-bis(4-MeO
phenyl)-6-oxoverdazl-5-yl]benzene, singlet 2nd orient\\0,1\N,-2.199569
8519,-0.3261965636,1.0625101198\N,-4.6632348645,-1.0377564582,0.153048
8734\C,-5.9784684196,-1.2779564733,-0.3593601763\C,-6.7329518244,-0.18
60099924,-0.8051073163\C,-8.0214181932,-0.3728258946,-1.2733621129\C,-
8.5839838534,-1.6550495681,-1.3075843484\C,-7.8324537624,-2.744930549,
-0.8608820765\C,-6.5360023615,-2.5550195391,-0.387463642\C,-1.19920645
79,-2.2747478082,0.1904974313\C,-1.2143880514,-3.6718047138,0.19887401
27\C,-0.0188375589,-4.3576983307,0.0148102976\C,1.1849237717,-3.686795
53,-0.1709798094\C,1.1866511145,-2.2896407296,-0.1661583064\C,-0.00204
7846,-1.5833308128,0.0112803685\C,-3.0934323561,1.7211206549,1.9317676
095\C,-1.8254817942,2.1151523994,2.3891673275\C,-1.6527844106,3.317067
2694,3.0521350523\C,-2.7461327123,4.1633153913,3.2805011768\C,-4.01497
73752,3.7826038393,2.8341546631\C,-4.1772521923,2.5710919878,2.1671382
259\O,-2.4703871341,5.3192877336,3.9399154029\O,-9.8531123187,-1.73224
89371,-1.7886205121\C,5.9776300721,-1.3493617647,0.3812666234\C,6.5196
60701,-2.6330054045,0.412666858\C,6.7452849336,-0.2654876708,0.8242199
786\C,7.8137229298,-2.8373823392,0.8865988634\C,8.031399181,-0.4666867
02,1.2929799673\C,8.5783990367,-1.7555358726,1.330511305\C,3.129087974
3,1.678546449,-1.9175902346\C,1.8659947714,2.0867296363,-2.3760182007\
C,4.2231156885,2.5147294412,-2.1551135424\C,1.7078527096,3.2889485713,
-3.0420592995\C,4.0755115616,3.7264081085,-2.8252275945\C,2.8113632183
,4.1213111639,-3.2725684686\N,2.2105168714,-0.3555748208,-1.0431275092
\N,4.6653961276,-1.0945729566,-0.131785505\O,2.5496242401,5.2788478856
,-3.9349402321\O,9.846504455,-1.8468596869,1.8117726985\C,3.628441847,
6.1583967071,-4.1985878932\C,-3.5384719342,6.2125057824,4.201296396\C,
-10.4678821922,-3.0074527952,-1.8479534327\C,10.4457933556,-3.12925598
55,1.8743840967\C,-3.614859213,-1.9209016867,-0.1249731404\C,3.6064093
635,-1.9642500213,0.1484669439\O,-3.7574947774,-2.942182363,-0.7649710
731\O,3.7366741528,-2.985542171,0.7910790741\C,-3.2773023407,0.4364650
503,1.2188362088\C,3.2974006118,0.3935853141,-1.2013741883\N,-2.390358

9393,-1.5093755783,0.4091888636\N,2.38697735,-1.5393013752,-0.38677835
21\N,-4.5123076437,0.1455925435,0.8102476853\N,4.5287963571,0.08883300
06,-0.792010546\H,-5.9621403583,-3.4050402893,-0.049256175\H,-8.241806
5304,-3.7466064145,-0.8715211402\H,-8.6139547396,0.463543842,-1.625919
941\H,-6.2983696893,0.8045637677,-0.7744206652\H,0.0044814273,-0.50446
71637,0.0099073067\H,2.1047362684,-4.2339405141,-0.3082018979\H,-0.025
4015852,-5.4422884164,0.0161905018\H,-2.1407524718,-4.2074268222,0.337
4741822\H,-5.1620521168,2.2761998163,1.8261711248\H,-4.8777124411,4.41
49533313,2.9996989828\H,-0.6764839545,3.6272457597,3.4071186834\H,-0.9
767731525,1.4639613908,2.2209568935\H,5.9355442352,-3.476881959,0.0766
353694\H,6.3227295706,0.7301935492,0.7909775942\H,8.2109152692,-3.8439
113725,0.8998221771\H,8.6340250428,0.3633496319,1.6433982454\H,1.00946
66606,1.4462937253,-2.2061586171\H,5.2042777648,2.208808519,-1.8133764
827\H,0.7353743965,3.6100157995,-3.3978519682\H,4.9458369493,4.3478409
954,-2.9923726612\H,4.0982096265,6.5103129144,-3.271701171\H,4.3902719
753,5.684852941,-4.8302172178\H,3.2003006945,7.0096567689,-4.729701886
2\H,-3.1000464885,7.0598756685,4.730236859\H,-4.003959809,6.5677113999
,3.273505593\H,-4.3059692458,5.7498383577,4.834124333\H,-11.463253379,
-2.8432160151,-2.2628682221\H,-9.912687791,-3.6925828151,-2.5005378851
\H,-10.561642744,-3.4566157945,-0.8514322983\H,11.4430869731,-2.976016
4351,2.2888897695\H,9.8823579267,-3.8059389405,2.5287166278\H,10.53409
18888,-3.5820749715,0.8790194139\\Version=ES64L-G09RevD.01\State=1-A\H
F=-2356.6228439\S2=1.043418\S2-1=0.\S2A=0.3524\RMSD=5.945e-09\RMSF=2.0
77e-07\Dipole=0.0030798,0.5105888,-0.0006504\Quadrupole=23.6863672,5.5
092783,-29.1956455,-0.1143584,-3.4793845,-0.0233942\PG=C01 [X(C38H32N8
O6)]\\@

1′-Nm-T

1\1\GINC-GAUSIANDELL\FOpt\UB3LYP\6-31G(2d,p)\C38H32N8O6(3)\PKASZYNSKI\
30-May-2023\0\\#P UB3LYP/6-31G(2d,p) Fopt(tight) geom(noangle, nodista
nce) fcheck #P freq(noraman, readIso) guess=check\\1,3-Bis-[1,3-bis(4-
MeOphenyl)-6-oxoverdazl-5-yl]benzene, triplet\\0,3\N,-2.2036887092,-0.
331116903,1.054704769\N,-4.6667847805,-1.0488813794,0.1495312745\C,-5.
9825631142,-1.2928152994,-0.3599720477\C,-6.7406485117,-0.2031644474,-
0.8051359516\C,-8.02960878,-0.3936381798,-1.270567982\C,-8.5890256457,
-1.6772970027,-1.3024380695\C,-7.8338621433,-2.7648915354,-0.856283316
5\C,-6.5369112289,-2.571283187,-0.3857446068\C,-1.1998939227,-2.275867
1476,0.1788544562\C,-1.2108633723,-3.6734676621,0.1826896384\C,-0.0132
389442,-4.3549755699,-0.0041061045\C,1.1886133557,-3.6803050594,-0.188
5518952\C,1.1863558219,-2.2826856564,-0.1798753811\C,-0.0045905165,-1.
5805751584,0.0006981378\C,-3.100904314,1.7147110816,1.924169295\C,-1.8
330932623,2.1119564004,2.3791387581\C,-1.6621306351,3.314402634,3.0416
271856\C,-2.7571211079,4.1579863648,3.27187794\C,-4.0258487684,3.77408
48142,2.8279036923\C,-4.1863707646,2.5620683305,2.1614091774\O,-2.4830
343084,5.3147334735,3.9306344676\O,-9.8589534032,-1.7581235569,-1.7807
535345\C,5.9750154108,-1.3312764078,0.3624926644\C,6.5214055068,-2.613
2460466,0.3838893375\C,6.7398257523,-0.2478975828,0.8114461135\C,7.817
0938504,-2.8165307595,0.8538256139\C,8.0275431216,-0.4479806448,1.2762
862239\C,8.5789689413,-1.7351937787,1.303763734\C,3.1122641226,1.70195
21265,-1.911414572\C,1.8469787864,2.1086351846,-2.3650750106\C,4.20299
48252,2.5433534977,-2.1456891496\C,1.683541791,3.3143915586,-3.0234337
805\C,4.0500579264,3.7586287612,-2.8080202621\C,2.7837722639,4.1519364
345,-3.2507315293\N,2.2022825712,-0.3412276679,-1.0490182314\N,4.66081
39739,-1.0774163379,-0.1462307327\O,2.5169289006,5.3126230647,-3.90552
05121\O,9.8483344908,-1.8255581841,1.7818647696\C,3.5923162265,6.19737
0039,-4.165762245\C,-3.5528869384,6.2052502569,4.1939901489\C,-10.4707
287308,-3.0348956799,-1.8373892553\C,10.4521579323,-3.1062956232,1.834
138036\C,-3.6172083868,-1.9291402212,-0.1304465218\C,3.6057663834,-1.9
520899529,0.1306690636\O,-3.7581437107,-2.9508254012,-0.7701495704\O,3
.740304658,-2.9768335522,0.7668476476\C,-3.2830818802,0.4294310385,1.2
11881208\C,3.2863978421,0.4131231308,-1.2035410862\N,-2.3918905175,-1.

514226166,0.4011359059\N,2.3830830787,-1.5277269135,-0.3995185995\N,-4
.5177697493,0.1355783587,0.8055223612\N,4.5192090329,0.1101979221,-0.7
981478882\H,-5.9602139025,-3.4195662211,-0.0479704672\H,-8.2407154144,
-3.7675996225,-0.8651769664\H,-8.6249874624,0.4409168654,-1.6226341335
\H,-6.3084841234,0.7885155646,-0.7761764422\H,-0.0012264032,-0.5017325
818,0.002566054\H,2.1097889848,-4.2244905005,-0.3279429091\H,-0.016620
7674,-5.439590852,-0.005984384\H,-2.1354140247,-4.2123801335,0.3202069
251\H,-5.1710786711,2.2646595029,1.8223633028\H,-4.8898319314,4.404333
4325,2.994944202\H,-0.6859380848,3.6270343269,3.394749573\H,-0.9830789
947,1.4628526597,2.2094886945\H,5.9394717124,-3.4567598475,0.043165451
6\H,6.3138344897,0.7465428706,0.7858805786\H,8.2177070833,-3.821772737
4,0.859286314\H,8.6280711096,0.3816403146,1.6312561631\H,0.9929331387,
1.4642503444,-2.1976970057\H,5.185811259,2.2386633453,-1.8076219963\H,
0.7093362909,3.6342993166,-3.3755254705\H,4.9179547917,4.384063023,-2.
9728529041\H,4.0625298148,6.545307628,-3.2376003991\H,4.3546239078,5.7
301843167,-4.8015377109\H,3.1603861988,7.0503494801,-4.6910174523\H,-3
.1156364708,7.0537156084,4.7221464287\H,-4.0209707229,6.559295507,3.26
70622365\H,-4.3180556336,5.7406299514,4.8282067571\H,-11.4674381051,-2
.8735221009,-2.2502085269\H,-9.9153398451,-3.7193317426,-2.4905350783\
H,-10.561114645,-3.4832564265,-0.8401979339\H,11.4498197333,-2.9525422
444,2.2475646608\H,9.8924761791,-3.7895104642,2.4848935947\H,10.539831
089,-3.5517743909,0.8354140605\\Version=ES64L-G09RevD.01\State=3-A\HF=
-2356.6230866\S2=2.046977\S2-1=0.\S2A=2.001224\RMSD=4.949e-10\RMSF=2.1
91e-07\Dipole=0.0015914,0.5126692,0.0009503\Quadrupole=23.7146621,5.55
4817,-29.2694791,-0.050018,-3.5641512,0.0708001\PG=C01 [X(C38H32N8O6)]
\\

1′-Nm-CS

1\1\GINC-LOCALHOST\FOpt\RB3LYP\6-31G(2d,p)\C38H32N8O6\PIOTR\31-May-202
3\0\\#P B3LYP/6-31G(2d,p) Fopt(tight) geom(noangle, nodistance) #P fch
eck freq(noraman, readIso)\\1,3-Bis-[1,3-bis(4-MeOphenyl)-6-oxoverdazl
-5-yl]benzene, singlet 2nd orient\\0,1\N,-2.1374476735,-0.5250194886,1
.3643378651\N,-4.5687431981,-0.9856371728,0.2086329929\C,-5.8534076265
,-1.094266819,-0.3981541515\C,-6.5715348699,0.0837845565,-0.6577387904
\C,-7.8343790483,0.0241020829,-1.2157505832\C,-8.4174820672,-1.2119704
035,-1.5257332597\C,-7.7074196386,-2.3869255263,-1.2636884769\C,-6.434
2167206,-2.3285416285,-0.7039857132\C,-1.1815546451,-2.3860451755,0.28
17872907\C,-1.1999171396,-3.7817760625,0.2861101403\C,-0.0195031957,-4
.4677310013,0.0149809972\C,1.1691226751,-3.7968092117,-0.2578831912\C,
1.1676550343,-2.4009517308,-0.2571298851\C,-0.0027144095,-1.6936642184
,0.0114338926\C,-2.9733155644,1.49407281,2.3449361328\C,-1.7040588194,
1.7992883648,2.8616080643\C,-1.5093785928,2.9208928683,3.6481459011\C,
-2.5820861983,3.7725946114,3.9435562022\C,-3.852481669,3.4788912416,3.
4395314097\C,-4.0367473156,2.3474843765,2.6487335088\O,-2.28646667,4.8
469083047,4.7223873664\O,-9.6596080215,-1.1601111593,-2.0722141519\C,5
.854811784,-1.1640804074,0.4195779932\C,6.4206431048,-2.4045086745,0.7
285747921\C,6.5871498638,0.0058524506,0.6761593957\C,7.6930548069,-2.4
768675741,1.2884446048\C,7.8491878662,-0.067683837,1.234341378\C,8.417
2913176,-1.3099268403,1.5474938481\C,3.0062171592,1.4519062482,-2.3301
672329\C,1.740739467,1.7711407471,-2.8476368044\C,4.0798962127,2.29160
31147,-2.6361307963\C,1.5596372141,2.8930042124,-3.637043665\C,3.90932
63522,3.4231312228,-3.4298226217\C,2.6425708985,3.7309005467,-3.934615
6203\N,2.1459866603,-0.5544064239,-1.3444212509\N,4.5715469254,-1.0414
61401,-0.1875047842\O,2.3599644446,4.8067184114,-4.716196283\O,9.65996
25779,-1.2717086604,2.0938592821\C,3.4167064901,5.6905168272,-5.045978
1639\C,-3.3324284024,5.7442739561,5.0498905894\C,-10.2934459676,-2.382
0574245,-2.4104266732\C,10.2789686554,-2.5003683672,2.4352061859\C,-3.
5488954743,-1.8983794314,-0.0886462212\C,3.5407308606,-1.9410294824,0.
1120920025\O,-3.6843237528,-2.8473606428,-0.831840418\O,3.6646744851,-
2.8896764656,0.8577130129\C,-3.1767166722,0.2953947531,1.4991282226\C,
3.1951077046,0.2530212281,-1.4812931538\N,-2.3437118367,-1.6035011689,

0.5563445306\N,2.3391945792,-1.6332360018,-0.5336693592\N,-4.386071304
8,0.1297061309,0.972736266\N,4.4023764688,0.0740534529,-0.9544607003\H
,-5.8962588016,-3.2435135963,-0.5106702708\H,-8.1324258652,-3.35689564
38,-1.4865857057\H,-8.3924567588,0.9285569296,-1.4292174633\H,-6.12183
29809,1.0373185465,-0.4174120781\H,0.003823386,-0.613403115,0.01005270
26\H,2.0817441045,-4.3412904198,-0.4532448381\H,-0.0260682858,-5.55250
61182,0.0163680839\H,-2.119058872,-4.3146699588,0.4828494269\H,-5.0232
227719,2.1177270849,2.2651028143\H,-4.6997910257,4.1165572626,3.656416
3686\H,-0.5315275308,3.1633161943,4.0486848049\H,-0.8719311851,1.14300
85924,2.6391204981\H,5.8716464966,-3.3133935333,0.537592265\H,6.149018
9898,0.9641420005,0.4333874171\H,8.1062925949,-3.4513376763,1.51382883
02\H,8.4181757906,0.8304927353,1.4455025015\H,0.9007324294,1.125552774
5,-2.6234836242\H,5.0635245275,2.0509037837,-2.2518992226\H,0.58478568
82,3.1462209872,-4.0382156439\H,4.7642887535,4.0499374291,-3.648325405
9\H,3.8567687958,6.145780811,-4.150022677\H,4.2053345594,5.182684578,-
5.6150574513\H,2.9739328149,6.4719295841,-5.6652166613\H,-2.8802194006
,6.5218510744,5.6671357437\H,-3.7669608464,6.202538411,4.1527665109\H,
-4.1271374605,5.247482092,5.6202539959\H,-11.2630811916,-2.1119998782,
-2.8306422687\H,-9.7176278436,-2.9404940066,-3.1585848358\H,-10.445593
7687,-3.0143966962,-1.5271072889\H,11.2518083005,-2.2409932654,2.85474
43565\H,9.6964450148,-3.0498785112,3.1847816541\H,10.4234374184,-3.136
7604799,1.5535095846\\Version=ES64L-G09RevD.01\State=1-A\HF=-2356.5865
406\RMSD=1.111e-09\RMSF=4.930e-07\Dipole=0.0029311,0.4835419,-0.000618
4\Quadrupole=26.7389936,-0.4218094,-26.3171841,-0.1621789,1.7184894,-0
.043521\PG=C01 [X(C38H32N8O6)]\\

2′-C

1\1\GINC-LOCALHOST\FOpt\UB3LYP\6-31G(2d,p)\C22H19N4O3(2)\PIOTR\08-Jul-
2017\0\\#P UB3LYP/6-31G(2d,p) FOpt freq(noraman, ReadIso) SCF=Direct #
P Geom=(NoDistance,NoAngle) fcheck\\1,5-Bis(4MeOphenyl)-3-Ph oxoverdaz
yl, C2 symm\\0,2\O,0.,0.,2.0858154497\C,0.,0.,0.8719207288\N,-0.037158
9072,-1.168078479,0.1042934747\N,-0.0169682363,-1.1867853551,-1.258996
0115\C,0.,0.,-1.858637663\N,0.0169682363,1.1867853551,-1.2589960115\N,
0.0371589072,1.168078479,0.1042934747\C,-0.0048414629,2.4594079017,0.7
224810596\C,0.6790356368,2.7359539322,1.9051886539\C,0.6373269917,4.01
48140818,2.4573239144\C,-0.0868242497,5.0296039745,1.8269330052\C,-0.7
677305885,4.7484144435,0.6357037161\C,-0.7276980282,3.4780735774,0.089
2194621\C,0.,0.,-3.3433026804\C,0.2121046642,1.1888845422,-4.052981431
5\C,0.2117376093,1.1863029263,-5.4440742919\C,0.,0.,-6.1450406024\C,-0
.2117376093,-1.1863029264,-5.4440742919\C,-0.2121046642,-1.1888845422,
-4.0529814314\C,0.0048414629,-2.4594079017,0.7224810596\C,0.7276980282
,-3.4780735774,0.0892194622\C,0.7677305885,-4.7484144435,0.6357037162\
C,0.0868242497,-5.0296039745,1.8269330053\C,-0.6373269917,-4.014814081
8,2.4573239144\C,-0.6790356368,-2.7359539322,1.9051886539\O,0.19260096
26,-6.3063684067,2.2821853591\O,-0.1926009626,6.3063684067,2.282185359
1\C,0.4793194838,6.6444585293,3.4827481133\C,-0.4793194838,-6.64445852
92,3.4827481133\H,-1.2480879848,3.2613333347,-0.8344583959\H,-1.327598
7846,5.5440474456,0.1576535537\H,1.1775576832,4.2030561215,3.375951541
\H,1.2383218947,1.9577865908,2.4026638564\H,-0.3809123031,-2.107035134
5,-3.5039740465\H,-0.3799816392,-2.1138934961,-5.9818303367\H,0.,0.,-7
.2303379828\H,0.3799816392,2.113893496,-5.9818303368\H,0.3809123031,2.
1070351344,-3.5039740465\H,-1.2383218947,-1.9577865908,2.4026638564\H,
-1.1775576832,-4.2030561214,3.3759515411\H,1.3275987846,-5.5440474456,
0.1576535538\H,1.2480879848,-3.2613333347,-0.8344583959\H,0.1151483617
,6.0500744173,4.3298718869\H,1.5641159751,6.5101275848,3.3894213257\H,
0.2623665112,7.6980921796,3.6634828839\H,-0.1151483617,-6.0500744172,4
.329871887\H,-1.5641159751,-6.5101275848,3.3894213258\H,-0.2623665112,
-7.6980921796,3.663482884\\Version=ES64L-G09RevD.01\State=2-A\HF=-1294
.4413849\S2=0.771923\S2-1=0.\S2A=0.750226\RMSD=6.066e-09\RMSF=1.383e-0
5\Dipole=0.,0.,0.7101411\Quadrupole=-11.6570586,7.3681349,4.2889237,5.
5153708,0.,0.\PG=C02 [C2(H1C1C1C1C1O1),X(C18H18N4O2)]\\@

2′-N


```
1\1\GINC-LOCALHOST\FOpt\UB3LYP\6-31G(2d,p)\C22H19N4O3(2)\PIOTR\08-Jul-
 2017\0\\#P UB3LYP/6-31G(2d,p) FOpt freq(noraman, ReadIso) SCF=Direct #
 P Geom=(NoDistance,NoAngle) fcheck\\1,3-Bis(4-MeOphenyl)-5-Ph oxoverda
 zyl, C2 symm\\0,2\O,-0.0297138875,0.050050034,2.0908380317\C,-0.022068
 3409,0.0319098275,0.8772571099\N,-0.0464957141,-1.1486469727,0.1275290
 857\N,-0.0200950485,-1.1903712156,-1.2342296497\C,-0.0160899129,-0.012
 3295275,-1.8562715256\N,-0.0152997549,1.1831569606,-1.2741724917\N,0.0
 088618337,1.1866757048,0.0900860291\C,-0.0395296103,2.4893688301,0.685
 8491803\C,0.645748799,2.7804320673,1.8687546573\C,0.5926966464,4.07080
 2727,2.3880163241\C,-0.1311094575,5.0706880877,1.7420549631\C,-0.80694
 41078,4.7729589635,0.5602036758\C,-0.7640178037,3.4884159503,0.0296277
 069\C,-0.014460382,-0.036254166,-3.336552252\C,0.1606604977,1.14596663
 25,-4.0738115723\C,0.160844004,1.1252703198,-5.4572025664\C,-0.0149836
 846,-0.0818231298,-6.1470912901\C,-0.1898904764,-1.2667285805,-5.42626
 3546\C,-0.1882588005,-1.2344206813,-4.0340619367\C,0.0027680113,-2.429
 10347,0.767691231 9\C,0.7384144605,-3.4517314106,0.1559790713\C,0.78575
 71171,-4.7124432821,0.7238103671\C,0.0994171282,-4.9798576049,1.915179
 935\C,-0.6374970316,-3.9612155454,2.52405926\C,-0.6864193727,-2.692120
 1548,1.9503888478\O,0.2130130584,-6.2480050377,2.3919023056\O,0.000039
 0427,0.0059544099,-7.5033479186\C,-0.4620182425,-6.5718508878,3.594717
 318\C,-0.1725495961,-1.1837539386,-8.253315087\H,-1.2798252553,3.24770
 32256,-0.890455329\H,-1.3754044591,5.5414121903,0.0466598126\H,1.12874
 02645,4.2919770262,3.3051025261\H,1.2055497628,2.0086913197,2.37515482
 95\H,-0.3293245157,-2.1526606804,-3.4770278037\H,-0.3306604729,-2.2124
 569699,-5.9335244685\H,0.2985452515,2.0336434635,-6.0328558124\H,0.301
 5845567,2.0810178042,-3.545751928\H,-1.255955616,-1.9109154714,2.43118
 95784\H,-1.1823470712,-4.1383991241,3.4420979219\H,1.3559283761,-5.510
 9395092,0.2630390007\H,1.2633997215,-3.2455998401,-0.7675224412\H,-0.1
 078767947,-5.9599627833,4.4336166499\H,-1.5475969412,-6.4496554373,3.4
 934648408\H,-0.2359790059,-7.6202056287,3.7942437428\H,-0.1673822767,6
 .0731891532,2.1551517804\H,-1.1440464502,-1.6511953193,-8.05029342\H,-
 0.1285638446,-0.8876555351,-9.3023096031\H,0.6248878261,-1.9089547036,
 -8.0490240016\\Version=ES64L-G09RevD.01\State=2-A\HF=-1294.4420949\S2=
 0.772496\S2-1=0.\S2A=0.750231\RMSD=7.305e-09\RMSF=6.584e-06\Dipole=-0.
 3152534,-0.7101733,-0.2211881\Quadrupole=-11.5753774,4.0430558,7.53232
 16,1.6406296,-0.8949012,-0.4605175\PG=C01 [X(C22H19N4O3)]\\
```


10. References

(1) Jiang, L.; Lu, X.; Zhang, H.; Jiang, Y.; Ma, D. CuI/4-Hydro-L-proline as a more effective catalytic system for coupling of aryl bromides with *N*-Boc hydrazine and aqueous ammonia, *J. Org. Chem.* **2009**, *74*, 4542–4546.

(2) Dumele, O.; Wu, D.; Trapp, N.; Goroff, N.; Diederich, F. Halogen Bonding of (Iodoethynyl)benzene Derivatives in Solution, *Org. Lett.* **2014**, *16*, 4722-4725.

(3) Yu, P.; Lv, H.; Li, C.; Ren, J.; Ma, S.; Xu, S.; Chen, X.; Yu, S. Stereospecific Synthesis and Biological Evaluation of Monodesmethyl Metabo- lites of (+)-13a-(S)-Deoxytylophorinine as Potential Antitumor Agents, *Synthesis* **2012**, *44*, 3757-3764.

(4) Rajca, A.; Olankitwanit, A.; Rajca, S. Triplet ground state derivative of aza-*m*-xylylene diradical with large singlet triplet energy gap, *J. Am. Chem. Soc.* **2011**, *133*, 4750–4753.
(5) Arnatt, C. K.; Adams, J. L.; Zhang, Z.; Haney, K. M.; Li, G.; Zhang, Y. Design, syntheses, and characterization of piperazine based chemokine receptor CCR5 antagonists as anti prostate cancer agents, *Bioorg. Med. Chem. Lett.* **2014**, *24*, 2319-2323.
(6) CrysAlis CCD and CrysAlis RED, O. D., Oxford Diffraction Ltd: Yarnton, 2008.
(7) Sheldrick, G. M. A short history of SHELX, *Acta Cryst. Sect. A* **2008**, *64*, 112–122.
(8) Dolomanov, O. V.; Bourhis, L. J.; Gildea, R. J.; Howard, J. A. K.; Puschmann, H. OLEX2: a complete structure solution, refinement and analysis program, *J. Appl. Cryst.* **2009**, *42*, 339–341.
(9) International Tables for Crystallography, U. S., Ed. Springer, New York, 2006.
(10) Spek, A. L. Structure validation in chemical crystallography, *Acta Cryst. Sect. D* **2009**, *65*, 148–155.
(11) Connelly, N. G.; Geiger, W. E. Chemical redox agents for organometallic chemistry, *Chem. Rev.* **1996**, *96*, 877−910.
(12) Bleaney, B.; Bowers, K. D. Anomalous paramagnetism of copper acetate, *Proc. R. Soc. London, Ser. A.* **1952**, *214*, 451–465.
(13) Stoll, S.; Schweiger, A. EasySpin, a comprehensive software package for spectral simulation and analysis in EPR, *J. Magn. Reson.* **2006**, *178*, 42–55.
(14) Bonner, J. C.; Fisher, M. E. Linear magnetic chains with anisotroyic coupling, *Phys. Rev.* **1964**, *135*, A640–A658.
(15) Gaussian 09, Revision A.02, M. J. Frisch, G. W. Trucks, H. B. Schlegel, G. E. Scuseria, M. A. Robb, J. R. Cheeseman, G. Scalmani, V. Barone, B. Mennucci, G. A. Petersson, H. Nakatsuji, M. Caricato, X. Li, H. P. Hratchian, A. F. Izmaylov, J. Bloino, G. Zheng, J. L. Sonnenberg, M. Hada, M. Ehara, K. Toyota, R. Fukuda, J. Hasegawa, M. Ishida, T. Nakajima, Y. Honda, O. Kitao, H. Nakai, T. Vreven, J. A. Montgomery, Jr., J. E. Peralta, F. Ogliaro, M. Bearpark, J. J. Heyd, E. Brothers, K. N. Kudin, V. N. Staroverov, R. Kobayashi, J. Normand, K. Raghavachari, A. Rendell, J. C. Burant, S. S. Iyengar, J. Tomasi, M. Cossi, N. Rega, J. M. Millam, M. Klene, J. E. Knox, J. B. Cross, V. Bakken, C. Adamo, J. Jaramillo, R. Gomperts, R. E. Stratmann, O. Yazyev, A. J. Austin, R. Cammi, C. Pomelli, J. W. Ochterski, R. L. Martin, K. Morokuma, V. G. Zakrzewski, G. A. Voth, P. Salvador, J. J. Dannenberg, S. Dapprich, A. D. Daniels, O. Farkas, J. B. Foresman, J. V. Ortiz, J. Cioslowski, and D. J. Fox, Gaussian, Inc., Wallingford CT, 2009.

(16) Scott, A. P.; Radom, L. Harmonic Vibrational Frequencies: An Evaluation of Hartree-Fock, Møller-Plesset, Quadratic Configuration Interaction, Density Functional Theory, and Semiempirical Scale Factors, *J. Phys. Chem.* **1996**, *100*, 16502-16513.
(17) Yamaguchi, K. The electronic structures of biradicals in the unrestricted Hartree-Fock approximation, *Chem. Phys. Lett.* **1975**, *33*, 330–335.
(18) Yamaguchi, K.; Takahara, Y.; Fueno, T.; Nasu, K. Ab initio MO calculations of effective exchange integrals between transition-metal ions via oxygen dianions: Nature of the copper-oxygen bonds and superconductivity, *Jpn. J. Appl. Phys.* **1987**, *26*, L1362.
(19) Yamaguchi, K.; Jensen, F.; Dorigo, A.; Houk, K. N. A spin correction procedure for unrestricted Hartree-Fock and Møller-Plesset wavefunctions for singlet diradicals and polyradicals, *Chem. Phys. Lett.* **1988**, *149*, 537–542.
(20) Cossi, M.; Scalmani, G.; Rega, N.; Barone, V. New developments in the polarizable continuum model for quantum mechanical and classical calculations on molecules in solution, *J. Chem. Phys.* **2002**, *117*, 43-54 and references therein.
(21) Constantinides, C. P.; Berezin, A. A.; Manoli, M.; Leitus, G. M.; Bendikov, M.; Rawson, J. M.; Koutentis, P. A. antiferromagnetic, *New J. Chem.* **2014**, *38*, 949-954.
(22) Stratmann, R. E.; Scuseria, G. E.; Frisch, M. J. An efficient implementation of time-dependent density-functional theory for the calculation of excitation energies of large molecules, *J. Chem. Phys.* **1998**, *109*, 8218–8224.
